# Revisiting the genetic diversity of emerging hantaviruses circulating in Europe using a pan-viral resequencing microarray

**DOI:** 10.1038/s41598-019-47508-7

**Published:** 2019-08-27

**Authors:** Claudia Filippone, Guillaume Castel, Séverine Murri, Myriam Ermonval, Misa Korva, Tatjana Avšič-Županc, Tarja Sironen, Olli Vapalahati, Lorraine M. McElhinney, Rainer G. Ulrich, Martin H. Groschup, Valérie Caro, Frank Sauvage, Sylvie van der Werf, Jean-Claude Manuguerra, Antoine Gessain, Philippe Marianneau, Noël Tordo

**Affiliations:** 1 Institut Pasteur, Antiviral Strategies Unit, Department of Virology, Paris, France; 2 Institut Pasteur, Unit of Epidemiology and Physiopathology of Oncogenic Viruses, CNRS, UMR 3569, Department of Virology, Paris, France; 3 CBGP, INRA, CIRAD, IRD, Montpellier SupAgro, Univ Montpellier, Montpellier, France, Montpellier, France; 4 ANSES, Virology Unit, Lyon, France; 5 University of Ljubljana, Microbiology and Immunology Institute, Faculty of Medicine, Ljubljana, Slovenia; 6 Haartman Institute, Department of Virology, Helsinki, Finland; 7 Animal and Plant Health Agency (APHA), Surrey, UK. University of Liverpool, South Wirral, United Kingdom; 8 Friedrich-Loeffler-Institut, Institute for Novel and Emerging Infectious Diseases, Greifswald, Insel Riems Germany; 9 Institut Pasteur, Laboratory for Urgent Response to Biological Threats - CIBU Unit, Paris, France; 10 University of Lyon, UMR- CNRS, 5558 Villeurbanne, France; 11 Institut Pasteur, Unit of Molecular Genetics of RNA viruses, Department of Virology, Paris, France; 12 Institut Pasteur de Guinée, Conakry, Guinea; 13 Present Address: Virology Unit, Institut Pasteur de Madagascar, Antananarivo, Madagascar

**Keywords:** Biological techniques, Genotyping and haplotyping, High-throughput screening

## Abstract

Hantaviruses are zoonotic agents transmitted from small mammals, mainly rodents, to humans, where they provoke diseases such as Hemorrhagic fever with Renal Syndrome (HFRS) and its mild form, Nephropathia Epidemica (NE), or Hantavirus Cardio-Pulmonary Syndrome (HCPS). Hantaviruses are spread worldwide and monitoring animal reservoirs is of primary importance to control the zoonotic risk. Here, we describe the development of a pan-viral resequencing microarray (PathogenID v3.0) able to explore the genetic diversity of rodent-borne hantaviruses endemic in Europe. Among about 800 sequences tiled on the microarray, 52 correspond to a tight molecular sieve of hantavirus probes covering a large genetic landscape. RNAs from infected animal tissues or from laboratory strains have been reverse transcribed, amplified, then hybridized to the microarray. A classical BLASTN analysis applied to the sequence delivered through the microarray allows to identify the hantavirus species up to the exact geographical variant present in the tested samples. Geographical variants of the most common European hantaviruses from France, Germany, Slovenia and Finland, such as Puumala virus, Dobrava virus and Tula virus, were genetically discriminated. Furthermore, we precisely characterized geographical variants still unknown when the chip was conceived, such as Seoul virus isolates, recently emerged in France and the United Kingdom.

## Introduction

Hantaviruses are enveloped tri-segmented negative-stranded RNA viruses belonging to the *Hantaviridae* family, *Bunyavirales* order, according to the 2018 classification of the International Committee on Taxonomy of Viruses (ICTV)^1^. Hantaviruses are distributed worldwide, their animal reservoirs are rodents, insectivores or bats (orders Rodentia, Soricomorpha, Chiroptera). When rodent-borne orthohantaviruses are transmitted to humans through aerosolized excreta of infected rodent reservoirs, they can provoke Hemorrhagic Fever with Renal Syndrome (HFRS), its mild form Nephropathia Epidemica (NE), or Hantavirus Cardio-Pulmonary Syndrome (HCPS), mainly depending on the virus species and its genotype/strain. In Europe, cases of HFRS associated with *Dobrava-Belgrade orthohantavirus* (DOBV) and *Seoul orthohantavirus* (SEOV), and cases of NE associated with *Puumala orthohantavirus* (PUUV), are continuously reported in endemic areas such as the Balkans, the Fennoscandia region, Germany, Belgium, The Netherlands, France, and more recently, United Kingdom^2–8^. Tools to investigate the genetic diversity of hantaviruses in their animal reservoirs along their geographical distribution are particularly needed to better understand the epidemiology and predict hantavirus disease outbreaks in humans, and to set up appropriate public health measures^9–14^.

The recent development of molecular methods without an *a priori* hypothesis has brought a critical benefit in the field of diagnosis and research on infectious diseases. They can permit to obtain the sequence of a pathogen present in a biological sample, in absence of specific probes required for classic PCR or hybridization^15–23^. Among them, Next Generation Sequencing (NGS) have become widely used for identification and whole genome sequencing of novel animal or zoonotic pathogens and metagenome analysis^22,23^. These techniques are more accessible in terms of equipment and cost, but still require a complex downstream bioinformatics analysis which may represent a cumbersome step demanding a specific expertise. On the other hand, resequencing microarray do not demand a sophisticated analysis for interpretation. Indeed, the sequence obtained following hybridization of the amplified genetic material on the chip is used without any intermediate step for both BLASTN enquiry and phylogenetic analysis. Although the random amplification does not allow to specifically concentrate the viral material present in the specimen compared to classic or multiplex PCR assays, the following hybridization step on the resequencing microarray further improves the sensitivity and the specificity of the assay. In addition, the assay has the advantage of tolerating critical divergence levels, up to 20%, permitting a precise characterization from a short stretch of about 20 detected nt^20^, that can hardly be achieved when few reads are obtained following NGS assays. Resequencing microarray has been broadly used over the last ten years, for the detection and identification of emerging agents, such as monkeypox virus^17,18^, pandemic influenza viruses^19^, hemorrhagic fever viruses^20^, rhabdoviruses^21^. Several steps of the assay can be performed in the field (mobile laboratory) including sample preparation, random amplification by Phy29 (stable at room temperature) up to hybridization on the microarray. A specific equipment (fluidic station/scanner) is only required for the post-hybridization analysis and scanning.

PathogenID is a collaborative effort of teams of Institut Pasteur that combined expertise to develop 3 generations of resequencing microarrays for the detection of different emerging pathogens, bacteria and viruses, during public health urgencies and for research studies. The 1^st^ and 2^nd^ generations of the microarray contained only a limited number of viral sequences, 46 (PathogenID v1.0) and 126 (PathogenID v2.0), respectively. In particular, only sequences of six prototype orthohantaviruses, mainly associated with human diseases, were included: PUUV, DOBV, SEOV, *Hantaan orthohantavirus* (HTNV), *Sin Nombre orthohantavirus* (SNV), *Andes orthohantavirus* (ANDV)^17,20^. The targeted sequence corresponded to part of the large (L) segment coding for the RNA-dependent RNA-polymerase (RdRp), reputed to be the most conserved region of the genome^24^. While PathogenID v2.0 has been validated for detection of DOBV, SEOV, HTNV, ANDV and SNV in infected Vero E6 cells, it was not for PUUV, yet the most commonly circulating orthohantavirus in Europe^20^.

Therefore, the 3^rd^ generation resequencing microarray (PathogenID v3.0), employed in the present cooperative study at the European scale has been specialized for virus detection and contains more than 800 viral sequences. This pan-viral resequencing microarray covers many viral pathogens critical for both animal and public health and in particular most of the known circulating species and variants of zoonotic viruses. Among those sequences, 52 were strategically chosen to cover the diversity of hantaviruses, in particular the most frequent rodent-borne species that are the only one reported today to have a zoonotic potential. The small (S) segment of the genome encoding the nucleocapsid (N) protein was chosen as target to design the probes since there are more efficient to discriminate variants within a species; in addition more reference sequences were present in GenBank at the moment of the pan-viral chip conception.

The present manuscript illustrates the use of the PathogenID v3.0 resequencing microarray to map the genetic diversity of several endemic hantaviruses mainly associated with human disease in Europe, such as PUUV, DOBV, including genotypes Dobrava and Saarema^25^, TULV, SEOV, Topografov hantavirus (TOPV) according to their geographical distribution.

## Methods

### Design of PathogenID v3.0 pan-viral resequencing microarray

The objective for the conception of the 3^rd^ generation resequencing microarray (PathogenID v3.0) was to reach the widest coverage in virus diversity of both medical and veterinarian interest. Due to the technical limits of the microarray, we selected the minimum number of probes for each viral species/variant, in order to include the highest number of sequences. For each viral family, the included probes were chosen taking into account the reference sequences published in GenBank and our experience from the earlier generations of the microarray (PathogenID v1.0 and PathogenID v2.0)^17–21^.

PathogenID v3.0 contains 838 sequences including virus prototypes and their variants belonging to different families (complete list available upon request to the authors). Regarding hantaviruses, 52 N protein encoding S segment partial sequences were included (Table 1; Supplement 1), according to the known ICTV taxonomy in force when we designed the chip (e.g. choice of the probe sequences). The length of the tiled sequences was: i) 425 nucleotides (nt) for those available in GenBank, with the exception of seq234 (251 nt); ii) 303 nt for those sequenced in the laboratory during previous analyses and not published at the moment of the study (Table 1; Supplement 1).

**Table 1:** List of 52 hantavirus sequences fixed on the panviral chip PathogenID v3.0.

Family *Hantaviridae* (Species)	Isolate/Variant/strain Description*	GenBank Accession number	Sequence size (N segment size: nt-nt position)^§^	Position chip^#^
Puumala orthohantavirus	CG14444	AJ277075	425 (1837: 395–819)	222
	CG 13891	U22423	425 (1847: 395–819)	223
	Bavaria CG9/04	AY954722	425 (1722: 392–816)	224
	Sotkamo NC 005224	X61035	425 (1830: 395–819)	225
	Puu/Kazan_	Z84204	425 (1826: 395–819)	226
	Umea/hu	AY526219	425 (1829: 395–819)	227
	PUU/Ernstbrunn/Cg641/1995	AJ888752	425 (1852: 395–819)	228
	Berkel	L36943	425 (1068: 224–648)	229
	Couvin/59Cg/97	AJ277034	425 (1839: 395–819)	230
	Pallasjarvi/63Cg/98	AJ314597	425 (1827: 395–819)	231
	Munga/Mg16/05	GQ339487	425 (1854: 395–819)	232
	PUU/Mignovillard/CgY02/2005	AM695638	425 (1851: 395–819)	233
	France/Perpignan1999	AY101391	251 (251: 1–251)°	234
	Fusong 900–06	EF488806	425 (1302: 353–777)	235
	CH-214 Franche Comté	KT247596^^^	425 (1302: 353–777)	236
	AR-21 Charleville Meziéres	unpub^^^	425 (1775: 364–786)	237
	167-2 Charleville Meziéres	unpub^^^	303 (1302: 352–654)	238
	167-4 Charleville Meziéres	unpub^^^	303 (1302: 352–654)	239
	178-2 Charleville Meziéres	unpub^^^	303 (1302: 352–654)	240
	180-78 Charleville Meziéres	unpub^^^	303 (1775: 362–664)	241
	RU-11 Ruminy Troyes	KY364996^^^	303 (1785: 362–664)	242
	OR-52 Orleans	KT247595^^^	303 (1302: 352–654)	243
Sin Nombre orthohantavirus	Convict Creek 107	L33683	425 (2083: 395–819)	244
Dobrava-Belgrade orthohantavirus	Dobrava-Belgrade	L41916	425 (1670: 388–812)	245
	Esl/81Aa/01	AY533120	425 (1697: 388–812)	246
Saaremaa orthohantavirus	Saaremaa/Lolland/Aa1403/2000	AJ616854	425 (1673: 388–812)	247
Kenkeme orthohantavirus	MSB148794	GQ306148	425 (1640: 403–827)	248
Hantaan orthohantavirus & unclassified hantaviruses	1980471	M14626	425 (1696: 389–813)	249
	KY	GU140098	425 (1699: 389–813)	250
	Z10	EF533944	425 (1701: 389–813)	251
	AA1719	AF427319	425 (1643: 366–790)	252
	CGHu1	EU092218	425 (1681: 389–813)	253
	Q32	AB027097	425 (1635: 353–777)	254
Seoul orthohantavirus	Z37	AF187082	425 (1754: 395–819)	255
Hantaan orthohantavirus	Nc167	AB027523	425 (1654: 354–778)	256
Seoul orthohantavirus	L99	AF488708	425 (1290: 353–777)	257
Seoul orthohantavirus	80-39	NC005236	425 (1769: 395–819)	258
Asama orthohantavirus	N10	EU929072	425 (1710: 371–795)	259
Nova mobatvirus	MSB95703	FJ539168	425 (1839: 405–829)	260
Prospect Hill orthohantavirus	PHV 1980485	M34011	425 (1675: 395–819)	261
Soochong virus (unclass.)	SC-1	AY675349	425 (1695: 388–812)	262
Tula orthohantavirus	Sennickerode Sen05/204	EU439950	425 (1700: 395–819)	263
	Tula/Kosice144/Ma/95	Y13979	425 (1833: 395–819)	264
	Tula/Moravia/5293 Ma/94	Z48574	425 (1832: 395–819)	265
	MG23/Omsk	AF442621	425 (1758: 353–777)	266
Thottapalayam thottivirus	Thottapalayam 1980493	AY526097	425 (1530: 450–874)	267
Andes orthohantavirus	Chile-9717869	AF291702	425 (1871: 395–819)	268
	NK104619	EU241691	425 (927: 189–613)	269
Sin Nombre orthohantavirus	NM R11	L37904	425 (2060: 395–819)	270
New York orthohantavirus	RI-1 44755	U09488	425 (2078: 395–819)	271
Choclo virus orthohantavirus	169173	DQ285046	425 (1972: 395–819)	272
Laguna Negra orthohantavirus	510B 1980476	AF005727	425 (1904: 395–819)	273

All sequences are partially targeting the S segment encoding the hantavirus nucleocapsid (N) protein. Complementary information is found in Supplement 1.

^*^Description of the isolate/strain as indicated in GenBank or according to laboratory nomenclature for experimentally obtained sequences.

^§^Position according to the corresponding sequence available in GenBank.

^°^Sequence corresponding to a different region of the S gene.

^#^The number is referred to the position on the pan-viral chip PathogenID v3.0 (total number: 838 sequences).

^^^unpublished at the moment of the study.

Upon sequence selection, PathogenID v3.0 was manufactured by Affymetrix (Santa Clara, California) according to their high density resequencing approach, based on the use of stepwise overlapping 25 nt long probes, the first covering position 1–25 of the tiled sequence, the second position 2–26, etc.: each probe comprises a set of 4 different alleles differing in the central 13th position (e.g. A, C, G or T)^16,17^. A total of 2.5 millions of 25 mer oligonucleotides were tiled on the microarray PathogenID v3.0.

### Hantavirus plasmids

Synthetic plasmids containing the S segment of PUUV, the most endemic hantavirus in Europe (strain Sotkamo 2009), HTNV (strain 76/118), TULV (strain Moravia) were used in a preliminary assay to evaluate the potential of the microarray for hantavirus detection, prior testing the field animal samples. PUUV plasmid was taken as reference and tested alone and in pool with the HNTV and TULV plasmids.

### Laboratory strains

**Figure 1:**
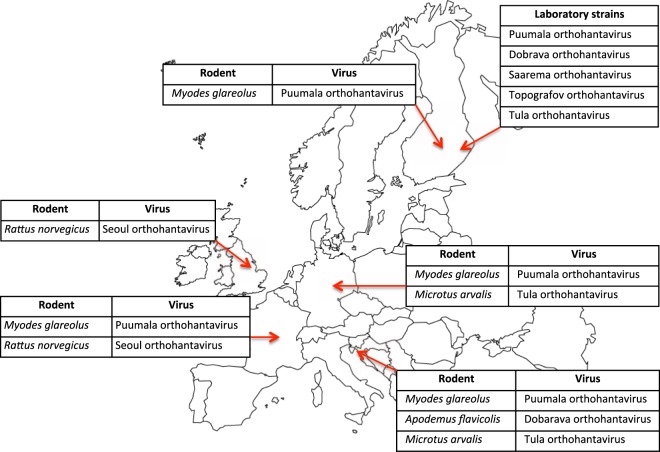
Cooperative work for genetic characterization study of endemic hantaviruses in Europe. Hantaviruses from rodents: Bank vole (*Myodes glareolus*), Common vole (*Microtus arvalis*), Yellow-necked mouse (*Apodemus flavicolis*), Striped field mouse (*Apodemus agrarius*), and Norway rat (*Rattus norvegicus*) captured in different endemic areas in Europe were genetically characterized using a resequencing pan-viral chip during a cooperative work among European research institutes: France, Germany, Scandinavia, Balkans, United Kingdom. Laboratory viral strains were also obtained from one of the participant laboratory. The tested RNA included: (i) RNA extracted from tissues (e.g. lung) originating from infected wild rodents; (ii) RNA extracted from supernatants from inoculated cells (Vero E6).

Laboratory prototype strains of PUUV, DOBV (genotypes Dobrava and Saaremaa) TULV and TOPV isolated on Vero E6 cells in BSL3 containment were provided by the virology participant laboratory in Finland^26–30^ (Fig. 1).

### Endemic hantaviruses from wild rodents captured in Europe

Hantavirus rodent reservoirs of the Bank vole (*Myodes glareolus)*, Common vole (*Microtus arvalis)*, Yellow-necked mouse (*Apodemus flavicolis)*, Striped field mouse (*Apodemus agrarius)*, and Norway rat (*Rattus norvegicus)* were previously captured in different hantavirus endemic areas in Europe: France, Germany, Finland, Slovenia, and United Kingdom^30–41^. Figure 1 illustrates the capture areas and rodent species as well as the Institutes participating to the study and providing positive animals.

Lung samples were kept at −80 °C in RNAlater (Ambion, Thermo Fisher Scientific, Waltham, USA) prior to RNA extraction (QiaAmp Viral RNA Kit, Qiagen, Hilden, Germany). Hantavirus positive RNAs, routinely confirmed by the reference laboratories by RT-PCR^30–41^, were employed for exploring their genetic diversity by resequencing microarray.

#### France

PUUV, the most frequent hantavirus circulating in France, was obtained from bank voles samples captured in France in 2011 in the Ardennes region^31^, SEOV (Lyon strain) from Norway rats^39^.

#### Finland for Fennoscandia region

Lung tissues originating from PUUV-positive bank voles captured in Konnevesi in 2008 were used^30^.

#### Germany

PUUV and TULV RNAs originating from lungs of animal reservoirs captured across Germany^32–36^ were used.

#### Balkan region

PUUV, TULV, DOBV RNAs originating from lungs were obtained from rodents captured in Slovenia^37,38^.

#### United Kingdom

SEOV (Cherwell strain) obtained from organs of domestic rats in United Kingdom (UK) was used^40,41^.

All experiments were performed in accordance with relevant guidelines and regulations. RNAs extracted from tissue sample from wild animals were received from various Hantavirus Reference Centers in Europe (Fig. 1) through the EU program EVA (European Virus Archive - n° 228292) which facilitates access to virus/tissue library under MTA (Material Transfer Agreement). All of them have been previously published in peer-reviewed literature. All handling procedures of captured rodents followed the regulations of each respective country. The species studied are not protected and all efforts were made to minimize animal suffering.

### Genetic detection and phylogeographical characterization of hantaviruses by the pan-viral chip PathogenID v3.0

The experimental procedure for hantavirus typing includes the following steps, detailed in previous works^19,20^ (Supplement 2).

#### RNA extraction

RNA was extracted using the QIAamp Viral RNA Extraction kit (Qiagen) from both animal organ homogenates or cell supernatants. cDNA was synthetized by Superscript III system (Invitrogen, Thermo Fisher).

#### Random amplification

Genetic material, either plasmid DNA, or cDNA from animal organs or cell supernatants, was amplified by WGA (Whole Genome Amplification) and WTA (Whole Transcriptome Amplification) approaches, respectively, using ϕ29 polymerase-mediated random amplification (Qiagen), followed by a ligation step^17^.

#### Hybridization on the microarray and sequence detection

Amplified products were hybridized overnight at 45 °C on the microarray PathogenID v3.0 after fragmentation and labeling using GeneChip Resequencing Reagent Kit (Affymetrix). Chips were then subjected to washing, fluorescence detection and scanning using the Affymetrix equipment (Wash Control, Scan Control). Resequencing analysis was performed using the software GSEQ. 4.1 (Affymetrix). For each of the 52 hantavirus sequences fixed on the chip (positions seq222 to seq273), an output sequence was obtained in a .txt format, with determined (A, G, T or C) or non-determined (N) positions (example in Supplement 2). Significant sequences obtained were used for Call Rate calculation and BLASTN analysis.

#### Call Rate calculation

Call Rate (CR) was calculated as the ratio (%) between the number of determined (*‘called’*) nucleotides (e.g. A, G, T, C) following hybridization and the total number of nucleotides for each tiled sequence (e.g. 425, 251 or 303 nt, for hantaviruses).

#### BLASTN analysis

For samples presenting a minimum stretch of informative sequence^20^, the entire raw sequence was submitted to BLASTN (Basic Local Alignment Search Tool Nucleotide) query [National Center for Biotechnology Information, National Institute of Health], to interrogate all the sequences present in GenBank. In case of positive BLASTN result, scores, identity (%), query coverage (%) and e-value were taken into account.

### Phylogenetic analysis

Phylogenetic analysis was performed for PUUV (as representative of European orthohantavirus) by using (i) the reference sequences (S segment) available on GenBank, (ii) the sequences tiled on the chip, (iii) the sequences corresponding to the tested hantaviruses, when known.

Firstly, a phylogenetic tree was constructed with the complete coding part of reference sequences by the maximum-likehood method (ML) with PhyML v3.0, implemented in Seaview (v.4.6.1) under the most appropriate nucleotide substitution model as determined by SMS program (available online at http://www.atgc-montpellier.fr/sms/)^42^. Branch supports were evaluated by approximate likelihood-ratio test (aLRT SH-Like). Then, short sequences of the chips and of tested hantaviruses were placed in this backbone tree using RAxML available online on the CIPRES portal at (http://www.phylo.org). Branch supports of these phylogenetic placements were evaluated by the rapid bootstrap procedure with MRE-based Bootstopping criterion as highly recommended on the online software version.

For each sample giving a positive result following hybridization, BLASTN analysis of the resulting sequence, was pointed out on the phylogenetic tree, and compared to the sequences having permitted the detection and genetic characterization.

## Results

### Initial validation of the 3^rd^ generation pan-viral resequencing microarray for hantavirus detection and genetic characterization

The performance in hantavirus detection and genetic characterization of the resequencing chip Pathogen ID v3.0 was first evaluated using plasmids encoding prototype hantavirus sequences (PUUV, HTNV, TULV). The PUUV plasmid encompassing the N protein coding region (1831 nt) of the reference Sotkamo strain 2009 was hybridized to Pathogen ID v3.0 which includes 22 PUUV S segment sequences (seq222 to seq243) (Table 1; Supplement 1). For each of these sequences, Table 2 summarizes (i) the calculated sequence similarity (%) with the tested PUUV Sotkamo strain sequence; (ii) the percentage of correctly identified nucleotides (CR) following its hybridization to the chip and (iii) the result of the BLASTN analysis performed with the obtained raw sequences (Supplement 3).

**Table 2:** Validation of the Pathogen ID v3.0 resequencing microarray using a nucleocapsid (N) protein encoding plasmid of the PUUV prototype strain Sotkamo.

PUUV sequence on the chip (position)	Sequence similarity to PUUV Sotkamo (nbr identical nt/total nbr nt) - (%)	BLASTN identification	Call Rate (CR) (%)
seq222	344/422 - 81.5	yes: Sotkamo	23.9
seq223	335/422 - 79.4	no	23.7
seq224	346/425 - 81.4	no	18.9
seq225*	425/425* - 100*	yes: Sotkamo	97.7
seq226	351/425 - 82.6	no	26.2
seq227	357/424 - 84.2	yes: Sotkamo	39.6
seq228	346/423 - 81.8	yes: Sotkamo + FIN variants	22.7
seq229	346/425 - 81.4	no	19.4
seq230	347/422 - 82.2	yes: Sotkamo	33.9
seq231	387/423 - 91.5	yes: Sotkamo	75.0
seq232	353/424 - 83.2	yes: Sotkamo + FIN variants	34.4
seq233	342/424 - 80.7	yes: Sotkamo + FIN variants + others^°^	19.7
seq234^#^	^#^	yes: Sotkamo	62.1
seq235	323/422 - 76.5	no	7.0
seq236	343/424 - 80.9	yes: Sotkamo + FIN variants + others^°^	19.9
seq237	348/423 - 82.3	yes: Sotkamo	35.2
seq238	244/299 - 81.6	no	24.7
seq239	247/299 - 82.6	yes: Sotkamo	34.4
seq240	249/299 - 83.3	yes: Sotkamo	38.3
seq241	242/299 - 80.9	no	26.9
seq242	245/299 - 81.9	yes: Sotkamo + FIN variants + others^°^	30.8
seq243	246/301 - 81.7	no	25.1

**Figure 2:**
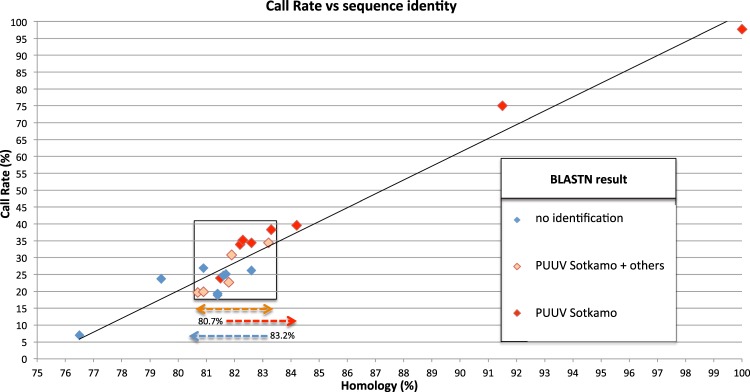
Performance of the Pathogen ID v3.0 resequencing microarray to detect and identify hantavirus sequences. A plasmid encoding the nucleocapsid (N) protein of the PUUV prototype strain Sotkamo was used to evaluate the range of detection of the Pathogen ID v3.0 resequencing microarray. This figure compares the calculated sequence identity (%) between the tested sequence (PUUV Sotkamo) and each PUUV sequences tiled on the microarray with the respective Call Rate (CR, i.e. % of determined nucleotides) obtained after hybridization. Colours, values and arrows outline a window of sequence identity (%) for BLASTN results obtained from each output sequence following hybridization: from no detection/identification (blue), to general PUUV characterization (Sotkamo + others, orange) to precise and exclusive characterization (Sotkamo, red). Complementary information is described in Table 2 and Supplements 3–4.

A plasmid containing the N protein encoding sequence of the PUUV Sotkamo strain was used to evaluate the range of detection of the Pathogen ID v3.0 resequencing microarray. Sequence identity (%) was calculated by aligning the tested Sotkamo PUUV sequence against each PUUV sequence tiled on the array (seq222–seq243). Following hybridization of the Sotkamo plasmid on the chip, each significant output sequence was evaluated by its Call Rate (CR) value (% of the correctly determined nucleotides of the sequence following hybridization) and the result(s) of its analysis by BLASTN. Complementary information is given in Supplement 3 and Fig. 2.

^*^Sotkamo sequence tiled on the chip.

^#^different region of the S segment tiled on the chip.

^°^others: PUUV variants other than Sotkamo and Finnish (FIN) variants.

It clearly appeared that the CR values were proportional to the sequence similarity between the tested sequence and the tiled ones (Table 2). From 100% (seq225, Sotkamo itself) to 91.5% (seq231) of identity, the CR was very high (97.7% to 75%, resp.) and the BLASTN identified the Sotkamo strain without ambiguity. Down to 82.2% of sequence identity (seq230), the CR remained above 34%, still designing Sotkamo in priority by BLASTN, although some tiled sequences already hesitated in precise identification (seq232) or even failed in identification (seq226). Down to 82% of homology, the CR decreased dramatically and the tested sample failed to be identified in some cases. Plotting of the sequence identity to the CR confirmed these observations (Fig. 2): below 80.7% of identity between the tested and the tiled sequences the microarray becomes inefficient for specific detection; the window between 80.7% and 83.2% of identity is critical and versatile between no detection, detection with unspecific determination, and precise genetic characterization of the tested sequence; higher than 83.2% of identity, the microarray identifies precisely the tiled sequence.

We verified that simultaneous detection was possible when plasmids containing the N protein coding region of the three hantavirus reference strains (PUUV Sotkamo 2009, TULV Moravia, HTNV 76/118) were mixed in pool (Supplement 3–4). The CR values of PUUV output sequences (seq222–seq243) were even higher (e.g. more determined nucleotides) for the pool of the three viruses, most likely due to cross-contribution from the three viruses in hybridizing the same sequence tiled on the chip, thus producing better results in BLASTN analysis (Supplements 3–4).

### Mapping genetic diversity of hantaviruses circulating in Europe

The same technology validated with plasmids encoding hantavirus N protein was applied to tissue samples of PUUV, TULV, DOBV, SAAV, SEOV, TOPV infected rodents originating from different endemic areas in Europe as well as to supernatants of cells infected with laboratory strains (Tables 3–7; Supplement 5). Using RNA extracted from lung and liver of PUUV RNA positive bank voles captured in the Ardennes region of France in 2011^31^ we first observed that, at comparable viral RNA titer (Ct value), lung was more performing for hantavirus investigation on the chip (data not shown). Therefore, lung derived RNAs were priorized for further investigation.

**Table 3:** Detection and genetic characterization of Puumala orthohantaviruses (PUUV) from various endemic area in Europe: PUUV isolates or laboratory strain were hybridized to the hantavirus sequences tiled on the PathogenID v3.0 resequencing microarray (seq222 to seq273; Table 1; Supplement 1). For each tested sample, the tiled sequences giving a significant signal are listed and the Call Rate (CR, i.e. % of correctly determined nucleotides) indicated. The corresponding raw sequences were subjected to BLASTN enquiry. The number (nbr) of BLASTN identical results is indicated; the most related sequence is indicated in italics and a maximum of the first 3 closer sequences are listed. “Total Score” evaluates the overall quality of the alignment by BLASTN. Complementary data including all sequences obtained following BLASTN analysis and further details are described in Supplement 5.

Tested virus (geographical origin)	Tiled sequence allowing detection (position on the chip)	BLASTN identification		Score (total)	Call rate (%)
		nbr	sequences		
PUUV 87 (France)	222: PUUV CG14444	3	*Ardennes_2011_87;* Ardennes/Mg75/2011; Ardennes/Mg156/2011	226	65.6
	223: PUUV CG13891	1	*Ardennes_2011_87*	89.1	35.9
	230: Couvin/59Cg/97	3	*Ardennes_2011_87*; Ardennes/Mg75/2011; Ardennes/Mg156/2011	351	73.8
	237: AR-21 Charlesville_Mezieres	3	*Ardennes_2011_87*; Ardennes/Mg75/2011; Ardennes/Mg156/2011	268	67.8
	238: 167-2 Charlesville-Mezieres	6	*Ardennes_2011_87*; Ardennes/Mg75/2011; Ardennes/Mg156/2011	108	52.7
	239: 167-4 Charlesville-Mezieres	3	*Ardennes_2011_87*; Ardennes/Mg75/2011; Ardennes/Mg156/2011	288	78.5
	240: 178-2 Charlesville-Mezieres	3	*Ardennes_2011_87*; Ardennes/Mg75/2011; Ardennes/Mg156/2011	268	77.4
	241: 180-78 Charlesville-Mezieres	3	*Ardennes_2011_87*; Ardennes/Mg75/2011; Ardennes/Mg156/2011	239	73.8
PUUV 153 (France)	222: PUUV CG14444	3	Ardennes_2011_87; Ardennes/Mg75/2011; Ardennes/Mg156/2011	488	83.0
	223: PUUV CG13891	3	Ardennes_2011_87; Ardennes/Mg75/2011; Ardennes/Mg156/2011	141	52.3
	229: Berkel_L36943	28	Ardennes_2011_87; Ardennes/Mg75/2011; Ardennes/Mg156/2011	44.6	26.4
	230: Couvin/59Cg/97	3	Ardennes_2011_87; Ardennes/Mg75/2011; Ardennes/Mg156/2011	581	89.5
	234: Perpignan 1999	1	Ardennes/Mg156/2011	150	64.3
	237: AR-21 Charlesville_Mezieres	3	Ardennes_2011_87; Ardennes/Mg75/2011; Ardennes/Mg156/2011	547	87.5
	238: 167-2 Charlesville-Mezieres	3	Ardennes_2011_87; Ardennes/Mg75/2011; Ardennes/Mg156/2011	156	68.1
	239: 167-4 Charlesville-Mezieres	3	Ardennes_2011_87; Ardennes/Mg75/2011; Ardennes/Mg156/2011	392	88.9
	240: 178-2 Charlesville-Mezieres	3	Ardennes_2011_87; Ardennes/Mg75/2011; Ardennes/Mg156/2011	408	91.0
	241: 180-78 Charlesville-Mezieres	3	Ardennes_2011_87; Ardennes/Mg75/2011; Ardennes/Mg156/2011	345	84.2
PUUV Mu557 Gilserberg/10 (Germany)	223: PUUV CG13891	27	*Mu557Gilserberg/10*	44.6	33.4
	230: Couvin/59Cg/97	1	*Mu557Gilserberg/10*	121	34.2
	233: Mignovillard/CgY02/2005	1	*Mu557Gilserberg/10*	153	35.4
	234: Perpignan1999	2	PUUV/NL/Mg591/2008; Heidelberg/hu	80.6	54.2
	236: CH-214_Franche_Comté	1	*Mu557Gilserberg/10*	57.2	28.7
	237: AR-21_Charlesville_Mezieres	1	*Mu557Gilserberg/10*	73.4	32.4
	238: 167-2Charleville_Mezieres	1	*Mu557Gilserberg/10*	57.2	41.2
	239: 167-4Charleville_Mezieres	1	*Mu557Gilserberg/10*	57.2	38.0
	240: 178-2Charleville_Mezieres	7	*Mu557Gilserberg/10*	53.6	40.9
	241: 180-78Charleville_Mezieres	1	*Mu557Gilserberg/10*	90.9	41.6
	242: RU-11Ruminy-Troies	2	*Mu557Gilserberg/10*; H290 Fulda/10	64.4	30.8
PUUV Mu2232 Bramsche/09 (Germany)	224: Bavaria CG9/04	34	*Mu2232Bramsche/09*	60.8	31.2
	229: Berkel_L36943	6	KS10/3078; KS14/873; KS14/715	158	39.7
	233: Mignovillard/CgY02/2005	2	KS10/3078; KS14/778	94.5	28.4
	234: Perpignan 1999	2	Mu/07/1219; Mu362Osnabrueck/05	48.2	41.0
	242: RU-11RuminyTroyes	11	*Mu2232Bramsche/09*	60.8	30.5
	243: OR-52_Orleans	42	*Mu2232Bramsche/09*	44.6	30.1
PUUV Mu978 Weissach/10 (Germany)	222: CG14444	6	*Mu978Weissach/10*	129	42.4
	224: Bavaria CG9/04	3	*Mu978Weissach/10*	172	48.6
	234: Perpignan1999	1	Heidelberg/hu	132	59.5
	240: 178-2Charleville-Mezieres	6	*Mu978Weissach/10*	89.7	45.5
	241: 180-78Charleville-Mezieres	6	*Mu978Weissach/10*	87.8	40.1
	242: RU-Ruminy-Troyes	6	*Mu978Weissach/10*	75.2	39.4
PUUV laboratory strain Sotkamo	222: CG14444	2	*Sotkamo 2009; Puumala virus genomic RNA for nucleocapsid protein*	51.8	23.9
	225: Sotkamo_NC_005224	2	*Sotkamo 2009; Puumala virus genomic RNA for nucleocapsid protein*	673	96.5
	227: Umea	2	*Sotkamo 2009; Puumala virus genomic RNA for nucleocapsid protein*	51.8	28.9
	228: Ernstbrunn/Cg641/1995	6	*Sotkamo 2009*	69.8	22.7
	229: Berkel_L36943	10	*Sotkamo 2009*	44.6	21.2
	230: Couvin/59Cg/97	2	*Sotkamo 2009; Puumala virus genomic RNA for nucleocapsid protein*	50.0	30.4
	231: Pallasjarvi/63Cg/98	1	*Puumala virus N and Ns genes*, *segment S*, *genomic RNA*, *Sotkamo2009*	361	70.1
	232: Munga/Mg16/05	5	*Puumala virus N and Ns genes*, *segment S*, *genomic RNA*, *Sotkamo2009*	68.0	27.4
	233: Mignovillard/CgY02/2005	3	*Puumala virus N and Ns genes*, *segment S*, *genomic RNA*, *Sotkamo2009*	51.8	15.5
	234: Perpignan1999	2	*Sotkamo2009; Puumala virus genomic RNA for nucleocapsid protein*	64.4	50.2
	236: CH-14_Franche_Comté? Jura	3	*Sotkamo2009*	55.4	18.9
	237: AR-21_Charleville_Mezières	2	*Sotkamo 2009; Puumala virus genomic RNA for nucleocapsid protein*	46.4	31.2
	239: 167-4Charleville_Mezières	2	*Sotkamo 2009; Puumala virus genomic RNA for nucleocapsid protein*	51.8	31.2
	240: 178-2Charleville_Mezières	2	*Sotkamo 2009; Puumala virus genomic RNA for nucleocapsid*	44.6	30.1
	242: RU-11Ruminy-Troyes	3	*Sotkamo 2009*	60.8	23.3
PUUV 335 Konnevesi (variant A) (Finland)	225: Sotkamo_NC_005224	1	*Puumala virus strain PUUV/Konnevesi/Mg_O78A/2005 segment S*	112	44.4
	231: Pallasjarvi/63Cg/98	2	*PUUV/Konnevesi/Mg_O78A/2005*	127	38.6
	234: Perpignan1999	48	*PUUV/Konnevesi/Mg_O78A/2005 segment S*	46.4	25.5
	242: RU-11Ruminy-Troyes	5	*Puumala virus strain PUUV/Konnevesi/Mg_O78A/2005 segment S*	55.4	13.6
PUUV Slovenia 8098 (Slovenia)	225: Sotkamo_NC_005224	37	Puumala virus isolate HtSi_339_p2012 nucleocapsid protein gene, partial	53.6	16.2
	226: Kazan_Z84204	19	Puumala virus isolate HtSi_293_p2010 nucleocapsid protein gene, partial	44.6	12.7
	228: Ernstbrunn/Cg641/1995	1	Puumala virus isolate HtSi_293_p2010 nucleocapsid protein gene, partial	167	55.9
	231: Pallasjarvi/63Cg/98	36	Puumala virus isolate HtSi_339_p2012 nucleocapsid protein gene, partial	73.4	28.2
	233: Mignovillard/CgY02/2005	41	Puumala virus isolate HtSi_293_p2010 nucleocapsid protein gene, partial	48.2	20.4
	234: Perpignan1999	5	Puumala virus mRNA for nucleocapsid protein (N gene), strain Balkan-2	109	58.6
	242: RU-11Ruminy-Troyes	7	Puumala virus isolate HtSi_293_p2010 nucleocapsid protein gene, partial	50.0	24.0

**Table 4:** Detection and genetic characterization of Dobrava orthohantaviruses (DOBV) from various endemic area in Europe: DOBV isolates or laboratory strains were hybridized to the hantavirus sequences tiled on the PathogenID v3.0 resequencing microarray (seq222 to seq273; Table 1; Supplement 1).

Tested virus (geographical origin)	Tiled sequence allowing detection (position on the chip)	BLASTN identification		Score (total)	Call rate (%)
		nbr^°^	sequences		
DOBV-laboratory strain	245: Belgrade L41916	1	DOBV/Ano-Poroia/Afl9/1999	293	70.8
Saaremaa laboratory strain	245: Belgrade L41916	1	*DOB/Saaremaa/160 V*	137	44.1
	247: Saaremaa virus AJ616854	1	*DOB/Saaremaa/160 V*	145	48.4
	256: Hantavirus NC167 AB027523	9	*DOB/Saaremaa/160 V*	50.0	17.9
DOBV 15/01 (Slovenia)	245: DOBV Belgrade L41916	2	Dobrava-Belgrade virus isolate HtSi_275_p2008; HtSi_1036_a2001	688	97.5
	247: Saaremaa virus AJ616854	12	HtSi_275_p2008; HtSi_1036_a2001	103	37.4
	254: Hantaan Q32 AB027097	76	HtSi_275_p2008; HtSi_1036_a2001	69.8	24.2
	262: Soochong SC1 AY675349	100	HtSi_275_p2008; HtSi_1036_a2001	51.8	24.4
DOBV 86/98 *86/18* (Slovenia)	245: DOBV Belgrade L41916	8	Dobrava-Belgrade virus isolate HtSi_1012_a1997	159	43.1
	246: DOBV Esl/81Aa/01 AY533120	5	Dobrava-Belgrade virus isolate HtSi_1012_a1997	221	61.8
	247: Saaremaa virus AJ616854	9	Dobrava-Belgrade virus isolate HtSi_1012_a1997	99.0	39.6

See legend of Table 3 for details on presented results.

**Table 5:** Detection and genetic characterization of Tula orthohantaviruses (TULV) from various endemic area in Europe: TULV isolates or laboratory strain were hybridized to the hantavirus sequences tiled on the PathogenID v3.0 resequencing microarray (seq222 to seq273; Table 1; Supplement 1).

Tested virus (geographical origin)	Tiled sequence allowing detection (position on the chip)	BLASTN identification		Score (total)	Call rate (%)
		nbr^°^	sequences		
S666_13 Schlindermanderscheid (Germany)	261:ProspectHill M34011	2	S667_13_Marv; S666_13_Marv	64.4	25.9
	264: Kosice144/Ma/95	2	S667_13_Marv; S666_13_Marv nucleocapsid protein gene, partial	46.4	23.2
	265: Moravia5293Ma/94	2	S667_13_Marv; S666_13_Marv nucleocapsid protein gene, partial	107	38.1
TULV09_1000 Cunnersdorf (Germany)	265: Moravia5293Ma/94	9	09_1000_Marv nucleocapsid protein gene, partial cds	42.8	38.4
TULV laboratory strain Moravia	234: Perpignan1999	8	Tula virus S segment nucleocapsid protein mRNA, complete cds	55.4	23.8
	244: CovictCreek107	49	Tula virus segment S, strain Tula/Moravia/5294 Ma/94, genomic RNA	42.8	16.2
	261: Prospect Hill M34011	7	Tula/Moravia/5294 Ma/94, genomic RNA	48.2	30.9
	263: Sennickerode_Sen05/204	72	Tula/Moravia/5294 Ma/94, genomic RNA	42.8	25.4
	264: Kosice144/Ma/95	10	Tula/Moravia/5294 Ma/94, genomic RNA	55.4	21.2
	265: Moravia5293Ma/94	1	Tula virus segment S, strain Tula/Moravia/5302 Ma/94	688	97.5
	266: MG23/Omsk	8	Tula/Moravia/5302 Ma/94, genomic RNA	44.6	21.4
TULV (Slovenia)	265: Moravia5293Ma/94	1	Tula virus isolate HtSi_1087_a1999 nucleocapsid protein gene, partial cds	123	37.4

See legend of Table 3 for details on presented results.

**Table 6:** Detection and genetic characterization of Seoul orthohantaviruses (SEOV) from various endemic area in Europe: SEOV isolates were hybridized to the hantavirus sequences tiled on the PathogenID v3.0 resequencing microarray (seq222 to seq273; Table 1; Supplement 1).

Tested virus (geographical origin)	Tiled sequence allowing detection (position on the chip)	BLASTN identification\		Score (total)	Call rate (%)
		nbr^°^	sequences		
Seoul Lyon 892 (France)	255: Hantavirus SEOV Z37	1	*Seoul virus isolate LYON/Rn/FRA/2013/LYO852 segment S*, *complete*	235	64.0
	257: Hantavirus SEOV L99	28	*Seoul virus isolate LYON/Rn/FRA/2013/LYO852 segment S*, *complete*	127	44.6
	258: SEOV 8039	10	*Seoul virus isolate LYON/Rn/FRA/2013/LYO852 segment S*, *complete*	140	53.6
Seoul Cherwell (UK)	255: Hantavirus SEOV Z37	1	*Seoul virus nucleocapsid protein gene*, *complete cds Cherwell*	161	52.9
	257: Hantavirus SEOV L99	1	*Seoul virus nucleocapsid protein gene*, *complete cds Cherwell*	91.5	48.1
	258: SEOV 8039	8	*Seoul virus nucleocapsid protein gene*, *complete cds Cherwell*	136	51.6

See legend of Table 3 for details on presented results.

**Table 7:** Detection and genetic characterization of Topografov orthohantavirus (TOPV): the laboratory strain Topografov was hybridized to the hantavirus sequences tiled on the PathogenID v3.0 resequencing microarray (seq222 to seq273; Table 1; Supplement 1).

Tested virus (geographical origin)	Tiled sequence allowing detection (position on the chip)	BLASTN identification		Score (total)	Call rate (%)
		nbr^°^	sequences		
Topografov laboratory strain	228: Ernstbrunn/Cg641/1995	1	*Topografov hantavirus S segment gene for N protein*	44.6	15.0
	234: Perpigna1999	12	*Topografov hantavirus S segment gene for N protein*	60.8	30.8
	235: Fusong90006	1	*Topografov hantavirus S segment gene for N protein*	50.0	29.4

See legend of Table 3 for details on presented results.

#### Genetic characterization of Puumala viruses circulating in Europe

**Figure 3:**
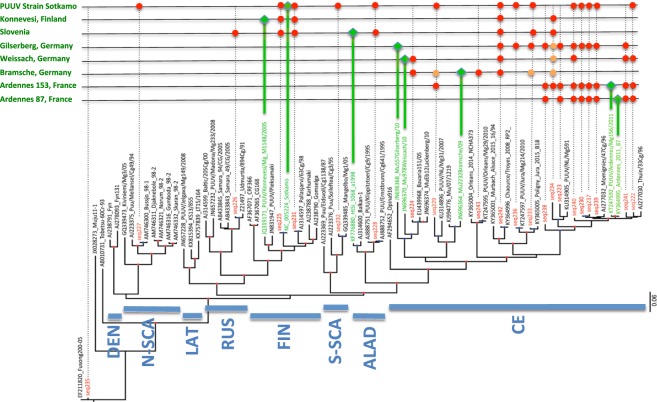
Phylogenetic analysis of European Puumala orthohantavirus (PUUV) genetically characterised by PathogenID v3.0 resequencing microarray. A phylogenetic tree backbone was first constructed from complete S segment coding sequences of reference hantaviruses available in GenBank using the maximum-likelihood method (ML) with PhyML v3.0, implemented in Seaview (v.4.6.1) with the most appropriate substitution model as determined by SMS program. Shorter sequences (tiled on the chip & tested viruses) were then placed in the backbone tree using RaxML. Nodes with branch support values > 0.8 are indicated by a red point (reference tree, aLRT branch support test) or by a blue point (phylogenetic placement of short sequence in the reference tree, rapid bootstrap procedure with MRE-based Bootstopping criterion). Scale bar represents the average number of substitutions per site. Under the tree are indicated the main geographical clusters of PUUV in Europe: CE (Central Europe); ALAD (Alpes-Adrian); S-SCA (South Scandinavia); FIN (Finland); RUS (Russia); LAT (Latvia); N-SCA (North Scandinavia); DEN (Denmark). The 22 PUUV sequences fixed on the chip (seq222 to seq243) are outlined in red, those of the 8 tested viruses in green. In the upper grid, the 8 tested Puumala viruses (the laboratory PUUV Sotkamo strain and 7 European geographical variants) are presented with: their exact position in the tree (green diamond); the tiled sequences having permitted their detection (significant CR) with exact (red circles) or close (orange circles) genetic identification by BLASTN. Complementary information is described in Table 3 and Supplement 5.

RNA extracts from supernatant of Vero E6 cells infected with the PUUV Sotkamo strain, and RNA extracts from lungs of seven PUUV-infected bank voles originating from France, Germany, Finland or Slovenia were individually hybridized to the 22 PUUV sequences (seq222 to seq243) tiled on the PathogenID v3.0 resequencing microarray. It is of note that only two of the tested samples had their exact sequence tiled on the chip: the PUUV Sotkamo strain (seq225) and the French Ardennes PUUV strain 87 (seq237). When a significant signal was detected, the corresponding raw sequence was subjected to BLASTN enquiry for genetic typing (Table 3). Figure 3 pictures the results in the context of a phylogenetic tree illustrating the currently known diversity of PUUV by combining references sequences available in GenBank, sequences tiled on the chip (in red) and sequences of the tested viruses (in green). An unequivocal determination of the correct geographical variant was observed for all the tested samples at least with one tiled sequence (red dots in Fig. 3), even when the corresponding sequence was not tiled itself on the chip. In very few cases (<8%, orange dots in Fig. 3) tiled sequences designated only an approximate origin, however always in the same genetic cluster.

The French Ardennes PUUV lung-derived variants 87 and 153 were precisely identified by BLASTN not only using the very homologous French Ardennes PUUV tiled sequences (seq237-seq241), but also with tiled sequences from Belgium (seq222-seq223, seq230) or even from North-West Germany (seq229 for variant 153) more distant phylogenetically within the Central European (CE) clade. With the same logic, the three PUUV variants from Germany were precisely characterized by 91% (10/11; Gilserberg), 84% (5/6; Weissach) and 50% (3/6; Bramsche) of the hybridizing homologous and heterologous tiled sequences from the Central European clade (Table 3). More interestingly, the Konnevesi variant from Finland was exactly identified not only with tiled sequences from its specific clade (Finland, FIN) but also from the Central European (CE) clade. Equally, the variant 8098 from Slovenia (clade Alpes-Adrian, ALAD) was exactly identified with sequences from the ALAD clade, and also from the CE, FIN and Russia (RUS) clades. Finally, the laboratory PUUV Sotkamo strain was systematically identified by tiled sequences from almost all clades of the PUUV phylogenetic tree.

It is of note that these very precise characterisations could be obtained despite very low CR values, for example down to only 13% of defined nucleotides between the tested PUUV variant Slovenia 8098 and the tiled seq226 (Kazan_Z84204) (Table 3). It is explained by the exact determination of short fragments (stretches) of significant sequence (e.g. minimum of 15 nucleotides) that allow precise BLASTN identification despite poor CR values (Table 3; Supplement 5).

#### From Puumala virus to other orthohantaviruses circulating in Europe

The potential in detection and genetic characterization of the resequencing chip Pathogen ID v3.0 was further evaluated for variants and laboratory strains of other hantavirus species circulating in Europe, namely DOBV, TULV, SEOV and TOPV. For this purpose, 30 additional hantavirus sequences (seq244 to seq273) were tiled on the microarray.

For DOBV, RNA extracts from two cell supernatants infected with reference laboratory strains Belgrade and Saaremaa and RNA extracts from two Yellow-necked mice from Slovenia were tested (Table 4). All of them were exactly characterized: (1) by the DOBV (seq245-seq246) and/or SAAV (seq247) sequences tiled on the chip, as expected from the genetic similarity of both viruses; (2) more interestingly, by tiled sequences from more distant orthohantavirus species such as HTNV and Soochong virus. Significant results were also observed for TULV RNA extracts from one laboratory strain and three Common vole samples from Germany, Finland and Slovenia (Table 5). Following BLASTN, the correct sequence was either exclusively or at least dominantly characterized. Here again, precise detection and identification was possible with tiled sequences from very distant clades of vole-associated New world orthohantaviruses such as Prospect Hill or Sin Nombre orthohantaviruses (Table 5). Finally the SEOV present in RNA extracts from Norway rats from France and UK, although unknown when the chip was designed, was perfectly identified by heterologous SEOV tiled sequences (Table 6). Similarly for RNA extracts from a TOPV laboratory strain, in absence of the corresponding sequence tiled on the chip, the exact characterization was achieved with PUUV tiled sequences (seq228, seq234, seq325) even at low CR of 15% (Table 7).

## Discussion

Hantaviruses are zoonotic agents distributed world-wide. In Europe, rodent-borne hantaviruses are regularly provoking episodes of Hemorrhagic Fever with Renal Syndrome (HFRS). Tools to survey hantavirus circulation, geographical distribution and genetic features in the animal reservoir are essential to a better understanding and prevention of hantavirus infection in humans. Resequencing microarray has been shown powerful to precisely identify new genetic variants of emerging viruses^15–21^.

The present work represents a significant improvement of the resequencing microarray PathogenID developed through a collaborative study for detection and identification of orthohantaviruses circulating in Europe. The 1^st^ and 2^nd^ generations of PathogenID allowed to detect different viruses associated with hemorrhagic fevers, including hantaviruses; however they also showed their limits for the detection of PUUV^20^ which is the most common and widespread European hantavirus causing a mild form of HFRS, Nephropatia Emidemica (NE)^2–8^. Therefore, we have switched the strategy for the design of the 3^rd^ generation PathogenID v3.0 used in this study. Tiled sequences did not target anymore the most conserved region of the genome, the L segment^24^, but the S segment encoding the N protein, which present two advantages: it is more efficient to discriminate variants within a species; more sequences are present in GenBank. After a critical analysis of the taxonomy, 52 representative hantavirus sequences were selected among those available at the time of the conception of the chip (Table 1). The resequencing methodology (Supplement 2) allowed to recognize both known viruses and previously unknown geographical variants.

Validation carried out by using hantavirus N protein encoding plasmids allowed to show a global tolerated divergence (up to 20%) between the tiled and the tested sequences for a correct identification of the PUUV prototype strain Sotkamo (Table 1, Table 2, Fig. 1; Supplement 3). A synergic effect in detection was observed when three viral species (PUUV; TULV; HTNV) were simultaneously tested (Supplement 4), most likely due their cross-contribution in hybridizing orthohantavirus conserved nucleotides, favoring identification by BLASTN analysis.

Identification of European hantaviruses, PUUV, DOBV, TULV, SEOV, TOPV present in tissue samples or in supernatants of Vero E6 cells infected with laboratory strains was demonstrated not only using the homologous sequences tiled on the chip, but also using phylogenetically distant sequences (Tables 3–7; Fig. 3). The key factor for the precise characterization of the tested hantavirus sequence was obviously its genetic distance from the tiled one (Fig. 3) outlining the importance of designing the chip from sequences encompassing the global diversity of hantaviruses. However, even for samples with a low Call Rate (CR: % of determined/total number of nucleotides following hybridization), precise taxonomical identification was possible when short specific fragments of significant sequence (about 15 nucleotides) were obtained for BLASTN analysis (Supplement 5). This was in particular the case for TOPV and PUUV Konnevesi variant in the present study (Tables 3–7). A similar observation was previously reported for hemorrhagic fever viruses detected by PathogenID v.2^20^. These short stretches of highly conserved sequences among hantavirus could serve for developing other hybridization methods such as hybrid captures^43^.

Geographical variants of PUUV, the most frequent hantavirus in Europe, was correctly determined in the different endemic areas, such as France, Germany, Finland, and Slovenia (Table 3, Fig. 3). SEOV isolate, recently pointed out to circulate both in France and in UK^39–41^, was also precisely characterized despite neither Lyon nor Cherwell isolates, respectively, were known when the microarray was designed (Table 6). Interestingly, both isolates were detected and correctly identified from two heterologous sequences tiled on the microarray, SEOV and HTNV (Table 6). In any cases, when a tiled sequence did not achieve the determination of the exact geographical variant, it was at least designing the phylogenetic clade and the number of sequences tiled on the microarray always allowed to reach the deepest level of precision (Fig. 3).

Altogether, the results obtained with DOBV, TULV, SEOV and TOPV clearly outline the potential of Pathogen ID v3.0 to largely explore the hantavirus genetic space and to deliver precise identification of the species and local variants present in the infected tissue or in the cell supernatant. The suitability of this approach was demonstrated to map the wide diversity of hantaviruses within the European continent, including new variants unknown at the moment of the design of the chip. Detection by resequencing microarray which is applicable to both animal and human samples, is of interest for both research and public health aspects. Our results are promising to enlarge evaluation to other hantaviruses from different continents, both the pathogenic ones circulating in other endemic areas, such as Americas where they provoke severe HCPS and also in other animal reservoirs such as insectivores and bats.

## Supplementary information

Supplementary data
