# Supplementary material for: Revisiting the genetic diversity of emerging hantaviruses circulating in Europe using a pan-viral resequencing microarray
Source: Sci Rep. 2019 Aug 27;9:12404. doi: 10.1038/s41598-019-47508-7 (PMC6712034; doi:10.1038/s41598-019-47508-7)
Supplement: Supplementary file 1 — Supplementary data [file 41598_2019_47508_MOESM1_ESM.pdf]

SUPPLEMENTARY INFORMATION

Title

Revisiting the genetic diversity of emerging hantaviruses circulating in Europe using a pan-viral resequencing microarray

Authors

Claudia Filippone, Guillaume Castel, Séverine Murri, Myriam Ermonval, Misa Korva, Tatjana Avšič-Županc, Tarja Sironen, Olli Vapalahati, Lorraine Marie McElhinney, Rainer G. Ulrich, Martin H. Groschup, Valérie Caro, Frank Sauvage, Sylvie van der Werf, Jean-Caude Manuguerra, Antoine Gessain, Philippe Marianneau, Noël Tordo

SUPPLEMENT 1

List of hantavirus sequences tiled on the 3rd generation resequencing microarray (PathogenID v3.0).

The number of the sequence is referred to the position on the pan-viral chip (total number: 838 sequences). Sequences are named as in the NCBI database (Description and Accession number) or according to laboratory nomenclature, for the sequences experimentally obtained at the laboratory. The size of hantavirus sequences tiled on the microarray is in most cases 425 nucleotides (nt), except for the sequence >seq234 (251 nt), and sequences from position >seq238 to >seq243 (303 nt). Complementary information is found in Table 1.

>seq222 Puumalavirus_RNA_for_nucleocapsid_protein_CG14444_AJ277075
ACTGGTATACAATTGGAGTGTATGTTATAGGCTTCACACTTCCTATTATCCTGAAGGCTCTATATAT
GTTGTCAACACGTGGGAGACAAACTGTGAAAGAAAACAAAGGGACTCGTATCCGTTTTAAAGATG
ACACTTCCTTCGAGGACATTAATGGTATCAGGAGACCGAAGCACCTATATGTTTCCATGCCTACTG
CTCAATCAACTATGAAGGCTGAAGAACTGACCCCTGGGCGATTTAGAACCATAGTATGCGGTCTAT
TTCCGACTCAAATTCAAGTACGCAACATAATGAGCCCTGTAATGGGAGTCATTGGTTTTTCCTTTTT
TGTCAAGGACTGGGCAGATCGGATTAGGGAATTCATGGAAAAAGAGTGCCCTTTTATCAAACCAG
AGGTGAAGCCAGGAACACCAGCCCAGGAA
>seq223 Puumalavirus_CG13891_RNA_nucleocapsid_protein_gene_U22423
ACTGGTATACAATCGGAGTGTATGTCATAGGCTTTACACTTCCTATTATCCTGAAGGCCTTGTATAT
GTTGTCAACACGTGGGAGACAAACTGTGAAAGAAAACAAAGGAACTCGCATTCGTTTCAAAGATG
ACACTTCTTTTGAGGACATTAACGGTATCAGGAGACCAAAGCACCTATATGTTTCCATGCCTACTG
CTCAATCAACCATGAAGGCTGAAGAACTGACCCCTGGGCGATTTAGAACCATAGTATGTGGTCTAT
TCCCAACTCAAATTCAAGTGCGTAACATAATGAGTCCTGTAATGGGAGTAATTGGCTTCTCTTTTTT
CGTTAAAGACTGGGCAGATCGGATTAGAGAATCTATGGAAAAAGAGTGCCCCTTTCTAAAACCAG
ATGTAAAGCCAGGGACACCAGCCCAGGAA
>seq224 Puumalavirus_Bavaria_CG9/04_AY954722_nucleocapsid_protein
ATTGGTATACAATTGGGGTTTATGTCATAGGTTTCACACTCCCTATCATCTTGAAGGCCTTGTATAT
GTTATCAACCCGTGGGAGACAGACTGTGAAGGAGAACAAAGGGACTCGCATCCGGTTTAAAGATG
ATACTTCATTTGAGGACATTAATGGTATTAGGAGACCAAAGCATTTGTATGTGTCCATGCCCACTG
CTCAGTCAACTATGAAAGCTGAAGAATTAACCCCTGGTAGGTTCAGGACTATAGTCTGCGGTTTAT
TTCCAACTCAGATTCAAGTACGTAATATAATGAGTCCTGTGATGGGAGTCATCGGTTTTTCTTTCTT
TGTAAAGGACTGGGCAGAACGTATTAGGGAATTTATGGAGAAAGAATGCCCCTTCATCAAACCAG
AAATGAAACCAGGAATACCAGCACAGGAG
>seq225 Puumalavirus_Sotkamo_NC_005224
ATTGGTATACAATTGGAGTGTATGTGATAGGGTTCACACTTCCTATCATCCTTAAAGCTTTATACAT
GCTCTCAACGCGTGGGAGACAGACTGTAAAGGAAAATAAGGGGACACGTATAAGGTTTAAGGATG
ATACATCATTTGAAGACATCAATGGCATAAGGAGACCAAAGCATTTATATGTTTCTATGCCTACTG
CCCAGTCAACTATGAAAGCAGAAGAACTCACACCTGGCAGATTTCGCACAATAGTATGTGGTCTTT
TTCCCACTCAGATCCAGGTTCGTAACATCATGAGTCCAGTTATGGGGGTCATTGGTTTTTCATTCTT
TGTGAAGGATTGGTCTGAGAGAATCAGAGAGTTCATGGAAAAAGAGTGCCCATTCATAAAGCCTG
AAGTAAAACCAGGCACACCAGCACAGGAG
>seq226 Puumalavirus_Puu/Kazan_Z84204
ATTGGTATACTATTGGAGTCTATGTAATAGGTTTTACAATTCCCATCATTTTAAAGGCTCTATACAT
GTTGTCAACACGTGGAAGACAGACCGTAAAAGAAAATAAAGGAACACGGATCAGGTTCAAAGATG
ACACATCGTTTGAGGATATTAATGGCATCAGGAGACCAAAACATCTATATGTATCCATGCCTACTG
CCCAGTCCACCATGAAAGCTGAAGAACTTACACCCGGACGGTTCCGTACAATAGTATGTGGCTTAT
TTCCTACACAGATACAAGTGCGTAACATCATGAGTCCAGTAATGGGAGTGATTGGTTTTTCTTTCTT
TGTCAAAGACTGGCCAGAGAGGATTAGGGATTTTATGGAAAAGGAATCCCCCTTCATAAAGCCAG
AAGTTAAACCCGGGACACCAGCACAGGAG
>seq227 Puumalavirus_Umea/hu_AY526219
ATTGGTATACAATTGGAGTGTATGTAATAGGGTTTACACTACCTATTATTTTGAAGGCCTTATACAT
GCTTTCCACACGTGGGAGACAAACTGTTAAGGAGAATAAAGGGACACGTATCCGCTTTAAGGATG

ACACTTCATTTGAAGATATCAATGGCATTAGGCGACCAAAGCACCTCTATGTATCTATGCCTACAG
CCCAGTCTACTATGAAAGCAGAGGAACTTACACCAGGTCGGTTTCGCACAATAGTTTGTGGCTTAT
TTCCTACACAAATTCAAGTTCGAAATATAATGAGTCCAGTAATGGGGGTGATTGGCTTTTCCTTCTT
TGTTAAGGACTGGCCAGACAGGATAAGAGAGTTTATGGAAAAGGAATGCCCATTTATAAAGCCAG
AAGTTAAACCAGGGACACCAACACAGGAA
>seq228 Puumalavirus_PUU/Ernstbrunn/Cg641/1995_AJ888752
ACTGGTATACAATTGGAGTTTATGTCATTGGCTTTACACTCCCAATCATACTAAAAGCCCTCTACAT
GTTATCAACACGAGGAAGACAAACTGTAAAAGAGAATAAAGGAACACGTATACGCTTTAAGGATG
ATACATCATTTGAAGACATCAATGGCATTAAAAGACCGAAGCATTTATATGTATCCATGCCAACAG
CCCAGTCAACAATGAAGGCAGAAAAGCTTACTCCAGGTAGGTTTCGGACAATTGTGTGTGGTCTTT
TCCCAACTCAGATTCAGGTGCGCAACATTATGAGTCCTGTAATGGGGGTTATAGGATTCTCTTTTTT
TGTAAAGGATTGGTCAGAGAGGGTAAGAGATTTTATGGAGAAAGAATGCCCTTTTATAAAACCAG
AAATCAAACCAGGAACACCCCCTCAGGAG
>seq229 Puumalavirus_Berkel_L36943
ATTGGTACACAATTGGAGTCTATGTCATTGGCTTTACACTCCCTATTATCCTAAAGGCCTTATATAT
GTTGTCAACACGTGGAAGACAAACTGTTAAGGAGAATAAAGGAACCCGTATTCGGTTTAAGGATG
ATACTTCATTTGAGGACATCAACGGCATCAGAAGACCAAAACATTTGTATGTATCCATGCCAACTG
CTCAATCAACTATGAAGGCTGAAGAGCTAACTCCTGGTCGCTTTAGGACTATAGTGTGTGGCCTGT
TCCCAACTCAGATCCAAGTACGAAATATAATGAGTCCTGTAATGGGGGTTATAGGCTTTTCCTTTTT
TGTCAAGGATTGGGCAGATCGGATCAGAGAATTCATGGAAAAAGAGTGCCCTTTTATCAAGCCAG
AAGTCAAGCCAGGGACACCAGCACAAGAG
>seq230 Puumalavirus_Couvin/59Cg/97_AJ277034
ACTGGTATACAATTGGAGTGTATGTTATAGGCTTCACACTTCCTATTATCCTGAAGGCTTTATATAT
GTTGTCAACACGTGGGAGACAGACTGTGAAAGAAAACAAAGGAACTCGTATCCGTTTCAAAGATG
ACACTTCCTTCGAGGACATCAATGGTATCAGGAGACCAAAGCACCTATATGTTTCCATGCCTACTG
CTCAATCAACTATGAAGGCTGAAGAATTAACTCCTGGGCGATTTAGAACCATAGTATGTGGTCTAT
TTCCAACTCAAATTCAAGTACGCAACATAATGAGCCCTGTAATGGGAGTCATTGGTTTTTCTTTTTT
TGTCAAGGACTGGGCAGATCGGATTAGGGAGTTCATGGAAAAAGAGTGCCCTTTTATCAAACCAG
AGGTGAAGCCAGGAACACCAGCCCAGGAA
>seq231 Puumalavirus_Pallasjarvi/63Cg/98_AJ314598
ACTGGTATACGATTGGAGTTTATGTAATAGGATTCACACTTCCCATTATCCTTAAAGCCTTATACAT
GCTTTCAACACGTGGAAGACAGACTGTAAAGGAGAATAAGGGGACACGCATAAGGTTTAAGGATG
ATACATCATTTGAAGACATCAATGGCATAAGGAGACCAAAGCATTTATATGTTTCCATGCCTACTG
CCCAGTCAACCATGAAGGCAGAAGAACTCACACCAGGTAGATTTCGTACAATAGTATGTGGTCTAT
TTCCAACTCAGATTCAAGTCCGTAACATCATGAGTCCAGTAATGGGGGTTATTGGGTTTTCATTTTT
TGTAAAGGATTGGTCAGAGAGAATCAGAGAGTTCATGGAAAAAGAGTGCCCATTCATAAAGCCGG
AGGTTAAGCCAGGTACCCCAGCACAAGAG
>seq232 Puumalavirus_Munga/Mg16/05_GQ339487
ATTGGTATACTATTGGAGTTTATGTGATAGGCTTTACACTCCCTATTATTTTAAAGGCACTCTACAT
GTTGTCAACACGTGGAAGACAAACTGTAAAGGAAAACAAAGGGACAAGGATCAGATTTAAGGATG
ACACCTCATTTGAAGACATCAATGGCATCAGGAGACCAAAGCACTTATATGTGTCTATGCCCACAG
CACAGTCAACAATGAAAGCAGAAGAGTTAACACCAGGTCGATTCCGGACAATAGTATGTGGCCTA
TTCCCTACCCAGATTCAAGTACGTAATATAATGAGTCCAGTTATGGGGGTCATTGGCTTTTCTTTCT
TTGTCAAGGATTGGGCAGAAAGGATTAGGGAATTTATGGAGAAGGAATGCCCTTTCATAAAACCA
GAAGTAAAACCTGGAACACCAGCGCAGGAA
>seq233 Puumalavirus_PUU/Mignovillard/CgY02/2005_AM695638
ATTGGTATACAATTGGGGTATATGTCATAGGCTTTACACTCCCTATTATCTTAAAGGCCTTATACAT
GTTATCAACACGTGGGAGACAGACTGTAAAGGAAAATAAGGGAACCCGCATTCGATTTAAGGATG
ACACTTCATTTGAGGACATAAATGGCATTAGAAGACCAAAGCACTTGTATGTATCCATGCCTACAG
CACAATCCACCATGAAAGCAGAAGAGTTAACTCCTGGACGATTTAGGACCATAGTATGTGGCCTAT
TTCCAACTCAAATTCAAGTGCGAAACATAATGAGTCCTGTGATGGGAGTCATCGGGTTTTCATTTTT
TGTTAAGGATTGGACAGATCGGATTAGGGAATTTATGGAAAAGGAATGCCCCTTTATAAAACCAGA
GATGAAACCGGGAACACCAGCCCAAGAA
>seq234Puumalavirus_strainFrance/Perpignan1999_nucleocapsid_protein_gene_partial
ATGAGTGACTTGACAGACATTCAGGAAGATATAACCCGCCATGAGCAACAACTTGTTGTTGCCAGA
CAAAAGCTCAAGGATGCGGAAAAGGCAGTGGAGATGGACCCAGATGACGTTAACAAAAACACATT
GCAAGCAAGGCAACAGACAGTGTCAGCATTGGAGGACAAACTTGCAGACTTCAAGCGACAGATGG
CAGATGCTGTCTCAAGGAAAAAGATGGATACTAGGCCTACTGACCCGACTGGGAT
>seq235 Puumala_Fusong90006_EF488806

ACTGGTATACTATTGGGGTATACATAATTGGATTCACACTGCCTATAATACTCAAAGCCCTGTACAT
GCTGTCAACGAGGGGAAGGCAGACTGTCAAAGAAAATAAAGGGACACGGATCCGTTTCAAGGATG
ACACTTCTTTTGAGGATGTGAATGGGATCAGACGACCAAAACATCTCTATGTGTCAATGCCAACCG
CACAGTCTACCATGAAGGCAGAAGAACTGACACCAGGGAGATTCAGAACAATTGTTTGCGGCCTA
TTTCCTACACAGATTCAAGTGAGAAATATCATGAGCCCCGTAATGGGAGTGATCGGATTCTCCTTC
TTTGCAAAGGACTGGCCAGATAGGATCAAAGAATTTATGGAGAAAGAATGTCCCTTTATAAAACCT
GAAGTCAAGCCTGGAACACCAGCAAAGGAA
>seq236 Puumala_CH-214_Franche_Comt»_
ATTGGTATACAATTGGGGTATATGTTATAGGCTTTACACTCCCTATCATCTTGAAGGCCTTATACAT
GTTATCAACACGTGGGAGACAGACTGTAAAGGAAAATAAGGGAACCCGCATTCGATTTAAGGATG
ACACTTCATTTGAGGACATAAACGGCATTAGAAGACCAAAGCACTTGTATGTATCCATGCCTACAG
CACAATCTACCATGAAAGCAGAGGAGTTAACTCCTGGACGATTTAGGACCATAGTATGTGGTCTTT
TTCCAACTCAAATTCAAGTGCGAAATATAATGAGTCCTGTGATGGGAGTCATCGGGTTTTCATTTTT
TGTTAAGGATTGGGCAGACCGGATTAGGGAATTTATGGAGAAGGAGTGCCCCTTTATTAAACCAGA
GATAAAACCGGGAACACCAGCTCAGGAA
>seq237 Puumala_AR-21_Charleville_MeziÀres
CTGGTATACAATTGGAGTGTATGTTATAGGCTTCACACTTCCTATTATCCTGAAGGCTTTATATATG
TTGTCAACACGTGGGAGACAGACTGTGAAAGAAAACAAAGGAACTCGTATCCGCTTCAAAGATGA
CACTTCCTTCGAGGACATCAATGGTATTAGGAGACCAAAGCACCTATATGTTTCCATGCCTACTGCT
CAATCAACTATGAAGGCTGAAGAATTAACTCCTGGGCGATTTAGAACCATAGTATGTGGTCTATTT
CCAACTCAAATTCAAGTACGCAACATAATGAGCCCTGTAATGGGAGTCATTGGTTTTCTTTTTTTG
TCAAGGACTGGGCAGATCGGATTAGGGAGTTCATGGAAAAAGAGTGCCCTTTTATCAAACCAGAG
GTGAAGCCAGGAACACCAGCCCAGGAGG
>seq238 Puumala_167-2Charleville-Mezieres
GACTGGTATACAATTGGAGTATATGTTATAGGCTTTACACTTCCTATTATCCTGAAGGCTTTGTATA
TGTTGTCAACACGTGGGAGACAAACTGTAAAAGAAAACAAAGGAACTCGTATCCGTTTCAAAGAT
GATACTTCTTTTGAGGACATCAATGGTATTAGAAGACCGAAGCATCTGTATGTCTCCATGCCTACTG
CTCAATCAACTATGAAGGCTGAAGAATTGACCCCTGGGCGATTTAGGACCATAGTATGTGGTCTAT
TTCCAACTCAAATTCAAGTACGCAACATAATGAGCCCT
>seq239 Puumala_167-4Charleville-Mezieres
GACTGGTATACAATTGGAGTGTATGTTATAGGCTTCACACTTCCTATTATCCTGAAGGCTTTATATA
TGTTGTCAACACGTGGGAGACAGACTGTGAAAGAAAACAAAGGAACTCGTATCCGTTTCAAAGAT
GACACTTCCTTCGAGGACATCAATGGTATCAGGAGACCAAAACACCTATATGTTTCCATGCCTACT
GCTCAATCAACTATGAAGGCTGAAGAATTAACCCCTGGGCGATTTAGAACCATAGTATGTGGTCTA
TTTCCAACTCAAATTCAAGTACGCAACATAATGAGCCCT
>seq240 Puumala_178-2Charleville-Mezieres
GACTGGTATACAATTGGAGTGTATGTTATAGGCTTCACACTTCCTATTATCCTGAAGGCCTTGTATA
TGTTGTCAACACGTGGGAGACAAACTGTGAAAGAAAACAAAGGGACTCGTATCCGTTTTAAAGAT
GACACTTCCTTTGAGGACATCAATGGTATCAGGAGACCAAAGCACCTATATGTTTCCATGCCTACT
GCTCAATCAACTATGAAGGCTGAAGAATTAACCCCTGGGCGATTTAGAACCATAGTATGTGGTCTA
TTTCCAACTCAGATTCAAGTACGCAACATAATGAGCCCT
>seq241 Puumala_180-78Charleville-Mezieres
GACTGGTATACAATTGGAGTGTATGTTATAGGCTTTACACTGCCTATTATCCTGAAGGCTTTATATA
TGTTGTCAACACGTGGGAGACAAACTGTGAAAGAAAACAAAGGGACTCGTATCCGTTTCAAAGAT
GACACTTCCTTCGAGGACATCAATGGTATCAGGAGACCGAAGCACCTTTATGTTTCCATGCCTACT
GCTCAATCAACCATGAAGGCTGAAGAATTGACCCCTGGGCGATTTAGAACCATAGTATGTGGTCTA
TTTCCAACTCAAATTCAAGTACGCAATATAATGAGCCCT
>seq242 Puumala_RU-11Ruminy-Troyes
GACTGGTACACAATTGGGGTATATGTTATAGGCTTCACACTCCCTATTATCCTAAAGGCCTTATACA
TGTTATCAACACGTGGGAGACAAACTGTAAAGGAAAATAAAGGGACACGTATTCGGTTTAAAGAT
GACACTTCATTTGAGGACATAAATGGTATTCGAAGACCAAAGCATTTGTATATATCCATGCCCACA
GCACAGTCTACTATGAAAGCAGAGGAGTTAACTCCTGGACGATTTAGAACCATAGTATGTGGTCTC
TTCCCAACTCAAATTCAAGTGCGGAATATAATGAGTCCT
>seq243 Puumala_OR-52_Orleans
GATTGGTATACAATTGGAGTCTATGTTATAGGCTTTACACTCCCTATTATCCTAAAGGCCCTGTACA
TGCTTTCAACACGAGGGAGGCAAACTGTGAAAGAAAACAAGGGAACTCGTATTCGATTTAAAGAT
GATACATCTTTTGAAGATATCAATGGTATTCGCAGGCCAAAGCACCTATATGTGTCCATGCCTACT
GCCCAATCAACTATGAAGGCTGAAGAGCTGACTCCTGGGCGGTTTAGGACCATAGTGTGTGGTCTT
TTTCCAACCCAGATCCAAGTGCGTAATATAATGAGTCCT
>seq244 Pulmonary_syndrome_hantavirus_ConvictCreek107_L33683

ATTGGAAATCCATCGGGCTTTACATCTTGAGTTTTGCCCTACCGATAATTCTTAAGGCCTTATATAT
GTTATCCACCAGGGGCCGTCAAACAATTAAAGAGAACAAAGGGACGAGAATTCGATTTAAGGATG
ATTCATCTTATGAAGAGGTCAATGGGATACGCAAACCAAGACATCTGTATGTTTCCATGCCTACTG
CTCAATCTACAATGAAAGCAGATGAAATCACTCCTGGGAGGTTCCGTACAATTGCTTGTGGCTTGT
TTCCTGCTCAGGTCAAGGCTAGAAATATCATCAGTCCTGTCATGGGTGTGATTGGATTTAGTTTTT
TGTTAAAGATTGGATGGAGAGGATTGATGAATTTCTGGCTGCTCGTTGTCCATTCTTGCCTGAACAA
AAAGACCCTAGGGATGCCGCATTAGCA
>seq245 DobravaBelgrade_L41916
ACTGGCTAAGCATTGTGATCTACCTGACATCATTCGTGGTCCCAATACTGTTGAAGGCTCTTTACAT
GCTTACCACCAGAGGGAGACAAACTACTAAAGACAATAAGGGAATGAGGATTCGATTTAAGGATG
ACAGCTCTTTTGAAGATGTGAATGGGATTCGAAAGCCAAAGCACCTGTTCTTGTCAATGCCCAATG
CACAATCTAGTATGAAGGCAGATGAGATTACACCAGGTCGGTTCAGGACTGCAATCTGTGGACTAT
ACCCAGCCCAGGTTAAGGCAAGGAATTTAATCAGTCCTGTGATGAGTGTGATTGGGTTTGTAGCCC
TTGCAAAAAACTGGACAGAACGGGTTGAAGAATGGCTTGACCTCCCGTGCAAGCTACTATCTGAGC
CATCTCCAACGTCTTTGACCAAAGGCCCA
>seq246 Dobrava_Esl/81Aa/01_AY533120
ATTGGTTAAGTATTGTGGTCTATTTAACATCTTTTGTGGTCCCGATACTCTTAAAGGCTCTTTATATG
TTAACAACTAGAGGGAGACAGACTACTAAGGACAACAAGGGAATGAGGATTCGCTTCAAGGATGA
CAGCTCGTTTGAGGATGTGAACGGGATCCGGAAACCAAAACATTTGTTCCTGTCAATGCCCAATGC
TCAGTCCAGTATGAAGGCAGATGAAATTACACCTGGCCGATTCAGAACTGCAATTTGTGGATTATA
TCCAGCTCAGGTTAAAGCAAGAAATTTGGTTAGTCCTGTCATGAGTGTGATTGGTTTTTGGCCCTT
GCAAAGAACTGGACAGAGAGGGTTGAAGAGTGGTTAGACCTTCCATGTAAGCTGCTATCTGAGCC
ATCTCCAACATCTTTGACCAAAGGCCCA
>seq247 Saarema_virus_AJ616854
ACTGGTTAAGCATTGTTGTCTATTTGACATCTTTTGTGGTCCCAATACTCCTGAAGGCTCTTTATATG
TTGACAACTAGAGGGAGACAAACTACTAAGGACAACAAAGGGATGAGGATTCGGTTCAAGGATGA
TAGCTCATTTGAAGATGTGAATGGGATCCGGAAGCCAAAACATCTATTTTTGTCAATGCCTAATGC
CCAGTCTAGCATGAAAGCAGATGAGATTACACCCGGTCGGTTCAGGACTGCAGTCTGTGGATTATA
CCCGGCACAGGTTAAAGCAAGAAATCTGGTCAGCCCCGTTATGAGTGTGATTGGTTTTTAGCCCT
TGCAAAGAACTGGACAGAACGGGTTGAGGAGTGGCTTGATCTTCCATGCAAGCTATTATCTGAGCC
ATCCCCAACATCTTTGACCAAAGGTCCA
>seq248 Kenkemevirus_MSB148794_GQ306148
ACTGGATAAATATAATAACATACATTTTGACATTCGTAGATACTATATTGCTTAAGGGACTCTACAT
GCTAACAACAAGGGGGAGGCAGACAGTAAAGGATAATAAAGGAACCAGGATCAGACTAAAGGAT
GATACATCATTTAGTGACACAGCAGCTGGTCGTAAACCCAAGATGTTATATATTTCCATGCCGAAT
GCACAATCTAGCATGAGAGCTGATGAAATTACACCTGGTCGTTACCGAACTGTAGTCTGTGGGCTT
TACCCTGCCCAAATTAGGCAGAGGCAGATGATAAGTCCCGTGATGGGCGTCATCGGATTTCCTGCA
ATTGCAAAAAACTGGACAGATCGTGTTGAGCAGTTCTTAGAAAGTGATTGCCCTTTCCTAAAACAG
ACTTTACACCTTACAATTGGGGCCCTTGAT
>seq249 Hantaan_M14626_reference
ACTGGCTGAGCATCATCGTCTATCTTACATCCTTTGTCGTCCCGATACTTCTGAAAGCTCTGTATAT
GTTGACAACAAGGGGGAGGCAAACTACCAAGGATAATAAAGGGACCCGGATTCGATTTAAGGATG
ATAGCTCGTTCGAGGATGTTAACGGTATCCGGAAACCAAAACATCTTTACGTGTCCTTGCCAAATG
CACAGTCAAGCATGAAGGCAGAAGAGATTACACCTGGTAGATATAGAACAGCAGTCTGTGGGCTC
TACCCTGCACAGATTAAGGCACGGCAGATGATCAGTCCAGTTATGAGTGTAATTGGTTTTCTAGCA
TTAGCAAAGGACTGGAGTGATCGTATCGAACAATGGTTAATTGAACCTTGCAAGCTTCTTCCAGAT
ACAGCAGCAGTTAGCCTCCTTGGTGGTCCT
>seq250 Hantaan_KI_GU140098
ATTGGCTCAGTATTGTTATATATTTAACATCCTTTGTTGTCCCAATACTTCTAAAGGCCCTATATATG
CTTACAACAAGAGGGAGGCAGACAACCAAAGATAATAAAGGGACTAGGATTCGATTCAAGGATGA
CAGCTCCTTTGAGGATGTTAACGGGATTCGGAAACCAAAACATTTATATGTTTCCTTGCCCAATGCC
CAGTCAAGCATGAAGGCAGAAGAGATCACACCAGGGAGATACAGGACAGCAATCTGTGGACTCTA
CCCTGCACAGATTAAGGCTAGACAGATGATCAGCCCTGTCATGAGTGTTATTGGATTTTTGGCTCTG
GCAAAAGATTGGAGTGACCGTATTGAGCAGTGGCTAAGTGAACCTTGTAAGCTACTCCCAGATACA
GCAGCAGTAAGTCTCCACGGGGGCCCT
>seq251 Hantaan_Z10_EF533944
ACTGGTTAAGTATTGTTATCTATTTGACATCCTTTGTTGTACCGATCCTTCTGAAAGCCCTGTATATG
CTAACAACGAGGGGGAGACAAACAACCAAAGACAACAAAGGGACCAGAATTCGGTTCAAGGATG
ACAGTTCTTTTGAGGATGTCAATGGGATCCGGAAACCAAAACATCTGTATGTATCCTTGCCCAATG
CTCAATCAAGCATGAAGGCAGAAGAGATTACACCTGGTAGGTACAGGACAGCAATCTGTGGACTC

TATCCTGCACAGATCAAGGCCAGACAGATGATTAGCCCAGTCATGAGTGTAATTGGTTTCCTGGCA
CTGGCTAAAGACTGGAGTGACCGAATTGAGCAATGGCTTAGTGAACCTTGTAAACTACTTCCAGAT
ACAGCAGCAGTAAGCCTCCATGGAGGCCCT
>seq252 Hantaan_AA2499_AF427320
ACTGGCTGAGCATTGTTGTCTATCTTACATCCTTTGTTGTCCCAATACTTCTGAAAGCTCTGTATATG
TTAACAACAAGGGGGAGGCAGACCACCAAGGACAATAAGGGAACTCGGATTCGATTCAAGGATGA
TAGTTCCTTCGAGGATGTCAATGGCATCCGGAAGCCAAAACATCTATATGTGTCCTTGCCAAATGC
ACAGTCAAGTATGAAAGCAGAAGAGATTACACCTGGTAGATATAGAACAGCAATTTGTGGGCTTT
ATCCTGCACAAATTAAGGCAAGACAGATGATTAGTCCTGTCATGAGTGTGATCGGATTCCTGGCTC
TGGCAAAAGATTGGAGTGACCGCATTGAGCAGTGGTTAAGTGAACCCTGCAAGCTTCTTCCAGACA
CAGCAGCAGTTAGCCTCCTTGGTGGTCCT
>seq253 Hantaan_CGHu1_EU092218
ACTGGTTGAGTATTGTTGTCTATCTCACATCACTTGTTGTCCCGATACTTCTAAAAGCTCTGTATATG
TTAACAACAAAGGGGAGGCAGACCACCAAGGACAATAAAGGGACTCGGATTCGATTCAAAGATGA
TAGCTCCTTCGAGGATGTCAATGGCATTCGGAAGCCTAAACATCTATATGTGTCTTTGCTTAATGCA
CAGTCAAGTATGAAGGCAGAAGAGATTACACCTGGTAGATATAGGACAGCAATCTGTGGACCTTA
TCCTGCACAAATTAAGGCAAGACAGATGATTAGTCCTGTCATGAGTGTGATTGGATTCCTGGCCTT
GGCAAAAGATTGGAGTGACCGCATTGAGCAGTGGTTAAGTGAACCCTGTAAGCTTCTTCCAGACAC
AGCGGCAGTTAGCCTCCTTGGTGGTCCT
>seq254 Hantaan_Q32_AB027097
ACTGGCTTAGTATTGTTATATATCTGACATCTTTTGTTGTCCCGATACTTCTGAAGGCCTTATATATG
CTCACAACAAGAGGAAGGCAGACAACCAAAGATAACAAAGGGACCAGGATTCGATTCAAGGATG
ACAGCTCTTTTGAGGATGTTAACGGGATTCGAAAGCCAAAGCATCTATATGTGTCCTTGCCCAATG
CCCAATCAAGCATGAAGGCAGAAGAGATCACACCAGGTAGGTACAGAACAGCAATCTGTGGACTT
TATCCTGCACAGATCAAAGCCAGACAAATGATTAGCCCTGTCATGAGTGTAATTGGATTTTTGGCG
CTGGCAAAAGATTGGAGTGACCGTATTGAACAGTGGTTAAGTGAACCTTGTAAGCTACTCCCGGAC
ACAGCAGCAGTAAGCCTTCATGGAGGCCCT
>seq255 Hantavirus_Z37_AF187082
ATTGGTTGACCATAATTGTCTATCTAACATCATTTGTGGTCCCGATCATCTTGAAGGCACTGTACAT
GTTAACAACAAGAGGTAGGCAGACTTCAAAGGACAACAAGGGGATGAGGATTAGATTCAAGGATG
ACAGCTCATATGAGGATGTCAATGGAATCAGAAAGCCCAAACATCTGTATGTGTCAATGCCAAATG
CCCAATCCAGCATGAAAGCTGAAGAGATAACACCTGGAAGATTCCGCACAGCAGTATGCGGGCTA
TATCCTGCACAGATAAAGGCAAGGAACATGGTAAGCCCTGTCATGAGTGTAGTTGGGTTTTTGGCA
CTGGCAAAAGACTGGACATCTAGAATTGAAGAATGGCTTGGCGCACCTTGCAAATTCATGGCGGA
GTCTCCCATTGCCGGGAGTTTATCTGGGAAT
>seq256 Hantavirus_Nc167_AB027523
ACTGGTTGAGCATCATTGTATATTTGACCTCATTTGTAGTCCCAATCTTGTTGAAAGCACTATACAT
GTTAACAACAAGAGGAAGGCAAACAGCTAAGGACAACAAGGGAACACGGATCAGATTTAAGGAT
GACAGTTCTTTTGAGGATGTCAATGGAATTCGGAAACCTAAACATCTTTATGTGTCAATGCCTAATG
CCCAATCAAGCATGAAGGCAGAAGAGATTACACCAGGAGGCTACCGAACAGCTGTATGTGGGCTT
TTTCCTGCACAGATCAAAGCAAGACAGATGGTTAGCCCTGTTATGAGTGTGATTGGCTTCATGGCA
TTGGCAAAGGACTGGACTGATCGGATTGAGAGCTGGCTTGGTGAACCCTGCAAACTTTTACCAGAC
ACATCAGCTGTGAGTCTCCTGGGAGGACCG
>seq257 Hantavirus_L99_AF488708
ATTGGTTGACCATAATTGTCTATCTGACTTCATTTGTGGTCCCGATCATCTTAAAGGCACTGTACAT
GTTAACAACAAGAGGCAGGCAGACTTCAAAGGACAACAAAGGGATGAGGATCAGATTCAAGGAT
GACAGCTCATATGAGGATGTCAATGGAATCAGAAAACCCAAGCATCTGTATGTGTCAATGCCAAA
CGCCCAATCCAGCATGAAGGCTGAAGAGATAACACCTGGAAGATTCCGCACTGCAGTATGTGGGC
TATATCCTGCACAGATAAAGGCAAGGAACATGGTGAGCCCTGTCATGAGTGTAGTTGGGTTTTTGG
CACTGGCAAAAGATTGGACATCTAGAATTGAAGAATGGCTTGGTGCACCCTGCAAGTTTATGGCGG
AATCTCCAATTGCTGGGAGTTTATCTGGGAAT
>seq258 Seoul_8039_NC005236
ATTGGCTGACTATAATTGTCTATCTAACATCATTCGTGGTCCCGATCATCTTGAAGGCACTGTACAT
GTTAACAACAAGAGGTAGGCAGACTTCAAAGGACAACAAGGGGATGAGGATCAGATTCAAGGAT
GACAGCTCATATGAGGATGTCAATGGGATCAGAAAGCCTAAACATCTGTATGTGTCAATGCCAAAC
GCCCAATCCAGTATGAAGGCTGAAGAGATAACACCAGGAAGATTCCGCACTGCAGTATGTGGGCT
ATATCCTGCACAGATAAAGGCAAGGAATATGGTAAGCCCTGTCATGAGTGTAGTTGGGTTTTTGGC
ACTAGCAAAAGACTGGACATCTAGAATTGAAGAATGGCTTGGCGCACCCTGCAAGTTCATGGCAG
AGTCTCCTATTGCTGGGAGTTTATCTGGGAAT
>seq259 Asamavirus_N10_EU929072

ATTGGATTTCGATTATCACATATCTGATAGCATTTGTGGATGTTATTCTTCTTAAAGGCCTTTATATG
CTAACTACCAGAGGTAGACAAACAATTAAGGACAACAAAGGGACAAGAATTAGGCTTAAAGATGA
CTCATCTTATATTGAAGTCAATGGGATCCGAAAACCTAAGCACTTATATATTTCCCTCCCTAATGCA
CAGTCAAGTATGAGAGCAGAGGAATTAACTCCAGGTAGGTACCGGACAGTGATTTGTGGATTGTAT
CCAGCACAAATAAAAGCAAAGAGTATGATTAGCCCTGTAATGGGAGTTATTGGATTCCCGAAATTG
TCTAAAGACTGGGATAAACGAATTAATGATTTTTAAAAGAAGATTGTCCATTTCTTCGACCTGTAG
CTTTCCAGCCCTTTTCTTTGACAGAT
>seq260 Novavirus_MSB95703_FJ539178
ACTGGGTTGCAATTTGTACTTACATTTTAGGATTTGGTGCTACAATTCTTCTCAAAGCACTTTATAT
GTTAACTACACGTGGCAGACAGACCATTAAAGAGCATAAGGGTAATCGGGTTCGATTTCGTGATGA
TTCATCATTTATTGAGAAAGGTGGTGTTAAAGTCCCAAAGCATCTTTATGTTTCACTTCCAACAGCT
CAATCTGCAATAAAGGCAGAAGAACTCACTCCAGGGAGATTCAGGACCATTGTTTGTGGATTATAC
CCCTCTGAGGCAAAAGTGCGCGGGCTGATAAGTCCTGTTATGGGAGTGATAGGCTTTCAATATATG
GCACAGAATTGGAGTGAAATGATTCATAAGTTCATGCATATGAGGTGCCCATTCATTGAAGCCCCT
AGCCTACAGGTTGATTCTGCTCTCTTA
>seq261 ProspectHill_M34011
ATTGGCTTAAGATTGGCAGCTACATCATAGAATTTGCACTACCTATCATCTTGAAAGCCTTGCATAT
GTTGTCAACTAGAGGGAGGCAAACTGTAAAAGAGAATAAGGGGACAAGGATCAGGTTCAAAGAT
GATAGTTCCTATGAAGATGTGAATGGCATTAGACGCCCAAAGCACCTTTATGTGTCTATGCCAACA
GCCCAGTCAACAATGAAAGCTGAGGAATTAACACCAGGGAGATTCAGGACAATTGTTTGTGGACT
ATTTCCTGCACAGATCATGGCAAGAAATATCATCAGTCCTGTAATGGGTGTGATCGGATTTGCATTT
TTTGTAAAGGATTGGGCTGACAAAGTAAAGGCATTTCTTGACCAGAAATGTCCATTCCTAAAGGCT
GAGCCACGTCCTGGACAGCCTGCCGGTGAA
>seq262 Soochong_SC-1_AY675349_mRNA
ACTGGCTGAGCATTGTTGTTTATCTTACATCCTTTGTGGTTCCGATACTCCTGAAAGCCCTTTACAT
GCTGACAACAAGGGGAAGACAGACAACTAAGGACAATAAAGGGACAAGAATCCGATTCAAAGAT
GACAGCTCTTTTGAAGATGTGAATGGAATTCGGAAGCCGAAACATCTCTATGTGTCTTTACCTAAT
GCACAGTCTAGTATGAAGGCAGAAGAGATAACACCTGGAAGGTACAGGACAGCAATCTGTGGATT
ATACCCTGCTCAGATTAAAGCCAGACAGATGGTAAGCCCAGTCATGAGTGTAATTGGATTCCTTGC
CTTGGCTAAAGACTGGGGTGATAGGATTGAGCAGTGGCTAAGTGAACCCTGTAAGCTCCTCCCAGA
CACAGCAGCAGTAAGCCTCCATGGTGGTCCT
>seq263 Tula_Sennickerode_Sen05/204_EU439950
ATTGGTTTTCGATTGGGCAATACATCATTGGGTTTGCACTGGCTATTGTCCTAAAGGCATTGTACAT
GTTGTCAACTCGTGGAAGGCAGACAATTAAAGAGAACAAAGGCACTAGAATTAGGTTTAAGGATG
ACAGCTCGTTTGAAGAAATTAACGGGATAAGGCGACCAAAGCATTTATATGTCTCAATGCCAACTG
CCCAATCCACCATGAAAGCTGATGAGTTAACACCAGGCAGGTTTAGGACAATTGTGTGTGGATTAT
TCCCTGCTCAGATAATGCACAGGAATATAATAAGTCCTGTCATGGGTGTGATTGGATTCTCCTTCTT
TGTCAAGGACTGGCCGGACAAGATTGAAGAATTTCTCATAAAACCTTGCCCATTCCTAAAGAAACA
AGGTGGCCCCAACAAGGATGAAGACTTT
>seq264 Tula_Kosice144/Ma/95_Y13979
ACTGGTTCTCCATTGGACAATACATTATAGGGTTTGCACTAGCAATAGTCCTAAAGGCATTATACA
TGTTATCAACAAGAGGAAGGCAGACAATTAAGGAAAACAAGGGGACTAGAATCAGGTTCAAAGAT
GACAGTTCCTTTGAGGAGATCAATGGCATACGGCGTCCTAAGCACCTCTATGTGTCAATGCCTACT
GCACAGTCCACTATGAAAGCTGATGAGTTAACACCAGGTAGGTTTAGAACAATTGTTTGTGGACTT
TTCCCGGCTCAAATCATGCACAGAAATATCATAAGTCCAGTCATGGGTGTTATTGGGTTTTCATTCT
TTGTGAAAGACTGGCCTGAGAAGATCGAAGAATTTTTAATTAAGCCTTGTCCTTTTTTAAAGAAAC
AAGGTGCACCTGGGAAAGAGGAAGACTTT
>seq265 Tula/Moravia/5293Ma/94_Z48574
ATTGGTTTTCTATCGGCCAGTACATTACAGGCTTTGCACTTGCAATAATCTTGAAGGCATTGTATAT
GCTGTCAACTAGAGGGAGGCAAACAATCAAGGAAAATAAGGGGACAAGAATCCGGTTCAAGGAT
GACAGCTCATATGAAGAGATCAATGGCATTAGACGCCCGAAACATCTGTATGTGTCTATGCCAACA
GCCCAGTCTACTATGAAAGCCGATGAATTGACACCAGGTAGGTTTAGAACAATTGTTTGTGGACTC
TTTCCTGCTCAAATTATGCACAGAAACATCATAAGTCCTGTCATGGGTGTGATTGGATTTTCTTTTTT
CGTTAAAGATTGGCCTGAAAAGATTGAGGAGTTCCTTATTAAACCCTGCCCATTCCTGAAGAAAAG
TGGTCCTAGTAAGGAAGAGGATTTTCTT
>seq266 Tula/MG23/Omsk/AF442621
ACTGGTTTTCTATCGGTCAGTACATAATGGGATTTGCACTTGCAATCGTGCTTAAGGCGTTGTACAT
GTTGTCGACACGCGGGAGACAAACCATCAAGGAAAATAAAGGGACAAGAATTAAGTTTAAAGATG
ACAGTTCAATTGAGGAGATTAACGGGATAAGACGTCCAAAACACCTGTATGTCTCAATGCCAACTG
CACAGTCCACAATGAAAGCAGATGAGTTAACACCAGGAAGATTTAGGACAATTGTCTGTGGCCTCT

TCCCTGCCCAAATAATGCATAGAAACATCATCAGTCCAGTAATGGGTGTAATAGGATTCTCTTTCTT
TGTCAAAGATTGGCCAGATAAGATTGAGGAGTTCCTTATTAAACCATGTCCCTTCTTGAAAAAACA
AGGTTCCACCAAAGAAGAAGAATTTCTT
>seq267 Thottapalayam_AY526097
ATTGGGGAAAGATATTCGAATACATTTTGACCCTTACACAGGTTCTTCTCCTTAAAGGCCTCTACAT
CTTGACCACCCGAGGAAGACAGACTAGTAAAGACAACAAAGGTACTAGAATCAAGCTTAAGGATG
ACTCATGCATGGAGGACCGGAATGGGATAAAACAACACAAGTACCTTTACATATCCCTTCCAACAA
GCCAATCTTCCATCCAAGATGATGAACTCACACCTGGACGTTTTAGGACAATGATCTCAGGACTCT
TGCCAAATGAGATAAAGGCAAAAAAGCTGATGAGCCCTGTAATGGGTGTCATTGGTTTTCAACATT
TAGCTGAGGCTTGGCCAGGTGCAATGGAAAAGATGTTGAGTGACCAGTGTGAATATATGACCAAG
GATAAAGCAAACCCATCAAATAGCACTAAT
>seq268 Andes_Chile-9717869_AF291702
ATTGGAAGGCTATAGGAGCATACATCTTAGGGTTTGCAATTCCGATCATCCTAAAGGCCTTATACA
TGCTGTCAACCCGTGGGAGACAAACTGTGAAAGACAACAAAGGGACCAGGATAAGGTTTAAGGAT
GATTCTTCCTTTGAAGAAGTCAATGGGATACGTAAACCAAAACACCTTTACGTCTCAATGCCAACT
GCACAGTCCACTATGAAGGCTGAAGAAATCACGCCAGGACGATTTAGGACAATTGCTTGTGGCCTT
TTTCCAGCACAGGTCAAAGCCCGAAATATAATAAGTCCTGTAATGGGAGTAATTGGATTTGGCTTC
TTTGTAAAGGATTGGATGGATCGGATAGAAGAGTTTCTGGCTGCAGAGTGTCCATTCTTACCTAAG
CCAAAGGTCGCCTCAGAAGCCTTCATGTCT
>seq269 Andes_NK104619_EU241691
ACTGGAAAGCAATAGGAGCATATATCCTAGGATTTGCAATCCCAATCATCTTAAAAGCCCTATACA
TGCTGTCAACTCGTGGGAGACAAACTGTGAAGGACAACAAGGGGACCCGGATAAGATTCAAGGAT
GATTCCTCCTTTGAAGAGGTGAACGGGATACGCAAACCAAAACATCTTTATGTCTCAATGCCAACT
GCACAATCCACAATGAAAGCTGAAGAGATCACACCAGGGCGGTTCAGGACAATTGCTTGTGGCCT
TTTTCCAGCACAGGTCAAAGCTAGGAACATAATAAGCCCTGTAATGGGTGTAATTGGGTTTGGTTT
CTTTGTGAAAGACTGGATGGACCGGATAGAAGAATTCTTGGCTGCAGAGTGCCCATTCTTGCCTAA
GCCAAAGGTAGCTTCTGAATCCTTTATGTCT
>seq270 SinNombre_NMR11_L37904
ATTGGAAATCCATCGGACTCTACATTCTAAGTTTCGCATTACCGATTATTCTTAAAGCCTTGTACAT
GTTATCTACTAGGGGCCGTCAAACAATCAAAGAAAACAAGGGAACAAGAATTCGATTCAAGGATG
ATTCATCTTATGAAGAAGTCAATGGGATACGTAAGCCAAGACATCTGTATGTTTCTATGCCAACTG
CCCAGTCTACAATGAAAGCAGATGAGATTACTCCCGGGAGGTTCCGTACAATTGCTTGTGGATTAT
TCCCAGCCCAAGTCAAAGCAAGGAATATTATCAGTCCTGTCATGGGTGTGATTGGCTTTAGTTTTT
TGTGAAAGATTGGATGGAAAGGATTGATGACTTCCTGGCTGCACGTTGCCCATTTCTGCCTGAGCA
GAAAGACCCTAGAGATGCTGCATTGGCA
>seq271 NewYorkhantavirus_nucleocapsid_U09488
ATTGGAAAGCTATTGGAATGTATATCTTGAGCTTTGCAATACCAATCATCTTAAAGGCCCTCTATAT
GTTATCAACCAGAGGTCGTCAAACAGTTAAGGAAAATAAGGGGACAAGAATCAGGTTCAAGGATG
ACTCATCTTATGAAGAAGTCAACGGAATCAGAAAGCCAAGACATTTATATGTATCAATGCCTACAG
CCCAATCCACAATGAAGGCAGATGAGATCACTCCAGGGCGTTTCCGCACGATTGCATGTGGATTGT
TCCCTGCACAAGTGAAGGCTAGGAATATTATCAGTCCAGTCATGGGTGTGATAGGATTTAACTTTTT
TGTTAAGGACTGGATGGACAGGATTGATGATTTTCTAGCTGAGCGGTGCCCATTCCTACCTGAGCC
AAGAGACCCTAAGGATGCTGCATTAGGA
>seq272 Choclovirus_segmentS_DQ285046
ATTGGAGGTCAATTGGTGCTTATATCCTCGGATTTGCACTACCAATCATCTTGAAAGCCCTCTACAT
GTTATCCACAAGAGGGAGGCAGACTGTGAAAGAAAACAAAGGCACCAGGATCAGATTTAAAGATG
ATTCATCCTTCGAAGAGGTAAATGGGATAAGAAAGCCTAAACATCTTTATGTGTCGATGCCCACTG
CACAGTCAACAATGAAGGCTGATGAAATAACACCTGGAAGGTTCCGGACAATTGCATGTGGCCTTT
TCCCAGCTCAAGTCAAAGCCAGGAATATAATTAGCCCAGTAATGGGTGTCATCGGTTTTGGTTTTTT
TGTAAAGGATTGGATGGATCGTATTGATAATTTCTTATCTGCTGAGTGTCCATTTTTGCAGAAGCCA
AAGGTTCCATCTGAGGCATTTACAACC
>seq273 LagunaNegra_AF005727
ATTGGAAGGCTATTGGAGCCTATATTCTAGGATTTGTAATACCAATTATCCTAAAGGCATTATATAT
GCTTTCAACAAGAGGGAGGCAGACTGTTAAAGAGAACAAAGGGACCAGGATTCGATTCAAGGATG
ATTCATCATTTGAAGAAGTCAATGGCATCCGAAAACCTAAACACTTGTATGTGTCAATGCCTACTG
CACAATCTACAATGAAGGCAGATGAGATAACACCGGGGAGGTTTAGGACAATTGCATGTGGCTTA
TTTCCTGCTCAAATCAAAGCTCGGAACATTATAAGTCCAGTCATGGGTGTCATTGGCTTTGGTCACT
TTGTGAAGGACTGGATGGAAAGGATTGACAACTTTCTAGGAGAAGATTGCCCATTTCTTCCAAAGG
CTAGGGTTCAATCAGAGGCATTCATGTCA

SUPPLEMENT 2

Hantavirus resequencing from animal reservoirs in Europe.

Flowchart of the experimental procedure for European hantavirus detection and genetic characterization using PathogenID v3.0 resequencing pan-viral chip.

Plasmids used for the preliminary assay were not subjected to the Retro-Transcription step. However they were processed by WGA (Whole Genome Amplification) as well as the cDNAs from extracted RNAs.

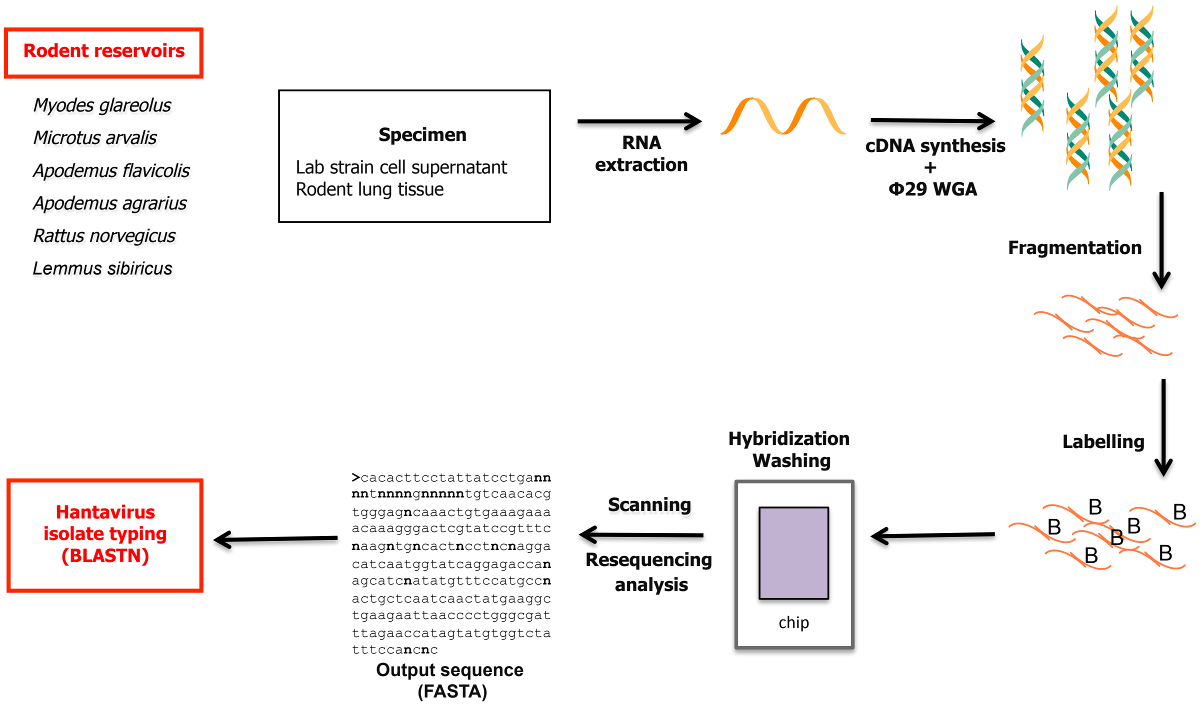

SUPPLEMENT 3

Validation of PathogenID v3.0 resequencing microarray using plasmids coding orthohantavirus N proteins: Call Rate and BLASTN analysis

Plasmids containing the S segment of Puumala virus (PUUV, strain Sotkamo 2009), Tula virus (TULV, strain Moravia) and Hantaan virus (HTNV, strain 76/118) were hybridized on the PathogenID v3.0 resequencing microarray separately (Assay 1) or in pool (Assay 2) to evaluate its potential in detection.

After hybridization of the plasmid(s) to sequences tiled on the array, the following parameters are indicated: (A) the Call Rate (CR: % of determined/total number of nucleotides) of the output sequence; (B) the raw results when BLASTN analysis allowed virus/variant identification including: Max score, Total score, Query coverage, E value, Identity. In case of multiple BLASTN results, sequence(s) with the highest values was(were) indicated, with corresponding accession number(s). In case of no BLASTN identification this was indicated.

Complementary informations are found in Table 2, Figure 2 and Supplement 4.

Assay 1
PUUV (sequences 222 - 243 on the chip)

>seq222:Hanta_Puumala_Plasmid_1_13-02-2013_(VirIDv3r520827) Start=12 End=412
ttggagtgtatgtgntagggttnacacttcctatcnnnnnnnnnnnnnnnnnnnnnnnnnnnacgcgtgggagacagnnnnnnnnnnnn
nnnnnnncnnnnnnnnnnnnnnnnnnnnnnnnnnnnnnnnnnnnnnnnnnnnnnnnnnnnnngnnnannnggnnnnnnnnn
nnctatgcnnnnnnnccngnnnnnnnnnnnannnnnnnnnnncnnnnnnnnnnnnnnnnnnnnnnnnnnnnnnnncnntnnnnn
nnnnnnnnnnnnnnnnnnnnnnnnncnnnnnnnnnnnnnnnngtcattggttttnnannnnnnnnnnnnnnnnnnnnnnnnnnn
nnnnnnnngnnnntggaaaaagagnnccnnnnnnnnnnnnnnnnnnnnnnncnnnncnnn
(A) CR (96/401): 23.9%
(B)
Max score: 57.2
Total score: 57.2
Query coverage: 8%
E value: 4e-04
Identity: 94%

- HE801633.1: Puumala virus N and Ns genes, segment S, genomic RNA, strain Sotkamo 2009.
- X61035.1: Puumala virus genomic RNA for nucleocapsid protein.

>seq223:Hanta_Puumala_Plasmid_1_13-02-2013_(VirIDv3r520827) Start=12 End=412
ttggagtgtatgngnnnngcttnacacttncnnncnnnnnnnnnnnnnnnnnnnnnnnnnnacgcgtgggagacagnnnnnnnnnnn
nnnnnnnnnnnnnnnnnnnnnnnnnnnnnnnnnnnnnnnnnnnnnnnnnnnnnnnnnnnnnnnggagaccaaagcnttnnnnnnntt
tctatgcctncnnncnngnnnnnnnnnnnnnnnnnnnnnnncnnnnnnnnnnnnnnnnnnnnnnnnnnntntgnncnnnnnnnnn
nnnnnnnnnnnnnnnnnnnncannannnnnnnnnnnnnnnnnnnnnnnnnnnnnnnnnnnnnnnnnnnnnngnnnnnnnnn
nnnnnnnnnnnnnnnggaaaaagagngccnnnnnnnnnnnnnnnnnnnnnnnancnnncacn
(A) CR (95/401): 23.7%
(B)
no BLASTN identification

>seq224:Hanta_Puumala_Plasmid_1_13-02-2013_(VirIDv3r520827) Start=12 End=412

tnnnannnnnnnnnnnnnnngnnnncacttnnnnnnnnnnnnnnnnnnnnnnnnnnnnnnnnnnnangcgtgggagacagactgnannn
nnnnnnnnnnnnnnnnnnnnnnnnnnnngnnnnnnnannnnnnnnannnnnnnnnnnnnnnanggagaccaaagcatnnannn
nnnnnnnnnnnnnnngcccagtcaactatgaaagcnnnnnnnnnnnnnnnnnnnnnnnnnnnnnnnnngnnnnnnnnnnnnnnnnn
ncnnnnnnnnnnnnnnnnnnnnnnnnnnnnnnnnnnntnnnnngnnnnntnnnnnnnnannnnnnnnnnnnnnnnnnnnnnnn
nnnnnnnnnnnnnnnnnnnnnnnnnnnnnnnnnnnnnnnnnnnnnnnnnnnnnnnnnnccca
(A) CR (76/401): 18.9%
(B)
no BLASTN identification

>seq225:Hanta_Puumala_Plasmid_1_13-02-2013_(VirIDv3r520827) Start=12 End=412
ttggagtgtatgtgatagggttcacacttcctatcatccttaaagctttatacatgctctcaacgcgtgggagacagactgtaaaggaaaataaggga
cacgtataaggtttaaggatgatacatcatttgaagacatcaatggcataaggagaccaaagcatttatangtttctangcntanngcccagtcaacta
tgaaagcagaagaacncanaccaggcagatttcgcacaatagtatgtggtctttttcccactcagatccaggttcgtaacatcatgagtccagttatgg
nggtcattggtttttcattctttgtgaaggattggtctgagagaatcagagagttcatggaaaaagagtgcccattcataaagcctgaagnaaaaccag
gcaca
(A) CR (392/401): 97.7%
(B)
Max score: 691
Total score: 691
Query coverage: 100%
E value: 0.0
Identity: 98%
- HE801633.1: Puumala virus N and Ns genes, segment S, genomic RNA, strain Sotkamo 2009.

>seq226:Hanta_Puumala_Plasmid_1_13-02-2013_(VirIDv3r520827) Start=12 End=412
nnnnnnngnnnnnnnnnnnnnnnnnnnnnnnnnnnnnnnnnnnnnnnnnnnnnnnnnnnnngngtgggngacagnntnnn
nngnnnnnnnnnnngnnnnnnnnnnnnnnnnnnnnnnnnnnnnnnnnnnnnnnnngcatanggagaccnnngnnnnn
nnnnnnnnntnngcctactgcccagtcanctannanngnagaagaanncnnnnnnnnnnnnnnnncncnatnntntgnnnnnnnnnn
nnnnnnnnnnnnnnnnntngtaacatcatgagtccagttnnnnnnnncnnnnnnnnnnnnannnnnnnnnnnnnnnnnnnnnnnnn
nnnnnnnnnnnnnnnnnnnnnnnnnnnnnnnnannnataaagcctgaannannnncnnnnnca
(A) CR (105/401): 26.2%
(B)
no BLASTN identification

>seq227:Hanta_Puumala_Plasmid_1_13-02-2013_(VirIDv3r520827) Start=12 End=412
ttggagtgtatgtgatnnnnnncnnnnnnnnnnnnnnnnnnnnnnnnnnnnnnnnnnncnnannnnnngggagacagncnnnnnnn
nnnnnnnngnggacacgtannnggnttaagnatgntnnannnnnnnnnagacatcaatgncanangnagaccaaagnnnnnnnnnnntn
nnntgcctactgcccagtcanctatgaaagcagnagaacncnnnnnnnncnnatttcgcacaatagtanncnnnnnnnnnnnnnnnnnnn
nnnnnnnnnnnnnnnnnnnnnnccagttatgnnnnnnnnnnnnnnnnnnnnnnnnnnnnnnnnnnnnncnnnnnnnnnnnnnnnn
nnncnnnnnnnnanngnnnnnanncntanannntnaagtaaanncanncncn
(A) CR (159/401): 39.6%
(B)
Max score: 91.5
Total score: 91.5
Query coverage: 36%
E value: 2e-14
Identity: 67%
- HE801633.1: Puumala virus N and Ns genes, segment S, genomic RNA, strain Sotkamo 2009.
- X61035.1: Puumala virus genomic RNA for nucleocapsid protein.

>seq228:Hanta_Puumala_Plasmid_1_13-02-2013_(VirIDv3r520827) Start=12 End=412
ttgganngnnnnnnnnnnnctttncnnnnnnnnnnnnnnnnnnnnnnnnnnnnnnnnnnnnnnnnnnnnngnnnngnnnnnnn
nnnnnnnnnnnnnnnnnnnnnnnangtttaaggatgatacatcatttgaagacatcaatggcannnnnnnnnnannncannnnnnnnntn
nnncnnntnctgcccagtcaactatnnnnnnnnnnnnnnnnnnnnnnnnnnnnnnnnnnnnnnnnnnntnnnannnnnnnnnntnnnnnnnn

nnnnnnnnnngnnnnnnnnnnnnnnnnnnnntnnnnnnnnnnnnnnnnnnnnnnnnnnnnnngnnggattggtntnnnnnnnnn
nnnnnnnnnnnnnnnnnnnnnnnnnnnnnnnnnnnnnnnnnnnngnnnnnnncnnn
(A) CR (91/401): 22.7%
(B)
Max score: 69.8
Total score: 69.8
Query coverage: 9%
E value: 7e-08
Identity: 100%
- HE801633.1: Puumala virus N and Ns genes, segment S, genomic RNA, strain Sotkamo 2009.
- AJ314597.1: Puumala virus mRNA for nucleocapsid protein (N gene), strain Pallasjarvi/63Cg/98.
- Z30705.1: Puumala virus gene for N protein, genomic RNA, strain Evo/15Cg/93.
- Z30704.1: Puumala virus gene for N protein, genomic RNA, strain Evo/14Cg/93.
- Z30702.1: Puumala virus gene for N protein, genomic RNA, strain Evo/12Cg/93.
- X61035.1: Puumala virus genomic RNA for nucleocapsid protein.

>seq229:Hanta_Puumala_Plasmid_1_13-02-2013_(VirIDv3r520827) Start=12 End=412
nnnnnnngnnnnnnnnnnnnctttacanntnnnnnnnnnnnnnnnnnnnnnnnnnnnnnnnnnngnnnnnggnnnngnnnnnnn
nnnnnnnnnnnnnnnnnnnnnnnngtttaaggatgatacancnnnnnnanacannnntngnnnnnnnnnnnnnngnnnnnnnn
nnnnnnnnnnnnnnnnncnngnnnannnnnnnnannnnnnnnnnnnnannnnnnnnnnnnnnnnnnnnnnnnnnnnnnnnngnn
nnncnnctcagatnnngnnnnnnnnnnnnnnnnnnnnnnnnnngnngnnnnnnnnnnnnnnnnnnnnnnngnnnnnnnnnnn
nnnnnnnnnnnnnngntcatggaaaaagagngccnannnnnnnnnnnnnnnnnnnnnncnnncnca
(A) CR (78/401): 19.4%
(B)
no BLASTN identification

>seq230:Hanta_Puumala_Plasmid_1_13-02-2013_(VirIDv3r520827) Start=12 End=412
ttggagtgtatgtgnnngggttnacacttcctancnnnnnnnnnnnnnnnnnnnnnnnnnnnacgcgtgggagacagactgnnnnnnnnn
nnnnnnnnnnnnnnnnnnnnnnnnnnnnnnnnnnnnnnnnnnnnncannnnncantgncananggagaccaaagcnttnnnnnnnnnc
tatgcntnnnncccngncnnnnnnnnnannnnnnnnnnnnnnnnnnnnnnnnnnnnnnnnnnnnngnatgtggtctttnnnnnnnnnn
nnnnnnnnnnnnnnnnnncnnnnnnnnnnnnnnnnngtcattggttnnnnnnnnnnnnnnnnnncnnnngncnnnnnnnnnnnna
nngttcatggaaaaagagngccnnnnnnnnnnnnnnnncncnnncnnnncncn
(A) CR (136/401): 33.9%
(B)
Max score: 46.4
Total score: 46.4
Query coverage: 8%
E value: 0.78
Identity: 88%
- HE801633.1: Puumala virus N and Ns genes, segment S, genomic RNA, strain Sotkamo 2009.
- X61035.1: Puumala virus genomic RNA for nucleocapsid protein.

>seq231:Hanta_Puumala_Plasmid_1_13-02-2013_(VirIDv3r520827) Start=12 End=412
nnnnnnngnnnnngnnnnggttcacnctnnntnncnnnnnnnnnngctttnnannngctctcaacgngtgggagacagactgtaaaggnan
ataaggggacacgtataaggtttaaggatgatacatcatttgaagacatcaatggcataaggagaccaaagcatttatatgtttctatgcctactgccca
gtcaactatgaaancagaagaactcacaccaggcagatttcgcncaatagtatgtggtcnttttnncnnnnnnnnnnnnnntcgtaacatcatgag
tccagttatgggnnncannnntnnnnnnnncnnnnngnnngantggtctgagagaatcagagagttcatggaaaaagagtgcccattcataaa
gnntnnnnnnnnnnnnnncnna
(A) CR (301/401): 75%
(B)
Max score: 423
Total score: 423
Query coverage: 83%
E value: 3e-114

Identity: 85%

- HE801633.1: Puumala virus N and Ns genes, segment S, genomic RNA, strain Sotkamo 2009.

>seq232:Hanta_Puumala_Plasmid_1_13-02-2013_(VirIDv3r520827) Start=12 End=412
nnnnngtgnatgtnnnngggttcacacnnnnnnnnnnnnnnnnnnnnnnnnnnnnnnnnnngnnnnngggncagactgtnaan
nnaannnnnnnnnnnnnnnnnnnnnnnnnnnnnnnntncntcatttgaagacatcaatggcatanggagaccaaagcatnnnnnnnnntnn
nnnnnnnnnnnncnnnncaactatgaaagcagnnnnnnnnnnnnnnnnnnnnnnnnncncaatagtatgnngtnnnnnnnnnnnn
nnnnnnnnnnnnnnnnnncnngagtccagttatggnggtcattnntntnnnannnnnnnnganggnnnnnnnnnnnnnnnnnnn
nnnnnnnnnnnnnnnnnnnnnnannnnnnnnnnnntnnnnnnnnnncagncnnn
(A) CR (138/401): 34.4%
(B)
Max score: 68.0
Total score: 68.0
Query coverage: 9%
E value: 2e-07
Identity: 97%

- HE801633.1: Puumala virus N and Ns genes, segment S, genomic RNA, strain Sotkamo 2009.
- GU808825.1: Puumala virus strain Kuhmo/X11 nucleocapsid protein (N) mRNA, complete cds.
- GU808824.1: Puumala virus strain Kuhmo/X5 nucleocapsid protein (N) mRNA, complete cds.
- AJ314597.1: Puumala virus mRNA for nucleocapsid protein (N gene), strain Pallasjarvi/63Cg/98.
- AJ238789.1: Puumala virus RNA for nucleocapsid protein, strain Kolodozero.
- AJ238788.1: Puumala virus RNA for nucleocapsid protein, strain Karhumaki.
- X61035.1: Puumala virus genomic RNA for nucleocapsid protein.

>seq233:Hanta_Puumala_Plasmid_1_13-02-2013_(VirIDv3r520827) Start=12 End=412
tnnnannnnnnnnnnnnngctttacacnnnnnnnnnnnnnnnnnnnnnnnnnnnnnnncncnnnacgcgtgggagacagactgtaaagg
aaaataagggnnnnnnnnnnnnnnnnnnnnnnnntnnannnnnnnnnannnnncnnnnnnnnanggngacnnnnnnntnnnnnnnn
nnnnnnnnnnnnnnnnnnnnnnnntnnnnaagcagannnnnnnnnnnnnnnnnnnnnnnnnnnnnnannnnnnnnnnnntnntnnnnn
nnnnnnnnnnnnnnnnnnnnnnncnnnnnnnnnnnntnnnnnnnnnngtnnnnnnnnnnncnnnnngnnnnnnnnnnnnnnn
nnnnnnnnnnnnnnnnnnnnnnnnnnnnnnnnnnnnnnnnnnnnnnnnnnnnnnnnnanccnnn
(A) CR (79/401): 19.7%
(B)
Max score: 64.4
Total score: 64.4
Query coverage: 8%
E value: 3e-06
Identity: 100%

- HE801633.1: Puumala virus N and Ns genes, segment S, genomic RNA, strain Sotkamo 2009.
- AJ238790.1: Puumala virus RNA for nucleocapsid protein, strain Gomselga.
- X61035.1: Puumala virus genomic RNA for nucleocapsid protein.

>seq234:Hanta_Puumala_Plasmid_1_13-02-2013_(VirIDv3r520827) Start=12 End=238
acagntnnnnnnnngnntataacccgccatgnanngnnnnnnanngttgccagacaaaaacntnnnnnnnnnnnnnnnnnnnnnnnnag
tggacccagatgacgttaataannacanacngcaagccnggcaacaaacagtgtcagcacnggaggacaaactcgnannnnannnnannng
gatggcagatgctgngnncnnnnnnnnnanngg nnnnnaannnnactga
(A) CR (141/227): 62.1%
(B)
Max score: 129
Total score: 129
Query coverage: 47%
E value: 4e-26
Identity: 83%

- HE801633.1: Puumala virus N and Ns genes, segment S, genomic RNA, strain Sotkamo 2009.
- X61035.1: Puumala virus genomic RNA for nucleocapsid protein.

>seq235:Hanta_Puumala_Plasmid_1_13-02-2013_(VirIDv3r520827) Start=12 End=412
nnnnnnnnnnnnnnnnnnnnnnnnnnnntnnnnnnnnnnnnnnnnnnnnnnnnnnnncnnnnnnnnnnnnnnnnnnnnnn
nnngnnnnnnanggggnnnnnnnnnnnnnnnnnnnnnnnnnnnnnnnnnnnnnnnnnnnnnnnnnnnnnnnnnnnnnnnn
nncnnnnnnnnnnnnnnnnnnnannnnnnnnnnnnnnnnnnnnagaactcacaccanncnnnnnnnnnnnnnnnnnnnnc
nnnnnnnnnnnnnnnnnnnnnnnnnnnnnnnnnnnnnnnnnnnnnnnnnnnnnnnnnnnnnnnnnnnnnnnnnnncnnnnnn
nnnnnnnnnnnnnnnnnnnnnnnnnnnnnnnnnnnnnnnnnnnnnnnngnnnnnnnnnnnnnnnnncnnn
(A) CR (28/401): 7.0%
(B)
no BLASTN identification

>seq236:Hanta_Puumala_Plasmid_1_13-02-2013_(VirIDv3r520827) Start=12 End=412
nnnnanngnnnnnnnnnngcttnacacntnnnnnnnnnnnnnnnnnnnnnnnnnnncncnnnacgcgtgggagacagactgtaaagg
aaaataaggggnnnnnnnnnnnnnnnnnnnnnntnnnnnnnnnnnnnnnnnnnnnnnnnnggnnaccannnnntnnnnnnnn
nnnnnnnnnnnnnnnnnnnnnnnnnnnnnnnnnnnnnnnnnnnnnnnnnnnnnnnnnnnnnnannagtatgtggtcttttnncc
nnnnnnnnnnnnnnnnnnnnnnnnnnnnnnnnnnnnnnnnnnnnnnnnnnnnnnnnnncnnnngnnnnnnnnnnnnnnnn
nnnnnnnnnnnnnnnnnnnnnnnnnnnnnnnnnnnnnnnnnnnnnnnnnnnnnnnanccnnn
(A) CR (80/401): 19.9%
(B)
Max score: 66.2
Total score: 66.2
Query coverage: 8%
E value: 8e-07
Identity: 100%
- HE801633.1: Puumala virus N and Ns genes, segment S, genomic RNA, strain Sotkamo 2009.
- AJ238790.1: Puumala virus RNA for nucleocapsid protein, strain Gomselga.
- X61035.1: Puumala virus genomic RNA for nucleocapsid protein.

>seq237:Hanta_Puumala_Plasmid_1_13-02-2013_(VirIDv3r520827) Start=12 End=412
tggagtgtatgngntngggttnacacttcctatcnnnnnnnnnnnnnnnnnnnnnnnnacgcgtgggagacagactgnannnnnnnnn
nnnnnnnnnnnnnnnnnnnnnnnnnnnnnnnnnnnnnnnncannnnnnnatggcananggagaccaaagcnttnnnnnnnnttctat
gccnnnnncccngtcaacnnnnnnnnnnnnnnnnnnnnnnnnnnnnnnnnnnnnnnnnnnnagtatgtggtcttnnnnnnnnnnnnn
nnnnnnnnnnnnnnnnnnnnnccnnnnnnnnnnnnnnnnnnnnngtcattggtttnnnannnnnnnnnnnnnnnnnnnnncnnnnnnnnnnnnnnnn
nttcatggaaaaagagngccnnnnnnnnnnnnnnnnnnnnnnnnnccnnncncnn
(A) CR (141/401): 35.2%
(B)
Max score: 48.2
Total score: 90.9
Query coverage: 22%
E value: 0.22
Identity: 88%
- HE801633.1: Puumala virus N and Ns genes, segment S, genomic RNA, strain Sotkamo 2009.
- X61035.1: Puumala virus genomic RNA for nucleocapsid protein.

>seq238:Hanta_Puumala_Plasmid_1_13-02-2013_(VirIDv3r520827) Start=12 End=290
attggagngnnnnngnnnngcttnacacttcctnncnnnnnnnnnnnnnnnnnnnnnnnnnnnnacgcgtgggagacagactgnnnnnn
nnnnnnnnnnnnnnnnnnnnnnnnnnnnnnnnnnnnnnnnnnnnnnnannnnnnnnnnnncnnnnnnnnnnnannnnnnnnnnnn
nnnnnntnnncnnnnnnncccngtcannnnnnnnnnnnnnnnnnnnnnnnnnnnnnnnnnnnnnnnnnnnnannagtatgtggtctttnnnn
nnnnnnnnnnnnnnnnnnnnnn
(A) CR (69/279): 24.7%
(B)
no BLASTN identification

>seq239:Hanta_Puumala_Plasmid_1_13-02-2013_(VirIDv3r520827) Start=12 End=290

attggagtgtatgtgntagggttnacacttcctatcnnnnnnnnnnnnnnnnnnnnnnnnnnnacgcgtgggagacagactgnannnnnnn
nnnnnnnnnnnnnnnnnnnnnnnnnnnnnnnnnnnnnnnnnncannnancnatgncanannnnnnnnnnnngngnnnnnnnnnn
nctatgccnncngcccagtnancnnnnnnannnnnnnnnnnnnnnnnnnnnnnnnnnnnnnnnnnannnnnatgtggtctttnnnnnnnnn
nnnnnnnnnnnnnnnn
(A) CR (96/279): 34.4%
(B)
Max score: 59.0
Total score: 59.0
Query coverage: 12%
E value: 8e-05
Identity: 94%

- HE801633.1: Puumala virus N and Ns genes, segment S, genomic RNA, strain Sotkamo 2009.
- X61035.1: Puumala virus genomic RNA for nucleocapsid protein.

>seq240:Hanta_Puumala_Plasmid_1_13-02-2013_(VirIDv3r520827) Start=12 End=290
attggagtgtatgtgnnngggttnanacttnctatcannnnnnnnnnnnnnnnnnnnnnnnnnnacgcgtgggagacagnnnnnnnnnnn
nnnnnnnnncnnnnnnnnnnnnnnnnnnnnnnnnnnnnnnnnnnnnannnnncaatggcananggagaccaaagcnttnnnnnnntttct
atgcctnnnncccngtcaannnnnnnannnnnnnnnnnnnnnnnnnnnnnnnnnnnnnnnnnnnnntatgtggtctttttcncnnnnnnnn
nnnnnnnnnnnnn
(A) CR (107/279): 38.3%
(B)
Max score: 51.8
Total score: 98.2
Query coverage: 33%
E value: 0.012
Identity: 75%

- HE801633.1: Puumala virus N and Ns genes, segment S, genomic RNA, strain Sotkamo 2009.
- X61035.1: Puumala virus genomic RNA for nucleocapsid protein.

>seq241:Hanta_Puumala_Plasmid_1_13-02-2013_(VirIDv3r520827) Start=12 End=290
attggagtgtatgngnnnggnttnacacntnnnnnnnnnnnnnnnnnnnnnnnnnnnnnnnnnnnacgcgtgggaganagnnnnnnnnn
nnnnnnnnnnnncnnnnnnnnnnnnnnnnnnnnnnnnnnnnnnnnnnnnnncnnnnnnnaanggcananngngnncannnnnnnnnnn
nncnnctatgcctncnnncnngnnnnnnnnnnnnnnnnnnnngnnnnnnnnnnnnnnnnnnnnnnnnnnnnnngnatgtggtctttnnnnn
nnnnnnnnnnnnnnnnnnnnn
(A) CR (75/279): 26.9%
(B)
no BLASTN identification

>seq242:Hanta_Puumala_Plasmid_1_13-02-2013_(VirIDv3r520827) Start=12 End=290
nnnnnnnnnnnnnnnnnnngnnttnnnnnntnnnnnnnnnnnnnnnnnnnnnnnnnnnnnnnnncnnnacgcgtgggagncagnctgtaaa
ggaaaatanggggacacgtatnnggnnnnnnnnnnnnnnnnnnnnnnnannnnnnnnnnnnnnnnnnnngaccaaagcatnnnnnnn
nnnnnnnnnnnnnnnnnnnnnnaactatgaaagcagnnnnnnnnnnnnnnnnnnnnnnnnnnnnnnnnnagtatgtggtcntnnnnnn
nnnnnnnnnnnnnnnnnnnn
(A) CR (86/279): 30.8%
(B)
Max score: 69.8
Total score: 69.8
Query coverage: 15%
E value: 5e-08
Identity: 93%

- HE801633.1: Puumala virus N and Ns genes, segment S, genomic RNA, strain Sotkamo 2009.
- AJ238790.1: Puumala virus RNA for nucleocapsid protein, strain Gomselga.
- X61035.1: Puumala virus genomic RNA for nucleocapsid protein.

>seq243:Hanta_Puumala_Plasmid_1_13-02-2013_(VirIDv3r520827) Start=12 End=290
attggagngnnnnnnnnnggctttacacttnnnnncnnnnnnnnnnnnnnnnnnnnnnncnnnnngnnnnnncnnnnnnnnnnnnn
nnnnnnnnnnnnnnnnnnnnnnnnnnnnnngnnnnnnnnannatnnnnnnncnnnnnnnnnnnnnnnnnannnnnnnnnnnnn
ncnnnnntntgcctactgcccagtcaactannnnannnnnnnnnnnnnnnnnnnnnnnnnnnnnnnnnnnnnnangtggtcttttnccc
nnnnnnnannngnnnnnnnnn
(A) CR (70/279): 25.1%
(B)
no BLASTN identification

Assay 2
PUUV+HTNV+TULV
(PUUV: sequences 222 - 243 on the chip)
(HTNV: sequences 249 - 254, 256 on the chip)
(TULV: sequences 263 - 266 on the chip)

>seq222:Hantavirus_Puumala_2_13-02-2013_(VirIDv3r520827) Start=12 End=412
ttggagtgtatgtgntagggttnacacttcctatcnnnnnnnnnnnnnngnnnnnncnnncnacgngngggagncagnnnnnnnnnnnn
nnnnnnnnnnnnnnnnnnngnnnnngnnnnnnnnnnnnnnnnnnnnnnnnnnnncnnnnnanngnnnnnannnnnnnnnnnnnntt
nctatgcctnntncccngnnnannnnnnnancnnnnnngnnnnnnnnnnnnnnnnnnnnnnnnnnnnnnnnnnntnnnnntgnnnnnnn
nnnnnnnnnnnnnnnnnnnnnnccnnnnnnnnnnnnnnnnngtcattggtttttnannnnnnnnnnnnncnnnnnncnnnnannnnnn
nnnngnncatggaaaaagagtnccnannnnnnnnnnnnnnnnnnnnnnnnnnnncnnn
(A) CR (114/401): 28.4%
(B)
Max score: 57.2
Total score: 57.2
Query coverage: 8%
E value: 4e-04
Identity: 94%
- HE801633.1: Puumala virus N and Ns genes, segment S, genomic RNA, strain Sotkamo 2009.
- X61035.1: Puumala virus genomic RNA for nucleocapsid protein.

>seq223:Hantavirus_Puumala_2_13-02-2013_(VirIDv3r520827) Start=12 End=412
ttggagtgtangngnncngcttngcacttncnntcnnnnnnnnnnnnanngtatatgctgacaacgngngggagncagnnnnnnnnnnnn
nnnnnnnnnnnnncnnnnnnnnnnnnnnnnnnnnnnnnnnnnnnnnnnnnncnccnnanggagaccaaagcnttnnnatgtttcta
tgcctncnncccngnnnngnnngnnnncannnnngnncnnnnnnnnnnnnnnnnnnnnnannngtatgtggtcttnnnnnnnnnnnn
nnnnnnnnnnnnanatcatgnnnnnnnncnnnnnnnnnnnnnnnnnntnnntntttcgttaaagntngnncnnnnannnnnnnnnnnnn
nnnnganaaagagtgccnannnnnnnnnnnnnnnnnnnncancnnccncn
(A) CR (153/401): 38.1%
(B)
Max score: 46.4
Total score: 46.4
Query coverage: 9%
E value: 0.78
Identity: 82%
- HE801633.1: Puumala virus N and Ns genes, segment S, genomic RNA, strain Sotkamo 2009.
- X61035.1: Puumala virus genomic RNA for nucleocapsid protein.

>seq224:Hantavirus_Puumala_2_13-02-2013_(VirIDv3r520827) Start=12 End=412
tnnnannnnnnnnnnnnnngnnnncacttcntnnnnnnnnnaaggcatnnnnnnnncngnnnangcgtgggagacagactgtannnnn
nnnnnnnnnnnnnganncnnnnnnnagnnnnntnnancntnnnnannnnncnnnnncnnaaggagaccaaagcatctacnngtgnn
tnnnnnnnnngcccagtcaactatgaaagcannnnnnnnnnnnnnnnnnnnnnnnnnnnnnnnngnnnnnnnnnnnnnnnnnnnnnn
nnnnnnnnnnnnnnnnnnnnnnnnnnnnnnnnnncnnnnnnnnnnntnnnnttnnanncnnnnngnnnnnnnnnnnnnnnnnnnn
nnnnnnnnnnnnnnnnnnnnnnnnnnnnnnnnnnnnnnnnnnnnnngnnnnncncn

(A) CR (107/401): 26.7%
(B)
Max score: 44.6
Total score: 44.6
Query coverage: 14%
E value: 2.7
Identity: 72%

- HE801633.1: Puumala virus N and Ns genes, segment S, genomic RNA, strain Sotkamo 2009.
- X61035.1: Puumala virus genomic RNA for nucleocapsid protein.

>seq225:Hantavirus_Puumala_2_13-02-2013_(VirIDv3r520827) Start=12 End=412
ttggagtgtatgtgatagggttcacacttcctatcatccttaaagctttatacatgctctcaacgcgtgggngacagactgtaaaggaaaataaggga
cacgtataaggtttaaggatgatacatcatttgaagacatcaatggcataaggagaccaaagcatttatatgtttctatgccnactgcccagtcaactat
gaaagcagaagaactcacaccaggcagatttcgcacaatagtatgtggtctttttcccactcagatccaggttcgnaacatcatgagtccagttatgg
nggtcattggtttttcattctttgtgaaggattggtctgagagaatcagagagttcatggaaaaagagtgcccattcataaagcctgaagnaaaaccng
gcaca
(A) CR (395/401): 98.5%
(B)
Max score: 702
Total score: 702
Query coverage: 100%
E value: 0.0
Identity: 99%

- HE801633.1: Puumala virus N and Ns genes, segment S, genomic RNA, strain Sotkamo 2009.

>seq226:Hantavirus_Puumala_2_13-02-2013_(VirIDv3r520827) Start=12 End=412
ttnnnnngnnnnnnnnnnnnnnnnnnnnnnnnnnnnnnnnnnnnnnnnnnnnnncncnnnnngngtgggngncagnntgnnn
ngnnnnnnnnnnnggnnnnnnnnnnnnnnnnnnnnnnnnnnnnnnnnnnnnnnnnnnnggcataagnnaaccnnagnnntnnn
nnnntnntntgcctactgcccagtcnannatgaaagcngangaanncnnnnnnnnnnnnnnnnncnnaatagtatgnnnnnntnnnnnnn
nnnnnnnnnnnnnntngtaacatcatgagtccagttatnnnnnncattnnanntncannnnnnnnnnnnnnnnnnnnnnnnnnnnnnnnnnn
nnnnnnnnnnnnnnnnnnnngnnannnataaagcctgaannnnnnnnanncnca
(A) CR (128/401): 31.9%
(B)
Max score: 46.4
Total score: 46.4
Query coverage: 8%
E value: 0.78
Identity: 86%

- KT885052.1: Puumala virus strain CG1820/POR segment S, complete sequence.
- HE801633.1: Puumala virus N and Ns genes, segment S, genomic RNA, strain Sotkamo 2009.
- GQ339478.1: Puumala virus strain Jockfall/Mg12/05 segment S, complete sequence.
- EU652442.1: Puumala virus isolate Tyumen/223_Myod_gl/06 nucleocapsid protein (N) gene, partial cds.
- EU652436.1: Puumala virus isolate Tyumen/198_Myod_gl/06 nucleocapsid protein (N) gene, partial cds.
- EU652434.1: Puumala virus isolate Tyumen/195_Myod_gl/06 nucleocapsid protein (N) gene, partial cds.
- EU652432.1: Puumala virus isolate Tyumen/185_Myod_gl/06 nucleocapsid protein (N) gene, partial cds.
- EU652431.1: Puumala virus isolate Tyumen/181_Myod_gl/06 nucleocapsid protein (N) gene, partial cds.
- EU652428.1: Puumala virus isolate Tyumen/170_Myod_gl/06 nucleocapsid protein (N) gene, partial cds.

- EU652427.1: Puumala virus isolate Tyumen/165_Myod_gl/06 nucleocapsid protein (N) gene, partial cds.
- EU563003.1: Puumala virus isolate Orenburg/33_Human/06 nucleocapsid protein (N) gene, partial cds.
- EU549815.1: Puumala virus isolate Ufa/05_Human/05 nucleocapsid protein (N) gene, partial cds.
- EU549814.1: Puumala virus isolate Ufa/04_Human/05 nucleocapsid protein (N) gene, partial cds.
- EU549813.1: Puumala virus isolate Ufa/03_Human/05 nucleocapsid protein (N) gene, partial cds.
- EU549812.1: Puumala virus isolate Ufa/02_Human/05 nucleocapsid protein (N) gene, partial cds.
- EU549810.1: Puumala virus isolate Izhevsk/66_Human/05 nucleocapsid protein (N) gene, partial cds.
- AB297665.2: Puumala virus strain: DTK/Ufa-97 viral cRNA, small segment, complete sequence.
- AF442613.1: Puumala virus isolate CG17/Baskiria-2001 nucleocapsid protein gene, complete cds.
- AF411448.1: Puumala virus isolate CG28 nucleocapsid protein N gene, partial cds.
- DQ064684.1: Puumala virus isolate Izh/542-Hs/04 nucleocapsid protein (N) gene, partial cds.
- DQ064654.1: Puumala virus isolate Izh/508-Hs/04 nucleocapsid protein (N) gene, partial cds.
- AY217102.1: Puumala virus isolate Fushun28 nucleocapsid protein mRNA, partial cds.
- AY197740.1: Puumala virus Fusong48 nucleocapsid protein mRNA, partial cds.
- Z84204.1: Puumala virus gene for N protein, genomic RNA, strain Puu/Kazan.
- Z30708.1: Puumala virus gene for N protein, genomic RNA, strain Udmurtia/338Cg/92.
- Z30707.1: Puumala virus gene for N protein, genomic RNA, strain Udmurtia/458Cg/88.
- Z30706.1: Puumala virus gene for N protein, genomic RNA, strain Udmurtia/444Cg/88.
- Z21497.1: Puumala virus gene encoding N protein, genomic RNA, strain Udmurtia/894Cg/91.
- M32750.1: Puumala virus CG1820 segment S nucleocapsid protein mRNA, 5' end.
- L08804.1: Hantavirus Puumala nucleocapsid protein, complete cds.
- L11347.1: Hantavirus Puumala P360 nucleocapsid protein mRNA, complete cds.
- X61035.1: Puumala virus genomic RNA for nucleocapsid protein.

>seq227:Hantavirus_Puumala_2_13-02-2013_(VirIDv3r520827) Start=12 End=412
ttggagtgtatgtgatnnnnnncnnnnntnnnnnnnnnnnnnnnnnntnnnnnnnnnnncnnanngnnngggagncagnnnnnnnnn
nnnnnnngnggncnngtannnggtttaaggatgntnnannnnnngaagacatcaatggcatangnagaccanannnnnnnnnnnntnnn
atgccaactgcccagtcnncnatgaaagcagnanaacnctnnnnaggnaaatttcgnacaatagtanncnnnnnnnnnnnnnnnnnnn
nnnnnnnnnnnnnncnnnnntccagttatgcnnnnnnnnnnannnnnannnnnnnnnnnnnnnnnnnnnnnnnnnnnnnnnnnnn
ncnnnnnnnnanngnnnncatncataaannntnaagtaaaaccnnncncn
(A) CR (172/401): 42.9%
(B)
Max score: 87.8
Total score: 87.8
Query coverage: 33%
E value: 3e-13
Identity: 69%

- HE801633.1Puumala virus N and Ns genes, segment S, genomic RNA, strain Sotkamo 2009.

>seq228:Hantavirus_Puumala_2_13-02-2013_(VirIDv3r520827) Start=12 End=412
ttggagngnnnnnnnnnnnctttncncnnnnnnnnnnnnnnnnnnnnnnnnnnnnnnnnnnnnnnnnnnnngnnnngnnnnnnnn
nnnnnnnnnnnngnnnnnnnnnangtttaaggatgatacatcatttgaagncatcaatggcannnngnnnnnanngcatttntnngntnntn
nnccaacngcccagtcaannntgannncanannnnnannnancaggtaggttnannnnnnnnnnnngngntntttttnccnnncagnncc
nnnngcgnnncgncnnnnnnnnnnnnnnnnnnnnnnnnnnnnnnnnnnnnnnnnnnngnnggattggtctnnnnnnnnnnnnnnnn
nnnnnnnnnnnnnnnnnnnnnnnnnnnnnnnnnnnnnnnngnnnnanncncn
(A) CR (136/401): 33.9%
(B)
Max score: 71.6
Total score: 71.6
Query coverage: 21%

E value: 2e-08
Identity: 73%
- HE801633.1: Puumala virus N and Ns genes, segment S, genomic RNA, strain Sotkamo 2009.
- X61035.1: Puumala virus genomic RNA for nucleocapsid protein.

>seq229:Hantavirus_Puumala_2_13-02-2013_(VirIDv3r520827) Start=12 End=412
nnggnnngnnnnnnnncnnctttncacttnnnnncnnnnnnnnnnnnnnnnnnnncnnnnnnngnnnnnnggnnnnnnnnnnnn
nnnnnnnnnnnggnnccngntncgatttaaggatgatacatcattnnnanacatnnntngcannngntnannnnngnnncnnnnnnnngnn
tnnnnnnnnnnncnngncnannnnnnnannnnnnnagcnnnnnnnnnnnnnnnnnnnnnnnnnnnnnncnnnggtcnnncnnc
tcagannnngnnnnnnnnnnnnnnnnnnnnnnnnnnnnnnnnnnnnnnnnnnnnnngnnnnnnnnnnnnnnnnnn
annannngttcatggaaaaagagtgccnannnnnnnnnnnnnnnnnnnnnnncnnncnca
(A) CR (112/401): 27.9%
(B)
Max score: 44.6
Total score: 44.6
Query coverage: 7%
E value: 2.7
Identity: 88%
- AB675463.1: Hokkaido virus Small gene for nucleocapsid protein, complete cds, isolate: Kitahiyama128S/2008.
- AB675478.1: Hokkaido virus Small gene for nucleocapsid protein, complete cds, isolate: ShariP5-3S/2010.
- AB675477.1: Hokkaido virus Small gene for nucleocapsid protein, complete cds, isolate: Shari6-10S/2010.
- AB675475.1: Hokkaido virus Small gene for nucleocapsid protein, partial cds, isolate: Abashiri22S/1994.
- AB675472.1: Hokkaido virus Small gene for nucleocapsid protein, partial cds, isolate: Nakagawa73S/2004.
- AB675471.1: Hokkaido virus Small gene for nucleocapsid protein, complete cds, isolate: Nakagawa49S/2004.
- AB675470.1: Hokkaido virus Small gene for nucleocapsid protein, partial cds, isolate: Nakagawa13S/2004.
- AB675468.1: Hokkaido virus Small gene for nucleocapsid protein, partial cds, isolate: Tobetsu84S/2010.
- AB675467.1: Hokkaido virus Small gene for nucleocapsid protein, partial cds, isolate: Tobetsu60S/2010 .
- AB675466.1: Hokkaido virus Small gene for nucleocapsid protein, partial cds, isolate: Tobetsu57S/2004 .
- AB675465.1: Hokkaido virus Small gene for nucleocapsid protein, complete cds, isolate: Tobetsu27S/2004.
- AB675464.1: Hokkaido virus Small gene for nucleocapsid protein, partial cds, isolate: Tobetsu89S/2000.
- AB675462.1: Hokkaido virus Small gene for nucleocapsid protein, partial cds, isolate: Kitahiyama124S/2008.
- AB675461.1: Hokkaido virus Small gene for nucleocapsid protein, partial cds, isolate: Kitahiyama96S/2008.
- AB675460.1: Hokkaido virus Small gene for nucleocapsid protein, partial cds, isolate: Kitahiyama85S/2008.
- AB675459.1: Hokkaido virus Small gene for nucleocapsid protein, partial cds, isolate: Kitahiyama77S/2008.
- AB675458.1: Hokkaido virus Small gene for nucleocapsid protein, partial cds, isolate: Kitahiyama63S/2008.
- AB675457.1: Hokkaido virus Small gene for nucleocapsid protein, partial cds, isolate: Kitahiyama57S/2008.

- AB675456.1: Hokkaido virus Small gene for nucleocapsid protein, partial cds, isolate: Kitahiyama55S/2008.
- AB675450.1: Hokkaido virus Small gene for nucleocapsid protein, complete cds, isolate: Tobetsu35S/2010 .
- AB010731.1: Puumala virus N gene for nucleocapsid protein, complete cds, isolate: Tobetsu-60Cr-93.
- AB010730.1: Puumala virus N gene for nucleocapsid protein, complete cds, isolate: Kamiiso-8Cr-95.

>seq230:Hantavirus_Puumala_2_13-02-2013_(VirIDv3r520827) Start=12 End=412
ttggagtgtatgngntagggttcacacttcctntcnnnnnnnnnnnnnnnnnnnnncnnnnaacgcgtgggagacagactgnnnnnnnnnn
nnnnnnnnnnnnnnnnnnnnnnnnnnnnnnnnnnnnnnnnnnnnnnntcaatgncatanggagaccaaagcnttnnnnnnttnctatg
cctncngcccngtcannnnnnnnannnnnnnnnnnnnnnnnnnnnnnnnnnnnnnnantagtatgtggtctttnnnnnnnnnnnnnnn
nnnnnnnnnnnnnccnnnnnnnnnnnnnnnngtcattggntttnnannnnncnnnnnncnnnnngcnnnnannnnnnnanngttca
tggaaaaagagtgccnannnnnnnnnnnnnnnnnnnnnnnnnncnnn
(A) CR (155/401): 38.6%
(B)
Max score: 55.4
Total score: 108
Query coverage: 22%
E value: 0.001
Identity: 77%
- HE801633.1: Puumala virus N and Ns genes, segment S, genomic RNA, strain Sotkamo 2009.
- X61035.1: Puumala virus genomic RNA for nucleocapsid protein.

>seq231:Hantavirus_Puumala_2_13-02-2013_(VirIDv3r520827) Start=12 End=412
nnnnnnngnnnnngnnngngttcacactnnntnncnnnnnnnnnagctttatacntgcnctcaacgcgtgggagacagactgtaaaggnaaat
aaggggacacgtataaggtttaaggatgatacatcatttgaagncatcaatggcataaggagaccaaagcatttatatgtttctatgcctactgcccag
ncnagnatgaangcagaagaactcacaccaggcagntttcgcacaatagtatgtggtcttttncccnnnnnnnnncnngnnncgtaacatcatga
gtccagttatgnnnnncntnnntnnnnnnnnncnnnntgnnggattggtctgagagaatcagagagttcatggaaaaagagtgcccattcataaa
gcnncnnnnnnngccnntcnna
(A) CR (313/401): 78.0%
(B)
Max score: 439
Total score: 439
Query coverage: 84%
E value: 4e-119
Identity: 86%
- HE801633.1: Puumala virus N and Ns genes, segment S, genomic RNA, strain Sotkamo 2009.

>seq232:Hantavirus_Puumala_2_13-02-2013_(VirIDv3r520827) Start=12 End=412
tnngngtgnatgtnnnngggttnncacnngnnnnnnnnnnnnnnnnnnnnnnnnnnncnnnnngnnnnngggacagactgtaaagn
naaatnngnnnnnnnnnnnncnntttaaggatganngntcatttgaagacatcaatggcatnnggagaccaaagcattnnnntgtttctatgcca
acnnccnnntcaactatgaangcagaagnnnnnnnnnnnnnnnnnntnncncaatagtatgnngtngnnnnnnnnnnnnnnnnnn
nnnnnnnnnncnnnagtccagttatggnggtcattnnttntnnannnnnnnnngnnnnnnnnnnnnnnnnnnnnnnnnnnnnnnnn
nnnnnnnnnnnnnannnnnnnnngnntgaannnnanccagncnnn
(A) CR (180/401): 44.9%
(B)
Max score: 118
Total score: 118
Query coverage: 24%
E value: 1e-22
Identity: 83%
- HE801633.1: Puumala virus N and Ns genes, segment S, genomic RNA, strain Sotkamo 2009.

- X61035.1: Puumala virus genomic RNA for nucleocapsid protein.

>seq233:Hantavirus_Puumala_2_13-02-2013_(VirIDv3r520827) Start=12 End=412
tnnnannnnnnnnnncnnnctttncacttnnnnnnnnnnnnnnnnnnnnnnnnnnnnnncnnnacgcgtgggagacagactgtaaagga
aaataagggnnncnggattcgatttaaggatnntgnnnnnnnnnnannnancncnnnnnnanggnganccnnnnnntnnnnnnnngnnt
nnnnnnnnnnnnnnnnnggtntnnaggccgaagnnnnnnnnnnnnnnnnnnnnnnnnannngnnnnnnntngtnnnnnnnnn
nnnnnnnnnnnnnnnnnnncnnannnnnnnnnnnnnnnnnnnngtnnncnnnnnnncnnnnngnnnnnnnnnnnnnnnnnnn
nnnnnnnnnnnnnnnnnannnnnnnnnnnnnnnnnnnnnnnnnnnnnnnnnnnanccnnn
(A) CR (110/401): 27.4%
(B)
Max score: 80.6
Total score: 80.6
Query coverage: 14%
E value: 4e-11
Identity: 89%
- KY365007.1: Puumala orthohantavirus isolate Septmoncel-Alsace_2015_Camp5 nucleoprotein gene, complete cds.
- KY364999.1: Puumala orthohantavirus isolate MontsousVaudrey-Jura_2014_NCHA14 nucleoprotein gene, complete cds.
- KY364998.1: Puumala orthohantavirus isolate ChauxdesCrotenay_Jura_2014_NCHA71 nucleoprotein gene, complete cds.
- KY364997.1: Puumala orthohantavirus isolate ChauxdesCrotenay-Jura_2014_CI11 nucleoprotein gene, complete cds.
- KT247596.2: Puumala virus isolate PUUV/Jura/Mg2/2010 segment S nucleoprotein gene, complete cds.
- KT247597.1: Puumala virus isolate PUUV/Jura/Mg214/2010 segment S nucleoprotein gene, complete cds .
- AM695638.1: Puumala virus S gene for nucleocapsid protein, strain PUU/Mignovillard/CgY02/2005, genomic RNA.

>seq234:Hantavirus_Puumala_2_13-02-2013_(VirIDv3r520827) Start=12 End=238
acagntnnnnnnnngnntataacccgncatgaannnnnnnnnnattgttgccagacaaaaacntnnnnnnnnnnnnnnnnnnnnnnagt
ggacccagatgacgttaataaanacanncngcaagccnggcaacaaacagtgtcagcantggaggacaaacnngcagactnnnnnangngg
atggcagatgctgngnncnnnnnnnaannggannnnnaannnacnga
(A) CR (146/227): 64.3%
(B)
Max score: 136
Total score: 136
Query coverage: 77%
E value: 3e-28
Identity: 72%
- HE801633.1: Puumala virus N and Ns genes, segment S, genomic RNA, strain Sotkamo 2009.
- X61035.1: Puumala virus genomic RNA for nucleocapsid protein.

>seq235:Hantavirus_Puumala_2_13-02-2013_(VirIDv3r520827) Start=12 End=412
nnnnnnnnnnnnnnnnnnnnttcnnnnntnnnnnnnnnnnnnnncnnnnnnnnntntnnncncaactnnnnnnnnnnnnnnnnnnn
gnnnnataaggggnnnnnnnnnnngnntnnnnnnnnnnnnnnnnnnnnnnnnnnnnnnnnnnnnnnnnnnncnngnnnnnnnngn
nngtgtctatgccnnnnagcccantctagcatgaaagcagaaganntcacaccnnncnnnnnntnnnncantngttgnnnnnnnnnnnnnn
nnnnnnnnnnnnnnnnnnnnnnnnnnnnnngnnnnnntnnnntgnnnnnnnnngnnnnnnnancanaggactnngnngnnnngnn
cnnnnnnnnnnnnnnnnnnnnnnnnnnnnnnnnnngnnnnnnnnnnnnnnnnncnng
(A) CR (101/401): 25.2%
(B)
Max score: 55.4
Total score: 55.4

Query coverage: 12%
E value: 0.001
Identity: 82%
- HQ697348.1: Tula virus isolate GER/109/Arv nucleocapsid protein gene, partial cds.
- FJ495098.1 : Tula virus strain TULV/Sestrze/Mag98_02 segment S nucleocapsid protein gene, partial cds.
- EU337015.1: Puumala virus isolate plasma-1 nucleocapsid protein gene, partial cds.
- EU337014.1: Puumala virus isolate saliva-1 nucleocapsid protein gene, partial cds.

(Note) Simultaneous detection of PUUV, TULV, HTNV (at lower values).

>seq236:Hantavirus_Puumala_2_13-02-2013_(VirIDv3r520827) Start=12 End=412
tnnnanngnnnnnnnnnngcttngcacttnnnnnannnnnnnnnnnnnnnnnnnnnnnnnncnnnacgcgtgggagacagactgtaaagga
aaataagggnnnnnggattcgatttaaggatgatnnnnnnnnnnnannnnnnnnnnnnnnnnnggngaccanngnntnnnnnnnnnnnt
nnnnnnnnnnncnngnctancannnaagccnnnnnnnnnnnnnnnnnnnnnnnnnnnnncnnantagtatgtggtcttttncccnnnnnnn
nnnnnnnnnnnnnnncnnnnnnnnnnnncnnnnnnnnnggnnnnnnnnnnnncnnnnngnnnnnnnnnnnnnnnnnnnnnn
nnnnnnnnnnnnnnnnnnnnnnnnnnnnnnnnnnnnnnnnnnnnnnnnnnacccnnn
(A) CR (124/401): 30.9%
(B)
Max score: 80.6
Total score: 80.6
Query coverage: 14%
E value: 4e-11
Identity: 88%
- KY365007.1: Puumala orthohantavirus isolate Septmoncel-Alsace_2015_Camp5 nucleoprotein gene, complete cds.
- KY364999.1: Puumala orthohantavirus isolate MontsousVaudrey-Jura_2014_NCHA14 nucleoprotein gene, complete cds.
- KY364998.1: Puumala orthohantavirus isolate ChauxdesCrotenay_Jura_2014_NCHA71 nucleoprotein gene, complete cds.
- KY364997.1: Puumala orthohantavirus isolate ChauxdesCrotenay-Jura_2014_CI11 nucleoprotein gene, complete cds.
- KT247596.2: Puumala virus isolate PUUV/Jura/Mg2/2010 segment S nucleoprotein gene, complete cds.
- KT247597.1: Puumala virus isolate PUUV/Jura/Mg214/2010 segment S nucleoprotein gene, complete cds .
- AM695638.1: Puumala virus S gene for nucleocapsid protein, strain PUU/Mignovillard/CgY02/2005, genomic RNA.

>seq237:Hantavirus_Puumala_2_13-02-2013_(VirIDv3r520827) Start=12 End=412
tggagtgtatgtgntagggttnacacttcctatcnnnnnnnnnnnnnngnnnnnncnnncaacgcgtgggagacagactgcannnnnnnnn
nnnnnnnnnnnngnnnnnnnnnnnnnnnnnnnnnnnnnnnncnnnnnnnaatggcatanggagaccaaagcntttnnntntttctatgcc
tnctgcccngtcaannnnnnnannnnnnnnnnnnnnnnnnnnnnnnnnnnnnnnnantagtatgtggtctttnnnncnnnnnnnnnnn
nnnnnnnnnnnccnnnnnnnnnnnnnnnngtcattggntttnnnnnnnnnnnnnnncnnnngncnnnnannnnnnnnnnngttcatg
gaaaaagagtnccnnnnnnnnnnnnnnncnnnnnnnnncnnncncnn
(A) CR (164/401): 40.9%
(B)
Max score: 71.6
Total score: 127
Query coverage: 22%
E value: 2e-08
Identity: 85%
- HE801633.1: Puumala virus N and Ns genes, segment S, genomic RNA, strain Sotkamo 2009.
- X61035.1: Puumala virus genomic RNA for nucleocapsid protein.

>seq238:Hantavirus_Puumala_2_13-02-2013_(VirIDv3r520827) Start=12 End=290
attgganngnnnnnnnnnngcttngcacttncnnncnnnnnnnnnnnacnnnntnnncnnacnacgcgngggagacagactgnnnnnn
nnnnnnnnnnnnnnnnnnnnnnnnnnnnnnnnnnnnnnnnannnnnannnnnnnnnnnncnnannnnnnnnnnnnanntcnnnnn
nngnntnngncnnnngcccngtnannnnnnnnannnnnnnnnnnnnnnnnnnnnnnnnnnnnnnnnnnantannatgtggtctttnnnnn
nnnnnnnnnnnnnnnnnnnn

(A) CR (76/279): 27.2%
(B)
No BLASTN identification

>seq239:Hantavirus_Puumala_2_13-02-2013_(VirIDv3r520827) Start=12 End=290
attggagtgtatgtgntagggttcacacttcctatcnnnnnnnnnnnnnnnnnnnnnncnnncnacgcgtgggagacagactgcannnnnnnn
nnnnnnnnnnnnnnnnnnnnnnnnnnnnnnnnnnnnnnnnncntncnncaatggcanannnnancncccgngnnnnnnngtttctat
gcctnctgcccngtcannnnnnnnannnnnnnnnnnnnnnnnnnnnnnnnnnnncnnnnannagtatgtggtctttnnnncnnnnnnnnn
nnnnnnnnnnn

(A) CR (117/279): 41.9%
(B)
Max score: 62.6
Total score: 62.6
Query coverage: 12%
E value: 7e-06
Identity: 97%

- HE801633.1: Puumala virus N and Ns genes, segment S, genomic RNA, strain Sotkamo 2009.
- X61035.1: Puumala virus genomic RNA for nucleocapsid protein.

>seq240:Hantavirus_Puumala_2_13-02-2013_(VirIDv3r520827) Start=12 End=290
attggagtgtatgtgntagggttnacacttcctatcnnnnnnnnnnnnanngtntntgctgacaacgngngggagncagncnnnnnnnnnnn
nnnnnnncnnnnnnnnnngnnnnngnnnnnnnnnnnnnnnnnnannnnncaangncataaggagaccaaagcnttnnnnnnnttnctat
gcctacngcccngtcaannnnnnnnannnnnnnnnnnnnnnnnnnnnnnnnnnnnnnnnnantagtatgtggtctttttcccnnnnnnnnncn
nnnnnnnnnn

(A) CR (130/279): 46.6%
(B)
Max score: 59.0
Total score: 117
Query coverage: 32%
E value: 8e-05
Identity: 94%

- HE801633.1: Puumala virus N and Ns genes, segment S, genomic RNA, strain Sotkamo 2009.
- X61035.1: Puumala virus genomic RNA for nucleocapsid protein.

>seq241:Hantavirus_Puumala_2_13-02-2013_(VirIDv3r520827) Start=12 End=290
attggagtgtatgngnnnggnttngcacctnnnnnnnnnnnnnnnnnnnngnnnnnncnnncaacgngngggagncagnnnnnnnnn
nnnnnnnnnnnncnnnncnnnnnnnnnnnnnnnnnnnnnnnnnnnnnnncntncnncnatggcananggnnnncannnnnnnnannnn
ncnnctatgcctacnnccncgnnnngnannnnnncnnnnncnnnnnnnnnnnnnnnnnnnnnnnnnnnnngtatgtggtctttnnnnnn
nnnnnnnnnnnnnnnnnnn

(A) CR (90/279): 32.2%
(B)
No BLASTN identification

>seq242:Hantavirus_Puumala_2_13-02-2013_(VirIDv3r520827) Start=12 End=290
nnnnnnnnnnnnnnnnnngngttnncncntnnnnncnnnnnnnnnnnnnnnnnnnnnnnncnnnacgcgtgggagacagnctgtaaa
ggaaaataaggggacncgnatnnnnntnnngnnnnnnnnnnnnnnnnnannnnnnnnnnnnnnnnnnngaccaaagcatnnannnn
nnnnnnnnnaannngcccnntcaaccatgaaagcagnnnnnnnnnnnnnnnnnnnnnnnnnnnnnannagtatgtggtcntnnnnnnn
nnnnnnnnnnnnnnnnnnn

(A) CR (100/279): 35.8%
(B)

Max score: 69.8
Total score: 69.8
Query coverage: 15%
E value: 5e-08
Identity: 93%

- HE801633.1: Puumala virus N and Ns genes, segment S, genomic RNA, strain Sotkamo 2009.
- AJ238790.1: Puumala virus RNA for nucleocapsid protein, strain Gomselga.
- X61035.1: Puumala virus genomic RNA for nucleocapsid protein.

>seq243:Hantavirus_Puumala_2_13-02-2013_(VirIDv3r520827) Start=12 End=290
attggagngnnnnngnnnngctttgcacttnnnnncnnnnnnnnnnnnnnnnnnnnnnncnnnncnngngggaggcaaacnnnnnnn
nnnnnnnnnnnnnnnnngnnnnnnntttangnnnnnnacannatnngaagncnnnnnnnnnnnnnnnnnannnnnnnnnnnnnncn
ntnntntgcctactgcccagtcaactannnnannngnnnnnnnnnnnnnnnnnnnnnnnnnnnnnnnnnnnangtggtctttttcccnntnn
nnannngnntnnnnnc
(A) CR (98/279): 35.1%
(B)
No BLASTN identification

>seq244:Hantavirus_Puumala_2_13-02-2013_(VirIDv3r520827) Start=12 End=412
nnnnnnnnnnnnnnnnnnnnnnnnnnggnnnnnnnnnnnnnnnnnnnnnnnnnnnnnnnnnnnnnnnnnnnnnnnnnnnn
nnnnnnnnnnnnnnnnnnncngnnncgatttaaggatnnnannnnnnnnnnnnnnnnncnnnnnnnnnnncnnnnnnnannnnnnn
nnnnngnctatgccnannnnnnnnnnnnncnnnnnnnnncnnannncnnnnnnnnnnnnnnnnnnnnnnnnnnnntnnnnnnnnn
nnnnnnnnnnnnnnnnnnnnnnnnncngnnnaagtcctgtcatgggtgtgattggattnnnnnnnnncnnnnnnnnnnnnnnnnn
nnnnnnnnnnnnnnnnnnncnnnnnnncnnnnnnnnnnnnnnnnnnnnnnnnnnnnnnnnnn
(A) CR (66/401): 16.4%
(B)
Max score: 50.0
Total score: 50.0
Query coverage: 6%
E value: 0.064
Identity: 100%

- KU139579.1: Tula virus isolate 10_1188_Magr nucleocapsid protein gene, partial cds.
- KU139578.1: Tula virus isolate 10_0932_Marv nucleocapsid protein gene, partial cds.
- KU139577.1: Tula virus isolate 10_0908_Marv nucleocapsid protein gene, partial cds.
- KU139576.1: Tula virus isolate 10_0905_Marv nucleocapsid protein gene, partial cds.
- KU139575.1: Tula virus isolate 12_1121_Marv nucleocapsid protein gene, partial cds.
- KU139574.1: Tula virus isolate 12_1068_Marv nucleocapsid protein gene, partial cds.
- KU139573.1: Tula virus isolate 10_1533_Marv nucleocapsid protein gene, partial cds.
- KU139572.1: Tula virus isolate 12_526_Magr nucleocapsid protein gene, partial cds.
- KU139571.1: Tula virus isolate 12_0492_Magr nucleocapsid protein gene, partial cds.
- KU139570.1: Tula virus isolate 08_0545_Marv nucleocapsid protein gene, partial cds.
- KU139569.1: Tula virus isolate 08_0362_Marv nucleocapsid protein gene, partial cds.
- KU139568.1: Tula virus isolate 08_0356_Marv nucleocapsid protein gene, partial cds.
- KU139567.1: Tula virus isolate 08_0352_Marv nucleocapsid protein gene, partial cds.
- KU139566.1: Tula virus isolate 08_0350_Marv nucleocapsid protein gene, partial cds.
- KU139565.1: Tula virus isolate 08_0538_Marv nucleocapsid protein gene, partial cds.
- KU139564.1: Tula virus isolate 08_0534_Marv nucleocapsid protein gene, partial cds.
- KU139548.1: Tula virus isolate 10_1625_Marv nucleocapsid protein gene, partial cds.
- KU139546.1: Tula virus isolate 07_0081_Magr nucleocapsid protein gene, partial cds.
- KU139545.1: Tula virus isolate 08_0894_Marv nucleocapsid protein gene, partial cds.
- KU139544.1: Tula virus isolate 08_0849_Marv nucleocapsid protein gene, partial cds.
- KU139543.1: Tula virus isolate 08_0848_Marv nucleocapsid protein gene, partial cds.
- KU139542.1: Tula virus isolate 08_0802_Marv nucleocapsid protein gene, partial cds.

- KU139541.1: Tula virus isolate 08_0789_Marv nucleocapsid protein gene, partial cd
- KU139540.1: Tula virus isolate 11_1433_Marv nucleocapsid protein gene, partial cds.
- KU139539.1: Tula virus isolate 11_1429_Marv nucleocapsid protein gene, partial cds.
- KU139536.1: Tula virus isolate 11_1373_Marv nucleocapsid protein gene, partial cds.
- HQ697347.1: Tula virus isolate GER/09/2155/Arv nucleocapsid protein gene, partial cds.
- GU300137.1: Tula virus strain dpz06-29 nucleocapsid protein gene, partial cds.
- GU300135.1: Tula virus strain dpz06-1 nucleocapsid protein gene, partial cds.
- EU439952.1: Tula virus strain Sennickerode Sen05/222 nucleocapsid protein gene, partial cds.
- EU439951.1: Tula virus strain Sennickerode Sen05/205 nucleocapsid protein gene, complete cds.
- EU439950.1: Tula virus strain Sennickerode Sen05/204 nucleocapsid protein gene, complete cds.
- EU439949.1: Tula virus strain Sennickerode Sen05/175 nucleocapsid protein gene, complete cds.
- EU439948.1: Tula virus strain Sennickerode Sen05/174 nucleocapsid protein gene, partial cds.
- EU439946.1: Tula virus strain Sennickerode Sen05/121 nucleocapsid protein gene, partial cds.
- AJ223600.1: Tula virus genomic RNA for nucleocapsid protein, (strain Tula/Koziky/5247Ma/94).
- Y13984.1: Tula virus partial genomic RNA for nucleocapsid protein, strain (Tula/D540/Ma/95).
- U95304.1: Tula virus O20 nucleocapsid protein gene, partial cds.
- U95303.1: Tula virus O52 nucleocapsid protein gene, partial cds.
- U95302.1: Tula virus O24 nucleocapsid protein gene, partial cds.
- Z69991.1: Tula virus genomic RNA for nucleocapsid protein, strain (Tula/Moravia/5302v/95).
- Z49915.1: Tula virus segment S, strain Tula/Moravia/5302Ma/94, genomic RNA.
- Z48741.1: Tula virus segment S, strain Tula/Moravia/5294Ma/94, genomic RNA.
- Z48574.1: Tula virus segment S, strain Tula/Moravia/5293Ma/94, genomic RNA.
- Z48573.1: Tula virus segment S, strain Tula/Moravia/5286Ma/94, genomic RNA.

>seq245:Hantavirus_Puumala_2_13-02-2013_(VirIDv3r520827) Start=12 End=412
nnnnnnnnnnnnnnnnnannncgnnnnnnnnnnnnnnnnnnnnnnnnnngnnnnnnnnnnnnnnnnnnnngnnnnnnngn
nnnnnnnnnnnnnnnnnnnnnattcgatttaaggatganagctcanannnnnnnnnnnnnnnnnnnnnnnnnngnnnnnnnnn
nnnnnnnnnnnnnannnnnnnngnnanncnnnnnggcagaagagattacnnctgnnannnnnnnnnnnnnnnnnnnnnnnnnnn
nnnnnnnnnannnnnnnnnnnnnnnnnnnnnnnnnnannnatggnnnnnnnnnnannnnnnnnnnnnnnnncnnnnnnnnnnn
nnnnnnnnncnngnnnnnnnnnntngnnnnnnnnnnnnnnnnnnnnccnnnnnnnnnnnnnnnnnnn
(A) CR (68/401): 16.9%
(B)
No BLASTN identification.

>seq246:Hantavirus_Puumala_2_13-02-2013_(VirIDv3r520827) Start=12 End=412
nnnnnnnnnnnnnnnnnnnnnnnncgncccgnnncnnngnnnnnnnnnngnnnnnnnnnnnnnnnnnnnnnngcnnnnnnnn
nnnnnnnnnnnnnnnnngnnnnnnnnnnttaaggatgatagctcgttcgaggatnntnncggtntccggaaaccaaaacannnnnnnnn
nnnnnnannnnngnacagncangcntnncnnnnnnagngcnnnnnnnnnnnnnnnnnntnnnnnnnnnnnnnnnnnnnnnnn
nnnnnnnnnnnnnnnnnnnnnnnnnnnnnnnncctgtcatgggtgtaannnnantnccngngnnnnnnnncgnnnnnnnnnnnnnnn
nnnnnnnnnnnnnnnnnnnnngnnnnnnnnnnnnnnnncnnnnnnnnnnngcnnnnnnn
(A) CR (104/401): 25.9%
(B)
Max score: 77.0
Total score: 77.0
Query coverage: 12%
E value: 5e-10
Identity: 90%
- KX601186.1: Hantaan hantavirus isolate SNHRC-HTN-128 nucleocapsid protein gene, partial cds.
- KU207190.1: Hantaan virus isolate Aa04-722 segment S nucleocapsid protein gene, complete cds.
- KP970573.1: Hantaan virus isolate JS2 segment S, complete sequence.
- KP970572.1: Hantaan virus isolate JS1 segment S, complete sequence.
- EF208934.1: Hantavirus CJAa1109 nucleocapsid protein gene, partial cds.
- EF208933.1: Hantavirus CJAa716 nonfunctional nucleocapsid protein gene, partial sequence.

- EF208932.1: Hantavirus CJAa594 nucleocapsid protein gene, partial cds.
- AF288659.1: Hantavirus S85-46 nucleocapsid protein mRNA, complete cds.
- AF288294.1: Hantavirus LR1 segment S S protein mRNA, complete cds.
- U37768.1: Hantaan virus S segment nucleocapsid protein mRNA, complete cds.
- D25533.1: Hantaan virus gene for nucleocapsid protein, complete cds.
- D25530.1: Hantaan virus gene for nucleocapsid protein, complete cds.
- M14626.1: Hantaan virus S segment encoding nucleocapsid protein.

>seq247:Hantavirus_Puumala_2_13-02-2013_(VirIDv3r520827) Start=12 End=412
nnnnnnnnnnnnnnnnnnnnnnnncnnnnnnnnnnnnnnnnnnnnnnnngnatatgnnntcaacnngngggnggcnnncnnncn
nnnnngnnnnnnnnnnnnnnnnnnnnnnntnaggatgacanntnggnnnnnnnnnnnnnnnnnnnncggaaaccaaaacnnnnnnn
nnnnnnnnnnnctacngcncagtctnccanganggccgaagagattannnntnnnnnnnnnnnnnnannnnnnnnnnnncnnnnngn
ncnnnnnnnnnnnnnnnnncnnnnnnnnnnggagnnatnngtgtaantnnannncnnnnnnnnnnnnnngnnnnngnnnnnnnn
nnnnnnnnncnnnntgntnnnnnnnnnnnnnnnnnnnnnnnnnnnnnnnnnnnnnnn
(A) CR (106/401): 26.4%
(B)
No BLASTN identification

>seq248:Hantavirus_Puumala_2_13-02-2013_(VirIDv3r520827) Start=12 End=412
nnnnnnnnnnnnnnnnnnnnnnnnnnnnnnnnnnnnnnnnnnnnnnnnnnnnngncaacanggggggagncnnnntnnn
nnnnnnnnnnnnnngnnnnnnnnnnnnnnnnnngatgatacatcannnnnnnncacnncntncnnnnnnnnnnnnnnnnnnnn
nnnnnnnntnnnnnnnnnnnnnngnnannnnnnnnnnnnnnnnnnngcnnnnnnntnnnnnnnnnncnnnannnnnnnngngggctct
acnnnnntnnnnnnnnnnnccnncnnatggncngnnnnnnnnnnnnnngnngnnncnnnnnnnnnnnnnnnnnnnnnnnnnn
nnnnnnnnnnnnnnnnnnnnnnnnnnnnnnnnnnnnnnnnnnnnnnnnnnnnnnnnnnnnnnnncnnnn
(A) CR (66/401): 16.4%
(B)
No BLASTN identification

>seq249:Hantavirus_Puumala_2_13-02-2013_(VirIDv3r520827) Start=12 End=412
tcatcgnctatcttacatcctttgtcgtcccgatacttctgaaagctctgnatatgttgacaacangnnnnaggcaaactaccaaggataataaaggga
cccggattcgatttaaggatgatagctcgttcgaggatgttaacggtatccggnaaccaaaacatctttacgtgtccttgccaaangcncngncaagc
atgaaggcagaagagattacacctggtagatatagaacagcagtctgtgggctctaccctgcacagattnnggcncggcagnngancagtccagt
tatgagtgtaattggttttctagcattagcaaaggactggagtgatcgnatcgaacaatggttaattgaaccttgcaagcttcttccagatacagcagca
gttagcctc
(A) CR (382/401): 95.3%
(B)
Max score: 655
Total score: 655
Query coverage: 100%
E value: 0.0
Identity: 95%
- KP970573.1: Hantaan virus isolate JS2 segment S, complete sequence.
- KP970572.1: Hantaan virus isolate JS1 segment S, complete sequence.
- AF288659.1: Hantavirus S85-46 nucleocapsid protein mRNA, complete cds.
- U37768.1: Hantaan virus S segment nucleocapsid protein mRNA, complete cds.
- D25533.1: Hantaan virus gene for nucleocapsid protein, complete cds.
- D25530.1: Hantaan virus gene for nucleocapsid protein, complete cds.
- M14626.1: Hantaan virus S segment encoding nucleocapsid protein.

>seq250:Hantavirus_Puumala_2_13-02-2013_(VirIDv3r520827) Start=12 End=412
nnnnnnnnnnnnnnnnntnctttgtcgtcccgnnnnnnnnnnnnnnnnnnnnnnnnnnnnnnnnactagnnggnnncnannnnnnnng
nataatnnnnggacccggnttcgatttaaggatganagcnnnnncnaggatgttaacggtnnccggaaaccaaagcnnttatatgtttnnnnnn
nnaatgcacagtcaagcatgaaggcagaagagcttnnnnnnnncnnnnnnnnnnnnnngnnctgtgggctctaccctgcacagattaaggc

ncngcngatgnncngtcnngnnnnnggtgngnnnnnnnnnnnnnngnnnnnnnnnnnnnnnnnnnnnnnnnnnnnnnnncnnnn
nntnnnnnnnntncnnnnntnntccagatacagcagcagntnnnnnn

(A) CR (201/401): 50.1%

(B)

Max score: 132
Total score: 132
Query coverage: 50%
E value: 7e-27
Identity: 69%

- KP970573.1: Hantaan virus isolate JS2 segment S, complete sequence .
- KP970572.1: Hantaan virus isolate JS1 segment S, complete sequence.
- AF288659.1: Hantavirus S85-46 nucleocapsid protein mRNA, complete cds.
- U37768.1: Hantaan virus S segment nucleocapsid protein mRNA, complete cds.
- D25533.1: Hantaan virus gene for nucleocapsid protein, complete cds.
- D25530.1: Hantaan virus gene for nucleocapsid protein, complete cds.
- M14626.1: Hantaan virus S segment encoding nucleocapsid protein.

>seq251:Hantavirus_Puumala_2_13-02-2013_(VirIDv3r520827) Start=12 End=412
nnnnnnnnnnnnnnnnnnnnnnnnncnncncnntacttctgaaagctctntnnnnnnnnnnnnnnnnnnngnagncaaacnnnnnnnn
nnnnnnnnnnnnnannnnncnnnnttaaggntgnnnncnnnnnnnnnnncnnnnnnnntnnccggaaaccaaaacatctntnnnngn
nnnnnnnannngnanngtcaagcatgaaggcagaagagattacacctggtnnnnnnnnnnnnnnggnntgtgggctcttccctgcacagatt
annnngnnnncnnnnnnnnnnnnnnnntatgagtgtaattggttttnnnnngnnnnngnnggnctggannġntnnnncnnnnnnnnnnn
nnnnnnnnnnnnnccngnntnttccagatacagcagcagttngccnn

(A) CR (185/401): 46.1%

(B)

Max score: 80.6
Total score: 80.6
Query coverage: 29%
E value: 4e-11
Identity: 69%

- KP970573.1: Hantaan virus isolate JS2 segment S, complete sequence.
- KP970572.1: Hantaan virus isolate JS1 segment S, complete sequence.
- EF208934.1: Hantavirus CJAa1109 nucleocapsid protein gene, partial cds.
- EF208932.1: Hantavirus CJAa594 nucleocapsid protein gene, partial cds.
- AF288659.1: Hantavirus S85-46 nucleocapsid protein mRNA, complete cds.
- AF288294.1: Hantavirus LR1 segment S S protein mRNA, complete cds.
- U37768.1: Hantaan virus S segment nucleocapsid protein mRNA, complete cds.
- D25533.1: Hantaan virus gene for nucleocapsid protein, complete cds.
- D25530.1: Hantaan virus gene for nucleocapsid protein, complete cds.
- M14626.1: Hantaan virus S segment encoding nucleocapsid protein.

>seq252:Hantavirus_Puumala_2_13-02-2013_(VirIDv3r520827) Start=12 End=412
ncnncnnctatcttacatcctttgtcntcccgatacttctgaaagctctgtatatgttgncaacaagggggaggcannnnancaagnnnnnnnnnn
nnccccggattcgatttaaggatganagcncgntnncnnncnnnnnnacnngnngnaaaccaaaacnnntgtncgtgtccttgccaaatgcac
agtcaaccatgaaggcagaagagattacacctggtagatatagaacagcngncnnnnnnnnncnncnnngcncagattaagncncgnngnn
nnnnnnncctgtcatgggtgtgngnnnnnnnnnnnngnnnnnnnnnnncnnnnnnngnnnnnnnnnnnnncnnnnnntnganccttt
gcaagcttcttccagatacagcagcagttagcctc

(A) CR (272/401): 67.8%

(B)

Max score: 230
Total score: 303
Query coverage: 68%
E value: 3e-56
Identity: 79%

- KP970573.1: Hantaan virus isolate JS2 segment S, complete sequence.
- KP970572.1: Hantaan virus isolate JS1 segment S, complete sequence .
- AF288659.1: Hantavirus S85-46 nucleocapsid protein mRNA, complete cds.
- U37768.1: Hantaan virus S segment nucleocapsid protein mRNA, complete cds.
- D25533.1: Hantaan virus gene for nucleocapsid protein, complete cds.
- D25530.1: Hantaan virus gene for nucleocapsid protein, complete cds.
- M14626.1: Hantaan virus S segment encoding nucleocapsid protein.

>seq253:Hantavirus_Puumala_2_13-02-2013_(VirIDv3r520827) Start=12 End=412
nnnnnnnnnnnnntnnnnnnnnnnncgtcccgatacttctgaaagctctgtatatgttgncaacaagggggnnncnnnnnaccaaggataat
anagggacccggattcgatttnnggatgntagctcgntcgaggnnntnaanngcnnnnnnnnannnnnnnnnnngnnngnnnnnnnnnc
nnatgcacagtcaagcatgaaggcagaagagattacacctggtagatatagancnnnggnnnnnnnnnnnntnccnncncagattaangcnc
ngnnnnnnnnnnnnccngtcatgggtgtgattggnttnnnnnnnnnnnnnnnnncnnnnnnngnnnnnnnnnnnnnnnnnntnn
ntnntnncnagcntcttccagntacagcancagttagcctc
(A) CR (231/401): 57.6%
(B)
Max score: 176
Total score: 224
Query coverage: 59%
E value: 6e-40
Identity: 74%
- KP970573.1: Hantaan virus isolate JS2 segment S, complete sequence
- KP970572.1: Hantaan virus isolate JS1 segment S, complete sequence.
- AF288659.1: Hantavirus S85-46 nucleocapsid protein mRNA, complete cds.
- AF288294.1: Hantavirus LR1 segment S S protein mRNA, complete cds.
- U37768.1: Hantaan virus S segment nucleocapsid protein mRNA, complete cds.
- D25533.1: Hantaan virus gene for nucleocapsid protein, complete cds.
- D25530.1: Hantaan virus gene for nucleocapsid protein, complete cds.
- M14626.1: Hantaan virus S segment encoding nucleocapsid protein.

>seq254:Hantavirus_Puumala_2_13-02-2013_(VirIDv3r520827) Start=12 End=412
nnnnnnnnnnnnnnnnnnncnnnnncgtcccgatacttctgnnannnngnnnnnnnnnnnnnnncntnnnnncnnnnnnnnnn
nnnnnnnnnggacccggattcgatttaaggatgacagctcnnncnaggatgttaacgntnncnnnnnnnnnnnnnanttgtncnngtcctt
gccaaangcacngtcaagcatgaaggcagaagagattacnccnggnagnnttnnngnagcngnctgnnnnnncntcnnnnnannnnnn
nnnnnnnnnnnnnnngnnnnnnnnnntgggtgtgattnnnnnnnnnnngnngnngnnnnncnnnnnnnntcgnnncnnnnnann
nnnnntnnnnccnnncnnnnnnnnnntnnntnnagcagcagttngnnnn
(A) CR (175/401): 43.6%
(B)
Max score: 116
Total score: 116
Query coverage: 32%
E value: 5e-22
Identity: 75%
- KP970573.1: Hantaan virus isolate JS2 segment S, complete sequence.
- KP970572.1: Hantaan virus isolate JS1 segment S, complete sequence.
- EF208934.1: Hantavirus CJAa1109 nucleocapsid protein gene, partial cds.
- AF288659.1: Hantavirus S85-46 nucleocapsid protein mRNA, complete cds.
- U37768.1: Hantaan virus S segment nucleocapsid protein mRNA, complete cds.
- D25533.1: Hantaan virus gene for nucleocapsid protein, complete cds.
- D25530.1: Hantaan virus gene for nucleocapsid protein, complete cds.
- M14626.1: Hantaan virus S segment encoding nucleocapsid protein.

>seq255:Hantavirus_Puumala_2_13-02-2013_(VirIDv3r520827) Start=12 End=412

nnnnnnnnnnnnnnnnnnnnnnnnnncnngnnnnnntnnnnnnnnnnnntnnnnnnnnnnnnnnncnnnnnnccngcnnacttcnnn
nnnnnncangnnnnnnnnnnnnnnnnnntnnggatgacagctcatnnnnanncnnnnnnnnnnnnnnnnnnnnnggaacatctgtatgtg
tntatgnnnnnngcncannnnnnnnnnnnnggnagannagantacacnnnnnnnnnntnnnnnnnnnnnnnnnnnnntcnncnnngc
acagattannnnnnnnnngnnngtnannnnnnnnnnngnnnnnannnnnnnnnnnnngnnnnngnnnnnncnnnnnnnnnnnn
nnnnnnnnnnnnnnnnnnnnnnnnnngcnnnnnnnnnnnnnnnnnnnnnnnnnnntnnn
(A) CR (98/401): 24.4%
(B)
No BLASTN identification.

>seq256:Hantavirus_Puumala_2_13-02-2013_(VirIDv3r520827) Start=12 End=412
nnnncnnnnnnnnnnnnnnnnnnnncnnnnnnnnnnnnnnnncnnnnnnnnnnnnnnnnnnnnnanngggnggcannnnnnn
nnnnnnnnnnnnnnngnnnnncnnnnnttangnatgnnnncnnnnnnnnnnnnnnnnnnnnnnnnnnnnnaccaaancannngn
acgnnnntnnnncnnnngnacagtcaagcatgaaggcagaagagattacanntnncnnnnnnnnnnnntnanccnnnnntntntnnnnn
nnnnnnnnnngnnnnnnnnnnnnnannnnnanncnnngnnnnannnnnngnnnnnnnnnagcaaaggactggagtgannnn
nnnnnnnnnnnngnnncnnnnnnnnnnnnnnnnnnnnnnnnnnnnnnnnnnnnnnnnnnn
(A) CR (104/401): 25.9%
(B)
Max score: 57.2
Total score: 57.2
Query coverage: 7%
E value: 4e-04
Identity: 100%

- MF141838.1: Hantaan orthohantavirus isolate AYW118/2015 nucleocapsid protein gene, partial cds.
- MF141833.1: Hantaan orthohantavirus isolate AYW76/2014 nucleocapsid protein gene, partial cds.
- MF141832.1: Hantaan orthohantavirus isolate AWY69/2014 nucleocapsid protein gene, partial cds.
- MF141831.1: Hantaan orthohantavirus isolate AWY68/2014 nucleocapsid protein gene, partial cds.
- MF141830.1: Hantaan orthohantavirus isolate AWY66/2014 nucleocapsid protein gene, partial cds.
- MF141829.1: Hantaan orthohantavirus isolate SG40/2012 nucleocapsid protein gene, partial cds.
- KY807172.1: Hantaan orthohantavirus isolate GAN19/2011 nucleocapsid protein gene, complete cds.
- KY807168.1: Hantaan orthohantavirus isolate GAN13/2011 nucleocapsid protein gene, complete cds.
- KY807167.1: Hantaan orthohantavirus isolate GAN21/2011 nucleocapsid protein gene, complete cds.
- KP970573.1: Hantaan virus isolate JS2 segment S, complete sequence.
- KP970572.1: Hantaan virus isolate JS1 segment S, complete sequence .
- JQ665908.1: Hantaan virus strain WuhanHu16 nucleocapsid gene, partial cds.
- EF208934.1: Hantavirus CJAa1109 nucleocapsid protein gene, partial cds.
- EF208932.1: Hantavirus CJAa594 nucleocapsid protein gene, partial cds.
- DQ658415.1: Hantaan virus strain N8 nucleocapsid protein gene, complete cds.
- AF288659.1: Hantavirus S85-46 nucleocapsid protein mRNA, complete cds.
- AF288294.1: Hantavirus LR1 segment S S protein mRNA, complete cds.
- U71282.1: Hantaan virus nucleocapsid protein gene, partial cds.
- U37768.1: Hantaan virus S segment nucleocapsid protein mRNA, complete cds.
- D25533.1: Hantaan virus gene for nucleocapsid protein, complete cds.
- D25530.1: Hantaan virus gene for nucleocapsid protein, complete cds.
- M14626.1: Hantaan virus S segment encoding nucleocapsid protein.

>seq257:Hantavirus_Puumala_2_13-02-2013_(VirIDv3r520827) Start=12 End=412
ncnnnnnnnnnnnnnnnnnnnnnnnncnngnnnnnnnnnnnnnnnnnnnnnnnnnnnnnnnnnnnnnnnnncnngcagacttcan
nnnnngnnnnnnnnnnnnnnnnnnnnnnnntnnggatgacagctcatnnnnanncnnnnnnnnnnnnnnnnnnnnnnnnnnntctgtatg
tgtctatgccnngnnnnnnancnnncatgaaggcagaagagattacnnnnnncnnnnntnnngnnnnnnncnnnnnnnncnncccnnc
acagattannnncnnnnnnntnnnnnnngggnnnnnngnnnnnannnnnnnnnnnnnngnnnnnggnnngnnnnnnnnnnnnnn
nnnnnnnnnannnnnnnnnnnnnnnnnnnnnnnnnnnnnncnnnnnnnnnnnnnnnnnnn
(A) CR (104/401): 25.9%
(B)
No BLASTN identification.

>seq258:Hantavirus_Puumala_2_13-02-2013_(VirIDv3r520827) Start=12 End=412
ncnnnnnnnnnnnnnnnancatncgncnngnnnnnntnntngnnnnnntnnnnnnnnnngnnnncnannnnnnngcagacttcannn
nnnnncanggnnnnnnnnnnnnnnnnntnnggatgacagctcatnnnnanncnnnnnnnnnnnnnnnnnnnnngnnacatctgtatgtgt
ctatgcnnngnnnnnnnnnnnncnnnnnggcagaagagnntnnnnnnnncnganntnnngnnnnnnncnnnnntnncnncccnnca
nagattnnnnnnnnnnnnnnggtaannncnnnnnnngnnnnnannnnnnnnnnnnnnnnnnnnnnngnnnnnnnnnnnnnnnnnn
nnnnnnnnnnnnnnnnnnnnnnnnnnnnnnnnnnngnnnnnnnnnnnntnnnnnnnnnnn
(A) CR (110/401): 27.4%
(B)
No BLASTN identification.

>seq259:Hantavirus_Puumala_2_13-02-2013_(VirIDv3r520827) Start=12 End=412
nnnnnnnnnnnnnnnnnnnnnnnnnnnnnnnnnnnnnnnnnnnnnnnnnnnnnnnnnnnnnnnnnnnnnnnnnnnngnnnnnnn
cnnnnnngnnnnnnnnnnnnnnnnnnnnnnnnnnnnnnnnnnnnnnnnnnnnnnnnnnnnnnnnnngnnnnnnnnnnnnnnn
nnnnnnnnnnnnnnnnnnnnatgcacagtcaancannnnnnnnnnnnncnnngnnannnnnnnnnnnnnnnnnnnnnngnnnnnnn
nnnnnnnnnnnnnnnnnnnnnnnnnnnnnnnnnnnnnnnnnnnnnnnnncnnnnnnnnnnnnnnnnnnnnnnnnnnnnnnnnnnn
nnnnnnnnnnnnnnnnnnnnnnnnnnnnnnnnnnnnnnnnnnnnnnnnngnnnnnnncnnnnnnnnnnnnnnnn
(A) CR (25/401): 6.2%
(B)
No BLASTN identification.

>seq260:Hantavirus_Puumala_2_13-02-2013_(VirIDv3r520827) Start=12 End=412
nnnnnnnnnnnnnnnnnnnnnnnnnnnnnnnnnnnnnnnnnnnnnnnnnnnnnnnnnnnnnnnnnnnnnnnngnnncnnnnnnc
nnnnnnnnnnnnnnnnnnnnnnnnnnnnnnnnnnnnnnnnnnnnnnnnnnnnnnnnnnnnnnnnnnnnnnnnnnnnnnnnnnnnnn
nnnnnnnnnnnnnnnnnnnnntnnnnnnnnnnnnngnnnncagaagaactcncacnnnncnnnnnnnnnnnnannnnnnnnnnnn
cnnnnnnnnnnnnnnnnnnnntnnnnnnnnnnnnnnnnnnntngagncnnnnnnnnnnnnnnnnnnnnnnnnnnnnnnnnnnnnnn
nnnnnnnnnnnnnnnnnnnnnnnnnnnnnnnnnnnnnnnnnnnncnnnnnnnnnnnnnnnnnnnnnnnnnnnnn
(A) CR (29/401): 7.2%
(B)
No BLASTN identification.

>seq261:Hantavirus_Puumala_2_13-02-2013_(VirIDv3r520827) Start=12 End=412
nnnnnnnnnnnnnnnnnnnnnannnnnnnntnnnnnnnnnnnnnnnnnnnnnnntnnnnnctgtcaactagagggaggcaaacnnnnnnn
nnnnnnnnnnnnnnnccannnnnnnnnnnnnnnnnnnnnnnnnnnnnnnnnnnnnnnnnnnnncnntggcattagacgcnggnnnnnnnngnan
gtgtctatgccaacagcccagtcaactatgannnncnntnnnnngnnncnagncnnnnnnnnnnnnnattgtttgtggacnctntnnnnnnnn
nnntnnnnnnnnnnnnnnnnnannnnntgncatgggtgtgnttggnnnnnnnnnnnnnnngnnnnnnnnnnnnnnnnnnnnnn
nnnnnnnnnnnnnnnnnnnnnnnnnnnannnnncnnnnnnnnnnnnnnnnnnn
(A) CR (122/401): 30.4%
(B)
Max score: 59.0
Total score: 101
Query coverage: 20%
E value: 1e-04
Identity: 78%
- Z48574.1: Tula virus segment S, strain Tula/Moravia/5293Ma/94, genomic RNA.
- Z48573.1: Tula virus segment S, strain Tula/Moravia/5286Ma/94, genomic RNA.

(Note) Simultaneous detection of TULV and PUUV (at lower values).

>seq262:Hantavirus_Puumala_2_13-02-2013_(VirIDv3r520827) Start=12 End=412
ncnnnnncnnncttacatcctttgncnncnngntacttctgannnnnnnnannnnnnnngncaacaagggnnnnnnnngnnnnnnnnnnt
nnnnnggggacaagaatcnngnntcgnnnnnnngnnnanannnnnnnnnnnnnnnnnnnnnncnnnnnnacatctgtatnnnnn
nnnnnnannnnnnnngtcangcatgaaggcagaagagattacnnnnnnnnnnnnnnnnnnnnggnnnnnnnnnnnnnnnnnnac
annnnnnnnnnnnnnngatggtaagcnnnnntatgagtgtgattgntnnnnnnnnnnnnnnnnnnncnnnnnnnnnntnnnnnnnnn
nnnnnnnnnnnnntnnngnnnnnnnnnnnnnnnntacagcagcagttagcctc
(A) CR (151/401): 37.6%
(B)
Max score: 46.4
Total score: 46.4
Query coverage: 6%
E value: 0.78
Identity: 96%

- MF141838.1: Hantaan orthohantavirus isolate AYW118/2015 nucleocapsid protein gene, partial cds.
- MF141833.1: Hantaan orthohantavirus isolate AYW76/2014 nucleocapsid protein gene, partial cds.
- MF141832.1: Hantaan orthohantavirus isolate AWY69/2014 nucleocapsid protein gene, partial cds.
- MF141831.1: Hantaan orthohantavirus isolate AWY68/2014 nucleocapsid protein gene, partial cds.
- MF141830.1: Hantaan orthohantavirus isolate AWY66/2014 nucleocapsid protein gene, partial cds.
- MF141829.1: Hantaan orthohantavirus isolate SG40/2012 nucleocapsid protein gene, partial cds.
- KY807172.1: Hantaan orthohantavirus isolate GAN19/2011 nucleocapsid protein gene, complete cds.
- KY807168.1: Hantaan orthohantavirus isolate GAN13/2011 nucleocapsid protein gene, complete cds.
- KY807167.1: Hantaan orthohantavirus isolate GAN21/2011 nucleocapsid protein gene, complete cds.
- KP970573.1: Hantaan virus isolate JS2 segment S, complete sequence.
- KP970572.1: Hantaan virus isolate JS1 segment S, complete sequence.
- JQ665908.1: Hantaan virus strain WuhanHu16 nucleocapsid gene, partial cds.
- EF208934.1: Hantavirus CJAa1109 nucleocapsid protein gene, partial cds.
- EF208932.1: Hantavirus CJAa594 nucleocapsid protein gene, partial cds.
- DQ658415.1: Hantaan virus strain N8 nucleocapsid protein gene, complete cds.
- AF288659.1: Hantavirus S85-46 nucleocapsid protein mRNA, complete cds.
- AF288294.1: Hantavirus LR1 segment S S protein mRNA, complete cds.
- U71282.1: Hantaan virus nucleocapsid protein gene, partial cds.
- U37768.1: Hantaan virus S segment nucleocapsid protein mRNA, complete cds.
- D25533.1: Hantaan virus gene for nucleocapsid protein, complete cds.
- D25530.1: Hantaan virus gene for nucleocapsid protein, complete cds.
- M14626.1: Hantaan virus S segment encoding nucleocapsid protein.

>seq263:Hantavirus_Puumala_2_13-02-2013_(VirIDv3r520827) Start=12 End=412
nnnnnnngnnnnnnnannncnnnnnnnnnnnnnncnnnnnnnnnnnnnnnnnntntncnnnnnnngnnnnnnnnnnnnnnnnn
nnnnnnnnnnnnnnnnnnnnnnnncngtttaaggatganagctcanancnnnnnnnnnnnnggatnagnagaccaaagcatttatannnt
nntnnnnntanagnnnngnntnnnatgaaanccnangaanngnanncaggtagatttnnannnnnnnnntnnnnnnnnnnnnnnnnn
nnnnnnnnnnnnnnnnnncnnaagtcctgtcatgggtgtgattggattngnnnnnnnnnngnnnnnnnnnnnnnnnnnnnnnnn
nnnnnnnnnnnnnnnccngccnattcnngnnnnncnnnnncnnnngnnnnnnn
(A) CR (130/401): 32.4%

(B)
Max score: 50.0
Total score: 50.0
Query coverage: 6%
E value: 0.064
Identity: 100%

- KU139579.1: Tula virus isolate 10_1188_Magr nucleocapsid protein gene, partial cds.
- KU139578.1: Tula virus isolate 10_0932_Marv nucleocapsid protein gene, partial cds.
  KU139577.1: Tula virus isolate 10_0908_Marv nucleocapsid protein gene, partial cds.
- KU139576.1: Tula virus isolate 10_0905_Marv nucleocapsid protein gene, partial cds.
- KU139575.1: Tula virus isolate 12_1121_Marv nucleocapsid protein gene, partial cds.
- KU139574.1: Tula virus isolate 12_1068_Marv nucleocapsid protein gene, partial cds.
- KU139573.1: Tula virus isolate 10_1533_Marv nucleocapsid protein gene, partial cds.
- KU139572.1: Tula virus isolate 12_526_Magr nucleocapsid protein gene, partial cds.
- KU139571.1: Tula virus isolate 12_0492_Magr nucleocapsid protein gene, partial cds.
- KU139570.1: Tula virus isolate 08_0545_Marv nucleocapsid protein gene, partial cds.
- KU139569.1: Tula virus isolate 08_0362_Marv nucleocapsid protein gene, partial cds.
- KU139568.1: Tula virus isolate 08_0356_Marv nucleocapsid protein gene, partial cds.
- KU139567.1: Tula virus isolate 08_0352_Marv nucleocapsid protein gene, partial cds.
- KU139566.1: Tula virus isolate 08_0350_Marv nucleocapsid protein gene, partial cds.
- KU139565.1: Tula virus isolate 08_0538_Marv nucleocapsid protein gene, partial cds.
- KU139564.1: Tula virus isolate 08_0534_Marv nucleocapsid protein gene, partial cds.
- KU139548.1: Tula virus isolate 10_1625_Marv nucleocapsid protein gene, partial cds.
- KU139546.1: Tula virus isolate 07_0081_Magr nucleocapsid protein gene, partial cds.
- KU139545.1: Tula virus isolate 08_0894_Marv nucleocapsid protein gene, partial cds.
- KU139544.1: Tula virus isolate 08_0849_Marv nucleocapsid protein gene, partial cds.
- KU139543.1: Tula virus isolate 08_0848_Marv nucleocapsid protein gene, partial cds.
- KU139542.1: Tula virus isolate 08_0802_Marv nucleocapsid protein gene, partial cds.
- KU139541.1: Tula virus isolate 08_0789_Marv nucleocapsid protein gene, partial cds.
- KU139540.1: Tula virus isolate 11_1433_Marv nucleocapsid protein gene, partial cds.
- KU139539.1: Tula virus isolate 11_1429_Marv nucleocapsid protein gene, partial cds.
- KU139536.1: Tula virus isolate 11_1373_Marv nucleocapsid protein gene, partial cds.
- HQ697347.1: Tula virus isolate GER/09/2155/Arv nucleocapsid protein gene, partial cds.
- GU300137.1: Tula virus strain dpz06-29 nucleocapsid protein gene, partial cds.
- GU300135.1: Tula virus strain dpz06-1 nucleocapsid protein gene, partial cds.
- EU439952.1: Tula virus strain Sennickerode Sen05/222 nucleocapsid protein gene, partial cds.
- EU439951.1: Tula virus strain Sennickerode Sen05/205 nucleocapsid protein gene, complete cds.
- EU439950.1: Tula virus strain Sennickerode Sen05/204 nucleocapsid protein gene, complete cds.
- EU439949.1: Tula virus strain Sennickerode Sen05/175 nucleocapsid protein gene, complete cds.
- EU439948.1: Tula virus strain Sennickerode Sen05/174 nucleocapsid protein gene, partial cds.
- EU439946.1: Tula virus strain Sennickerode Sen05/121 nucleocapsid protein gene, partial cds.
- AJ223600.1: Tula virus genomic RNA for nucleocapsid protein, (strain Tula/Koziky/5247Ma/94).
- Y13984.1: Tula virus partial genomic RNA for nucleocapsid protein, strain (Tula/D540/Ma/95).
- U95304.1: Tula virus O20 nucleocapsid protein gene, partial cds.
- U95303.1: Tula virus O52 nucleocapsid protein gene, partial cds.
- U95302.1: Tula virus O24 nucleocapsid protein gene, partial cds.
- Z69991.1: Tula virus genomic RNA for nucleocapsid protein, strain (Tula/Moravia/5302v/95).
- Z49915.1: Tula virus segment S, strain Tula/Moravia/5302Ma/94, genomic RNA.
- Z48741.1: Tula virus segment S, strain Tula/Moravia/5294Ma/94, genomic RNA.
- Z48574.1: Tula virus segment S, strain Tula/Moravia/5293Ma/94, genomic RNA.
- Z48573.1: Tula virus segment S, strain Tula/Moravia/5286Ma/94, genomic RNA.

>seq264:Hantavirus_Puumala_2_13-02-2013_(VirIDv3r520827) Start=12 End=412
nnnnnnnnnnnnnnncnnncnnnnnnnnntnnnnnnannnnnnnnnnnnnnnnnnnnnnnnnnnnnnncnnnnnncnnannnnnc
nnnnnnantnngggnnnnnnnnnnnnnnnntnnnnnnnnnnnnnnnnnnnnnnnncancaatggcatnnnnnnnnnnnnnnnnnnn
nnnntnntnnnncnnctgcccagtcaacnatgannnncnntnnatngnnaccaggtaggtttagaacaattgtttgtggncnctttnntnnnnn
nnnnnnnnnnnnnaacancataagtcctgtcatgngtgtgnnnnntnnttcattctttgngnngnntnnnnnnnnannnnnnnnnnnnnn
nnnnnnnnnnnnnnnnnnnnnnnnnnnnnnnnnnnnnnnnnnnnncnnn
(A) CR (128/401): 31.9%
(B)
Max score: 77.0
Total score: 77.0
Query coverage: 29%
E value: 5e-10
Identity: 68%
- AJ223600.1: Tula virus genomic RNA for nucleocapsid protein, (strain Tula/Koziky/5247Ma/94).
- Y13984.1: Tula virus partial genomic RNA for nucleocapsid protein, strain (Tula/D540/Ma/95).
- Z69991.1: Tula virus genomic RNA for nucleocapsid protein, strain (Tula/Moravia/5302v/95).
- Z49915.1: Tula virus segment S, strain Tula/Moravia/5302Ma/94, genomic RNA.
- Z48741.1: Tula virus segment S, strain Tula/Moravia/5294Ma/94, genomic RNA.
- Z48574.1: Tula virus segment S, strain Tula/Moravia/5293Ma/94, genomic RNA.
- Z48573.1: Tula virus segment S, strain Tula/Moravia/5286Ma/94, genomic RNA.

(Note) Simultaneous detection of TULV and PUUV (at lower values).

>seq265:Hantavirus_Puumala_2_13-02-2013_(VirIDv3r520827) Start=12 End=412
tcggccagtacattncaggctttgcacttgcaataatcttgaaggcattgtatatgctgtcaactagagggaggcaaacaatcaaggaaaataagggg
acaagaatccggtttaaggatgacagctcatatgaaganatcaatggcattagacgcccgaaacatctgtatgtgtctatgccaacagcccagtcna
ccatgaaagccgatgaattgacaccaggtaggtttagaacaattgtttgtggactctttcctgctcaaattatgnacagaaacatcataagtcctgtcatg
ggtgtgattggattttctttttcgttaaagattggcctgaaaagattgaggagttccttattaaaccctgcccattcctgaagaaaagtggtcctagtaag
gaa
(A) CR (397/401): 99.0%
(B)
Max score: 710
Total score: 710
Query coverage: 100%
E value: 0.0
Identity: 99%
- Z69991.1: Tula virus genomic RNA for nucleocapsid protein, strain (Tula/Moravia/5302v/95).
- Z49915.1: Tula virus segment S, strain Tula/Moravia/5302Ma/94, genomic RNA.

>seq266:Hantavirus_Puumala_2_13-02-2013_(VirIDv3r520827) Start=12 End=412
tcggcnagnnnnnnnnnnncntnncnnntgnnnnnnnnnnnnnnnnnnnnnnnnnnnnnnnnnnnnnnnnnnnnccancnnacaatcnn
ngannataaggggnnnnnnnnnnnnnnnnnngnnnnnnnnnnnnnnnnnnnnnnnnnnnnnnnnnnnnnnnnnnnnnnnnnntnnnn
nnnngnntnnnncaaangcncagncanctangannnncnangaanngnnnnnnnncnnnnnnnnnnnnnnnnnntnncnggnnnnat
gnnnnnnnnnnnnnnnncnnnnncnnnannngtccngttannnnnnnnnnnnnnnnnnnnnnnnnnnnnnnnnnnnnnnnnncctgaaa
agattgaggagttccttattaannncnnnnnnnnnnnnnnnnnnnnnnnnnnnnnnnnnnnnnn
(A) CR (107/401): 26.7%
(B)
Max score: 53.6
Total score: 53.6
Query coverage: 7%
E value: 0.005
Identity: 100%
- AJ223601.1: Tula virus genomic RNA for nucleocapsid protein, (strain Tula/Koziky/5276Ma/94).
- AJ223600.1: Tula virus genomic RNA for nucleocapsid protein, (strain Tula/Koziky/5247Ma/94).
- Y13984.1: Tula virus partial genomic RNA for nucleocapsid protein, strain (Tula/D540/Ma/95).

- Z69991.1: Tula virus genomic RNA for nucleocapsid protein, strain (Tula/Moravia/5302v/95).
- Z49915.1: Tula virus segment S, strain Tula/Moravia/5302Ma/94, genomic RNA.
- Z48741.1: Tula virus segment S, strain Tula/Moravia/5294Ma/94, genomic RNA.
- Z48574.1: Tula virus segment S, strain Tula/Moravia/5293Ma/94, genomic RNA.
- Z48573.1: Tula virus segment S, strain Tula/Moravia/5286Ma/94, genomic RNA.

>seq267:Hantavirus_Puumala_2_13-02-2013_(VirIDv3r520827) Start=12 End=412
nnnnnnnnnnnnnnnnnnnnnnnnnnnnnnnnnnnngnnnnnnnnnnnnnnnnnnnnnnnnnnnnnnnnnnnncgnnnnn
nnnnnnnnnnnnnnnnnnnnnnnnnnnnnttnnnnnnnnnnnnnnnnnnnnnnnnnnncnnnncnnnnnnnnnnnnnnnnnn
nnnnnnnnnnnnnnnnnnnnnnnnnnnnnnnnnnnnnnnnncannnnnnncnnannnnnnnnnnnnnnnnnnnnnnnnnn
nnnnnnnnnnnnnnnnnnnnnnnnnnnnnnngnnnnnnnnnnnncnngngngtcattgntnnnnnnnnnnnnnnnnnnnn
nnnnnnnnnnnnnnnnnnnnncnnnnnnnnnnnnnnnnnnnnnnnnnnnnnnnnnnncnnnnnnnnnnnn
(A) CR (25/401): 6.2%
(B)
No BLASTN identification.

>seq268:Hantavirus_Puumala_2_13-02-2013_(VirIDv3r520827) Start=12 End=412
nnnnnnnnnnnnnnnnnnnnnnnnnnnnnnnnnnnnnnnnnnnnntnnnnntntgctctcaangcgngggagncagncnnnnnn
nnnnnnnnnnnnnnnnccnnnaaggtttaaggatgnnnnnnnnnnnnnnncannnnnnnnnnnnnnaaccaaaacatcttnnnnn
nnnnnnnncaaatgcccagncangnannnnnnnnnganganctcgnnnnnncnnnnnnnnnnnnnnnnnnntnnnnggngnttnggnnn
nnnnnnnnnnnnnnnnnnnncnncnnnnnnnnnnnncnnnnnnnnngnnnnnnnnnnnnnnnnnnnnngnnnnnnnnnnnnnnnnn
nnnnnnnncnnnnnnnnnnnnnnntnnncnnnnnnnnnnnnnnnnnnnnnnctnnnnngnnnnn
(A) CR (98/401): 24.4%
(B)
No BLASTN identification.

>seq269:Hantavirus_Puumala_2_13-02-2013_(VirIDv3r520827) Start=12 End=412
nnntnnnnnnnnnnnnnnnnnnnnnnnnnnnnnnnnnnnnnnnnnnnnntgctctcaacgngagggagncagnnnnnnnn
nnnnaanannnnnnnnnnnnnnncnnnntnnnnnnnnnnnnnnnnnnnnnnnnnnnnnnnnaaaccaaaacatctnnnc
nnnnnnnnnnnnnannnnnnnnnnnnnnnnnnnnnnnnnnacntnnnnnnnnnnnnnnnnnnnnnnnnnntnnnnggngnttnn
nnnnnnnnnnnnnnnnnnnnnnnnnccnnnnnnnnnnncnnnnnnnngnnnnntnnnnnnnnnnnnnnnngactggatggng
nnnnnnnnnnnnnnnnnnncnnngagtgccnattnnnnnnnnnnnnnnnnnnnnnnnnnn
(A) CR (79/401): 19.7%
(B)
No BLASTN identification.

>seq270:Hantavirus_Puumala_2_13-02-2013_(VirIDv3r520827) Start=12 End=412
nnnnnnnnnnnnnnnnnnnnnnnnnnnnnnnnnnnnnannnnnnnnnnnnnnnnnnnnnnnnnnnnnnnngnnnnnnaan
cnngnnnnnnnnnngnnnnnnnnnnnnnntnnnnnnnnnannnnnnnnnnnnnnannnnnnnnnncnnnnnnnnnnnannnnn
nntatgtgtctatgcctacagccnagtcaacnatganannncnannnnnnnnnnnnnnnnnnnnnnnnnnnnnnnnnnnnnnn
ngnnnnnnnnnnnnnnnnnnnnnnnnnnnnnangtcctgtcatgggtgtgattnnannnnnnnnnnntnngnnnnnnnnnnnnnn
nnnnnnnntgngnnnnnnnnnnnnnnnnnngnngtnnnnnnnnnnnnnnnnnnnnnnnnnn
(A) CR (78/401): 19.4%
(B)
Max score: 50.0
Total score: 50.0
Query coverage: 8%
E value: 0.064
Identity: 91%
- FJ495098.1: Tula virus strain TULV/Sestrze/Mag98_02 segment S nucleocapsid protein gene, partial cds.
- FJ495096.1: Tula virus strain TULV/Crna vas/Ma225_95 segment S nucleocapsid protein gene, partial cds.
- Z49098.1: Prospect Hill virus RNA, strain PH-1.

- M34011.1: Prospect Hill virus (PHV) S genomic segment encoding N protein, complete cds.

(Note) Simultaneous detection of TULV and PUUV (at lower values).

>seq271:Hantavirus_Puumala_2_13-02-2013_(VirIDv3r520827) Start=12 End=412
nnnnnnnnnnnnnnnnnnnnnnnnnnnnnnnnnnnnnnnnnnnnnnnnnnnnnnnnnnnnnnnnnnnnncnnnnngnnnnnnnn
cnnggaaaataaggggacaagaatccngnntnnnnnnnnnnnnnnnnnnnnnnnnnnnnnnnnnnnnnnnnngcnnnnnnnnnn
nnnnntnntnnnnnnanctnnnnngnnngnnnnnnnggcannagnnnnnnnnnnannnnnntnnnnnnnnnnnnnnnnnnnnn
nnnnnnnnnnnnnnnnnnnnnnnnnnnnnnnnnnnnnccctgtcatgggtgtgattggnnnnnnnnnnnnnnnnnnnnnnnnncgnnnnnnn
nnnnnnnnnnnnnnnnnnnnnnnnnnnnnnnnncnnnnnnnnnnnnnnnnnnnnnnnnnnnnnn
(A) CR (68/401): 16.9%
(B)
Max score: 42.8
Total score: 42.8
Query coverage: 5%
E value: 9.5
Identity: 100%

- Y13984.1: Tula virus partial genomic RNA for nucleocapsid protein, strain (Tula/D540/Ma/95).
- Z69991.1: Tula virus genomic RNA for nucleocapsid protein, strain (Tula/Moravia/5302v/95).
- Z49915.1: Tula virus segment S, strain Tula/Moravia/5302Ma/94, genomic RNA.
- Z48741.1: Tula virus segment S, strain Tula/Moravia/5294Ma/94, genomic RNA.
- Z48574.1: Tula virus segment S, strain Tula/Moravia/5293Ma/94, genomic RNA.
- Z48573.1: Tula virus segment S, strain Tula/Moravia/5286Ma/94, genomic RNA.

>seq272:Hantavirus_Puumala_2_13-02-2013_(VirIDv3r520827) Start=12 End=412
nnnnnnnnnnnnnnnnnnnnnnnnnnnnnnnnnannnnnnnnnnnnnnnnnnnnnnnnnnnnnnnnnnnnnngnnnacnannnnn
nnnnnnnnnnnncnnnnnnnnncnnnnnnnnnngnnnnnnnnnnnnnnnnnnnnnnnnnnnnnnnnnnnnnnnnnnnnnnnnnnnn
cngnncnnnnntnnnnnnnangcccngtcangnntnnnnnnnnnnnnnnnnnnnnnnnnnnnnnnnnnnnnnnnnnnnnnnnnnngtn
ttttgnnnnnnnnnnnnngnnnntnnnnnnnnnnnnnnnnnnnnnnnnnnnnngnnnnnggnnnnnnnnnnnnnnnngnnnnnnnnnn
nnnnnnnnnnnnnnnnnnnnnnnnnnnnnnnnnncnnngnnnnnnnnnnnnnnnnnnnnnnnnnnnnnnn
(A) CR (38/401): 9.5%
(B)
No BLASTN identification.

>seq273:Hantavirus_Puumala_2_13-02-2013_(VirIDv3r520827) Start=12 End=412
nnnnnnnnnnnnnnnnnnnnnnnnnnnnnnnnnnnnnnnnnnnnnnnnnnngnnnnnnnnngnnaactagnggnnnacnannnnnn
nnnnnnnnnnnnnnnnacccggattcgatttaagnnnnntacatcattnnaagncatcaannnnnnnnnngnnnnnannnnnnnnnnnntgn
ntctntnnnnnnnnnnnnnnnnnnnnnnnnnnnnnagaagannnnnnnnnnnnnnnnnnnnnnannnnnnnnnnnnnnnnnnnnntnn
nnnnnnnnnnnnnnnnnnnnnnnnncnngngtcctgtnatgngngtgnnnnnnnnnnnnnnnnnnnnnnnnnnnntnnnnnnnnnnnn
nnnnnnnnnnnnnnnnnnnnnnngnnnnnnnnnnnnnnnnnnnnnnnnnnnnnnnnnnnnnncn
(A) CR (84/401): 20.9%
(B)
No BLASTN identification.

SUPPLEMENT 4

Validation of PathogenID v3.0 resequencing microarray using plasmids coding orthohantavirus N proteins: comparison between hybridization results obtained with PUUV Sotkamo alone and in a pool with TULV and HTNV.

Plasmid containing the S segment of Puumala virus (PUUV, strain Sotkamo 2009), were hybridized on the PathogenID v3.0 resequencing microarray separately or in pool with Tula virus (TULV, strain Moravia) and Hantaan virus (HTNV, strain 76/118) to compare the efficiency of hybridization.

For each PUUV sequence tiled on the microarray (seq222 - seq243), a comparative analysis is presented with PUUV alone or PUUV mixed with TULV and HTNV. This comparison includes Call Rate and BLASTN results detailed in Supplement 3.

Complementary informations are found in Table 2 and Figure 2.

PUUV sequence on the chip (position)	Call Rate PUUV (%)	Call Rate PUUV+TULV+HTNV (%)	BLASTN identification PUUV	BLASTN identification PUUV+TULV+HTNV
seq222	23.9	28.4	yes: Sotkamo	yes: Sotkamo
seq223	23.7	38.1	no	yes: Sotkamo
seq224	18.9	26.7	no	yes: Sotkamo
seq225	97.7	98.5	yes: Sotkamo	yes: Sotkamo
seq226	26.2	31.9	no	yes: Sotkamo + others°
seq227	39.6	42.9	yes: Sotkamo	yes: Sotkamo
seq228	22.7	33.9	yes: Sotkamo + others° Finland	yes: Sotkamo
seq229	19.4	27.9	no	yes: others°
seq230	33.9	38.6	yes: Sotkamo	yes: Sotkamo
seq231	75.0	78.0	yes: Sotkamo	yes: Sotkamo
seq232	34.4	44.9	yes: Sotkamo + others° Finland	yes: Sotkamo
seq233	19.7	27.4	yes: Sotkamo + others°	yes: others°
seq234	62.1	64.3	yes: Sotkamo	yes: Sotkamo
seq235	7.0	25.2	no	yes: PUUV+ TULV, HTNV
seq236	19.9	30.9	yes: Sotkamo + others°	yes: others°
seq237	35.2	40.9	yes: Sotkamo	yes: Sotkamo
seq238	24.7	27.2	no	no
seq239	34.4	41.9	yes: Sotkamo	yes: Sotkamo
seq240	38.3	46.6	yes: Sotkamo	yes: Sotkamo
seq241	26.9	32.2	no	no
seq242	30.8	35.8	yes: Sotkamo + others°	yes: Sotkamo + others°
seq243	25.1	35.1	no	no

° others: PUUV geographical isolates other than Sotkamo.

SUPPLEMENT 5

Detection and genetic characterization of hantavirus species endemic in Europe using the PathogenID v3.0 resequencing microarray: Call Rate and BLASTN analysis

Ampified cDNAs of RNA extracts from European rodents infected with hantaviruses or from hantavirus laboratory strains were hybridized on the PathogenID v3.0 resequencing microarray.

After hybridization of the samples to each hantavirus sequence tiled on the array (seq222 - seq273 the following parameters are indicated (A) the Call Rate (CR: % of determined/total number of nucleotides) of the output sequence; (B) the raw results when BLASTN analysis allowed virus/variant identification including: Max score, Total score, Query coverage, E value, Identity. When the same score was obtained for different sequences (multiple BLASTN identical results), all data are presented with corresponding accession number(s).

Complementary informations are found in Tables 3-7.

<u>Species: Puumala orthohantavirus (PUUV)</u>

<u>Origin: France</u>

<u>PUUV 87 Ardennes</u>

>seq222:1-09-04-2013_(VirIDv3r520827) Start=12 End=412
ttggagtgtatgttataggctncacacttcctattatcctgnnnncntnnnnnnngntgtcancacgtggnagncaaactgtgaaagnaancnaag
ggnctcgtatccgttncnnngnngncacttccttcgaggncnncnnnggnnnnagnannnnnnnnncnnnnnnnnngtttccatgccnnctgc
tcaatcaactatgaaggcnnnnnnnnnnnnnnnnngggcgatttagaaccannnnnngtnnnncnnnncnancncaaattcaagnacgcaa
canaatgagccctgtnatgngagnnnnnnnnnnnnnnnnnntnncaagnnnnnngcagancggattagggaattcatggnaaannnntn
ncnttttatcaaaccagangngnagncagnncnn
(A) CR (263/401): 65.6%
(B)
Max score: 226
Total score: 226
Query coverage: 98%
E value: 4e-55
Identity: 66%

- KY364995.1: Puumala orthohantavirus isolate Ardennes_2011_87 nucleoprotein gene, complete cds.
- KT247593.1: Puumala virus isolate PUUV/Ardennes/Mg75/2011 segment S nucleoprotein gene, complete cds.
- KT247592.1: Puumala virus isolate PUUV/Ardennes/Mg156/2011 segment S nucleoprotein gene, complete cds.

>seq223:1-09-04-2013_(VirIDv3r520827) Start=12 End=412
ntnntnnnngngntnnnnncnncncncntcctattatcctgnnnnntnnnnnnnnnnntgtcancacgtggnagncaaactgtgaaagnann
nnnnnncnnnganncnnnnncaaagntgnnnnngnnnnnnnnnnnnnnnnnncngcagnannnnnnnncntnnnnntgtttccat
gccnnctgctcaancaantnngnnnnnnnnnnnnnnnnnnnngggcgatttagaaccatagnntgtggtcnnnntnnanctcnnnnncn
nnnnnnnnnnnnnnnnncnntgnnnnnnnnnnnnnnnnnnnnnnnnnnnnnnnnnnnnnnnnggcagatcggnnnnnnnnn
nngngnnnnnnnnnnnnnnnnnnnnnnnnnnnnnnnnnnnnnnnnn

(A) CR (144/401): 35.9%
(B)
Max score: 44.6
Total score: 89.1
Query coverage: 14%
E value: 2.5
Identity: 90%
- KY364995.1: Puumala orthohantavirus isolate Ardennes_2011_87 nucleoprotein gene, complete cds.

>seq230:1-09-04-2013_(VirIDv3r520827) Start=12 End=412
ttggagtgtatgttataggcttcacacttcctattatcctgaaggctttatatatgttgtcaacacnnggnnnnnnannnnngnaannannnnnnn
nnctcgnatccgtttcaaagntgncacttccttcgnggacatcaatggtatcaggnnnccnnnncntcnnnntgtttccangccnnctgctcaatc
aactatgaaggctgaagnannnncncctgggcgatttagaaccanngtntgtggtctatttccaactcaaattcaagnacgcaacanaatgagccc
tgnnatgggagnnnnnnnnnttnnnntntttgncaaggnnnnngcagatcggntnnggnnannnnnggnnnaanngtgnnnttttatcaaa
ccagangngnngncagnncnn
(A) CR (296/401): 73.8%
(B)
Max score: 351
Total score: 351
Query coverage: 98%
E value: 1e-92
Identity: 74%
- KY364995.1: Puumala orthohantavirus isolate Ardennes_2011_87 nucleoprotein gene, complete cds.
- KT247593.1: Puumala virus isolate PUUV/Ardennes/Mg75/2011 segment S nucleoprotein gene, complete cds.
- KT247592.1: Puumala virus isolate PUUV/Ardennes/Mg156/2011 segment S nucleoprotein gene, complete cds.

>seq237:1-09-04-2013_(VirIDv3r520827) Start=12 End=412
tggagtgtatgttataggctncacacttcctattatcctgaaggctttatatangttgtcancacnngnnnnnnnannnnngnaagnanncnnnnn
gnnnnnnnnccgttncnnanntgncacttccttcgaggacatcaatggnnncngnnnnnnnnnnncntcnnnntntttccatgccnnctgctca
atcaactatganggctgaannannnacncctgggcgatttagaaccanagtntgtggtcnatttccaactcaaattcaagnncgcaacanantgan
ccctgnnatgngagncnnnnnnntnnnnnnttttgncaaggnnngngcagntcggnnnnnnnnannnnnggnnaaanngtgncnttttat
caaaccagnggngnagncannncnnn
(A) CR (272/401): 67.8%
(B)
Max score: 268
Total score: 268
Query coverage: 98%
E value: 1e-67
Identity: 69%
- KY364995.1: Puumala orthohantavirus isolate Ardennes_2011_87 nucleoprotein gene, complete cds.
- KT247593.1: Puumala virus isolate PUUV/Ardennes/Mg75/2011 segment S nucleoprotein gene, complete cds.
- KT247592.1: Puumala virus isolate PUUV/Ardennes/Mg156/2011 segment S nucleoprotein gene, complete cds.

>seq238:1-09-04-2013_(VirIDv3r520827) Start=12 End=290
attnnngngnntgnnnnnggcnncncncttcctattatcctganggctnnannnnnnntgtcancacgtggnagncannnnnngnnnnnnn
nnnnanngnctcgnatccgtttcaaagnnnnnnnngnnnnnnngnncannantgnnnnnnnnnnnnnncnnncnnnnnnnnnnnc

nnnccnnctgctcaatcaactatgaaggcngnnnnnnnannnnntgnnnnnntnngancnngngnntgtggtcnatttccaactcaaattcaa
gnacgcaac
(A) CR (147/279): 52.7%
(B)
Max score: 62.6
Total score: 108
Query coverage: 55%
E value: 6e-06
Identity: 68%
- KY364995.1: Puumala orthohantavirus isolate Ardennes_2011_87 nucleoprotein gene, complete cds.
- KT247593.1: Puumala virus isolate PUUV/Ardennes/Mg75/2011 segment S nucleoprotein gene, complete cds.
- KT247592.1: Puumala virus isolate PUUV/Ardennes/Mg156/2011 segment S nucleoprotein gene, complete cds.
- AJ277034.1: Puumala virus RNA for nucleocapsid protein (N gene), strain Couvin/59Cg/97.
- AJ277033.1: Puumala virus RNA for nucleocapsid protein (N gene), strain Momignies/55Cg/96.
- AJ277032.1: Puumala virus RNA for nucleocapsid protein (N gene), strain Momignies/47Cg/96.

>seq239:1-09-04-2013_(VirIDv3r520827) Start=12 End=290
attggngtgtatgttataggctncacacttcctattatcctgaaggctttatntatgttgtcancacnnggnnnnnnannnnngnaagnnannnnn
nngnctcgnatccgtttcaaagntgncacttccttcgaggacatcaatggtatcagnnnnnnnnnnnnnnnnnnnnntttccatgccnnctgctca
atcaactatgaaggctgnagaattaacnnctgggcgatttagaaccanagtntgtggtcnatttccaactcaaattcaagnacgcaac
(A) CR (219/279): 78.5%
(B)
Max score: 288
Total score: 288
Query coverage: 100%
E value: 9e-74
Identity: 78%
- KY364995.1: Puumala orthohantavirus isolate Ardennes_2011_87 nucleoprotein gene, complete cds.
- KT247593.1: Puumala virus isolate PUUV/Ardennes/Mg75/2011 segment S nucleoprotein gene, complete cds.
- KT247592.1: Puumala virus isolate PUUV/Ardennes/Mg156/2011 segment S nucleoprotein gene, complete cds.

>seq240:1-09-04-2013_(VirIDv3r520827) Start=12 End=290
attggngtgtatgttataggctncacacttcctattatcctgnnnnntngnngnnnnntgtcancacgtggnagncaaactgtgnnagnaancaaa
gggactcgtatccgttncnnannnnncncnnncnncnaggncatcaatggnatcaggannccnnngcntnnnnntgtttccatgccnnctgct
caatcaactatgaaggctgaaganttaacnnctgggcgatttagaaccatagtatgtggtctatttcnannncnanntcaagnacgcaac
(A) CR (216/279): 77.4%
(B)
Max score: 268
Total score: 268
Query coverage: 100%
E value: 8e-68
Identity: 77%
- KY364995.1: Puumala orthohantavirus isolate Ardennes_2011_87 nucleoprotein gene, complete cds.
- KT247593.1: Puumala virus isolate PUUV/Ardennes/Mg75/2011 segment S nucleoprotein gene, complete cds.
- KT247592.1: Puumala virus isolate PUUV/Ardennes/Mg156/2011 segment S nucleoprotein gene, complete cds.

>seq241:1-09-04-2013_(VirIDv3r520827) Start=12 End=290
attggagtgtntgttatngnnnccncnnntnnnnnnnntcctgaaggctttatatatgttgtcaacacgtggnagncaaactgtgaaagnaancana
gggactcgtatccgtttcaaagntgncacttccttcgaggacatcaatggtancagnannnnnnnnncnnnnnnnnnnntccntgccnnctgctc
aancaactannnagncngnnnnnnnannnnnngggcgatttagaaccatagtntgtggtcnatttccaactcaaattnaagtncgnnnc
(A) CR (206/279): 73.8%
(B)
Max score: 239
Total score: 239
Query coverage: 98%
E value: 4e-59
Identity: 74%
- KY364995.1: Puumala orthohantavirus isolate Ardennes_2011_87 nucleoprotein gene, complete cds.
- KT247593.1: Puumala virus isolate PUUV/Ardennes/Mg75/2011 segment S nucleoprotein gene, complete cds.
- KT247592.1: Puumala virus isolate PUUV/Ardennes/Mg156/2011 segment S nucleoprotein gene, complete cds.

PUUV 153 Ardennes

>seq222:Lung29_pure_21-02-2013_(VirIDv3r520827) Start=12 End=412
ttggagtgtatgttataggcttcacacttcctattatcctgaangctttntntatgttgtcaacacgtgggagacaaactgtgaaagaaaacaaagggac
tcgtatccgtttcnnagntgacacttccttcgangacancnatggnnncagganncnanngcnnnnnnntgtttccatgcctactgctcaatcaac
tatgaaggctgnnnnnnnnnnnnntgggcgatttagaaccatagnntgtggnnnnnntccanctcaaattcaagtacgcaacataatgagccct
gtaatgggagtcnntgnnnnnnntnnttttntcaagnncngggcagatcggattagggaattcatggaaaaagagtgcccttttatcaaaccagag
gtgnagncagnnnnn
(A) CR (333/401): 83.0%
(B)
Max score: 488
Total score: 488
Query coverage: 98%
E value: 7e-134
Identity: 84%
- KY364995.1: Puumala orthohantavirus isolate Ardennes_2011_87 nucleoprotein gene, complete cds.
- KT247593.1: Puumala virus isolate PUUV/Ardennes/Mg75/2011 segment S nucleoprotein gene, complete cds.
- KT247592.1: Puumala virus isolate PUUV/Ardennes/Mg156/2011 segment S nucleoprotein gene, complete cds.

>seq223:Lung29_pure_21-02-2013_(VirIDv3r520827) Start=12 End=412
ttgnngngnnngntnnngncnncncacttcctattatcctgnnnnntnnnngnnnnntgtcaacacgtgggagacaaactgtgaaagaaaacn
nannnnnnganncnnnttcaaagatgncnnngncnnnnnnnnnnnnnntnncnncaggagaccanagcatcnatatgtttccatgccnn
ctgctcaatcaactatgnagncngnnnnnnnnnnnnnngggcgatttagaaccatagtatgtggtctntttcnaactcaannncnngnanncnn
nnnnnngngccctgngnnnncnnnnnnnnnnnnnnnnnnnnnnnnnnnnnngnnnngggcagatcggattnngnnnnnngnggnnnna
nngngnnnnnnnnnnnnnnnnnnnnnnnnnnnnnnnn
(A) CR (210/401): 52.3%
(B)
Max score: 141
Total score: 141
Query coverage: 59%
E value: 1e-29
Identity: 67%

- KY364995.1: Puumala orthohantavirus isolate Ardennes_2011_87 nucleoprotein gene, complete cds.
- KT247593.1: Puumala virus isolate PUUV/Ardennes/Mg75/2011 segment S nucleoprotein gene, complete cds.
- KT247592.1: Puumala virus isolate PUUV/Ardennes/Mg156/2011 segment S nucleoprotein gene, complete cds.

>seq229:Lung29_pure_21-02-2013_(VirIDv3r520827) Start=12 End=412
nnncnnngnnnnnnnnnnnncnncnnnnntnnnnnnnnnnngnnnnntnnnnntatgttgtcaacacnngggngnnnnnnnnnggnn
nnnnnnnnnnncggnnnnnnnnnnnngnnnnnnnnnggngnnnnnnnnnnnanncnnnncnnnnnnnnnnnnnnnnnnnn
gnnnnnnnnnncctnctgctcaatcaactatgaaggcngnnnnnnnnnnnnnncnnnnnnnnnnnnngnnnngngtnnnnnnnnn
nnnnnnnncnnnnnccnnnnnnnnngnnnnncgntnnnnnnnnnnnnnnnnnnnnnnnnnnnnnnnnnggncnnngcnnnn
nnnnntnngnnngncatggnaaaagagtgccnnttnnnnnnnnnannnncnnngncnnnnnnn
(A) CR (106/401): 26.4%
(B)
Max score: 44.6
Total score: 44.6
Query coverage: 6%
E value: 2.7
Identity: 96%
- KY364995.1: Puumala orthohantavirus isolate Ardennes_2011_87 nucleoprotein gene, complete cds.
- KU670634.1: Puumala hantavirus isolate KS11/2387 nucleocapsid protein gene, partial cds.
- KU670633.1: Puumala hantavirus isolate KS11/2384 nucleocapsid protein gene, partial cds.
- KT247593.1: Puumala virus isolate PUUV/Ardennes/Mg75/2011 segment S nucleoprotein gene, complete cds.
- KT247592.1: Puumala virus isolate PUUV/Ardennes/Mg156/2011 segment S nucleoprotein gene, complete cds.
- DQ322681.1: Puumala virus strain Cologne Mu05/273 nucleocapsid protein (N) gene, partial cds.
- DQ322679.1: Puumala virus strain Cologne Mu05/270 nucleocapsid protein (N) gene, partial cds.
- DQ322678.1: Puumala virus strain Cologne Mu05/269 nucleocapsid protein (N) gene, partial cds.
- DQ322676.1: Puumala virus strain Cologne Mu05/262 nucleocapsid protein (N) gene, partial cds.
- DQ322675.1: Puumala virus strain Cologne Mu05/258 nucleocapsid protein (N) gene, partial cds.
- DQ322672.1: Puumala virus strain Cologne Mu05/244 nucleocapsid protein (N) gene, partial cds.
- DQ322670.1: Puumala virus strain Cologne Mu05/242 nucleocapsid protein (N) gene, partial cds.
- DQ322669.1: Puumala virus strain Cologne Mu05/241 nucleocapsid protein (N) gene, partial cds.
- EU004036.1: Puumala virus strain Kobl3/05 nucleocapsid protein (N) mRNA, partial cds.
- DQ408276.1: Puumala virus strain Cologne Mu05/162 nucleocapsid (N) gene, partial cds.
- DQ408275.1: Puumala virus strain Cologne Mu05/161 nucleocapsid (N) gene, partial cds.
- DQ408274.1: Puumala virus strain Cologne Mu05/286 nucleocapsid (N) gene, partial cds.
- DQ408273.1: Puumala virus strain Cologne Mu05/285 nucleocapsid (N) gene, partial cds.
- DQ408271.1: Puumala virus strain Cologne Mu05/281 nucleocapsid (N) gene, partial cds.
- DQ408268.1: Puumala virus strain Cologne Mu05/274 nucleocapsid (N) gene, partial cds.
- AJ277076.1: Puumala virus RNA for nucleocapsid protein (n gene), strain CG14445.
- AJ277075.1: Puumala virus RNA for nucleocapsid protein (n gene), strain CG14444.
- AJ277034.1: Puumala virus RNA for nucleocapsid protein (N gene), strain Couvin/59Cg/97.
- AJ277033.1: Puumala virus RNA for nucleocapsid protein (N gene), strain Momignies/55Cg/96.
- AJ277032.1: Puumala virus RNA for nucleocapsid protein (N gene), strain Momignies/47Cg/96.
- AJ277031.1: Puumala virus RNA for nucleocapsid protein (N gene), strain Montbliart/23Cg/96.
- AJ238788.1: Puumala virus RNA for nucleocapsid protein, strain Karhumaki.
- AJ238779.1: Puumala virus mRNA for nucleocapsid protein.

>seq230:Lung29_pure_21-02-2013_(VirIDv3r520827) Start=12 End=412

ttggagtgtatgttataggcttcacacttcctattatcctgaaggctttatatatgttgtcaacacntggnagnnaannngngaaagaaancnnagng
nctcgtatccgtttcaaagatgacacttccttcgaggacatcaatggtatcaggagaccanagcatcnntatgtttccatgcctnctgctcaatcaacta
tgaaggctgaagaattaacncctgggcgatttagaaccatagtatgtggtctatttccaactcaaattcaagnacgcaacanaatgagccctgtaatg
ggagncantggntttnnttttttgtcaagnncngggcagatcggattaggnnannnnnggaaaaagagtgccctttatcaaaccagaggtgna
gncagnnnnn

(A) CR (359/401): 89.5%

(B)

Max score: 581
Total score: 581
Query coverage: 98%
E value: 4e-162
Identity: 91%

- KY364995.1: Puumala orthohantavirus isolate Ardennes_2011_87 nucleoprotein gene, complete cds.
- KT247593.1: Puumala virus isolate PUUV/Ardennes/Mg75/2011 segment S nucleoprotein gene, complete cds.
- KT247592.1: Puumala virus isolate PUUV/Ardennes/Mg156/2011 segment S nucleoprotein gene, complete cds.

>seq234:Lung29_pure_21-02-2013_(VirIDv3r520827) Start=12 End=238

ncagacattcagnnggntataanccncnannnannnncnnnnnttgttgccnnnnngnnnnnnnnngnngnagnaaaggcagtgnngat
ggacccagatgacgttaacaaanacacacngcangcaagncancagncagtgngngcncnggaggacaaacttgcngacttcaagcgacag
atggcngnngnnnngnnnnannnnnnnannnnnngnnnnannnnctga

(A) CR (146/227): 64.3%

(B)

Max score: 150
Total score: 150
Query coverage: 50%
E value: 1e-32
Identity: 87%

- KT247592.1: Puumala virus isolate PUUV/Ardennes/Mg156/2011 segment S nucleoprotein gene, complete cds.

>seq237:Lung29_pure_21-02-2013_(VirIDv3r520827) Start=12 End=412

tggagtgtatgttataggcttcacacttcctattatcctgaaggctttatatatgttgtcaacacntggnagnnnannnnngaaagnaaacanagngn
ctnnnnnccgtttcaaagatgacacttccttcgaggacatcaatggnatcngnannnnnnnagcatctatatgtttccatgcctactgctcaatcaact
atgaaggctgaagaattaacccctgggcgatttagaaccatagtatgtggtctatttccaactcaaattcaagnacgcaacanaatgagccctgtaatg
ggagtcantngntttnnttttttgtcaagnncngggcagatcggattaggnnannnnnggaaaaagagtgccctttatcaaaccagaggtgnag
ccagnancnn

(A) CR (351/401): 87.5%

(B)

Max score: 547
Total score: 547
Query coverage: 98%
E value: 9e-152
Identity: 88%

- KY364995.1: Puumala orthohantavirus isolate Ardennes_2011_87 nucleoprotein gene, complete cds.
- KT247593.1: Puumala virus isolate PUUV/Ardennes/Mg75/2011 segment S nucleoprotein gene, complete cds.
- KT247592.1: Puumala virus isolate PUUV/Ardennes/Mg156/2011 segment S nucleoprotein gene, complete cds.

>seq238:Lung29_pure_21-02-2013_(VirIDv3r520827) Start=12 End=290

attgganngnangtnnnnggctncncncttcctattatcctgaaggctntanntntgttgtcaacacgtgggagacaaacnnngnnnnnnnnnn
nagngnctcgtatccgtttcaaagntnncnnngnnnnncngnacancaatggngncnnnnnnnnncnnncngnnnnnnntnncntgccn
nctgctcaatcaactatgaaggctgaagannnaannncngnncgntttagaaccanngtatgtggtcnatttccaanncaanttcaagnacgcaa
c

(A) CR (190/279): 68.1%

(B)

Max score: 156
Total score: 156
Query coverage: 100%
E value: 4e-34
Identity: 66%

- KY364995.1: Puumala orthohantavirus isolate Ardennes_2011_87 nucleoprotein gene, complete cds.
- KT247593.1: Puumala virus isolate PUUV/Ardennes/Mg75/2011 segment S nucleoprotein gene, complete cds.
- KT247592.1: Puumala virus isolate PUUV/Ardennes/Mg156/2011 segment S nucleoprotein gene, complete cds.

>seq239:Lung29_pure_21-02-2013_(VirIDv3r520827) Start=12 End=290

attggagtgtatgttataggcttcacacttcctattatcctgaaggctttatatatgttgtcaacacntggnagnnnannnnngaaagnaaacanagn
gnctcgtatccgtttcaaagatgacacttccttcgaggacatcaatggtatcaggannnnnnngnnnnnnnntgtttccatgccnactgctcaatca
actatgaaggctgaagaattaacncctgggcgatttagaaccatagtatgtggtctatttccaactcaaattcaagtacgcaac

(A) CR (248/279): 88.9%

(B)

Max score: 392
Total score: 392
Query coverage: 100%
E value: 3e-105
Identity: 89%

- KY364995.1: Puumala orthohantavirus isolate Ardennes_2011_87 nucleoprotein gene, complete cds.
- KT247593.1: Puumala virus isolate PUUV/Ardennes/Mg75/2011 segment S nucleoprotein gene, complete cds.
- KT247592.1: Puumala virus isolate PUUV/Ardennes/Mg156/2011 segment S nucleoprotein gene, complete cds.

>seq240:Lung29_pure_21-02-2013_(VirIDv3r520827) Start=12 End=290

attggagtgtatgttataggcttcacacttcctattatcctgannnntnnnngnnnnntgtcaacacgtgggagncaaactgtgaaagaaaacaaag
ggactcgtatccgtttcnaagntgncactncctncnaggacatcaatggtatcaggagaccanagcatcnatatgtttccatgccnactgctcaatca
actatgaaggctgaagaattaacccctgggcgatttagaaccatagtatgtggtctatttccancncaaattcaagtacgcaac

(A) CR (254/279): 91.0%

(B)

Max score: 408
Total score: 408
Query coverage: 100%
E value: 4e-110
Identity: 91%

- KY364995.1: Puumala orthohantavirus isolate Ardennes_2011_87 nucleoprotein gene, complete cds.
- KT247593.1: Puumala virus isolate PUUV/Ardennes/Mg75/2011 segment S nucleoprotein gene, complete cds.
- KT247592.1: Puumala virus isolate PUUV/Ardennes/Mg156/2011 segment S nucleoprotein gene, complete cds.

>seq241:Lung29_pure_21-02-2013_(VirIDv3r520827) Start=12 End=290
attggagtgtatgttatagnnnncnnncntncnnttatcctgaaggctttatatatgttgtcaacacgtgggagncaaactgtgaaagaaaacaaagg
gactcgtatccgtttcaaagntgacacttccttcgaggacatcaatggtatcaggannnnannncnnnnnnnnnnttccatgccnactgctcaatc
aactangaaggcngnngnnnnaannnctgggcgatttagaaccatagtatgtggtctatttccaactcaaattcaagtacncanc
(A) CR (235/279): 84.2%
(B)
Max score: 345
Total score: 345
Query coverage: 99%
E value: 4e-91
Identity: 84%

- KY364995.1: Puumala orthohantavirus isolate Ardennes_2011_87 nucleoprotein gene, complete cds.
- KT247593.1: Puumala virus isolate PUUV/Ardennes/Mg75/2011 segment S nucleoprotein gene, complete cds.
- KT247592.1: Puumala virus isolate PUUV/Ardennes/Mg156/2011 segment S nucleoprotein gene, complete cds.

<u>Origin: Germany</u>

<u>PUUV Mu557 Gilserberg/10</u>

>seq223:19_16-09-2014_(VirIDv3r520827) Start=12 End=412
ttngtgntnnnnnnanngggtncncnnnnnnnnnnnnnnnnnnnnnnngnnnnnnncgnngcgncnntgggagacaaacnnnnnnn
nnnnnnnnnnncnncgannnnnnnnnnnngncncnnnntncatttgngnnnnnccntnncngcaggangcnnnnncnnnnnnnnnn
nnnnnnnnnagctgctcaatcaaccatgaaagctgnnnnnnnnnnnnnnnnnnncnnnnnnnnnnnnngnnngnnctatttccaacnc
nnnnncngnntnncgnnataatgagtcctgtgntggnnnngnnnnnnngnnnnnnnnnnnnannnnnnnnngcnnnannnnnnnnn
nnnnnngngnnnnnncnnnnnnnnnnnnnnnnnnnnnnnnnnngnnnnnnnnn
(A) CR (134/401): 33.4%
(B)
Max score: 44.6
Total score: 44.6
Query coverage: 5%
E value: 2.5
Identity: 100%

- MF687747.1: Tula orthohantavirus strain Tul/NL/Ma89-Twente/2007 nucleocapsid protein gene, partial cds.
- MF687746.1: Tula orthohantavirus strain Tul/NL/Ma135-Fr-A&W/2015 nucleocapsid protein gene, partial cds.
- MF687745.1: Tula orthohantavirus strain Tul/NL/Ma3-Fr-Nes/2015 nucleocapsid protein gene, partial cds.
- MF687744.1: Tula orthohantavirus strain Tul/NL/Ma1964-Fr-Feyte/2015 nucleocapsid protein gene, partial cds.
- MF687743.1: Tula orthohantavirus strain Tul/NL/Ma117-Fr-A&W/2015 nucleocapsid protein gene, partial cds.
- MF687742.1: Tula orthohantavirus strain Tul/NL/Ma75-Fr-A&W/2015 nucleocapsid protein gene, partial cds.
- MF687741.1: Tula orthohantavirus strain Tul/NL/Ma126-Fr-A&W/2015 nucleocapsid protein gene, partial cds.
- KU670641.1: Puumala hantavirus isolate Mu07/116 nucleocapsid protein gene, partial cds.
- KU670632.1: Puumala hantavirus isolate KS11/1342 nucleocapsid protein gene, partial cds.
- KU139600.1: Tula virus isolate 08_0277_Marv nucleocapsid protein gene, partial cds.
- KU139599.1: Tula virus isolate 08_0260_Marv nucleocapsid protein gene, partial cds.

- KU139598.1: Tula virus isolate 08_1033_Marv nucleocapsid protein gene, partial cds.
- KU139597.1: Tula virus isolate 10_0215_Marv nucleocapsid protein gene, partial cds.
- KU139558.1: Tula virus isolate D34_Gato_Marv nucleocapsid protein gene, partial cds.
- KU139553.1: Tula virus isolate 09_1477_Marv nucleocapsid protein gene, partial cds.
- KU139531.1: Tula virus isolate 10_0029_Marv nucleocapsid protein gene, partial cds.
- KU139529.1: Tula virus isolate 09_2375_Marv nucleocapsid protein gene, partial cds.
- KU139528.1: Tula virus isolate 09_2324_Marv nucleocapsid protein gene, partial cds.
- **JN696368.1: Puumala virus strain Mu557Gilserberg/10 nucleocapsid protein (N) gene, partial cds**.
- JN696333.1: Puumala virus strain H347Melsungen/10 nucleocapsid protein (N) gene, partial cds.
- GU300143.1: Puumala virus strain Sen05/176 nucleocapsid protein gene, partial cds.
- GU300140.1: Puumala virus strain Sen05/120 nucleocapsid protein gene, partial cds.
- GU300138.1: Puumala virus strain Sen05/107 nucleocapsid protein gene, partial cds.
- EF409820.1: Tula virus strain Brandenburg Mu95/1335 nucleocapsid protein (N) gene, partial cds.
- EF409819.1: Tula virus strain Brandenburg Mu95/1272 nucleocapsid protein (N) gene, partial cds.
- EF409818.1: Tula virus strain Brandenburg Mu95/1049 nucleocapsid protein (N) gene, partial cds.
- EF409817.1: Tula virus strain Brandenburg Mu05/520 nucleocapsid protein (N) gene, partial cds.

>seq230:19_16-09-2014_(VirIDv3r520827) Start=12 End=412
ttggngntgnnnnnnnnncnncnnnnnnncnnnnnnnnnnnnnnnngnnnnnngnnnnngngncntnnnnnnnnnannnnnnn
nnnnnnnnncnnccnncnacnnnnnnnnnnnnncnnncnnngnanntgaggacatcaatggtatcaggangcnnnnncnnnnnnnnn
nnnnnnnnnnanctgctcaatnaaccnngnnancnnnnnnncngnnngnnnnnnnncnnnnnnnnnnnngtgtggtctatttccaact
caaattcaggntcgcancanaatgagtcntgngnntnnnnngnnnnnnnnnnngnnnnnnnnnncnnnnnnnnnnannannnnnn
nnnnntnngnnnnnncnnngnnnnnncnnnnggnnnnnnnnnngnnannncnn
(A) CR (137/401): 34.2%
(B)
Max score: 77.0
Total score: 121
Query coverage: 18%
E value: 4e-10
Identity: 92%
- JN696368.1: Puumala virus strain Mu557Gilserberg/10 nucleocapsid protein (N) gene, partial cds.

>seq233:19_16-09-2014_(VirIDv3r520827) Start=12 End=412
nnnnatntnnnnnnnnnngggttcnnnnannnnnnnnnnnnnnnnannnttatacatgttatcaacccntggnngncaaannnngnnnnnn
nnnnnnnncnnnngtattcgatttaaggncgacacttcatttgaggacancnnnnnnnnnnnnnnnnncnnnnnnnnnnnnnnnnnn
nnnnnnnnnnnnnnnannnannnnngctnnnnnnnngnnngnnnnnnnnngnnnnnnnnnngngnnnnntctatttccaactcaaan
ncngnnnnncnnnanaatgagtcctgtgatggnnnngnnnnnnnnnnnnnnnnnntnnnnnnnnnnnnnnnnnnnnnnnnnnngn
tnatggaanannanngncnnnnnnnnnnnnnncnncnnnngnnnnnnnnn
(A) CR (142/401): 35.4%
(B)
Max score: 62.6
Total score: 153
Query coverage: 29%
E value: 9e-06
Identity: 97%
- JN696368.1: Puumala virus strain Mu557Gilserberg/10 nucleocapsid protein (N) gene, partial cds.

>seq234:19_16-09-2014_(VirIDv3r520827) Start=12 End=238

ncagacattcaggnggatataacccgccannaancgncnnnnnngnttgctngacaaaagctcaaggatgnagnnnnnnnnnngnnanng
gacccagatgacgttaacaaaaacanacnncannnnnnncaacaancagnnngngnnnnnnnnnnnnnnnnnnnnnnnnnnnng
nnnnngnnnnnnnnngngnncnnnnaaaagatggatacnnnnnnnnctga
(A) CR (123/227): 54.2%
(B)
Max score: 80.6
Total score: 80.6
Query coverage: 51%
E value: 2e-11
Identity: 70%
- Select seq KU314905.1: Puumala virus strain PUUV/NL/Mg591/2008 nucleocapsid protein gene, complete cds.
- Select seq DQ094844.1: Puumala virus strain Heidelberg/hu nucleocapsid protein (NP) gene, partial cds.

>seq236:19_16-09-2014_(VirIDv3r520827) Start=12 End=412
nnnnnnnnnngnnnnnnnnnnncnnnnannnnnnnnnnnngncnnnnnntatacatgttatcaacccntggnnnncaaacnnnnnnnn
nnnnnnnnnncnnnngtattcgatttaaggncgacacttcatttgaggnnnnnnnnnnnannnnnnnnnnnnnncnnnnnnnnnnnnn
nnnnnnncnnnnnnnnnanccnnnnnnnncnnnnnncnnnnnnnnnnnnnnnnngnnnnnnnngngnnnnnnnnnatttccaactc
aannncngnnnnnnnnnnnnntgagtcctgtgatggnnnngnnnnnnnnnnnngnnnnntnnnnnnnnnnnnnnnnnnnnnncntn
nnnnngnnnnnnnnncnnnnnnnnnnnnnnnnnnnnnnncnnnngnnnnngnnn
(A) CR (115/401): 28.7%
(B)
Max score: 57.2
Total score: 57.2
Query coverage: 8%
E value: 4e-04
Identity: 97%
- JN696368.1: Puumala virus strain Mu557Gilserberg/10 nucleocapsid protein (N) gene, partial cds.

>seq237:19_16-09-2014_(VirIDv3r520827) Start=12 End=412
tggngntngnnncnnnnncnnnnnnnnnncnnnnnnnnnnnnnnnggnnnnnnnnnnnnnncntnnnnnnnnnannnnngnn
nnnnnnnnnnccnncnnnnnnnnnnncncnnnnnncnnngnanntgaggacatcaatggtancngnnngnnnnnncnnnnnnnn
nnnnnnnnnnagctgctcaatnaaccnngnnancnnnnnnncngnnngnnnnnnnncnnnnnnnnngnnngngtggtctatttccaact
caaattcaggntcgcannanaatgagtcctgngnnnncnnngnnnnnnnnngnngnnnnnnnnnnncnnnnnnnnnnannanncnnn
nnnnntnngnnnnnncnnnnnnnnnncnnnnnngnnnnnnnnnngncannncnnn
(A) CR (130/401): 32.4%
(B)
Max score: 73.4
Total score: 73.4
Query coverage: 11%
E value: 5e-09
Identity: 92%
- JN696368.1: Puumala virus strain Mu557Gilserberg/10 nucleocapsid protein (N) gene, partial cds.

>seq238:19_16-09-2014_(VirIDv3r520827) Start=12 End=290
attggagntnnnnnnnnnnnnncnnnnnnncnnnnnnnnnnnntnnngnnnnnnnnnnnngngncnntgggagacaaactgnnnn
gnngnnnnnnnccnncnacngnnnnnnnnnnnnnnnngnngnanntgaggacatcaatggnnncnnnnnnnnnnnncnnnnnnnn
nngannnnnncanctgctcaatnaaccnngnnancngnnnnnnngnannnnnnnnnnngnnnnnnnngnnngngtggtctatttccaa
ctcaaattcaggntcgcann
(A) CR (115/279): 41.2%
(B)
Max score: 57.2
Total score: 57.2
Query coverage: 11%

E value: 3e-04
Identity: 97%
- JN696368.1: Puumala virus strain Mu557Gilserberg/10 nucleocapsid protein (N) gene, partial cds.

>seq239:19_16-09-2014_(VirIDv3r520827) Start=12 End=290
attggnnntngnnncnnnnncnnnnnnnnnnncnnnnnnnnnnnnnnnggnnnnnnnnnnngngncntnnnnnnnnnannnnnn
nnnnnnnnnnnnccnncnaccgnnnnnnnnnnncnnnnnnngnanntgaggacatcaatggtatcaggnngnnnnnnnnnnnnn
nncnnnnnnnnnanctgctcaatnaaccnngnnancannnnnncngnannnannnnnnnnnnnnnnnnnnngngtggtctatttccaa
ctcaaattcaggntcgcann
(A) CR (106/279): 38.0%
(B)
Max score: 57.2
Total score: 57.2
Query coverage: 11%
E value: 3e-04
Identity: 97%
- JN696368.1: Puumala virus strain Mu557Gilserberg/10 nucleocapsid protein (N) gene, partial cds.

>seq240:19_16-09-2014_(VirIDv3r520827) Start=12 End=290
attggngntngnnnnnnnnncnnnnnnnnnnnnnnnnnnnnnnnnnnnggngnnnncgnngngncnntgggagacaaacnnnnn
ngnnnngnncnnncnnnnnnnnnnnnnnnnncnncncnnnntnnatttgaggacatcaatggtatcaggaggcnnnnncnnnnnnnnnn
nnnnnnnnnagctgctcaatnanccnngnnancnnnnnnncngnanannnnnnnnncnnnnnnnnnnnnngtgtggtctatttccaacnn
nannncngnntngcann
(A) CR (114/279): 40.9%
(B)
Max score: 53.6
Total score: 53.6
Query coverage: 10%
E value: 0.003
Identity: 100%
- KU670632.1: Puumala hantavirus isolate KS11/1342 nucleocapsid protein gene, partial cds.
- JN696368.1: Puumala virus strain Mu557Gilserberg/10 nucleocapsid protein (N) gene, partial cds.
- JN696333.1: Puumala virus strain H347Melsungen/10 nucleocapsid protein (N) gene, partial cds.
- JN696331.1: Puumala virus strain H290Fulda/10 nucleocapsid protein (N) gene, partial cds.
- GU300143.1: Puumala virus strain Sen05/176 nucleocapsid protein gene, partial cds.
- GU300140.1: Puumala virus strain Sen05/120 nucleocapsid protein gene, partial cds.
- GU300138.1: Puumala virus strain Sen05/107 nucleocapsid protein gene, partial cds.

>seq241:19_16-09-2014_(VirIDv3r520827) Start=12 End=290
attggngntngnnnnnnnnngnccnnnntnnnnnnnnnnnnnnnnnnnnggnnnnnnnnnnnnngnccntgggagacaaacnnnnn
nnnnnngnnnnnncnnnnncnnnnnnnnnnnnnncnnncnnngnanttgaggacatcaatggtatcaggnngnnnnnnnnnnnnn
nnnnnnnnnnnagctgctcaatcaaccatgaaancngnnnnnnngnannnnnnnnnnncnnnnnnnnnnnnngtgtggtctatttccaact
caaattcngnctnnnnnn
(A) CR (116/279): 41.6%
(B)
Max score: 48.2
Total score: 90.9
Query coverage: 17%
E value: 0.14
Identity: 100%
- JN696368.1: Puumala virus strain Mu557Gilserberg/10 nucleocapsid protein (N) gene, partial cds.

>seq242:19_16-09-2014_(VirIDv3r520827) Start=12 End=290

nnnncnnnnnnnnnnnnnnnncnncnnnnnnnnnnnnnngnnnnnannnntatacatgttatcaacccgtgggagncaaactgtaaannn
nnnnnnnnncnnagnnnnnnnnnnnnnnnncnncacttcatttgaggacancnnnnnnnnnnnngnnnnnnnncnncnnnnnnnnn
nnnnnnnnnnnncnnnnnnncnnngngnggcnnnnnnnnngnnngnnnnnnncnnnnnnnnnnnnnngnnnnnnnnnnntcna
anncnnnnncngnngnnnngn
(A) CR (86/279): 30.8%
(B)
Max score: 64.4
Total score: 64.4
Query coverage: 13%
E value: 2e-06
Identity: 97%
- JN696368.1: Puumala virus strain Mu557Gilserberg/10 nucleocapsid protein (N) gene, partial cds.
- JN696331.1: Puumala virus strain H290Fulda/10 nucleocapsid protein (N) gene, partial cds.

PUUV Mu2232 Bramsche/09

>seq224:20_16-09-2014_(VirIDv3r520827) Start=12 End=412
ttggagtnnnnnnnnnnnnccnnnnnnngnnnnnnnnnngnnnnnnnngnnnnnnnntatcaacacgtgggagncagngnnngnnn
nnnnnnannnnnnnngnnngnnnnnnnnaggatgatacttcatttgaggatattaatggnatnagnnngnnnnnnnnnntntngtgtccat
gcnannnncnnnannnnnnnnnnnngnnnnnnnnnnnngngnnannnnnnnngnngnnnnnnntggnnnnnnncnnnnncnnctca
nattcannngnnnnnnnnnnngngcnnngnnnnnnnnnnnngnnnnnnngnnnnnnnnnnnnnnnnnnncnnnnnnnnnnn
nncnnnggnnnnnannnnnnnnnnnnnnnnnnncnncnnnnnnnnngncnnnnncn
(A) CR (125/401): 31.2%
(B)
Max score: 60.8
Total score: 60.8
Query coverage: 9%
E value: 4e-05
Identity: 95%
- KU670640.1: Puumala hantavirus isolate KS10/3078 nucleocapsid protein gene, partial cds.
- KU670639.1: Puumala hantavirus isolate KS14/778 nucleocapsid protein gene, partial cds.
- KU670638.1: Puumala hantavirus isolate KS14/766 nucleocapsid protein gene, partial cds.
- KU670637.1: Puumala hantavirus isolate KS14/873 nucleocapsid protein gene, partial cds.
- KU670636.1: Puumala hantavirus isolate KS14/833 nucleocapsid protein gene, partial cds.
- KU670635.1: Puumala hantavirus isolate KS14/715 nucleocapsid protein gene, partial cds.
- KR047313.1: Puumala virus strain varus_12_032 nucleocapsid protein gene, partial cds.
- KR047309.1: Puumala virus strain varus_10_026 nucleocapsid protein gene, partial cds.
- KR047308.1: Puumala virus strain tower_07_001 nucleocapsid protein gene, partial cds.
- KR047300.1: Puumala virus strain schle_12_148 nucleocapsid protein gene, partial cds.
- KR047294.1: Puumala virus strain schle_12_141 nucleocapsid protein gene, partial cds.
- KR047284.1: Puumala virus strain schle_11_124 nucleocapsid protein gene, partial cds.
- KR047281.1: Puumala virus strain schle_11_110 nucleocapsid protein gene, partial cds.
- KR047259.1: Puumala virus strain schle_08_059 nucleocapsid protein gene, partial cds.
- KR047257.1: Puumala virus strain schle_08_053 nucleocapsid protein gene, partial cds.
- KR047252.1: Puumala virus strain schle_05_015 nucleocapsid protein gene, partial cds.
- KR047245.1: Puumala virus strain eller_10_028 nucleocapsid protein gene, partial cds.
- KR047244.1: Puumala virus strain eller_08_025 nucleocapsid protein gene, partial cds.
- KR047228.1: Puumala virus strain astrup_12_067 nucleocapsid protein gene, partial cds.
- KR047225.1: Puumala virus strain astrup_11_062 nucleocapsid protein gene, partial cds.
- KR047207.1: Puumala virus strain astrup_09_042 nucleocapsid protein gene, partial cds.
- KR047202.1: Puumala virus strain astrup_08_033 nucleocapsid protein gene, partial cds.
- KR047199.1: Puumala virus strain astrup_07_017 nucleocapsid protein gene, partial cds.

- KR047195.1: Puumala virus strain astrup_07_005 nucleocapsid protein gene, partial cds.
- KJ994776.1: Puumala virus isolate Mu/07/1219 segment S, complete sequence.
- JN696365.1: Puumala virus strain Mu3072Bramsche/10 nucleocapsid protein (N) gene, partial cds.
- **JN696364.1: Puumala virus strain Mu2232Bramsche/09 nucleocapsid protein (N) gene, partial cds.**
- JN696358.1: Puumala virus strain Mu362Osnabrueck/05 nucleocapsid protein (N) gene, complete cds.
- JN696355.1: Puumala virus strain Mu727Astrup/07 nucleocapsid protein (N) gene, partial cds.
- JN696337.1: Puumala virus strain H362Melle/10 nucleocapsid protein (N) gene, partial cds.
- JN696336.1: Puumala virus strain H368Bad_Iburg/10 nucleocapsid protein (N) gene, partial cds.
- JN696335.1: Puumala virus strain H358Ostercappeln/10 nucleocapsid protein (N) gene, partial cds.
- JN696332.1: Puumala virus strain H389Gramberg/10 nucleocapsid protein (N) gene, partial cds.
- JN696323.1: Puumala virus strain H156Ostercappeln/10 nucleocapsid protein (N) gene, partial cds.

>seq229:20_16-09-2014_(VirIDv3r520827) Start=12 End=412
nnnnngntnnnnnnnnncngctnnnnncntnncncnnnnnnnncnnnnnnngnnnnnnnnancannnnnnangnnnnnnnnnngn
nnnnnnnnnnnnnnngangtattcggtttaaggatgatacttcatttnnnnnnnnnnnnnncnnnnnnnnnnnnnnnnnnccnnnnnngt
ccatgccaactgcncaatcaacnnngaaggctgaagnnnngnanncngnnnnannnnnannnnnnnnnnnnnnnntnnntcccanctcag
nntnnnnnggnnnncnnnnngngcnnngngntgggggttataggctnnnnanncnntgtcaaggattnngcagatcggnntnnnannng
nnngnnnnnncnangncnnnnnnngcnnnnnnnnnnnnnnnnnnnnn
(A) CR (159/401): 39.7%
(B)
Max score: 55.4
Total score: 158
Query coverage: 28%
E value: 0.001
Identity: 89%
- KU670640.1: Puumala hantavirus isolate KS10/3078 nucleocapsid protein gene, partial cds.
- KU670639.1: Puumala hantavirus isolate KS14/778 nucleocapsid protein gene, partial cds.
- KU670638.1: Puumala hantavirus isolate KS14/766 nucleocapsid protein gene, partial cds.
- KU670637.1: Puumala hantavirus isolate KS14/873 nucleocapsid protein gene, partial cds.
- KU670636.1: Puumala hantavirus isolate KS14/833 nucleocapsid protein gene, partial cds.
- KU670635.1: Puumala hantavirus isolate KS14/715 nucleocapsid protein gene, partial cds.

>seq233:20_16-09-2014_(VirIDv3r520827) Start=12 End=412
nnnganntnnnnnnnncggctnnncncntnnnnnnnnnncnnnnnnnnnncnnnnnntatcaacacgtgggagacagactgnnnaan
nnnnnnnnncggnnnnnnnnngnnnnngncngntactnnannnnngngnnnnnnnnnnnnnnnnnnnnnnnnnnnnnnnnn
nngnnnnnnnnnnnnnnnnnnnnnnnnnnngcnnnnnnnnngnnngnnncnnnnngnnnnnntnnnnnnnnnnnntnnnn
nnnnnnncagnttcaagtgcgaaacataatgagcnctgnnnnnnnnnnnnnnnnnnnnnnnnnnnnnnnnnnnnnntgggcagntcgg
nnnnnanngnnnnngnnnnnanaangnnnnnttantnnnnnnnnncnnnnnnnnnnnnn
(A) CR (114/401): 28.4%
(B)
Max score: 50.0
Total score: 94.5
Query coverage: 13%
E value: 0.064
Identity: 94%
- KU670640.1: Puumala hantavirus isolate KS10/3078 nucleocapsid protein gene, partial cds.
- KU670639.1: Puumala hantavirus isolate KS14/778 nucleocapsid protein gene, partial cds.

>seq234:20_16-09-2014_(VirIDv3r520827) Start=12 End=238
nnannnnnnnaanngnanataaccngccatgaacnnnnnnnnnngnnnnncnnnnnnnnnnnnnnnnnnnngnnnnngcagtgg
ngatggnnccngatgacgttnacangnntnnnnnnnannccnnnnnnnaannagtgnnagcattgnnnnacaaacttgcagacnannnnn
nnnnnnngnnnnnnnnngngnnnnnnnncnnacngnnnncnnannnnncnna

(A) CR (93/227): 41.0%
(B)
Max score: 48.2
Total score: 48.2
Query coverage: 39%
E value: 0.12
Identity: 64%

- KJ994776.1: Puumala virus isolate Mu/07/1219 segment S, complete sequence.
- JN696358.1: Puumala virus strain Mu362Osnabrueck/05 nucleocapsid protein (N) gene, complete cds.

>seq242:20_16-09-2014_(VirIDv3r520827) Start=12 End=290
nnnnnannnnnnnnnnnnnncnncnnnnnnnnnnnnnnnnnnnnnnnnnnnnnnnnnnnnntatcaacacgtgggagncagnnngngn
nannaaanaaagggacacgtattcggtttnnggnngntnnnnnnnnnnngnntnnnnnnnnngnnnnngnnnnnnnncnnnnnnnnn
nnnnnnnnnnnnnnncnnnnnnncnnnnngnggcnnnnnnnnnngnngnngntnnncnnagaactanngnnnnnnnnnnnnnn
nnnnncagntncnnnngnnanan
(A) CR (85/279): 30.5%
(B)
Max score: 60.8
Total score: 60.8
Query coverage: 20%
E value: 2e-05
Identity: 80%

- KU670640.1: Puumala hantavirus isolate KS10/3078 nucleocapsid protein gene, partial cds.
- KR047313.1: Puumala virus strain varus_12_032 nucleocapsid protein gene, partial cds.
- KR047309.1: Puumala virus strain varus_10_026 nucleocapsid protein gene, partial cds.
- KR047308.1: Puumala virus strain tower_07_001 nucleocapsid protein gene, partial cds.
- JN696365.1: Puumala virus strain Mu3072Bramsche/10 nucleocapsid protein (N) gene, partial cds.
- **JN696364.1: Puumala virus strain Mu2232Bramsche/09 nucleocapsid protein (N) gene, partial cds.**
- JN696339.1: Puumala virus strain H372Bissendorf/10 nucleocapsid protein (N) gene, partial cds.
- JN696338.1: Puumala virus strain H363Bissendorf/10 nucleocapsid protein (N) gene, partial cds.
- JN696336.1: Puumala virus strain H368Bad_Iburg/10 nucleocapsid protein (N) gene, partial cds.
- JN696334.1: Puumala virus strain H357Bissendorf/10 nucleocapsid protein (N) gene, partial cds.
- JN696322.1: Puumala virus strain H137Bissendorf/10 nucleocapsid protein (N) gene, partial cds.

>seq243:20_16-09-2014_(VirIDv3r520827) Start=12 End=290
attggannntnnnnnnnccnncnncnnncttnncnncnnccnnaaggcncnnnnnnnnnnnnncnnnnnnnnnnnnnnnnnnnnnn
nnnnnnncnnnccngagnnnnnnngnttnngnnnnnnnnnnnnnnnnnnnnggnngnggnngnnnannnnnnnnnnnnngn
nnnnnnncntgccancngntnnatcaactatgaaggctgaagngntgnnnnnnnnnntncgnnnnnnnnnngnnnnnnnnnnnnnn
cnnnnnnnnntnnnnnnnnagnc
(A) CR (84/279): 30.1%
(B)
Max score: 44.6
Total score: 44.6
Query coverage: 13%
E value: 1.7
Identity: 82%

- KU670640.1: Puumala hantavirus isolate KS10/3078 nucleocapsid protein gene, partial cds.
- KU670639.1: Puumala hantavirus isolate KS14/778 nucleocapsid protein gene, partial cds.
- KU670638.1: Puumala hantavirus isolate KS14/766 nucleocapsid protein gene, partial cds.
- KU670637.1: Puumala hantavirus isolate KS14/873 nucleocapsid protein gene, partial cds.
- KU670636.1: Puumala hantavirus isolate KS14/833 nucleocapsid protein gene, partial cds.
- KU670635.1: Puumala hantavirus isolate KS14/715 nucleocapsid protein gene, partial cds.

- KR047313.1: Puumala virus strain varus_12_032 nucleocapsid protein gene, partial cds.
- KR047309.1: Puumala virus strain varus_10_026 nucleocapsid protein gene, partial cds.
- KR047308.1: Puumala virus strain tower_07_001 nucleocapsid protein gene, partial cds.
- KR047300.1: Puumala virus strain schle_12_148 nucleocapsid protein gene, partial cds.
- KR047294.1: Puumala virus strain schle_12_141 nucleocapsid protein gene, partial cds.
- KR047284.1: Puumala virus strain schle_11_124 nucleocapsid protein gene, partial cds.
- KR047281.1: Puumala virus strain schle_11_110 nucleocapsid protein gene, partial cds.
- KR047259.1: Puumala virus strain schle_08_059 nucleocapsid protein gene, partial cds.
- KR047257.1: Puumala virus strain schle_08_053 nucleocapsid protein gene, partial cds.
- KR047252.1: Puumala virus strain schle_05_015 nucleocapsid protein gene, partial cds.
- KR047246.1: Puumala virus strain eller_10_029 nucleocapsid protein gene, partial cds.
- KR047245.1: Puumala virus strain eller_10_028 nucleocapsid protein gene, partial cds.
- KR047244.1: Puumala virus strain eller_08_025 nucleocapsid protein gene, partial cds.
- KR047228.1: Puumala virus strain astrup_12_067 nucleocapsid protein gene, partial cds.
- KR047225.1: Puumala virus strain astrup_11_062 nucleocapsid protein gene, partial cds.
- KR047207.1: Puumala virus strain astrup_09_042 nucleocapsid protein gene, partial cds.
- KR047202.1: Puumala virus strain astrup_08_033 nucleocapsid protein gene, partial cds.
- KR047199.1: Puumala virus strain astrup_07_017 nucleocapsid protein gene, partial cds.
- KR047195.1: Puumala virus strain astrup_07_005 nucleocapsid protein gene, partial cds.
- KJ994776.1: Puumala virus isolate Mu/07/1219 segment S, complete sequence.
- JN696365.1: Puumala virus strain Mu3072Bramsche/10 nucleocapsid protein (N) gene, partial cds.
- **JN696364.1: Puumala virus strain Mu2232Bramsche/09 nucleocapsid protein (N) gene, partial cds.**
- JN696358.1: Puumala virus strain Mu362Osnabrueck/05 nucleocapsid protein (N) gene, complete cds.
- JN696355.1: Puumala virus strain Mu727Astrup/07 nucleocapsid protein (N) gene, partial cds.
- JN696340.1: Puumala virus strain H373Osnabrueck/10 nucleocapsid protein (N) gene, partial cds.
- JN696339.1: Puumala virus strain H372Bissendorf/10 nucleocapsid protein (N) gene, partial cds.
- JN696338.1: Puumala virus strain H363Bissendorf/10 nucleocapsid protein (N) gene, partial cds.
- JN696337.1: Puumala virus strain H362Melle/10 nucleocapsid protein (N) gene, partial cds.
- JN696336.1: Puumala virus strain H368Bad_Iburg/10 nucleocapsid protein (N) gene, partial cds.
- JN696335.1: Puumala virus strain H358Ostercappeln/10 nucleocapsid protein (N) gene, partial cds.
- JN696334.1: Puumala virus strain H357Bissendorf/10 nucleocapsid protein (N) gene, partial cds.
- JN696332.1: Puumala virus strain H389Gramberg/10 nucleocapsid protein (N) gene, partial cds.
- JN696323.1: Puumala virus strain H156Ostercappeln/10 nucleocapsid protein (N) gene, partial cds.
- JN696322.1: Puumala virus strain H137Bissendorf/10 nucleocapsid protein (N) gene, partial cds.
- JN696321.1: Puumala virus strain H126Muenster/10 nucleocapsid protein (N) gene, partial cds.
- JN696317.1: Puumala virus strain H400Samern/10 nucleocapsid protein (N) gene, partial cds.

PUUV Mu978 Weissach/10

>seq222:21_16-09-2014_(VirIDv3r520827) Start=12 End=412
gnngngnnggnnnnnccgnanncncacttcctattnnnnnnnnnnnnnnnnnnnnnnnnnnnngnccntgggagacaaactgtganggn
aaacaaagggactcgtatccggtttaaagatgncacttntttcgaggacattaatggcnggnnnnnnnnnnnncnnnnnnnnnnnnncnnnc
cgnctgctcaatcnaccnngnnnncngnnnnnnngnnnnnnnnnnnncnnnnnnnnnnnnnnnnnnnnnnnnnnnnnnnnncagatt
caagnacgcaatnnnnnnannnnnggnnntncnnnnnnnnncnnnngnnnnnnnngnnngcnnnnnncnnnnnnnnnnggggan
ngnnnnnnnnnnnnntnnnnnttcatcannnnnnnnnnnnngncannncnn
(A) CR (170/401): 42.4%
(B)
Max score: 129
Total score: 129
Query coverage: 20%
E value: 8e-26

Identity: 94%

- KU670631.1: Puumala hantavirus isolate Mu10/1001 nucleocapsid protein gene, partial cds.
- **JN696370.1: Puumala virus strain Mu978Weissach/10 nucleocapsid protein (N) gene, partial cds.**
- JN696360.1 : Puumala virus strain Mu1054Heimerdingen2/10 nucleocapsid protein (N) gene, partial cds.
- EU085562.1: Puumala virus strain Weissach31 nucleocapsid protein gene, partial cds.
- EU085558.1: Puumala virus strain Hemmingen13 nucleocapsid protein gene, partial cds.
- EU085559.1: Puumala virus strain Weissach20 nucleocapsid protein gene, partial cds.

>seq224:21_16-09-2014_(VirIDv3r520827) Start=12 End=412
ttggggttnnnnnnnnnnncnncnnnnnnnnnnnntnnnngnnnannnnnnnnncntgttatcaacccgtgggagncaannnnngnngna
aaacaaaggnactcgtatccggtttaaagatgncngnggnnncnnggacattaatggtnnncnannnccaaagcatttgtatgtgtccatgcngn
nnnnnnnnnnnnncnnngnnnncnnnnnnnnngngnnnnnncnggttcagnnnnnnnnntngtnnnncnnncncgncncagattcaa
gtacgcaatatantgngtnctgnccnnncnnnnngnnncnnngnnnnnnnnnngnnncnnnnnncnnnnnnnnnngnnnnnnnnn
nnnnnnnnnannnnntttcatcaaaccagaanncnngnnnnnncnn
(A) CR (195/401): 48.6%
(B)
Max score: 123
Total score: 172
Query coverage: 40%
E value: 4e-24
Identity: 78%

- KU670631.1: Puumala hantavirus isolate Mu10/1001 nucleocapsid protein gene, partial cds.
- **JN696370.1: Puumala virus strain Mu978Weissach/10 nucleocapsid protein (N) gene, partial cds.**
- JN696360.1: Puumala virus strain Mu1054Heimerdingen2/10 nucleocapsid protein (N) gene, partial cds.

>seq234:21_16-09-2014_(VirIDv3r520827) Start=12 End=238
acagacattcaggnggatataaccngccatgaacanncnnnngttgttgccagncaaangctcaaggatgnagnnnnnncnnngnnanngg
acccagatgacgttaacaaaancanacnncangnaaggcaacngncagtgnnagctnnnnnnnnnnnnnnnnnnnnnnncnnnnnnnn
nangncnnnnnnngngnnncnnnanaagatggannnnnaannggcnna
(A) CR (135/227): 59.5%
(B)
Max score: 132
Total score: 132
Query coverage: 64%
E value: 4e-27
Identity: 75%

- DQ094844.1: Puumala virus strain Heidelberg/hu nucleocapsid protein (NP) gene, partial cds.

>seq240:21_16-09-2014_(VirIDv3r520827) Start=12 End=290
ngnngnnnnngnnnnnncnnanncncacttcctattntntngnnnnnnnnnnnnnnncnnngngncngtgggagacaaactgtgnngg
naaacaaagggactcgtatccggtttaaagatgncnctnnttncnnnnnnngtnnnnncngnngnnnnnnnnnnnnnnnnnnnnnnn
nngccgnctgctcaannnnncnnnnnnncannnnnnnngnannnnnnnnnncnnnnnnnnngnnnnnnngnnnannnnnnggnn
cagattcaagtacgcaat
(A) CR (127/279): 45.5%
(B)
Max score: 89.7
Total score: 89.7
Query coverage: 21%
E value: 4e-14
Identity: 92%

- KU670631.1: Puumala hantavirus isolate Mu10/1001 nucleocapsid protein gene, partial cds.
- **JN696370.1: Puumala virus strain Mu978Weissach/10 nucleocapsid protein (N) gene, partial cds.**
- JN696360.1: Puumala virus strain Mu1054Heimerdingen2/10 nucleocapsid protein (N) gene, partial cds.
- EU085562.1: Puumala virus strain Weissach31 nucleocapsid protein gene, partial cds.
- EU085558.1: Puumala virus strain Hemmingen13 nucleocapsid protein gene, partial cds.
- EU085559.1: Puumala virus strain Weissach20 nucleocapsid protein gene, partial cds.

>seq241:21_16-09-2014_(VirIDv3r520827) Start=12 End=290
ngnngngnnngnnnnnnnnnnncnnnnnnnnnnnnnnnnnnnnnnnnngnnnnnnnnnnnngncnnngggagacaaactgtg
nnggnaaacaaagggactcgtatcnggtttaaagnnnncacttntttcgagnncantnnnnncnggnnnnnnnnnnnncnnnnnnnnnn
nnnnnnncgnctgctcaatcaaccannnnnncngnnnnnnngnannnnnnnnnncnnnnnnnnnnnnnngngnnnnnnnnnng
nnnngnttcaagtacgcaat
(A) CR (112/279): 40.1%
(B)
Max score: 87.8
Total score: 87.8
Query coverage: 23%
E value: 2e-13
Identity: 86%

- KU670631.1: Puumala hantavirus isolate Mu10/1001 nucleocapsid protein gene, partial cds.
- **JN696370.1: Puumala virus strain Mu978Weissach/10 nucleocapsid protein (N) gene, partial cds.**
- JN696360.1: Puumala virus strain Mu1054Heimerdingen2/10 nucleocapsid protein (N) gene, partial cds.
- EU085562.1: Puumala virus strain Weissach31 nucleocapsid protein gene, partial cds.
- EU085558.1: Puumala virus strain Hemmingen13 nucleocapsid protein gene, partial cds.
- EU085559.1: Puumala virus strain Weissach20 nucleocapsid protein gene, partial cds.

>seq242:21_16-09-2014_(VirIDv3r520827) Start=12 End=290
nntgggnntnnnnnnnncnnnnncnnnnntnnnnnnnnnnnnnnnnnnncgnncntgtnatcaacccgtgggagacaaactgngnnn
nnnnncnnnnncnntnnnatccggtttaaagatgacactnntnncnnnnnnantaatggtattcgnngnccaaagcatttntnngngnncnnn
nnnnnnncnnnnnnnnnnnnnnnggcnnnannnnnggnngnnnnnnnccnnnnngnnnntnnnnnnnnnnnnnnnnnnnnnn
ggnncnnnnnnncnan
(A) CR (110/279): 39.4%
(B)
Max score: 75.2
Total score: 75.2
Query coverage: 40%
E value: 1e-09
Identity: 69%

- KU670631.1: Puumala hantavirus isolate Mu10/1001 nucleocapsid protein gene, partial cds.
- **JN696370.1: Puumala virus strain Mu978Weissach/10 nucleocapsid protein (N) gene, partial cds.**
- JN696360.1: Puumala virus strain Mu1054Heimerdingen2/10 nucleocapsid protein (N) gene, partial cds.
- EU085562.1: Puumala virus strain Weissach31 nucleocapsid protein gene, partial cds.
- EU085558.1: Puumala virus strain Hemmingen13 nucleocapsid protein gene, partial cds.
- EU085559.1: Puumala virus strain Weissach20 nucleocapsid protein gene, partial cds.

Origin: Finland

PUUV laboratory strain Sotkamo

>seq222:PUUV_Finland_cells_(VirIDv3r520827) Start=12 End=412
ttggagtgtatgtgntngggttcacacttcctnncnnnnnnnnnnnnnnnnnnnnnnnnnnnnnnngnnngggannnngnnnnnnnnnn
nnnnnnnnncnnnnncnnnnnnnnnnnnncnnnnnnnnnnannnnnnnnnnnnnnnncngnnnnnnnnnnnnnnnnnnnnn
nnntctatgncngcnnccccgnnannnnnnnnannnnnnnnnnnnnnnnnnnnnnnnnnnnnnnnnnnnnnnnnnnnnnnnnnn
nnnnnancnnnnnnnncngnnnnnnnngnnannnnnnngnnnnngntcattgnnnnnnnnnnnnnnnngnnncnnnnngcnncn
nnnnnnnnnnnnnnnatggaaaaagagtgcnnannncnnnnnnnnnnnnnnnngnnnnnnnnn
(A) CR (96/401): 23.9%
(B)
Max score: 51.8
Total score: 51.8
Query coverage: 7%
E value: 0.017
Identity: 94%
- HE801633.1: Puumala virus N and Ns genes, segment S, genomic RNA, strain Sotkamo 2009.
- X61035.1: Puumala virus genomic RNA for nucleocapsid protein.

>seq225:PUUV_Finland_cells_(VirIDv3r520827) Start=12 End=412
ttggagtgtatgtgatagggttcacacttcctatcatccttaaagctttatacatgctctcaacgcnngnnagacagactgtaaaggaaaataaggga
cacgtataaggtttaaggatgatacatcatttgaagacatcaatggcataaggagaccaaagcatttatatgtttctatgccnactgcccagtcaacnnt
gaaagcagaagaactcacaccnngcagatttcgcacaatagtatgtggtctttttcccactcagatccaggttcgnaacatcatgagtccagttatgn
nggtcattggtttttcattctttgtgaaggattggtctgagagaatcagagagttcatggaaaaagagtgcccattcataaagcctgaagnaaaaccng
gcaca
(A) CR (387/401): 96.5%
(B)
Max score: 673
Total score: 673
Query coverage: 100%
E value: 0.0
Identity: 97%
- HE801633.1: Puumala virus N and Ns genes, segment S, genomic RNA, strain Sotkamo 2009.
- X61035.1: Puumala virus genomic RNA for nucleocapsid protein.

>seq227:PUUV_Finland_cells_(VirIDv3r520827) Start=12 End=412
ttggagtgtatgtgannnnnnncnnnnnnnnnnnnnnnnnnnnnnnnnnnnnnnnnncnnccnnanngnnnnnnnnnnngnnnnngnn
nnnnnnngngnncnnnnnnnnnnngnnnnnnnnnnntnnannnnnnnaagacatcanngncanangnannnnnnnngnnnnnnn
nnntnnnnnncctactgcccagtcaactatgaaagcagnannanncgnnnnannnnaannncncacaatagtannnnnnngnnnnnnnn
nnnncnnnnnnnnnnnnnnnnnncnnnnnnccagttnnnnnnnnnnnnnnnnnnnnnnnnnnnnnnnnnnnnnnnnnnnnnngnnnn
nngnnnnnnnncnnggnnnnanngnnnnnnnncnnnnnnnntnaannagnnnnanncnnn
(A) CR (116/401): 28.9%
(B)
Max score: 51.8
Total score: 51.8
Query coverage: 6%
E value: 0.017
Identity: 100%
- HE801633.1: Puumala virus N and Ns genes, segment S, genomic RNA, strain Sotkamo 2009.
- X61035.1: Puumala virus genomic RNA for nucleocapsid protein.

>seq228:PUUV_Finland_cells_(VirIDv3r520827) Start=12 End=412

ttggnnnggnnnnnnnnnnnnnnnnnnnnnnnnnnnnnnnncnnnnnnnnnnnnnnnnnnnnnnnnnnnnnnnnnnnnnnnnnnnnnn
nnnnnnnnnnnnnnncnnnnnnnnnnnngtttaaggatgatacatcatttgaagacatcaatggcannnncnnnnnnnnncnntnnnnnnnt
nnnnnnnnngctgcncagncanctannnnnncnnnnnnnnannnnnnnnnccnnnnnnnnnnnngnnnnnnnnnnnnnntttcccnn
nnnnngcnnnnncannngnnncnnnnnnnnnnnntnnnncnnnnnnnnnnnnnnnnnnnnnnnngnnnganannnnntnnnnnnn
nnnnnnnnnnnnngnnnnnnnnnnnnnnnnnnnnnnnnnnnnnnnncnnnnnnnnncccnnn
(A) CR (91/401): 22.7%
(B)
Max score: 69.8
Total score: 69.8
Query coverage: 9%
E value: 6e-08
Identity: 100%
- **HE801633.1: Puumala virus N and Ns genes, segment S, genomic RNA, strain Sotkamo 2009.**
- AJ314597.1: Puumala virus mRNA for nucleocapsid protein (N gene), strain Pallasjarvi/63Cg/98.
- Z30705.1: Puumala virus gene for N protein, genomic RNA, strain Evo/15Cg/93.
- Z30704.1: Puumala virus gene for N protein, genomic RNA, strain Evo/14Cg/93.
- Z30702.1: Puumala virus gene for N protein, genomic RNA, strain Evo/12Cg/93.
- X61035.1: Puumala virus genomic RNA for nucleocapsid protein.

>seq229:PUUV_Finland_cells_(VirIDv3r520827) Start=12 End=412
nnnnnnnggnnnnnnnnnnnnnncnnnnnnnnnnnnnnnnnnnnnnnnnnnnnnnnnnnnnngnnnnnnnngnnnngnnnnn
nnnnnnnnnnnnnnncnnnnnnnnnnnggtttaaggatgatacancatttnnagacannngnnncngnnnnnnnnnannccnnnnnnn
nnnnnnnnnnnnncnnnncnngnnnnnnnnnnnnannanngannnnnnnnnnnnnnnnncnnnnnnnnnnnnngngnnnnnnnn
nnnnnnnncancnnncgncnnnnnnnnnnnnnngnnnnnnnntnncnnnnnnnnnnnnnntnnnnnnnnnngnnnnnnnnnncnn
nnnnnnnnnnnnngntcatggaaaaagagtncnnnnnnnnnnnnnnannnnnnnnnncanncnca
(A) CR (85/401): 21.2%
(B)
Max score: 44.6
Total score: 44.6
Query coverage: 7%
E value: 2.5
Identity: 90%
- JQ319163.2: Puumala virus strain PUUV/Konnevesi/Mg_M94A/2005 segment S, complete sequence.
- JQ319162.2: Puumala virus strain PUUV/Konnevesi/Mg_O78A/2005 segment S, complete sequence.
- JQ319161.2: Puumala virus strain PUUV/Konnevesi/Mg_O57A/2005 segment S, complete sequence.
- JN831943.1: Puumala virus strain PUUV/Pieksamaki/Mg7/2008 segment S, complete sequence.
- **HE801633.1: Puumala virus N and Ns genes, segment S, genomic RNA, strain Sotkamo 2009.**
- AJ314597.1: Puumala virus mRNA for nucleocapsid protein (N gene), strain Pallasjarvi/63Cg/98.
- Z30705.1: Puumala virus gene for N protein, genomic RNA, strain Evo/15Cg/93.
- Z30704.1: Puumala virus gene for N protein, genomic RNA, strain Evo/14Cg/93.
- Z30702.1: Puumala virus gene for N protein, genomic RNA, strain Evo/12Cg/93.
- X61035.1: Puumala virus genomic RNA for nucleocapsid protein.

>seq230:PUUV_Finland_cells_(VirIDv3r520827) Start=12 End=412
ttggagtgtatgtgntngggttcacacttccnnncnnnnnnnnnnnnnnnnnnnnnnnnnnnnngcgtgggagacagacnnnnnnnnnn
nnnnnnnncnnnnnnngnnnnnnnngnnnnnnnnnngnnnnnnnnnnnngnnnngncanaagnnnnncnnnncattnnnnnnnn
nctatgccnnnnncccngnnnnnnnnnnnnncnnnnnnnnnnnnnnnnnnnnnnncngnnnnnnnnnnnnngtggtcntnnnnnnn
nnnnnnnnnnnnccgnnnnnnnngnnannnnnngnnntnngntcattgnnntnnnnnnnnnnnnnnnnncnnnnngcnncnnnnnn
nnnnnnnttcatggaaaaagagtgnnnannncnnnnnnnnnnnnnnnngnnnncnnnn
(A) CR (122/401): 30.4%
(B)

Max score: 50.0
Total score: 50.0
Query coverage: 7%
E value: 0.064
Identity: 94%

- HE801633.1: Puumala virus N and Ns genes, segment S, genomic RNA, strain Sotkamo 2009.
- X61035.1: Puumala virus genomic RNA for nucleocapsid protein.

>seq231:PUUV_Finland_cells_(VirIDv3r520827) Start=12 End=412
nnnnnnngnnnnnnnnnnnngnncnnnnagnntnncgnnnnnnnagctttnnnnnnnncncnnnangnnngggngacagactgtaaag
gaaaanaagggnacacgtataaggtttaaggatgatacatcatttgaagacatcaatggcataaggngaccaaagcatttatatgtttctatgcctact
gcccagtcaactangnnancagaagaactcacaccaggcagatttcgcncaatagtatgtggtcnttnncncnnnnnnnnnnncgnntngtaac
atcatgagtccagttatgnnnnncnnnnntnnnnnnnnncnnnnngnnnnattggtctgagagaatcagagagttcatggaaaaagagtgccca
ttcnnaaagcnnnnntnngnngnnnnnnga
(A) CR (281/401): 70.1%
(B)
Max score: 361
Total score: 361
Query coverage: 78%
E value: 8e-96
Identity: 82%

- HE801633.1: Puumala virus N and Ns genes, segment S, genomic RNA, strain Sotkamo 2009.

>seq232:PUUV_Finland_cells_(VirIDv3r520827) Start=12 End=412
nnggnntgngngnnnnnnngnncnnnnnnnnnnnnnnnnnnnnnnnnnnnnnnnnnnnnnnnnnnnnnnnnnnnnagacngnn
nnnnnnnnnnnnnncnnnnnnnnnnnnnnnnnnnnnnntacancatttgaagacatcaatggcatanggagaccnaagcatnnnnnnn
ntnnnnnnnnnnnnncncnnncnactatgaaagcaannnannncnnnnnnnnnnnnnnntnncncnnnnnnnnnnnntnnnnnnnn
nnnnnnnnnnnnnnnnnnnnnncnngagtccagttatggnggtcantgntnnnnnnnnnnnnnngnnnnnnnnnnnagnnnnnnn
nnnnnnnnnnnnnnnnnnnnnnnnnnnnnnnncnnngnctnnnnnnnnnncanncnnn
(A) CR (110/401): 27.4%
(B)
Max score: 68.0
Total score: 68.0
Query coverage: 10%
E value: 2e-07
Identity: 93%

- **HE801633.1: Puumala virus N and Ns genes, segment S, genomic RNA, strain Sotkamo 2009.**
- AJ314597.1: Puumala virus mRNA for nucleocapsid protein (N gene), strain Pallasjarvi/63Cg/98.
- AJ238789.1: Puumala virus RNA for nucleocapsid protein, strain Kolodozero.
- AJ238788.1: Puumala virus RNA for nucleocapsid protein, strain Karhumaki.
- X61035.1: Puumala virus genomic RNA for nucleocapsid protein.

>seq233:PUUV_Finland_cells_(VirIDv3r520827) Start=12 End=412
nnnnannnnnnnnnnnnnnnnnncnnnnnnnnnnnnnnnnnnnnnnnnnnnnnnnnnnncncnnnnngcgtgggagacagactgnaa
aggaaaataannnnngnnnnnnnnnnnnngnnnnnnntnnagnnnnncnannnnncnnnnnnnnanggnnnnnnnnnnnnnnn
nnnnnnnnnnnnnnncnnnnncngnnnnnnnnnnnncnnnnnnnnnnnnnnnnnntnnnnnnnnnnnngnngnnnnnntnntn
nnnnnnnnncnnnnnnnnnnnnnnnnncnnnnnngnngntnnnnnnnnnnnnnnnnnnnnnnnnnnngnnnnnnnnnnn
nnnnnnnnnnnnnnnnnnnnnnnnnnnnnnnnnnnnnnnnnnnnnnnncncnnnnnnnnncnnn
(A) CR (62/401): 15.5%
(B)
Max score: 51.8
Total score: 51.8
Query coverage: 7%
E value: 0.018

Identity: 97%

- **HE801633.1: Puumala virus N and Ns genes, segment S, genomic RNA, strain Sotkamo 2009.**
- AJ238790.1: Puumala virus RNA for nucleocapsid protein, strain Gomselga.
- X61035.1: Puumala virus genomic RNA for nucleocapsid protein.

>seq234:PUUV_Finland_cells_(VirIDv3r520827) Start=12 End=238
acannnnnnnnnncgnntataacccgccatgnannnannnnnannnttgccagnnananannnnnnnnnnnnnnnnnnnnnngnnn
nnggacccagatgacgttaatannnnnanncnncannncnnnnnncaaacagtgncagcacnggaggacaaactcgcnnngnncnnnnn
gnnnntggcagntgctgggnccnannnnnnanngnnnnnnnnnnnnactga
(A) CR (114/227): 50.2%
(B)
Max score: 64.4
Total score: 64.4
Query coverage: 33%
E value: 2e-06
Identity: 73%

- HE801633.1: Puumala virus N and Ns genes, segment S, genomic RNA, strain Sotkamo. 2009.
- X61035.1: Puumala virus genomic RNA for nucleocapsid protein.

>seq236:PUUV_Finland_cells_(VirIDv3r520827) Start=12 End=412
nnnnnnnnnnnnnnnnnnnnnnnnnnnatnnnnnnnngnnnnnnnnnnnnnnnnnnncnnnnngcgtgggagacagactgtaaa
ggaaaataannnnnnnannnnnnnnnnngnnncnnnnnnngnnnnnnnnnnnnnnnnnnnnnnnngtnncnnnnnnnnnnnn
nnnnnnnnnnnnncnncnncnnnngnnnnnnngncnnnnnnnnnnnnnnnnnnnnnnnnnnnnnnnnannnntatgtggtctttttcc
cnnnnnnnnnnnnnnnnnnnnnnnnnnnnnngnnnnnnnnnnnnnnnnnnnnnnnnnngncnnnnngnnnnnnnnnnntnnnn
nnnnnnnnnnnnnnnnnnnnnnnnnnnnnngnnnnnnnnnnnnnnnnngcnnnnnanccnnn
(A) CR (76/401): 18.9%
(B)
Max score: 55.4
Total score: 55.4
Query coverage: 7%
E value: 0.001
Identity: 100%

- **HE801633.1: Puumala virus N and Ns genes, segment S, genomic RNA, strain Sotkamo 2009.**
- AJ238790.1: Puumala virus RNA for nucleocapsid protein, strain Gomselga.
- X61035.1: Puumala virus genomic RNA for nucleocapsid protein.

>seq237:PUUV_Finland_cells_(VirIDv3r520827) Start=12 End=412
tggagtgtatgngntngggttcacacttcctnncnnnnnnnnnnnnnnnnnnnnnnnnnnnnnncgtgggagacagacnnnnnnnnnn
gnnntnccnnnnnnnnnnnnnnnccnnncnnnnnngnnnnnnnngnnngnnnnggcananggannncnncgcattnnnnnnnnnc
tatnncnncnncccngnnnnnnnnnnnnnnnnnnnnnnnnnnnnnnnngnnnncngnnnnnnnnnnnntgtggncntnnnnnnnnn
nnnnnnnnnnccgnnnnnanngngnnnnnnnggnnnnngntcantgnnnnnnnnnnnnnnnnnnnncnnnnngcnncnnnnnnn
nnnnnnttcatggaaaaagagnncnnnnnnnnnnnnnnnnnnnnnnngnnancnnnnn
(A) CR (125/401): 31.2%
(B)
Max score: 46.4
Total score: 46.4
Query coverage: 7%
E value: 0.77
Identity: 90%

- HE801633.1: Puumala virus N and Ns genes, segment S, genomic RNA, strain Sotkamo 2009.
- X61035.1: Puumala virus genomic RNA for nucleocapsid protein.

>seq239:PUUV_Finland_cells_(VirIDv3r520827) Start=12 End=290

attggagtgtatgtgntngggttcacacttccnancnnnnnnnnnnnnnnnnnnnnnnnnnnnnnnnngcgtgggagacagacnnnnnnnnn
nnnnnntnncnnngnnngnnnnnnnngnannnnnnnngnnannnnnnnnngnnnngncananannnnnnnnnnngnnnnnnnnn
nnnctatgccnncnnccccgnnnnnnnnnnnannnnnnnnnnnnnnnnnnnnnnnnnnnnnnnnnnnnnnnnngtggncttnnnnn
nnnncnnnnncanncngnnnnn

(A) CR (87/279): 31.2%

(B)

Max score: 51.8
Total score: 51.8
Query coverage: 11%
E value: 0.012
Identity: 94%

- HE801633.1: Puumala virus N and Ns genes, segment S, genomic RNA, strain Sotkamo 2009.
- X61035.1: Puumala virus genomic RNA for nucleocapsid protein.

>seq240:PUUV_Finland_cells_(VirIDv3r520827) Start=12 End=290
attggagtgtatgngnnngggttcacacttccnancnnnnnnnnnnnnnnnnnnnnnnnnnnnnnngnnnggnagnnngnnnnnnnnn
nnnnnnnctnnnnnnnncnnnnnnnnnncnncnnnnnnngnnnnnncannnnnnnnnggcnnaaggnnnccnnngcantngnnn
nnnnctatgncnncnncccngnnnnnnnnnnnannnnnnnnnnnnnnnnnnnnnnnncnnnnnnnnnnnnnntgtggtcnttntccn
nnnnnnnnnnnncnnnnnnn

(A) CR (84/279): 30.1%

(B)

Max score: 44.6
Total score: 44.6
Query coverage: 11%
E value: 1.8
Identity: 88%

- HE801633.1: Puumala virus N and Ns genes, segment S, genomic RNA, strain Sotkamo 2009.
- X61035.1: Puumala virus genomic RNA for nucleocapsid protein.

>seq242:PUUV_Finland_cells_(VirIDv3r520827) Start=12 End=290
nnnnnnnnnnnnnnnnnnnnnnnnnnnntnnnnncnnnnnnnnnnnnnnnnnnnncncnnnncgnnngggagacagnctgta
aaggaaaataaggggacangtannnnnnnnnnnnnnnnnnnnngnnnnnnnnnnnnnnnnnnnnnnnangnnannnnacnnnnnnn
nnnnnnnnnnnnnnnnncncnnccaactatgannnnnnnnnnnnnnnnngnnnnnnnnnnngnngnnnnnnnnnnnnnnntn
gnnnnnnnncnnnnnnnnnnnnnnn

(A) CR (65/279): 23.3%

(B)

Max score: 60.8
Total score: 60.8
Query coverage: 13%
E value: 2e-05
Identity: 95%

- **HE801633.1: Puumala virus N and Ns genes, segment S, genomic RNA, strain Sotkamo 2009.**
- AJ238790.1: Puumala virus RNA for nucleocapsid protein, strain Gomselga.
- X61035.1: Puumala virus genomic RNA for nucleocapsid protein.

<u>**PUUV 335 Konnevesi (variant A)**</u>

>seq225:PUUV_Finland_335_(VirIDv3r520827) Start=12 End=412
ntngngttnnnntgntagggttcacacttcctatcatccttnannncnnnnnnnnnctatcaacgcgtggnagnnnannnnngnnnnnnnnn
nnannnacncgtataaggttnnagnntnanacancattngaanacantantgnnnnnaggaggncannnnnnnnntncgnnnnnnngnc
nnnngnnnnnnnnnnncnnnnngnnnnnagnanncannnnnnnnncnnnnnncacaatagtatgtgnnnnannnnnannnnnnnnn
naanttcntaacatcatgagtccagttatgnnnnnnnnnnnnnnnnnnnntnnnnnnnnnnnnnnnngnnnnnnnnntagngagtnnnn
ggnnnngnnnnnnnnnnnnncnnnnnngnnnntnanccaggcaca

(A) CR (178/401): 44.4%
(B)
Max score: 66.2
Total score: 112
Query coverage: 43%
E value: 8e-07
Identity: 62%

- JQ319162.2: Puumala virus strain PUUV/Konnevesi/Mg_O78A/2005 segment S, complete sequence.

>seq231:PUUV_Finland_335_(VirIDv3r520827) Start=12 End=412
ttggnnnnnnnnngnnnnnnnnnnnnnngnnnnncnnnnnnanagccttatacntncannnnnnnnnnnnnnnnnnnnnnnnnn
nnnnnnnnnnncnnnnntnnnaggtttnagnntgatacatcatttgaagacnntannnnnnnnaggaggccannnnnnnnnnncnnnnn
nnnnnnngnngcccagtcaaccatgaaggcagaagnactcacannnnncnngnnnnnnnnnnnagtntntngtctatttccaacnnagnnc
nnnnntngtaacatcatgagtccagttatgnnnnnnnnnnntnnnnnnnnnnnnngnnnnnnnnncnnnnnnnnnntngngagtncn
nggnnnngnnnnnnnnnnnnnnnnnnnnnnnnnnnnnnnnnnn
(A) CR (155/401): 38.6%
(B)
Max score: 80.6
Total score: 127
Query coverage: 40%
E value: 4e-11
Identity: 70%

- **JQ319162.2: Puumala virus strain PUUV/Konnevesi/Mg_O78A/2005 segment S, complete sequence.**
- JN831943.1: Puumala virus strain PUUV/Pieksamaki/Mg7/2008 segment S, complete sequence.

>seq234:PUUV_Finland_335_(VirIDv3r520827) Start=12 End=238
nnnnnnnnnnnnncnnnnnnaannnnnnnnnnnnnnnngnnnnnngttgccagannnnnnnnnnnnnnnnnnnnnnnnnnnnnn
nnnnnggacccagatgacnttaacaaanacacacnncannncnnnnnnnnannnnnnngnnnnnnnnnnnnnnncnnnnnnnnnc
ncnnnngnnnnngnnnnnnnnnnngnnnnnnnnnnnannnnnnnnnnnnanngnctga
(A) CR (58/227): 25.5%
(B)
Max score: 46.4
Total score: 46.4
Query coverage: 12%
E value: 0.41
Identity: 93%

- KY365007.1: Puumala orthohantavirus isolate Septmoncel-Alsace_2015_Camp5 nucleoprotein gene, complete cds.
- KY365006.1: Puumala orthohantavirus isolate Poligny-Jura_2015_C05 nucleoprotein gene, complete cds.
- KY365005.1: Puumala orthohantavirus isolate Poligny-Jura_2015_B18 nucleoprotein gene, complete cds.
- KY365004.1: Puumala orthohantavirus isolate Orleans_2014_NCHA373 nucleoprotein gene, complete cds.
- KY364999.1: Puumala orthohantavirus isolate MontsousVaudrey-Jura_2014_NCHA14 nucleoprotein gene, complete cds.
- KY364998.1: Puumala orthohantavirus isolate ChauxdesCrotenay_Jura_2014_NCHA71 nucleoprotein gene, complete cds.
- KY364997.1: Puumala orthohantavirus isolate ChauxdesCrotenay-Jura_2014_CI11 nucleoprotein gene, complete cds.
- KY364996.1: Puumala orthohantavirus isolate Chaource/Troyes_2008_RP2 nucleoprotein gene, complete cds.

- KY364995.1: Puumala orthohantavirus isolate Ardennes_2011_87 nucleoprotein gene, complete cds.
- KT247596.2: Puumala virus isolate PUUV/Jura/Mg2/2010 segment S nucleoprotein gene, complete cds.
- KU314905.1: Puumala virus strain PUUV/NL/Mg591/2008 nucleocapsid protein gene, complete cds.
- KU314901.1: Puumala virus strain PUUV/NL/Mg1439/2011 nucleocapsid protein gene, complete cds.
- KU314900.1: Puumala virus strain PUUV/NL/Mg1012/2010 nucleocapsid protein gene, complete cds.
- KU314899.1: Puumala virus strain PUUV/NL/Mg753/2009 nucleocapsid protein gene, complete cds.
- KU314898.1: Puumala virus strain PUUV/NL/Mg343/2008 nucleocapsid protein gene, complete cds.
- KU314897.1: Puumala virus strain PUUV/NL/Mg25/2007 nucleocapsid protein gene, complete cds.
- KU314896.1: Puumala virus strain PUUV/NL/Mg31/2007 nucleocapsid protein gene, complete cds.
- KT247597.1: Puumala virus isolate PUUV/Jura/Mg214/2010 segment S nucleoprotein gene, complete cds.
- KT247595.1: Puumala virus isolate PUUV/Orleans/Mg29/2010 segment S nucleoprotein gene, complete cds.
- KT247594.1: Puumala virus isolate PUUV/Orleans/Mg23/2010 segment S nucleoprotein gene, complete cds.
- KT247593.1: Puumala virus isolate PUUV/Ardennes/Mg75/2011 segment S nucleoprotein gene, complete cds.
- KT247592.1: Puumala virus isolate PUUV/Ardennes/Mg156/2011 segment S nucleoprotein gene, complete cds.
- JQ319163.2: Puumala virus strain PUUV/Konnevesi/Mg_M94A/2005 segment S, complete sequence.
- **JQ319162.2: Puumala virus strain PUUV/Konnevesi/Mg_O78A/2005 segment S, complete sequence.**
- JQ319161.2: Puumala virus strain PUUV/Konnevesi/Mg_O57A/2005 segment S, complete sequence.
- JQ319171.1: Puumala virus strain PUUV/Konnevesi/Mg_M114B/2005 segment S, complete sequence.
- JQ319167.1: Puumala virus strain PUUV/Konnevesi/Mg_O15B/2005 segment S, complete sequence.
- JN831943.1: Puumala virus strain PUUV/Pieksamaki/Mg7/2008 segment S, complete sequence.
- JN696373.1: Puumala virus strain MuEb10Karlstadt/10 nucleocapsid protein (N) gene, complete cds.
- JN696372.1: Puumala virus strain MuEb4Karlstadt/10 nucleocapsid protein (N) gene, complete cds.
- JN696371.1: Puumala virus strain MuEb6Karlstadt/10 nucleocapsid protein (N) gene, complete cds.
- GQ339479.1: Puumala virus strain Moskosel/Mg17/05 segment S, complete sequence.
- AM695638.1: Puumala virus S gene for nucleocapsid protein, strain PUU/Mignovillard/CgY02/2005, genomic RNA.
- EF488806.1: Puumala virus isolate Fusong 900-06 nucleocapsid gene, complete cds.
- EF488804.1: Puumala virus isolate Fusong 114-05 nucleocapsid gene, complete cds.
- EF488803.1: Puumala virus isolate Fusong 199-05 nucleocapsid gene, complete cds.
- EF442091.1: Puumala-like virus isolate Fusong-Cr-275 segment S nucleocapsid protein gene, complete cds.

- EF211824.1: Puumala virus isolate Fusong 114-05 nucleocapsid mRNA, partial cds.
- EF211823.1: Puumala virus isolate Fusong 199-05 nucleocapsid mRNA, partial cds.
- EF211821.1: Puumala virus isolate Fusong 99-05 nucleocapsid mRNA, partial cds.
- EF211820.1: Puumala virus isolate Fusong 200-05 nucleocapsid mRNA, complete cds.
- EF211819.1: Puumala virus isolate Fusong 84-05 nucleocapsid mRNA, complete cds.
- AF411447.1: Puumala virus isolate CG142 nucleocapsid protein N gene, partial cds.
- AF294652.1: Puumala virus segment S nucleocapsid protein gene, complete cds.
- DQ094844.1: Puumala virus strain Heidelberg/hu nucleocapsid protein (NP) gene, partial cds.
- AJ223369.1: Puumala virus RNA, strain Puu/Eidsvoll/Cg1138/87.
- AJ223368.1: Puumala virus RNA, strain Puu/Eidsvoll/1124v.
- Z46942.1: Puumala virus segment S, genomic RNA, strain Puu/Puu/1324Cg/79.

>seq242:PUUV_Finland_335_(VirIDv3r520827) Start=12 End=290
nnnnnnnnnnnnnnnnnnnnnnncnnnnnnnnnnnnnnnnnnnnnnnnnnnnnnnnncnnnnnncgcgtgggagacaaactgng
nngnaaaataaagggacannnnnnnnnnnnnnnnnnnnnnnnnnnnnnnnnnnnnnnnnnnnnnnnnnnnnnnnnnnnnnnnnnnnnn
nnnnnnnnnnnnnnnnnnnnnnnnnnnnnnnnnnnngnnnnnnnnnnnnnnnnnnnnnnnnnnnnnnnnnnnnnnnnnngnnnnnn
nnnnnnnnnnnnnnnnnnnnnnnnnnnnnn
(A) CR (38/279): 13.6%
(B)
Max score: 55.4
Total score: 55.4
Query coverage: 13%
E value: 0.001
Identity: 89%

- JQ319163.2: Puumala virus strain PUUV/Konnevesi/Mg_M94A/2005 segment S, complete sequence.
- **JQ319162.2: Puumala virus strain PUUV/Konnevesi/Mg_O78A/2005 segment S, complete sequence.**
- JQ319161.2: Puumala virus strain PUUV/Konnevesi/Mg_O57A/2005 segment S, complete sequence.
- JN831943.1: Puumala virus strain PUUV/Pieksamaki/Mg7/2008 segment S, complete sequence.
- Z46942.1: Puumala virus segment S, genomic RNA, strain Puu/Puu/1324Cg/79.

Origin: Slovenia

PUUV Slovenia 8098

>seq225:PUUV_Slovenia-8098_03-05-2013_(VirIDv3r520827) Start=12 End=412
ttggnnntnnnnnnnnnnnnnnnnnnnngnnnnnnnnnnnnnnnnnnnnnnnnnnnnnnnnnnntnnnnnnnnnnnnnnnnnna
nannnnnnnnnnnnnnnnnnnnnnnnnnnnnnnntgatacatcatttgnggacatcanngncannnnannaccaaagcatttannnnnn
nnnnnnnnnnnannnnnnnnnncanngnngnnnnnnnnnnnnnnnnnnnnnncnngnnncnnnnnnnnnnnnnnnnncnnnnn
nnnnnnnnnnnnnnnnnnnnnnnnnnnnnnnnnnnngnnnnnnnnnnnnnnnnnnnnnngnnnnnnnnnnnnnnnnngannn
nnnggnnnnnnnngnnngnnnnnnnnnnnnnnnnnnnnnnnnnnnnnnnnnnnnnnnnnnnn
(A) CR (65/401): 16.2%
(B)
Max score: 53.6
Total score: 53.6
Query coverage: 12%
E value: 0.005
Identity: 80%

- KF776903.1: Puumala virus isolate HtSi_339_p2012 nucleocapsid protein gene, partial cds.
- KF776902.1: Puumala virus isolate HtSi_293_p2010 nucleocapsid protein gene, partial cds.
- KF776901.1: Puumala virus isolate HtSi_283_p2009 nucleocapsid protein gene, partial cds.

- KF776898.1: Puumala virus isolate HtSi_254_p2008 nucleocapsid protein gene, partial cds.
- KF776897.1: Puumala virus isolate HtSi_252_p2008 nucleocapsid protein gene, partial cds.
- KF776896.1: Puumala virus isolate HtSi_250_p2008 nucleocapsid protein gene, partial cds.
- KF776895.1: Puumala virus isolate HtSi_248_p2008 nucleocapsid protein gene, partial cds.
- KF776894.1: Puumala virus isolate HtSi_247_p2008 nucleocapsid protein gene, partial cds.
- KF776893.1: Puumala virus isolate HtSi_246_p2008 nucleocapsid protein gene, partial cds.
- KF776892.1: Puumala virus isolate HtSi_235_p2007 nonfunctional nucleocapsid protein gene, partial sequence.
- KF776891.1: Puumala virus isolate HtSi_199_p2005 nucleocapsid protein gene, partial cds.
- KF776890.1: Puumala virus isolate HtSi_142_p2002 nucleocapsid protein gene, partial cds.
- KF776880.1: Puumala virus isolate HtSi_1068_a1998 nucleocapsid protein gene, partial cds.
- KF776879.1: Puumala virus isolate HtSi_1063_a1999 nucleocapsid protein gene, partial cds.
- KF776876.1: Puumala virus isolate HtSi_363_p2012 nucleocapsid protein gene, partial cds.
- KF776873.1: Puumala virus isolate HtSi_1069_a1999 nucleocapsid protein gene, partial cds.
- KF776866.1: Puumala virus isolate HtSi_434_p2012 nucleocapsid protein gene, partial cds.
- KF776865.1: Puumala virus isolate HtSi_424_p2012 nucleocapsid protein gene, partial cds.
- KF776860.1: Puumala virus isolate HtSi_410_p2012 nucleocapsid protein gene, partial cds.
- KF776857.1: Puumala virus isolate HtSi_377_p2012 nucleocapsid protein gene, partial cds.
- KF776856.1: Puumala virus isolate HtSi_374_p2012 nucleocapsid protein gene, partial cds.
- KF776855.1: Puumala virus isolate HtSi_335_p2012 nucleocapsid protein gene, partial cds.
- KC676615.1: Puumala virus strain PUUV/Croatia_Gerovo/Mg982/2008 nucleocapsid protein (N) mRNA, partial cds.
- KC676614.1: Puumala virus strain PUUV/Croatia_Gerovo/Mg980/2008 nucleocapsid protein (N) mRNA, partial cds.
- KC676613.1: Puumala virus strain PUUV/Croatia_Gerovo/Mg979/2008 nucleocapsid protein (N) mRNA, partial cds.
- KC676612.1: Puumala virus strain PUUV/Croatia_Gerovo/Mg978/2008 nucleocapsid protein (N) mRNA, partial cds.
- KC676611.1: Puumala virus strain PUUV/Croatia_Gerovo/Mg955/2008 nucleocapsid protein (N) mRNA, partial cds.
- KC676610.1: Puumala virus strain PUUV/Croatia_Gerovo/Mg954/2008 nucleocapsid protein (N) mRNA, partial cds.
- KC676609.1: Puumala virus strain PUUV/Croatia_Gerovo/Mg938/2008 nucleocapsid protein (N) mRNA, partial cds.
- JF499661.1: Puumala virus strain PUUcro97rd nucleocapsid protein gene, partial cds.
- JF499660.1: Puumala virus strain PUUcro62ps nucleocapsid protein gene, partial cds
- JF499659.1: Puumala virus strain PUUcro39vd nucleocapsid protein gene, partial cds.
- FN377822.1: Puumala virus N gene for nucleocapsid protein, strain PUUV/Mg23/HungaryTR17/00.
- AJ314601.1: Puumala virus mRNA for nucleocapsid protein (N gene), strain Balkan-2.
- AJ888738.1: Puumala virus partial mRNA for nucleocapsid protein (N gene), PUU/Slovenia-Pat7.
- AJ888736.1: Puumala virus partial mRNA for nucleocapsid protein (N gene), PUU/Slovenia-Pat3.
- AJ888734.1: Puumala virus partial mRNA for nucleocapsid protein (N gene), PUU/Slovenia-Cg4.

>seq226:PUUV_Slovenia-8098_03-05-2013_(VirIDv3r520827) Start=12 End=412
nnnnnnnnnnnnnnnnnnnnnnnnnnnnnnnnnnnnnnnnnnnnnnnnnnnnnnnnnnnnnnnnnnnaggnagncagactgtna
aagaaaataaaggnannnnnngnnnnnnnnnnnnnnnnnnnnnnnnnnnnnnnnnnnnnnnnnnngnnnnnnnnnncnnnnntnnn
nngtntcnntgncannannnnnnnnnnnnnnnnnnnnnnnnnnnnnnnngnngnnnnnnnnnnnnnnnnngncaattnnnnnnnnn
nnnnnnnnnnnnnnnnnnnnnnnnnnnnnncnnnnnnnnnnnnnnnnnnnnnnnnnnnnnnnnnnnnnnnnnnnnnnnnnnnnnnn
nnnnnnnnnnnnnnnnnnnnnnnnnnnnnnngnnnnnnnnnnnnnnnnnnnnnnnnnnnnnnnnnnn
(A) CR (51/401): 12.7%
(B)
Max score: 44.6

Total score: 44.6
Query coverage: 7%
E value: 2.7
Identity: 90%

- KF776902.1: Puumala virus isolate HtSi_293_p2010 nucleocapsid protein gene, partial cds.
- KF776901.1: Puumala virus isolate HtSi_283_p2009 nucleocapsid protein gene, partial cds.
- KF776898.1: Puumala virus isolate HtSi_254_p2008 nucleocapsid protein gene, partial cds.
- KF776891.1: Puumala virus isolate HtSi_199_p2005 nucleocapsid protein gene, partial cds.
- KF776885.1: Puumala virus isolate HtSi_1071_a1999 nucleocapsid protein gene, partial cds.
- KF776880.1: Puumala virus isolate HtSi_1068_a1998 nucleocapsid protein gene, partial cds.
- KF776879.1: Puumala virus isolate HtSi_1063_a1999 nucleocapsid protein gene, partial cds.
- KF776873.1: Puumala virus isolate HtSi_1069_a1999 nucleocapsid protein gene, partial cds.
- KF776868.1: Puumala virus isolate HtSi_483_p2012 nucleocapsid protein gene, partial cds.
- KF776855.1: Puumala virus isolate HtSi_335_p2012 nucleocapsid protein gene, partial cds.
- JF499663.1: Puumala virus strain PUUcroMP nucleocapsid protein gene, partial cds.
- JF499661.1: Puumala virus strain PUUcro97rd nucleocapsid protein gene, partial cds. .
- JF499660.1: Puumala virus strain PUUcro62ps nucleocapsid protein gene, partial cds.
- JF499659.1: Puumala virus strain PUUcro39vd nucleocapsid protein gene, partial cds.
- FN377822.1: Puumala virus N gene for nucleocapsid protein, strain PUUV/Mg23/HungaryTR17/00.
- AJ314600.1: Puumala virus mRNA for nucleocapsid protein (N gene), strain Balkan-1.
- AJ888738.1: Puumala virus partial mRNA for nucleocapsid protein (N gene), PUU/Slovenia-Pat7.
- AJ888734.1: Puumala virus partial mRNA for nucleocapsid protein (N gene), PUU/Slovenia-Cg4.
- AJ888733.1: Puumala virus partial mRNA for nucleocapsid protein (N gene), PUU/Slovenia-Cg3.

>seq228:PUUV_Slovenia-8098_03-05-2013_(VirIDv3r520827) Start=12 End=412
ttggagtttatntcantggntncncnnnnnnnnnnnnnnnnnnnnnnnnnnnntnnnntatcaacacgaggaagncngnnnnnnnnnnna
nnnnnnnnnnnnnncnnnnntttnnaggntgatacatcatttgnggncatcaatggcatnngnnnncnnnnnnatttatntgtatccatgccaa
cagnccagncaacaatgaaggcagangnnnnnnnnnnnnnccnnnnnnnnnnnnnngnannnnnnnnncnnnnnnnnnncaaattcag
gtncgcaatnttangagtcctgngntgggggttataggattctntntttncntaaaggattggtcagagaggnngnnngnnnnnntggagaaaga
atgcccttttataannnnnnnnnnnnnnnnnnnnnnnn
(A) CR (224/401): 55.9%
(B)
Max score: 167
Total score: 167
Query coverage: 67%
E value: 3e-37
Identity: 67%

- KF776902.1: Puumala virus isolate HtSi_293_p2010 nucleocapsid protein gene, partial cds.

>seq231:PUUV_Slovenia-8098_03-05-2013_(VirIDv3r520827) Start=12 End=412
ttggannnnnnnncnnnnncnnnnnnnngnnnnnnnnnnnnnnnnnncnnnnnnnnnnnnnnnnnnnaggaagacagacngngn
nnnnnnnnnnnncnnnnnnnnnncctttnnnggatgatacatcatttgnggacatcannggnantnnannaccaaagcatttatatgtannnn
nnnnancagnnnnnncaacaatgnagncnnnnnnnnnnnnnnnnnnncnnnnnnnnnnnnnnnnnnnnnnnnnnncnnnnnnnnnn
nnnnnnnnnnnnnnnnnnnnnnnnnnnnnnnnnnnnnnnnnnnnnnnnnnngnnnncnnnaaggattggtcagnnnnnngnn
nnnnnngnnnnnnnnnnnnnnnnnnnnnnnnnnnnnnnnnnannnnnnnnnnnnnnn
(A) CR (113/401): 28.2%
(B)
Max score: 73.4
Total score: 73.4
Query coverage: 14%
E value: 6e-09
Identity: 84%

- KF776903.1: Puumala virus isolate HtSi_339_p2012 nucleocapsid protein gene, partial cds.

- KF776902.1: Puumala virus isolate HtSi_293_p2010 nucleocapsid protein gene, partial cds.
- KF776901.1: Puumala virus isolate HtSi_283_p2009 nucleocapsid protein gene, partial cds.
- KF776897.1: Puumala virus isolate HtSi_252_p2008 nucleocapsid protein gene, partial cds.
- KF776896.1: Puumala virus isolate HtSi_250_p2008 nucleocapsid protein gene, partial cds.
- KF776895.1: Puumala virus isolate HtSi_248_p2008 nucleocapsid protein gene, partial cds.
- KF776894.1: Puumala virus isolate HtSi_247_p2008 nucleocapsid protein gene, partial cds.
- KF776893.1: Puumala virus isolate HtSi_246_p2008 nucleocapsid protein gene, partial cds.
- KF776892.1: Puumala virus isolate HtSi_235_p2007 nonfunctional nucleocapsid protein gene, partial sequence.
- KF776891.1: Puumala virus isolate HtSi_199_p2005 nucleocapsid protein gene, partial cds.
- KF776890.1: Puumala virus isolate HtSi_142_p2002 nucleocapsid protein gene, partial cds.
- KF776880.1: Puumala virus isolate HtSi_1068_a1998 nucleocapsid protein gene, partial cds.
- KF776879.1: Puumala virus isolate HtSi_1063_a1999 nucleocapsid protein gene, partial cds.
- KF776876.1: Puumala virus isolate HtSi_363_p2012 nucleocapsid protein gene, partial cds.
- KF776873.1: Puumala virus isolate HtSi_1069_a1999 nucleocapsid protein gene, partial cds.
- KF776866.1: Puumala virus isolate HtSi_434_p2012 nucleocapsid protein gene, partial cds.
- KF776865.1: Puumala virus isolate HtSi_424_p2012 nucleocapsid protein gene, partial cds.
- KF776860.1: Puumala virus isolate HtSi_410_p2012 nucleocapsid protein gene, partial cds.
- KF776857.1: Puumala virus isolate HtSi_377_p2012 nucleocapsid protein gene, partial cds.
- KF776856.1: Puumala virus isolate HtSi_374_p2012 nucleocapsid protein gene, partial cds.
- KF776855.1: Puumala virus isolate HtSi_335_p2012 nucleocapsid protein gene, partial cds.
- KC676615.1: Puumala virus strain PUUV/Croatia_Gerovo/Mg982/2008 nucleocapsid protein (N) mRNA, partial cds.
- KC676614.1: Puumala virus strain PUUV/Croatia_Gerovo/Mg980/2008 nucleocapsid protein (N) mRNA, partial cds.
- KC676613.1: Puumala virus strain PUUV/Croatia_Gerovo/Mg979/2008 nucleocapsid protein (N) mRNA, partial cds.
- KC676612.1: Puumala virus strain PUUV/Croatia_Gerovo/Mg978/2008 nucleocapsid protein (N) mRNA, partial cds.
- KC676611.1: Puumala virus strain PUUV/Croatia_Gerovo/Mg955/2008 nucleocapsid protein (N) mRNA, partial cds.
- KC676610.1: Puumala virus strain PUUV/Croatia_Gerovo/Mg954/2008 nucleocapsid protein (N) mRNA, partial cds.
- KC676609.1: Puumala virus strain PUUV/Croatia_Gerovo/Mg938/2008 nucleocapsid protein (N) mRNA, partial cds.
- JF499661.1: Puumala virus strain PUUcro97rd nucleocapsid protein gene, partial cds .
- JF499660.1: Puumala virus strain PUUcro62ps nucleocapsid protein gene, partial cds.
- JF499659.1: Puumala virus strain PUUcro39vd nucleocapsid protein gene, partial cds.
- FN377822.1: Puumala virus N gene for nucleocapsid protein, strain PUUV/Mg23/HungaryTR17/00.
- AJ314601.1: Puumala virus mRNA for nucleocapsid protein (N gene), strain Balkan-2.
- AJ888738.1: Puumala virus partial mRNA for nucleocapsid protein (N gene), PUU/Slovenia-Pat7.
- AJ888736.1: Puumala virus partial mRNA for nucleocapsid protein (N gene), PUU/Slovenia-Pat3.
- AJ888734.1: Puumala virus partial mRNA for nucleocapsid protein (N gene), PUU/Slovenia-Cg4.

>seq233:PUUV_Slovenia-8098_03-05-2013_(VirIDv3r520827) Start=12 End=412
nnnnnnntnnnnnnnnntnncnncnnnnnnnnnnnnnnnnnnnnnnnnnnnnnnnnnnnnnacnannangnnnnnnnnnn
nnannnnnnnnanncnnnnnnnnnnnntnnnnnnnnnnntnnannnnnnnnaggacancaatggcattagaagaccanancatnnannn
nnnnccanncnancnncnncnnnnnnnnnnnnngcnnnnnnncnngnngnnnnnnnnnnnnnnnnnnnnnngnnnnnnnnnn
nnnnnnncaaattcngnnnnnnnnnnnnnnngtnctgtgnnnnnnnnnnnnnnnnnnnnnnnnnnntnnnnnnnnnnnnnnnn
nnnnnnnnnnnnnnnnnnnnnnnnnnnnnnnntnttntaannnnnnnnnnnnnngnnnnnnnnn
(A) CR (82/401): 20.4%
(B)

Max score: 48.2
Total score: 48.2
Query coverage: 7%
E value: 0.22
Identity: 91%

- KF776902.1: Puumala virus isolate HtSi_293_p2010 nucleocapsid protein gene, partial cds.
- KF776901.1: Puumala virus isolate HtSi_283_p2009 nucleocapsid protein gene, partial cds.
- KF776900.1: Puumala virus isolate HtSi_269_p2008 nucleocapsid protein gene, partial cds.
- KF776899.1: Puumala virus isolate HtSi_262_p2008 nucleocapsid protein gene, partial cds.
- KF776898.1: Puumala virus isolate HtSi_254_p2008 nucleocapsid protein gene, partial cds.
- KF776896.1: Puumala virus isolate HtSi_250_p2008 nucleocapsid protein gene, partial cds.
- KF776895.1: Puumala virus isolate HtSi_248_p2008 nucleocapsid protein gene, partial cds.
- KF776892.1: Puumala virus isolate HtSi_235_p2007 nonfunctional nucleocapsid protein gene, partial sequence.
- KF776891.1: Puumala virus isolate HtSi_199_p2005 nucleocapsid protein gene, partial cds.
- KF776890.1: Puumala virus isolate HtSi_142_p2002 nucleocapsid protein gene, partial cds.
- KF776884.1: Puumala virus isolate HtSi_1079_a1995 nucleocapsid protein gene, partial cds.
- KF776881.1: Puumala virus isolate HtSi_1058_a1995 nucleocapsid protein gene, partial cds.
- KF776880.1: Puumala virus isolate HtSi_1068_a1998 nucleocapsid protein gene, partial cds.
- KF776879.1: Puumala virus isolate HtSi_1063_a1999 nucleocapsid protein gene, partial cds.
- KF776876.1: Puumala virus isolate HtSi_363_p2012 nucleocapsid protein gene, partial cds.
- KF776873.1: Puumala virus isolate HtSi_1069_a1999 nucleocapsid protein gene, partial cds.
- KF776871.1: Puumala virus isolate HtSi_317_p2012 nucleocapsid protein gene, partial cds.
- KF776869.1: Puumala virus isolate HtSi_485_p2012 nucleocapsid protein gene, partial cds.
- KF776866.1: Puumala virus isolate HtSi_434_p2012 nucleocapsid protein gene, partial cds.
- KF776864.1: Puumala virus isolate HtSi_1054_a1995 nucleocapsid protein gene, partial cds.
- KF776860.1: Puumala virus isolate HtSi_410_p2012 nucleocapsid protein gene, partial cds.
- KF776859.1: Puumala virus isolate HtSi_402_p2012 nucleocapsid protein gene, partial cds.
- KF776857.1: Puumala virus isolate HtSi_377_p2012 nucleocapsid protein gene, partial cds.
- KF776856.1: Puumala virus isolate HtSi_374_p2012 nucleocapsid protein gene, partial cds.
- KF776855.1: Puumala virus isolate HtSi_335_p2012 nucleocapsid protein gene, partial cds.
- KC676615.1: Puumala virus strain PUUV/Croatia_Gerovo/Mg982/2008 nucleocapsid protein (N) mRNA, partial cds.
- KC676614.1: Puumala virus strain PUUV/Croatia_Gerovo/Mg980/2008 nucleocapsid protein (N) mRNA, partial cds.
- KC676612.1: Puumala virus strain PUUV/Croatia_Gerovo/Mg978/2008 nucleocapsid protein (N) mRNA, partial cds.
- KC676611.1: Puumala virus strain PUUV/Croatia_Gerovo/Mg955/2008 nucleocapsid protein (N) mRNA, partial cds.
- KC676609.1: Puumala virus strain PUUV/Croatia_Gerovo/Mg938/2008 nucleocapsid protein (N) mRNA, partial cds.
- JF499661.1: Puumala virus strain PUUcro97rd nucleocapsid protein gene, partial cds .
- JF499660.1: Puumala virus strain PUUcro62ps nucleocapsid protein gene, partial cds.
- JF499659.1: Puumala virus strain PUUcro39vd nucleocapsid protein gene, partial cds.
- FN377822.1: Puumala virus N gene for nucleocapsid protein, strain PUUV/Mg23/HungaryTR17/00.
- AJ314601.1: Puumala virus mRNA for nucleocapsid protein (N gene), strain Balkan-2.
- AJ888739.1: Puumala virus partial mRNA for nucleocapsid protein (N gene), PUU/Slovenia-Pat5.
- AJ888738.1: Puumala virus partial mRNA for nucleocapsid protein (N gene), PUU/Slovenia-Pat7.
- AJ888736.1: Puumala virus partial mRNA for nucleocapsid protein (N gene), PUU/Slovenia-Pat3.
- AJ888735.1: Puumala virus partial mRNA for nucleocapsid protein (N gene), PUU/Slovenia-Pat1.
- AJ888734.1: Puumala virus partial mRNA for nucleocapsid protein (N gene), PUU/Slovenia-Cg4.

- AJ888731.1: Puumala virus partial mRNA for nucleocapsid protein (N gene), PUU/Slovenia-Cg1.

>seq234:PUUV_Slovenia-8098_03-05-2013_(VirIDv3r520827) Start=12 End=238
ncnnnnnnnnnnnnnnnnataacccnncannaanngnnnnntgttgttgccagncngnnncnnnnnnnnnnnanngnnnncnnnggna
atggacccagatgacgttaacannnntnnncngcaagcaaggcaacaancagtgtcagcattggaggacaaacncgcannnnctnnnnnnn
nnnngncnnnnnnngngnccnnnaaaaagatggatacnnancctactga
(A) CR (133/227): 58.6%
(B)
Max score: 109
Total score: 109
Query coverage: 36%
E value: 4e-20
Identity: 87%
- FN377822.1: Puumala virus N gene for nucleocapsid protein, strain PUUV/Mg23/HungaryTR17/00.
- FN377821.1: Puumala virus N gene for nucleocapsid protein, strain PUUV/Mg9/HungaryTR17/00.
- AJ314601.1: Puumala virus mRNA for nucleocapsid protein (N gene), strain Balkan-2.
- AJ888752.1: Puumala virus mRNA for nucleocapsid protein (N gene), PUU/Ernstbrunn/Cg641/1995.
- AJ888751.1: Puumala virus mRNA for nucleocapsid protein (N gene), PUU/Klippitztoerl/Cg9/1995.

>seq242:PUUV_Slovenia-8098_03-05-2013_(VirIDv3r520827) Start=12 End=290
nnnnnnnnnnnnnnnnntgnctncacactccngnncnnnnnnnnnnnnnnnnnnnnncnnnnnnnannannnnnnnnnnnnnnnn
nnnnnnnnnnnnnnnngnnnnnnnnnnnnnnnnnnnnnnnnnnnnnnnannnnnncnnnnncnnnannagaccanagcannnn
nnnnnnnnnnnnnaacnncncnncnnnnnnnnnnnncngnnnnnnnnnnnnnnnnnnnnnnnnnnnnnnnnnnntgtggtctctt
cccaactcaaattcagnnnnnnnnn
(A) CR (67/279): 24.0%
(B)
Max score: 50.0
Total score: 50.0
Query coverage: 9%
E value: 0.042
Identity: 100%
- KF776902.1: Puumala virus isolate HtSi_293_p2010 nucleocapsid protein gene, partial cds.
- KF776901.1: Puumala virus isolate HtSi_283_p2009 nucleocapsid protein gene, partial cds.
- KF776898.1: Puumala virus isolate HtSi_254_p2008 nucleocapsid protein gene, partial cds.
- KF776885.1: Puumala virus isolate HtSi_1071_a1999 nucleocapsid protein gene, partial cds.
- KF776880.1: Puumala virus isolate HtSi_1068_a1998 nucleocapsid protein gene, partial cds.
- FN377821.1: Puumala virus N gene for nucleocapsid protein, strain PUUV/Mg9/HungaryTR17/00.
- AJ888734.1: Puumala virus partial mRNA for nucleocapsid protein (N gene), PUU/Slovenia-Cg4.

Viral species: Dobrava virus (DOBV)

Origin: Finland

DOBV laboratory strain

>seq245:3_08-08-2013_(VirIDv3r520827) Start=12 End=412
nnntngnccgcctgncatcattcgtgnntnnnnnntnnnngnaggctctttacatgcttaccaccagaggnagncaaacnactnaanacnataa
gggnnngngnnnncnatttaaggatgacagcnctttgaagatgtgaatgggannannaagccanancacctgttcttgtcaatgccnaatgcac
natcnagcnngnnggcagntgagattncaccaggtcggntcnggnctgcaanttgtggactatannnagnncaggngnnnnnnngnnacn
nnnncngtcctgtgntgagtgtgnttngnttttntgnnnnnnnnnngnnnnnnnnanngnngncngngnnatngcnngnnctnccgtgcaa
gctactatctgagccatcnccaacnnctntg
(A) CR (284/401): 70.8%
(B)
Max score: 293
Total score: 293
Query coverage: 97%
E value: 3e-75
Identification: 71%

- AJ410615.1: Dobrava virus complete S segment gene for nucleocapsid protein, strain DOBV/Ano-Poroia/Afl9/1999.

SAAV laboratory strain

>seq245:4_08-08-2013_(VirIDv3r520827) Start=12 End=412
ttgtgatcngnnnnncnnntnnnnnanngnnnnnnnnccngnnggctctttncnnnnnnnnnnnnnnnnnncnngcaaactactaaagac
aataagggaatgaggattcgatttaaggatgacagtnnnnntgngnnngngnnnggannnnnnnnannnnnncntnnnntnttgtcgatgc
nannnnnnnnnnnnnnnnnnnnancagatgagattacacctgnnanncnnnnnnnnnnnnnnnnnnggntnatacccagcccaggttaaa
gcangnnnnnngnnnngngnngncnnnnnnnngnnnnnnnnnnntgnnnnnnnnnnnnnnnnnnnngnnnntnncngnggggnn
gcnngnnctcnntnnnnnnnnntnnncnnnancnnnnnnnnnannnnna
(A) CR (177/401): 44.1%
(B)
Max score: 95.1
Total score: 137
Query coverage: 19%
E value: 2e-15
Identity: 100%

- AJ009773.1: Dobrava virus S segment gene encoding nucleocapsid protein, strain DOB/Saaremaa/160V), genomic RNA.

>seq247:4_08-08-2013_(VirIDv3r520827) Start=12 End=412
nnnnganctattnnnnnnnnnncnnannnncaatactcctgaaggctctttncntgttgacaactagagggaggcanacnnnnnnnnnng
nnnnnnncnnnnnnngnnanntnnnnnnnnngnnngnnnnnngnnnnnnnnnnnnngnnnnnnnnnnnnnnnnnnncnnnn
ggnnnccnnnnncccagtccngcatgaaagcagatgagattacanctnnnnnncngnnannngcngtctgtggattatncnnanccnannn
ncnngnnnngnnnnnnnnnnncncngncatgagtgtgattggnnncncnannnnnnnnnnnnnnttggacgnaacgggttgaggagtgg
cgnnncnncnnnnncaagctattatctnaannnnntnnnnnnnnnnna
(A) CR (194/401): 48.4%
(B)
Max score: 78.8
Total score: 145
Query coverage: 61%
E value: 1e-10
Identity: 94%

- AJ009773.1: Dobrava virus S segment gene encoding nucleocapsid protein, strain DOB/Saaremaa/160V), genomic RNA.

>seq256:4_08-08-2013_(VirIDv3r520827) Start=12 End=412
nnnnnnnnnnnnnnnnnnnnnnnncnnnnnnngnnnnnnnnnnnnnnnnnnnnnnnnnnnnnnnntnnnnnnnnnnannnnnnn
nnnnnnngnnntnccnnnnnnnnnnnnnnttaaggatgacagttcttttgaggatgngnnngnanntnnanaancannnncnnnnnnnnn
ncnnnnnnnancnnnnnngngcnnnnnnnnancagntnnnnngnnnnnnnnnnnnnnnanngnnnnntnnnnnnnnnnnnnnnn
nnnnnnnnnnnnnnnnnnnnnnnnnnnnngnnnnnncnngnnngngnnnnntnnnnnnnnnnngnnnnnnnnnnnnnnnn
nnntngnnnnnnnnnnnnnnnnnnntnnnnnnnnnnnnnnnnnnnnnnnnnnnnnnncnnnnng
(A) CR (72/401): 17.9%
(B)
Max score: 50.0
Total score: 50.0
Query cover: 6%
E value: 0.064
Identity: 100%

- KU529946.1: Dobrava-Belgrade virus strain Sochi/hu nucleocapsid protein gene, partial cds.
- KP878313.1: Dobrava-Belgrade virus strain 10752/hu segment S nucleocapsid protein gene, complete cds.
- KP878311.1: Dobrava-Belgrade virus strain 10636/Ap segment S nucleocapsid protein gene, complete cds.
- AF288647.1: Hantavirus AH211 nucleocapsid protein mRNA, complete cds.
- AF442623.1: Dobrava-Belgrade virus isolate P-s1223/Krasnodar-2000 nucleocapsid protein gene, partial cds.
- AF442622.1: Dobrava-Belgrade virus isolate Ap-1/Goryachiy Klyuch-2000 nucleocapsid protein gene, partial cds.
- AF285264.1: Hantavirus AH09 segment S nucleocapsid protein gene, complete cds.
- AB027523.1: Hantaan virus RNA for nucleocapsid protein, complete cds.
- **AJ009773.1: Dobrava virus S segment gene encoding nucleocapsid protein, strain DOB/Saaremaa/160V), genomic RNA.**

Origin: Slovenia

DOBV 15/01 Slovenia

>seq245:2_14-08-2013_(VirIDv3r520827) Start=12 End=412
ttgtgatctacctgacatcattcgtggtcccaatnctgttgaaggctctttncatgcttaccaccagagggagacaaactactaaaganaannaagga
atnaggattcgatttaaggatgacagctcttttgaagatgtgaatgggattcgnaagccaaagcacctgttcttgtcaatgcccaatgcacaatctagta
tgaaggcagatgngattacaccaggtcggttcnggactgcaatctgtggactatacccagcccaggttaaggcaaggaatttaatcagtcctgtgat
gagtgtgattgggtttgtagcccttgcaaaaaactggacagaacgggttgaagaatggcttgacctcccgtgcaagctacnatctgagccatctccaa
cgtctttg
(A) CR (391/401): 97.5%
(B)
Max score: 688
Total score: 688
Query coverage: 100%
E value: 0.0
Identity: 98%

- KF776845.1: Dobrava-Belgrade virus isolate HtSi_275_p2008 nonfunctional nucleocapsid protein gene, partial sequence.
- KF776819.1: Dobrava-Belgrade virus isolate HtSi_1036_a2001 nucleocapsid protein gene, partial cds.

>seq247:2_14-08-2013_(VirIDv3r520827) Start=12 End=412
nnnngnnnnngnnnnnngnanncntggtnnnnnnncngnnnnnnnnnnnnnnnnnnnnnnngnnnnngggagacaaacnacnn
nnnnnnnnnnnnnncnnnnnnnnnnannnnnnnnnnnncngnnntnttgaagatgtgaatgggnntnnannnnnnnnnnnnnnnnnn
nnnnnnnnnnncnnnnnnnnnngcnnnnnnnnggcagatgagattacacnaggtcggttcaggactgnnannnntgnncnnnnnnnan
cnnnnnnnncgnnnnnnnnnnnnnnnngnnnnnngnnnnntgtnnnnnggnnggnngnnnntgcaaaaaactggacagaacgggnt
nnannannnnnnnnnnnnnnnnnncnnnnnctatctgagccanntnnancgncnntg
(A) CR (150/401): 37.4%
(B)
Max score: 60.8
Total score: 103
Query coverage: 14%
E value: 4e-05
Identity: 97%

- KT885043.1: Dobrava-Belgrade virus strain Stamforrad/POR segment S, complete sequence.
- KF776846.1: Dobrava-Belgrade virus isolate HtSi_301_p2010 nucleocapsid protein gene, partial cds.
- KF776845.1: Dobrava-Belgrade virus isolate HtSi_275_p2008 nonfunctional nucleocapsid protein gene, partial sequence.
- KF776842.1: Dobrava-Belgrade virus isolate HtSi_279_p2008 nonfunctional nucleocapsid protein gene, partial sequence.
- KF776841.1: Dobrava-Belgrade virus isolate HtSi_261_p2008 nucleocapsid protein gene, partial cds.
- KF776839.1: Dobrava-Belgrade virus isolate HtSi_256_p2008 nucleocapsid protein gene, partial cds.
- KF776819.1: Dobrava-Belgrade virus isolate HtSi_1036_a2001 nucleocapsid protein gene, partial cds.
- KC676594.1: Dobrava-Belgrade virus strain DOBV/Croatia_Gerovo/Af956/2008 nucleocapsid protein (N) gene, partial cds.
- GU904032.1: Dobrava-Belgrade virus clone B5 nucleocapsid protein (N) gene, complete cds.
- GU904031.1: Dobrava-Belgrade virus clone A36 nucleocapsid protein (N) gene, complete cds.
- GU904029.1: Dobrava-Belgrade virus strain Slo/Af-BER nucleocapsid protein (N) gene, complete cds.
- L41916.1: Dobrava-Belgrade virus segment S, complete sequence.

>seq254:2_14-08-2013_(VirIDv3r520827) Start=12 End=412
nnnnnnnnnnnnnnnccnnnnnnnncnnngntnnnnnnnnnnnnnnnnnnnnnccnncnnnnnnnnnnnnncnnncnnnnnnnn
nnnnnnnnnnnnncnnnnnnnnnngatttaaggatgacagctcttttgnannnnngnntgggattcgaaagccaaagcncnnnnnnntn
nnnnnnnnnnnnnannnnntnntnnnnnnncanntnnnnntnnngnnnnccnnnnnnnngntgcaatctgtggncnnnnnnnnncn
nnnnnnnnnnnncnnnnnnnnnngnnnnngnnnnnnnnnnnnnnngnnngnnnnngnnnnnnnnnnnnnnnngnnnn
nnnnnnnnnnnggnnanggnnnnnnnnnnnnnnnnnnnnnnngnnnnnnnnnnnnn
(A) CR (97/401): 24.2%
(B)
Max score: 69.8
Total score: 69.8
Query coverage: 13%
E value: 7e-08
Identity: 85%

- KY649187.1: Dobrava-Belgrade orthohantavirus isolate S_2925_Hu_BA_11 nucleoprotein gene, partial cds.
- KY649184.1: Dobrava-Belgrade orthohantavirus isolate S_3861_Hu_CRS_08 nucleoprotein gene, partial cds.
- KY649180.1: Dobrava-Belgrade orthohantavirus isolate S_2890_Hu_WRS_10 nucleoprotein gene, partial cds.

- KT315642.1: Dobrava-Belgrade virus isolate Turkey/Igneada/89Af/2009 segment S nucleocapsid protein gene, partial cds.
- KT315641.1: Dobrava-Belgrade virus isolate Turkey/Igneada/84Af/2009 segment S nucleocapsid protein gene, partial cds.
- KT315640.1: Dobrava-Belgrade virus isolate Turkey/Igneada/57Af/2009 segment S nucleocapsid protein gene, partial cds.
- KT315639.1: Dobrava-Belgrade virus isolate Turkey/Igneada/32Af/2009 segment S nucleocapsid protein gene, partial cds.
- KT315638.1: Dobrava-Belgrade virus isolate Turkey/Igneada/13Af/2009 segment S nucleocapsid protein gene, partial cds.
- KT315636.1: Dobrava-Belgrade virus isolate Turkey/Igneada/03Af/2009 segment S nucleocapsid protein gene, partial cds.
- KT885043.1: Dobrava-Belgrade virus strain Stamforrad/POR segment S, complete sequence.
- KF776849.1: Dobrava-Belgrade virus isolate HtSi_274_p2008 nonfunctional nucleocapsid protein gene, partial sequence.
- KF776848.1: Dobrava-Belgrade virus isolate HtSi_297_p2010 nucleocapsid protein gene, partial cds.
- KF776845.1: Dobrava-Belgrade virus isolate HtSi_275_p2008 nonfunctional nucleocapsid protein gene, partial sequence.
- KF776843.1: Dobrava-Belgrade virus isolate HtSi_268_p2008 nucleocapsid protein gene, partial cds.
- KF776841.1: Dobrava-Belgrade virus isolate HtSi_261_p2008 nucleocapsid protein gene, partial cds.
- KF776838.1: Dobrava-Belgrade virus isolate HtSi_231_p2007 nonfunctional nucleocapsid protein gene, partial sequence.
- KF776837.1: Dobrava-Belgrade virus isolate HtSi_230_p2007 nonfunctional nucleocapsid protein gene, partial sequence.
- KF776836.1: Dobrava-Belgrade virus isolate HtSi_215_p2005 nucleocapsid protein gene, partial cds.
- KF776835.1: Dobrava-Belgrade virus isolate HtSi_207_p2005 nucleocapsid protein gene, partial cds.
- KF776834.1: Dobrava-Belgrade virus isolate HtSi_200_p2005 nucleocapsid protein gene, partial cds.
- KF776833.1: Dobrava-Belgrade virus isolate HtSi_129_p2000 nucleocapsid protein gene, partial cds.
- KF776832.1: Dobrava-Belgrade virus isolate HtSi_132_p2000 nucleocapsid protein gene, partial cds.
- KF776831.1: Dobrava-Belgrade virus isolate HtSi_136_p2001 nucleocapsid protein gene, partial cds.
- KF776830.1: Dobrava-Belgrade virus isolate HtSi_193_p2005 nucleocapsid protein gene, partial cds.
- KF776829.1: Dobrava-Belgrade virus isolate HtSi_1022_a2000 nonfunctional nucleocapsid protein gene, partial sequence.
- KF776828.1: Dobrava-Belgrade virus isolate HtSi_1030_a1995 nucleocapsid protein gene, partial cds.
- KF776827.1: Dobrava-Belgrade virus isolate HtSi_1038_a2002 nucleocapsid protein gene, partial cds.
- KF776826.1: Dobrava-Belgrade virus isolate HtSi_130_p2000 nucleocapsid protein gene, partial cds.
- KF776824.1: Dobrava-Belgrade virus isolate HtSi_1040_a1999 nucleocapsid protein gene, partial cds.
- KF776823.1: Dobrava-Belgrade virus isolate HtSi_1043_a1995 nucleocapsid protein gene, partial cds.

- KF776821.1: Dobrava-Belgrade virus isolate HtSi_1042_a2008 nucleocapsid protein gene, partial cds.
- KF776820.1: Dobrava-Belgrade virus isolate HtSi_1037_a2001 nucleocapsid protein gene, partial cds.
- KF776819.1: Dobrava-Belgrade virus isolate HtSi_1036_a2001 nucleocapsid protein gene, partial cds.
- KF776818.1: Dobrava-Belgrade virus isolate HtSi_1035_a2002 nucleocapsid protein gene, partial cds.
- KF776817.1: Dobrava-Belgrade virus isolate HtSi_1034_a2001 nucleocapsid protein gene, partial cds.
- KF776816.1: Dobrava-Belgrade virus isolate HtSi_1033_a2001 nucleocapsid protein gene, partial cds.
- KF776815.1: Dobrava-Belgrade virus isolate HtSi_1032_a2001 nucleocapsid protein gene, partial cds.
- KF776813.1: Dobrava-Belgrade virus isolate HtSi_1029_a1995 nucleocapsid protein gene, partial cds.
- KF776812.1: Dobrava-Belgrade virus isolate HtSi_1024_a2000 nucleocapsid protein gene, partial cds.
- KF776811.1: Dobrava-Belgrade virus isolate HtSi_1021_a1995 nucleocapsid protein gene, partial cds.
- KF776810.1: Dobrava-Belgrade virus isolate HtSi_1020_a2008 nucleocapsid protein gene, partial cds.
- KF776808.1: Dobrava-Belgrade virus isolate HtSi_1015_a1995 nucleocapsid protein gene, partial cds.
- KF776807.1: Dobrava-Belgrade virus isolate HtSi_1011_a2007 nucleocapsid protein gene, partial cds.
- KF776805.1: Dobrava-Belgrade virus isolate HtSi_1009_a1995 nonfunctional nucleocapsid protein gene, partial sequence.
- KF776804.1: Dobrava-Belgrade virus isolate HtSi_1008_a1999 nucleocapsid protein gene, partial cds.
- KF776803.1: Dobrava-Belgrade virus isolate HtSi_1013_a2002 nucleocapsid protein gene, partial cds.
- KF776799.1: Dobrava-Belgrade virus isolate HtSi_1014_a2002 nucleocapsid protein gene, partial cds.
- KF776797.1: Dobrava-Belgrade virus isolate HtSi_270_p2008 nonfunctional nucleocapsid protein gene, partial sequence.
- KF776796.1: Dobrava-Belgrade virus isolate HtSi_499_p2012 nucleocapsid protein gene, partial cds.
- KF776795.1: Dobrava-Belgrade virus isolate HtSi_492_p2012 nonfunctional nucleocapsid protein gene, partial sequence.
- KF776794.1: Dobrava-Belgrade virus isolate HtSi_465_p2012 nonfunctional nucleocapsid protein gene, partial sequence.
- KF776793.1: Dobrava-Belgrade virus isolate HtSi_397_p2012 nucleocapsid protein gene, partial cds.
- KJ154958.1: Dobrava-Belgrade virus strain KOS-1 nucleocapsid protein (N) gene, partial cds.
- KC676600.1: Dobrava-Belgrade virus strain DOBV/Croatia_Gerovo/Af968/2008 nucleocapsid protein (N) mRNA, complete cds.
- KC676599.1: Dobrava-Belgrade virus strain DOBV/Croatia_Gerovo/Af967/2008 nucleocapsid protein (N) mRNA, complete cds.
- KC676598.1: Dobrava-Belgrade virus strain DOBV/Croatia_Gerovo/Af966/2008 nucleocapsid protein (N) mRNA, complete cds.
- KC676597.1: Dobrava-Belgrade virus strain DOBV/Croatia_Gerovo/Af965/2008 nucleocapsid protein (N) mRNA, complete cds.

- KC676596.1: Dobrava-Belgrade virus strain DOBV/Croatia_Gerovo/Af964/2008 nucleocapsid protein (N) mRNA, partial cds.
- KC676593.1: Dobrava-Belgrade virus strain DOBV/Croatia_Gerovo/Af933/2008 nucleocapsid protein (N) gene, partial cds.
- KC676591.1: Dobrava-Belgrade virus strain DOBV/Croatia_Gerovo/Af903/2008 nucleocapsid protein (N) gene, complete cds.
- KC676589.1: Dobrava-Belgrade virus strain DOBV/Croatia_Gerovo/Af882/2008 nucleocapsid protein (N) gene, complete cds.
- KC197343.1: Dobrava-Belgrade virus strain V 36/10 nucleocapsid protein gene, partial cds.
- JF499666.1: Dobrava-Belgrade virus strain DOBcro231lm nucleocapsid protein gene, partial cds.
- JF499664.1 : Dobrava-Belgrade virus strain DOBcro100kt nucleocapsid protein gene, partial cds.
- GU904032.1: Dobrava-Belgrade virus clone B5 nucleocapsid protein (N) gene, complete cds.
- GU904031.1: Dobrava-Belgrade virus clone A36 nucleocapsid protein (N) gene, complete cds.
- GU904029.1: Dobrava-Belgrade virus strain Slo/Af-BER nucleocapsid protein (N) gene, complete cds.
- FN377828.2: Dobrava-Belgrade virus partial N gene for nucleocapsid protein, strain DOBV/Af27/HungaryTR17/00.
- DQ305281.1: Dobrava-Belgrade virus isolate DOB/PV/02 nucleocapsid gene, partial cds.
- DQ305280.1: Dobrava-Belgrade virus isolate DOB/TV/02 nucleocapsid gene, partial cds.
- AY168576.1: Dobrava virus strain East Slovakia/400Af/98 segment S nucleocapsid protein gene, complete cds.
- AJ269554.1: Dobrava virus partial gene encoding nucleocapsid protein, genomic RNA, strain East Slovakia-400-Af.
- AJ251998.1: Dobrava virus proviral partial gene for nucleocapsid protein, genomic RNA.
- AJ251997.1: Dobrava virus proviral partial gene for nucleocapsid protein, genomic RNA.
- AJ251996.1: Dobrava virus proviral partial gene for nucleocapsid protein, genomic RNA.
- L41916.1: Dobrava-Belgrade virus segment S, complete sequence.

>seq262:2_14-08-2013_(VirIDv3r520827) Start=12 End=412
nnnnnnnnnnnnnnnnnggnnnnnnnncnnnncnnnancnnnnnnnnnnnnnncnnnnnnnnnnnnnnnnnnnnnnnnnn
nnnnnnnnnnannncnnngnnnngnnnngnncgnntgacagctcttttgaagatgtgaatgggnnnnnnnnnnnnnnnnnnnncn
nnnnnnnnnnnnnnnnnncnatctagtatgaaggcagntnnnnnngnnnnannnnnncnnnnnnctgcnatctntggncnnngnnnn
ncnnnnnngcnnnnnnnnnnnnnnnnnnnnnnngngnnnnnnnnnnnnnnnngnnnnnnnnnncnngnnnnnnnnnnnnnn
nnnnnnnnnnngnnannnnnnnnnnnnncnncnnnnnnnnnnnnnnnnnnnnnnnnnnnn
(A) CR (90/401): 24.4%
(B)
Max score: 51.8
Total score: 51.8
Query coverage: 6%
E value: 0.018
Identity: 100%

- MH427133.1: Dobrava-Belgrade orthohantavirus isolate DOBV/Aitoloakarnania-259/17 nucleocapsid protein gene, partial cds.
- MH427132.1: Dobrava-Belgrade orthohantavirus isolate DOBV/Ioannina-54/17 nucleocapsid protein gene, partial cds.
- MH427131.1: Dobrava-Belgrade orthohantavirus isolate DOBV/Fokida-223/16 nucleocapsid protein gene, partial cds.
- MH427130.1: Dobrava-Belgrade orthohantavirus isolate DOBV/Thessaloniki-43/15 nucleocapsid protein gene, partial cds.
- MH427129.1: Dobrava-Belgrade orthohantavirus isolate DOBV/Edessa-216/14 nucleocapsid protein gene, partial cds.
- MH427128.1: Dobrava-Belgrade orthohantavirus isolate DOBV/Pella-89/14 nucleocapsid protein gene, partial cds.

- MH427127.1: Dobrava-Belgrade orthohantavirus isolate DOBV/Chalkidiki-398/13 nucleocapsid protein gene, partial cds.
- MH427126.1: Dobrava-Belgrade orthohantavirus isolate DOBV/Lamia-135/13 nucleocapsid protein gene, partial cds.
- MH427125.1: Dobrava-Belgrade orthohantavirus isolate DOBV/Drama-29/09 nucleocapsid protein gene, partial cds.
- MH427123.1: Dobrava-Belgrade orthohantavirus isolate DOBV/Drama-196/07 nucleocapsid protein gene, partial cds.
- MH427122.1: Dobrava-Belgrade orthohantavirus isolate DOBV/Imathia-159/07 nucleocapsid protein gene, partial cds.
- MH427121.1: Dobrava-Belgrade orthohantavirus isolate DOBV/Kozani-116/07 nucleocapsid protein gene, partial cds.
- MH427119.1: Dobrava-Belgrade orthohantavirus isolate DOBV/Larisa-272/06 nucleocapsid protein gene, partial cds.
- MH427118.1: Dobrava-Belgrade orthohantavirus isolate DOBV/Florina-167/06 nucleocapsid protein gene, partial cds.
- MH427117.1: Dobrava-Belgrade orthohantavirus isolate DOBV/Pieria-188/06 nucleocapsid protein gene, partial cds.
- MH427116.1: Dobrava-Belgrade orthohantavirus isolate DOBV/Chalkidiki-177/06 nucleocapsid protein gene, partial cds.
- MH427115.1: Dobrava-Belgrade orthohantavirus isolate DOBV/Pella-246/05 nucleocapsid protein gene, partial cds.
- MH427113.1: Dobrava-Belgrade orthohantavirus isolate DOBV/Ioannina-203/05 nucleocapsid protein gene, partial cds.
- MH427112.1: Dobrava-Belgrade orthohantavirus isolate DOBV/Magnesia-167/05 nucleocapsid protein gene, partial cds.
- MH427111.1: Dobrava-Belgrade orthohantavirus isolate DOBV/Grevena-240/04 nucleocapsid protein gene, partial cds.
- MH427110.1: Dobrava-Belgrade orthohantavirus isolate DOBV/Trikala-102/04 nucleocapsid protein gene, partial cds.
- MH427109.1: Dobrava-Belgrade orthohantavirus isolate DOBV/Fthiotida-75/03 nucleocapsid protein gene, partial cds.
- MH427107.1: Dobrava-Belgrade orthohantavirus isolate DOBV/Magnesia-73/02 nucleocapsid protein gene, partial cds.
- KY649188.1: Dobrava-Belgrade orthohantavirus isolate S_3528_Hu_SRS_11 nucleoprotein gene, partial cds.
- KY649187.1: Dobrava-Belgrade orthohantavirus isolate S_2925_Hu_BA_11 nucleoprotein gene, partial cds.
- KY649186.1: Dobrava-Belgrade orthohantavirus isolate S_5502_Hu_ERS_10 nucleoprotein gene, partial cds.
- KY649185.1: Dobrava-Belgrade orthohantavirus isolate S_5312_Hu_NRS_10 nucleoprotein gene, partial cds.
- KY649184.1: Dobrava-Belgrade orthohantavirus isolate S_3861_Hu_CRS_08 nucleoprotein gene, partial cds.
- KY649183.1: Dobrava-Belgrade orthohantavirus isolate S_3748_Hu_CRS_09 nucleoprotein gene, partial cds.
- KY649182.1: Dobrava-Belgrade orthohantavirus isolate S_3526_Hu_SRS_11 nucleoprotein gene, partial cds.
- KY649181.1: Dobrava-Belgrade orthohantavirus isolate S_3431_Hu_SRS_10 nucleoprotein gene, partial cds.
- KY649180.1: Dobrava-Belgrade orthohantavirus isolate S_2890_Hu_WRS_10 nucleoprotein gene, partial cds.

- KY649179.1: Dobrava-Belgrade orthohantavirus isolate S_2331_Hu_SRS_10 nucleoprotein gene, partial cds.
- KT971014.1: Dobrava-Belgrade virus strain Geshtenjas/Pogradec/261Af/2007/ALB nucleocapsid protein gene, partial cds.
- KT971013.1: Dobrava-Belgrade virus strain Mollas/Kolonje/243Af/2007/ALB nucleocapsid protein gene, partial cds.
- KT971011.1: Dobrava-Belgrade virus strain Diellas/Korce/201Af/2007/ALB nucleocapsid protein gene, partial cds.
- KT971010.1: Dobrava-Belgrade virus strain Llogora/Vlore/85Af/2006/ALB nucleocapsid protein gene, partial cds.
- KT971009.1: Dobrava-Belgrade virus strain Voskopoje/Korce/65Af/2006/ALB nucleocapsid protein gene, partial cds.
- KT971008.1: Dobrava-Belgrade virus strain Togez/Librazhd/54Af/2006/ALB nucleocapsid protein gene, partial cds.
- KT971007.1: Dobrava-Belgrade virus strain Llasen/Diber/22Af/2006/ALB nucleocapsid protein gene, partial cds.
- KT971006.1: Dobrava-Belgrade virus strain Pilafe/Diber/20Af/2006/ALB nucleocapsid protein gene, partial cds.
- KT971005.1: Dobrava-Belgrade virus strain Brezhdan/Diber/19Af/2006/ALB nucleocapsid protein gene, partial cds.
- KT315642.1: Dobrava-Belgrade virus isolate Turkey/Igneada/89Af/2009 segment S nucleocapsid protein gene, partial cds.
- KT315641.1: Dobrava-Belgrade virus isolate Turkey/Igneada/84Af/2009 segment S nucleocapsid protein gene, partial cds.
- KT315640.1: Dobrava-Belgrade virus isolate Turkey/Igneada/57Af/2009 segment S nucleocapsid protein gene, partial cds.
- KT315639.1: Dobrava-Belgrade virus isolate Turkey/Igneada/32Af/2009 segment S nucleocapsid protein gene, partial cds.
- KT315638.1: Dobrava-Belgrade virus isolate Turkey/Igneada/13Af/2009 segment S nucleocapsid protein gene, partial cds.
- KT315637.1: Dobrava-Belgrade virus isolate Turkey/Igneada/09Af/2009 segment S nucleocapsid protein gene, partial cds.
- KT315636.1: Dobrava-Belgrade virus isolate Turkey/Igneada/03Af/2009 segment S nucleocapsid protein gene, partial cds.
- KT315635.1: Dobrava-Belgrade virus isolate Turkey/Igneada/02Af/2009 segment S nucleocapsid protein gene, partial cds.
- KT885043.1: Dobrava-Belgrade virus strain Stamforrad/POR segment S, complete sequence.
- KF776850.1: Dobrava-Belgrade virus isolate HtSi_286_p2009 nonfunctional nucleocapsid protein gene, partial sequence.
- KF776849.1: Dobrava-Belgrade virus isolate HtSi_274_p2008 nonfunctional nucleocapsid protein gene, partial sequence.
- KF776848.1: Dobrava-Belgrade virus isolate HtSi_297_p2010 nucleocapsid protein gene, partial cds.
- KF776846.1: Dobrava-Belgrade virus isolate HtSi_301_p2010 nucleocapsid protein gene, partial cds.
- KF776845.1: Dobrava-Belgrade virus isolate HtSi_275_p2008 nonfunctional nucleocapsid protein gene, partial sequence.
- KF776843.1: Dobrava-Belgrade virus isolate HtSi_268_p2008 nucleocapsid protein gene, partial cds.
- KF776842.1: Dobrava-Belgrade virus isolate HtSi_279_p2008 nonfunctional nucleocapsid protein gene, partial sequence.
- KF776841.1: Dobrava-Belgrade virus isolate HtSi_261_p2008 nucleocapsid protein gene, partial cds.

- KF776840.1: Dobrava-Belgrade virus isolate HtSi_239_p2008 nonfunctional nucleocapsid protein gene, partial sequence.
- KF776839.1: Dobrava-Belgrade virus isolate HtSi_256_p2008 nucleocapsid protein gene, partial cds.
- KF776838.1: Dobrava-Belgrade virus isolate HtSi_231_p2007 nonfunctional nucleocapsid protein gene, partial sequence.
- KF776837.1: Dobrava-Belgrade virus isolate HtSi_230_p2007 nonfunctional nucleocapsid protein gene, partial sequence.
- KF776836.1: Dobrava-Belgrade virus isolate HtSi_215_p2005 nucleocapsid protein gene, partial cds.
- KF776835.1: Dobrava-Belgrade virus isolate HtSi_207_p2005 nucleocapsid protein gene, partial cds.
- KF776834.1: Dobrava-Belgrade virus isolate HtSi_200_p2005 nucleocapsid protein gene, partial cds.
- KF776833.1: Dobrava-Belgrade virus isolate HtSi_129_p2000 nucleocapsid protein gene, partial cds.
- KF776832.1: Dobrava-Belgrade virus isolate HtSi_132_p2000 nucleocapsid protein gene, partial cds.
- KF776831.1: Dobrava-Belgrade virus isolate HtSi_136_p2001 nucleocapsid protein gene, partial cds.
- KF776830.1: Dobrava-Belgrade virus isolate HtSi_193_p2005 nucleocapsid protein gene, partial cds.
- KF776829.1: Dobrava-Belgrade virus isolate HtSi_1022_a2000 nonfunctional nucleocapsid protein gene, partial sequence.
- KF776828.1: Dobrava-Belgrade virus isolate HtSi_1030_a1995 nucleocapsid protein gene, partial cds.
- KF776827.1: Dobrava-Belgrade virus isolate HtSi_1038_a2002 nucleocapsid protein gene, partial cds.
- KF776826.1: Dobrava-Belgrade virus isolate HtSi_130_p2000 nucleocapsid protein gene, partial cds.
- KF776824.1: Dobrava-Belgrade virus isolate HtSi_1040_a1999 nucleocapsid protein gene, partial cds.
- KF776823.1: Dobrava-Belgrade virus isolate HtSi_1043_a1995 nucleocapsid protein gene, partial cds.
- KF776821.1: Dobrava-Belgrade virus isolate HtSi_1042_a2008 nucleocapsid protein gene, partial cds.
- KF776820.1: Dobrava-Belgrade virus isolate HtSi_1037_a2001 nucleocapsid protein gene, partial cds.
- KF776819.1: Dobrava-Belgrade virus isolate HtSi_1036_a2001 nucleocapsid protein gene, partial cds.
- KF776818.1: Dobrava-Belgrade virus isolate HtSi_1035_a2002 nucleocapsid protein gene, partial cds.
- KF776817.1: Dobrava-Belgrade virus isolate HtSi_1034_a2001 nucleocapsid protein gene, partial cds.
- KF776816.1: Dobrava-Belgrade virus isolate HtSi_1033_a2001 nucleocapsid protein gene, partial cds.
- KF776815.1: Dobrava-Belgrade virus isolate HtSi_1032_a2001 nucleocapsid protein gene, partial cds.
- KF776813.1: Dobrava-Belgrade virus isolate HtSi_1029_a1995 nucleocapsid protein gene, partial cds.
- KF776812.1: Dobrava-Belgrade virus isolate HtSi_1024_a2000 nucleocapsid protein gene, partial cds.

- KF776811.1: Dobrava-Belgrade virus isolate HtSi_1021_a1995 nucleocapsid protein gene, partial cds.
- KF776810.1: Dobrava-Belgrade virus isolate HtSi_1020_a2008 nucleocapsid protein gene, partial cds.
- KF776808.1: Dobrava-Belgrade virus isolate HtSi_1015_a1995 nucleocapsid protein gene, partial cds.
- KF776807.1: Dobrava-Belgrade virus isolate HtSi_1011_a2007 nucleocapsid protein gene, partial cds.
- KF776805.1: Dobrava-Belgrade virus isolate HtSi_1009_a1995 nonfunctional nucleocapsid protein gene, partial sequence.
- KF776804.1: Dobrava-Belgrade virus isolate HtSi_1008_a1999 nucleocapsid protein gene, partial cds.
- KF776803.1: Dobrava-Belgrade virus isolate HtSi_1013_a2002 nucleocapsid protein gene, partial cds.
- KF776799.1: Dobrava-Belgrade virus isolate HtSi_1014_a2002 nucleocapsid protein gene, partial cds.
- KF776797.1: Dobrava-Belgrade virus isolate HtSi_270_p2008 nonfunctional nucleocapsid protein gene, partial sequence.
- KF776796.1: Dobrava-Belgrade virus isolate HtSi_499_p2012 nucleocapsid protein gene, partial cds.
- KF776795.1: Dobrava-Belgrade virus isolate HtSi_492_p2012 nonfunctional nucleocapsid protein gene, partial sequence.
- KF776794.1: Dobrava-Belgrade virus isolate HtSi_465_p2012 nonfunctional nucleocapsid protein gene, partial sequence.
- KF776793.1: Dobrava-Belgrade virus isolate HtSi_397_p2012 nucleocapsid protein gene, partial cds.
- KJ154958.1: Dobrava-Belgrade virus strain KOS-1 nucleocapsid protein (N) gene, partial cds.
- KJ437511.1: Dobrava-Belgrade virus isolate DOBV_RAV69S nucleocapsid protein gene, partial cds.

<u>Dobrava 86/18 Slovenia</u>

>seq245:3_14-08-2013_(VirIDv3r520827) Start=12 End=412
nnntggncnnncnancnnnctncgtggtcccaanncncnnnnnnnnnnnnnnnncnnnnnnnnnnnnnnnnncnnnnngnnnnnnnn
nnnnnnnnnnggaatgaggattcgctttaaggatgacagctcgnttgaagatgtgaatgggattcgnaagccaaagcacctgtnncnancnnn
gcccaatgctcnntccngtatgaaggcagnnnnannnnnngnnnnccggntcagnnnngnnnnnnnngnntnnngnnnagnnnnnnn
nnnnnnnnnnnnnnnnnnnnngnnnncnngnnngnnnnnnnnnnnnnnnnnnngncaaagnacnnnnnnnnnnnancnnnnn
nnnnnnnnnnnnnnnnnnncnntnactatctgagccatcnnnnnnnnnnnnc
(A) CR (173/401): 43.1%
(B)
Max score: 159
Total score: 159
Query coverage: 27%
E value: 5e-35
Identity: 89%

- KF776882.1: Dobrava-Belgrade virus isolate HtSi_1012_a1997 nucleocapsid protein gene, partial cds.
- KF776853.1: Dobrava-Belgrade virus isolate HtSi_1091_a1997 nucleocapsid protein gene, partial cds.
- KF776852.1: Dobrava-Belgrade virus isolate HtSi_1096_a1995 nucleocapsid protein gene, partial cds.
- KF776822.1: Dobrava-Belgrade virus isolate HtSi_1095_a1997 nucleocapsid protein gene, partial cds.

- KF776802.1: Dobrava-Belgrade virus isolate HtSi_1090_a2000 nonfunctional nucleocapsid protein gene, partial sequence.
- KF776801.1: Dobrava-Belgrade virus isolate HtSi_1092_a1997 nucleocapsid protein gene, partial cds.
- KF776800.1: Dobrava-Belgrade virus isolate HtSi_1097_a1995 nucleocapsid protein gene, partial cds.
- AJ251999.1: Dobrava virus proviral partial gene for nucleocapsid protein, genomic RNA.

>seq246:3_14-08-2013_(VirIDv3r520827) Start=12 End=412
nnnnnnnnnnnnnnnnnngnnnnncnnnngnnaatactcttaaaggnncnnnncnnnnngnnaactagagggagncagactactaagga
taacangggaatgaggattcgctntnaggatgacagctcgtttgaagnnnnnngnnnccgnnnnnnnnnnnnnnnnnnnnnnnnnancaat
gcccantgctcaatccagtatgaaggcagatgaaancncacctgnnnngnncnnnnnnnnnngtntgtgnnttanncncnnnncaanncnna
gcaagaaatttggncngtcctgtcatgagtgtanntggttttttggcccttgcaaagaactggnnnnnannannngnagagtggttagatnngnn
nnnngnnnnnncnnntnagccatcnncancnnnncnn
(A) CR (248/401): 61.8%
(B)
Max score: 221
Total score: 221
Query coverage: 81%
E value: 2e-53
Identity: 69%
- KF776882.1: Dobrava-Belgrade virus isolate HtSi_1012_a1997 nucleocapsid protein gene, partial cds.
- KF776822.1: Dobrava-Belgrade virus isolate HtSi_1095_a1997 nucleocapsid protein gene, partial cds.
- KF776802.1: Dobrava-Belgrade virus isolate HtSi_1090_a2000 nonfunctional nucleocapsid protein gene, partial sequence.
- KF776801.1: Dobrava-Belgrade virus isolate HtSi_1092_a1997 nucleocapsid protein gene, partial cds.
- KF776800.1: Dobrava-Belgrade virus isolate HtSi_1097_a1995 nucleocapsid protein gene, partial cds.

>seq247:3_14-08-2013_(VirIDv3r520827) Start=12 End=412
nnnngngncnnnnnnnngnnnnccnggtnnnannncnntnnnnnnnnnntttacatgttgacaactagagggagacagncnncnntnnnt
nnnnnnnnnnnnnnnnnnnncnnnnnnnnnnnncnnnnngnttgaagatgtgaatgggantnnacnnnnnnnnnnnncnnnnnnnnn
nnnncnnnnnnnnnnnngcnnnnnnnnnnnnnnnnnnnnnnnnngnnnncnggttcaggnnngcnnnttgtggatnnnnnnnancnn
nnnnnnnngnnnnnanntngnnnngnnnnnngnnnnnnnnannnnnnnttttggcccttgcaaagaactggacnncnnnnncnnnnn
nnnnnnnnnntnttccangcnnntnncnnnctgagccatccccaacatcncnn
(A) CR (159/401): 39.6%
(B)
Max score: 53.6
Total score: 99
Query coverage: 13%
E value: 0.005
Identity: 100%
- KF776882.1: Dobrava-Belgrade virus isolate HtSi_1012_a1997 nucleocapsid protein gene, partial cds.
- KF776852.1: Dobrava-Belgrade virus isolate HtSi_1096_a1995 nucleocapsid protein gene, partial cds.
- KF776822.1: Dobrava-Belgrade virus isolate HtSi_1095_a1997 nucleocapsid protein gene, partial cds.
- KF776802.1: Dobrava-Belgrade virus isolate HtSi_1090_a2000 nonfunctional nucleocapsid protein gene, partial sequence.

- KF776801.1: Dobrava-Belgrade virus isolate HtSi_1092_a1997 nucleocapsid protein gene, partial cds.
- KF776800.1: Dobrava-Belgrade virus isolate HtSi_1097_a1995 nucleocapsid protein gene, partial cds.
- FN813291.1: Saaremaa virus partial S segment, strain SAAV/Croatia_Migalovci/Aa12/2003, genomic RNA.
- FN377826.2: Saaremaa virus partial N gene for nucleocapsid protein, strain SAAV/Aa1/HungaryTR16/00.
- AJ251999.1: Dobrava virus proviral partial gene for nucleocapsid protein, genomic RNA.

Viral species: Tula orthohantavirus (TULV)

Origin: Germany

TULV S666_13 Schlindermanderscheid

>seq261:23_16-09-2014_(VirIDv3r520827) Start=12 End=412
nngnnnnnnnnnnnnnnnnnnnnnnncnnnnnnnnnnnnnnnnnnnngncnnnnnntnnntnttgtcaacnnnnnncnggnnngnnnnnnn
nnnnnnnnnannnnnnnnnngncnnngnnnnnnnnnnnnnnnnnngnnnngnnnnnnnnnnnnnnnnnnnngnnnnntnnnn
nnnnnnnnnnnnnnnnnnnnnnnnntcnnnnnnngnnnnnnnnnnnnnnnnnnnnngtncaggacaattgtttgtggacttttcccn
gncnncncgnnnccccnnnncnncanangncctgtgatgggtgtgatcggattttncnnnnnnnnnnnnnnnnnnnnnnnnnnnnnn
nnnnnnnngnncnnnnnnnnnnnnnnnnnnnnnnncncnnngnnnnnnnnnnncnnnn
(A) CR (104/401): 25.9%
(B)
Max score: 64.4
Total score: 64.4
Query coverage: 21%
E value: 3e-06
Identity: 72%
- KU139603.1: Tula virus isolate S667_13_Marv nucleocapsid protein gene, partial cds.
- KU139602.1: Tula virus isolate S666_13_Marv nucleocapsid protein gene, partial cds.

>seq264:23_16-09-2014_(VirIDv3r520827) Start=12 End=412
ncnnnnnnnnnnnnnnnnnnnnnnnnnnnnnnnnnnnnnnnnnnnnnnnnnnnnnnnnnnnnnnnnnnnnncngncnnnnnnc
nnnnnnnnnnnnnnnnnnnngnnnncnnnnnnngncnnnnnncgnnnnnnnnnnnnnnnnnggnncnngnnnnnnnnnnnntn
nnnntgtnnncnnnncgnnnnnnnnnncnnngtnnnnnnnagatnagntgncaccngnnnnnnncnggnnnattgtttgtggacttttnc
ntnnnncnnnnntgcacagaaatatcataannnntnngnnnnnnnnnnnnnnnnnnnnnnnnnnnnnnannnnncngnnnngnannn
ngnnntnnnnnnnnnnngcnnnnngnnnnnnnnnnnnnnncnnnnncnnnnnnnnnnnnn
(A) CR (93/401): 23.2%
(B)
Max score: 46.4
Total score: 46.4
Query coverage: 19%
E value: 0.77
Identity: 66%
- KU139603.1: Tula virus isolate S667_13_Marv nucleocapsid protein gene, partial cds.
- KU139602.1: Tula virus isolate S666_13_Marv nucleocapsid protein gene, partial cds.

>seq265:23_16-09-2014_(VirIDv3r520827) Start=12 End=412
tcggcnnncnngncnnnnnntnncancnnnncnacnnnnnnnnnnnnnnnnnntntgttgtnannnnnnnnnnnnnnnnnnnnnnn
nnnnnnnnanngacaagaatccggttcanggatgacagctctnnnnnnnnnnnngnncnnnnnnnnnnnnnnnnnnnnnnnnnn
ngnnnnnnnnnnnnnnnnnnnnnantgannnnagatgngttgacaccaggnaggnncnggnnnattgtttgtggacntnnnncnnnnnc
nnnccnnnnnnnnaaatatcatnagtcctgngntngnngtgntcggatnnnnnnnnnnnnnccnnnncnggcctgaaaagattgnncnnnn
nnnnnnnnnnnnnnnnngnnnnngnnnnnngnnnnnnnnnnngnnnnn
(A) CR (153/401): 38.1%
(B)
Max score: 53.6
Total score: 107
Query coverage: 33%
E value: 0.005
Identity: 97%
- KU139603.1: Tula virus isolate S667_13_Marv nucleocapsid protein gene, partial cds.
- KU139602.1: Tula virus isolate S666_13_Marv nucleocapsid protein gene, partial cds.

TULV 09_1000 Cunnersdorf

>seq265:24_16-09-2014_(VirIDv3r520827) Start=12 End=412
nnnnnnacnnnnncnnnnnnnngnannnnnncnnnnnnngnnnnncnnnnnnnnnnctgncaacnngagnnnggcannnanncn
ngnngnnnnnngnnacaagnannggnncnnngnngncagcncnnnngaagagatcaanggnnncnnnnnnnnnnnnnnnnn
nngnnngnnnnnnnnnnnnnnnnnncncnntgaaagccgnngnnnnnnnngnnnncnnanncngngnnnnnnnntgnggnnnnn
nnnnngcncngnnnnngcacaganacnncanaagtcctgncntnnnnnnnnnnncnnnnnnngnnnnnnnnaagattggccngaaa
agnnnnnncnnngnnngnncnnnnnnngnnnnnnntngaagnaaagtggtcctagtaaggaa
(A) CR (154/401): 38.4%
(B)
Max score: 42.8
Total score: 42.8
Query coverage: 6%
E value: 9.4
Identity: 96%
- KU139560.1: Tula virus isolate 09_1000_Marv nucleocapsid protein gene, partial cds.
- KU139559.1: Tula virus isolate 09_0972_Marv nucleocapsid protein gene, partial cds.
- AJ223601.1: Tula virus genomic RNA for nucleocapsid protein, (strain Tula/Koziky/5276Ma/94).
- Y13984.1: Tula virus partial genomic RNA for nucleocapsid protein, strain (Tula/D540/Ma/95).
- Z69991.1: Tula virus genomic RNA for nucleocapsid protein, strain (Tula/Moravia/5302v/95).
- Z49915.1: Tula virus segment S, strain Tula/Moravia/5302Ma/94, genomic RNA.
- Z48741.1: Tula virus segment S, strain Tula/Moravia/5294Ma/94, genomic RNA.
- Z48574.1: Tula virus segment S, strain Tula/Moravia/5293Ma/94, genomic RNA.
- Z48573.1: Tula virus segment S, strain Tula/Moravia/5286Ma/94, genomic RNA.

Origin: Finland

TULV laboratory strain Moravia

>seq234:2_08-08-2013_(VirIDv3r520827) Start=12 End=238
nnnnnnncnnnnncnnnngncnnnnnnnnnnnancgnnnnnnnngtnnnnnnnngnnnnnnnnnnnnnnnnnnnnnnnnncnnn
nnnnnnnnnnnnnnnnnntnnngnnnnnnnnnnnnnngngnncnnnnnnnnnnnnnngtcagcattggaggacaaacnggcagant
tcaagnnnnnnnngnnnnnnnnnggnnnnnnnnnnnnnnnnnnnnnnnnnnggnngn
(A) CR (54/227): 23.8%
(B)
Max score: 55.4
Total score: 55.4
Query coverage: 14%
E value: 8e-04
Identity: 94%
- AF063897.1: Hantavirus Lodz-2 S segment nucleocapsid protein mRNA, complete cds.
- AF063892.1: Hantavirus Lodz-1 S segment nucleocapsid protein mRNA, complete cds.
- AF289821.1: Tula virus isolate D63-98 segment S nucleocapsid protein mRNA, complete cds.
- AF289820.1: Tula virus isolate D17-98 segment S nucleocapsid protein mRNA, complete cds.
- AF289819.1: Tula virus isolate D5-98 segment S nucleocapsid protein mRNA, complete cds.
- AF164094.1: Tula virus S segment nucleocapsid protein mRNA, complete cds.
- Z69991.1: Tula virus genomic RNA for nucleocapsid protein, strain (Tula/Moravia/5302v/95).
- Z49915.1: Tula virus segment S, strain Tula/Moravia/5302Ma/94, genomic RNA.

>seq244:2_08-08-2013_(VirIDv3r520827) Start=12 End=412
nnnncnnnngnnnnnnnnnnnnnnnnnnnnnncnnnnnnnnnnnnnnnnnnnnnnnnnnnnnnnnnnnnnnnnnnnnngnnnn
nncnnnnngnnnnnncnnnnnnnnnnngnncnnnnnnnnnnnnnnnnnnnnnnncnnnnnnnannnnnnnncnnnnnnncncnn

nnnnnnngntnnnnnnnnnnnnnnncnnnnncnnnnngnncccanncnnnnnngnnnncnnnngnnnnnnnnngnnnnnnnn
nnnnnnngngnnnnnnnncnnnnnnnnngngncnnangtcctgtcatgggtgtgattggannnnnnnngnncnnnnnnnnnnnnnn
nncnnnnnnnnngnnnnngnnnnnnnnnnnnnnnnnnnnngncnnnnnnnnnnnnnnnnnnnn

(A) CR (65/401): 16.2%

(B)

Max score: 42.8
Total score: 42.8
Query coverage: 5%
E value: 9.4
Identity: 100%

- KU139579.1: Tula virus isolate 10_1188_Magr nucleocapsid protein gene, partial cds.
- KU139578.1: Tula virus isolate 10_0932_Marv nucleocapsid protein gene, partial cds.
- KU139577.1: Tula virus isolate 10_0908_Marv nucleocapsid protein gene, partial cds.
- KU139576.1: Tula virus isolate 10_0905_Marv nucleocapsid protein gene, partial cds.
- KU139575.1: Tula virus isolate 12_1121_Marv nucleocapsid protein gene, partial cds.
- KU139574.1: Tula virus isolate 12_1068_Marv nucleocapsid protein gene, partial cds.
- KU139573.1: Tula virus isolate 10_1533_Marv nucleocapsid protein gene, partial cds.
- KU139572.1: Tula virus isolate 12_526_Magr nucleocapsid protein gene, partial cds.
- KU139571.1: Tula virus isolate 12_0492_Magr nucleocapsid protein gene, partial cds.
- KU139570.1: Tula virus isolate 08_0545_Marv nucleocapsid protein gene, partial cds.
- KU139569.1: Tula virus isolate 08_0362_Marv nucleocapsid protein gene, partial cds.
- KU139568.1: Tula virus isolate 08_0356_Marv nucleocapsid protein gene, partial cds.
- KU139567.1: Tula virus isolate 08_0352_Marv nucleocapsid protein gene, partial cds.
- KU139566.1: Tula virus isolate 08_0350_Marv nucleocapsid protein gene, partial cds.
- KU139565.1: Tula virus isolate 08_0538_Marv nucleocapsid protein gene, partial cds.
- KU139564.1: Tula virus isolate 08_0534_Marv nucleocapsid protein gene, partial cds.
- KU139548.1: Tula virus isolate 10_1625_Marv nucleocapsid protein gene, partial cds.
- KU139546.1: Tula virus isolate 07_0081_Magr nucleocapsid protein gene, partial cds.
- KU139545.1: Tula virus isolate 08_0894_Marv nucleocapsid protein gene, partial cds.
- KU139544.1: Tula virus isolate 08_0849_Marv nucleocapsid protein gene, partial cds.
- KU139543.1: Tula virus isolate 08_0848_Marv nucleocapsid protein gene, partial cds.
- KU139542.1: Tula virus isolate 08_0802_Marv nucleocapsid protein gene, partial cds. .
- KU139541.1: Tula virus isolate 08_0789_Marv nucleocapsid protein gene, partial cds.
- KU139540.1: Tula virus isolate 11_1433_Marv nucleocapsid protein gene, partial cds.
- KU139539.1: Tula virus isolate 11_1429_Marv nucleocapsid protein gene, partial cds.
- KU139536.1: Tula virus isolate 11_1373_Marv nucleocapsid protein gene, partial cds.
- KT885046.1: Sin Nombre virus strain CC107/POR segment S, complete sequence.
- HQ697347.1: Tula virus isolate GER/09/2155/Arv nucleocapsid protein gene, partial cds.
- GU300137.1: Tula virus strain dpz06-29 nucleocapsid protein gene, partial cds.
- GU300135.1: Tula virus strain dpz06-1 nucleocapsid protein gene, partial cds.
- EU439952.1: Tula virus strain Sennickerode Sen05/222 nucleocapsid protein gene, partial cds.
- EU439951.1: Tula virus strain Sennickerode Sen05/205 nucleocapsid protein gene, complete cds.
- EU439950.1: Tula virus strain Sennickerode Sen05/204 nucleocapsid protein gene, complete cds.
- EU439949.1: Tula virus strain Sennickerode Sen05/175 nucleocapsid protein gene, complete cds.
- EU439948.1: Tula virus strain Sennickerode Sen05/174 nucleocapsid protein gene, partial cds.
- EU439946.1: Tula virus strain Sennickerode Sen05/121 nucleocapsid protein gene, partial cds.
- U54575.1: Muleshoe hantavirus nucleocapsid protein (N) and nonstructural protein (NSx) mRNA, complete cds.
- AJ223600.1: Tula virus genomic RNA for nucleocapsid protein, (strain Tula/Koziky/5247Ma/94).
- Y13984.1: Tula virus partial genomic RNA for nucleocapsid protein, strain (Tula/D540/Ma/95).
- U95304.1: Tula virus O20 nucleocapsid protein gene, partial cds.
- U95303.1: Tula virus O52 nucleocapsid protein gene, partial cds.

- U95302.1: Tula virus O24 nucleocapsid protein gene, partial cds.
- Z69991.1: Tula virus genomic RNA for nucleocapsid protein, strain (Tula/Moravia/5302v/95).
- Z49915.1: Tula virus segment S, strain Tula/Moravia/5302Ma/94, genomic RNA.
- Z48741.1: Tula virus segment S, strain Tula/Moravia/5294Ma/94, genomic RNA.
- Z48574.1: Tula virus segment S, strain Tula/Moravia/5293Ma/94, genomic RNA.
- Z48573.1: Tula virus segment S, strain Tula/Moravia/5286Ma/94, genomic RNA.
- U10890.1: Four Corners hantavirus Sweetwater Canyon 1 nucleocapsid protein gene, partial cds.
- L33683.1: Pulmonary syndrome hantavirus (Convict Creek 107) S RNA segment.

>seq261:2_08-08-2013_(VirIDv3r520827) Start=12 End=412
nnnncnnnnnnnnnnnnncncnnnnnnnnnnnncnnnnnnncnccnnnnnngcnnnncngtcaactagaggnaggcaaannnnnnn
nnnnnnnnggnnncnngnngnnnncgnnnnannnnngnnnnnnnnnnnnnnnnnntggcattagacgccngnnncnnnnnnnt
ntgtctatgccaacagcccagnctcccnnnnnnnnnnnnnnnnncgnnnnnnnncnnnngnngnnnaattgtttgtggncncnntnnnnnn
nncnngnnnnnnnnnnnnnnnnnnannnnntgncatgggtntgnntnnnnnnnnnnnnnnnnnnnnnnnnnnnnnnnnnnngnnnn
nnnnnnnnnnnnnnnnncncncnnnngngnngnnncnnnnnnnnnnnnnnnncnnnn
(A) CR (124/401): 30.9%
(B)
Max score: 48.2
Total score: 48.2
Query coverage: 13%
E value: 0.22
Identity: 75%
- Y13982.1: Tula virus partial genomic RNA for nucleocapsid protein, strain (Tula/Ma715/Ma/95).
- AJ223601.1: Tula virus genomic RNA for nucleocapsid protein, (strain Tula/Koziky/5276Ma/94).
- AJ223600.1: Tula virus genomic RNA for nucleocapsid protein, (strain Tula/Koziky/5247Ma/94).
- Y13984.1: Tula virus partial genomic RNA for nucleocapsid protein, strain (Tula/D540/Ma/95).
- Z69991.1: Tula virus genomic RNA for nucleocapsid protein, strain (Tula/Moravia/5302v/95).
- Z49915.1: Tula virus segment S, strain Tula/Moravia/5302Ma/94, genomic RNA.
- Z48741.1: Tula virus segment S, strain Tula/Moravia/5294Ma/94, genomic RNA.
- Z48574.1: Tula virus segment S, strain Tula/Moravia/5293Ma/94, genomic RNA.
- Z48573.1: Tula virus segment S, strain Tula/Moravia/5286Ma/94, genomic RNA.

>seq263:2_08-08-2013_(VirIDv3r520827) Start=12 End=412
nntngcnnnnnnnnnnnnnnncnnnnnnnnnnnnnnnnnnnnnnnnnnnnnnnnnntnnncnnnnnggnnnnnnnnnnnnnnnnnn
nnnnnnnnnnnnnnnnngnnnnncnntttaaggatgacagctnananannnnnnnnnnnnncnnnnnnnncnnncannnnnnnnnnn
nnnnagnccncnnnnannnnnnnnntnacnnganagncgnnnnnnngnnngcnggtaggttnnnnnnnnggnnnnnngnnnnnngn
nnnnnnnnagnnnnnnnnnnnnnnnncnnnagtcctgtcatgggtgtgattggnngngnnngnngnnngnnnnnnnnnnnnnnnn
nnngnngncnngnnnnnnnnnnnnncnnnnnnnnnnngnnnnnnnnnnncnnntnnngnnnn
(A) CR (102/401): 25.4%
(B)
Max score: 42.8
Total score: 42.8
Query coverage: 5%
E value: 9.4
Identity: 100%
- MF687741.1: Tula orthohantavirus strain Tul/NL/Ma126-Fr-A&W/2015 nucleocapsid protein gene, partial cds.
- KU139600.1: Tula virus isolate 08_0277_Marv nucleocapsid protein gene, partial cds.
- KU139599.1: Tula virus isolate 08_0260_Marv nucleocapsid protein gene, partial cds.
- KU139598.1: Tula virus isolate 08_1033_Marv nucleocapsid protein gene, partial cds.
- KU139597.1: Tula virus isolate 10_0215_Marv nucleocapsid protein gene, partial cds.
- KU139596.1: Tula virus isolate 10_0185_Marv nucleocapsid protein gene, partial cds.
- KU139595.1: Tula virus isolate 10_0183_Marv nucleocapsid protein gene, partial cds.

- KU139579.1: Tula virus isolate 10_1188_Magr nucleocapsid protein gene, partial cds.
- KU139578.1: Tula virus isolate 10_0932_Marv nucleocapsid protein gene, partial cds.
- KU139577.1: Tula virus isolate 10_0908_Marv nucleocapsid protein gene, partial cds.
- KU139576.1: Tula virus isolate 10_0905_Marv nucleocapsid protein gene, partial cds.
- KU139575.1: Tula virus isolate 12_1121_Marv nucleocapsid protein gene, partial cds.
- KU139574.1: Tula virus isolate 12_1068_Marv nucleocapsid protein gene, partial cds.
- KU139573.1: Tula virus isolate 10_1533_Marv nucleocapsid protein gene, partial cds.
- KU139572.1: Tula virus isolate 12_526_Magr nucleocapsid protein gene, partial cds.
- KU139571.1: Tula virus isolate 12_0492_Magr nucleocapsid protein gene, partial cds.
- KU139570.1: Tula virus isolate 08_0545_Marv nucleocapsid protein gene, partial cds.
- KU139569.1: Tula virus isolate 08_0362_Marv nucleocapsid protein gene, partial cds.
- KU139568.1: Tula virus isolate 08_0356_Marv nucleocapsid protein gene, partial cds.
- KU139567.1: Tula virus isolate 08_0352_Marv nucleocapsid protein gene, partial cds.
- KU139566.1: Tula virus isolate 08_0350_Marv nucleocapsid protein gene, partial cds.
- KU139565.1: Tula virus isolate 08_0538_Marv nucleocapsid protein gene, partial cds.
- KU139564.1: Tula virus isolate 08_0534_Marv nucleocapsid protein gene, partial cds.
- KU139548.1: Tula virus isolate 10_1625_Marv nucleocapsid protein gene, partial cds.
- KU139546.1: Tula virus isolate 07_0081_Magr nucleocapsid protein gene, partial cds.
- KU139545.1: Tula virus isolate 08_0894_Marv nucleocapsid protein gene, partial cds.
- KU139544.1: Tula virus isolate 08_0849_Marv nucleocapsid protein gene, partial cds.
- KU139543.1: Tula virus isolate 08_0848_Marv nucleocapsid protein gene, partial cds.
- KU139542.1: Tula virus isolate 08_0802_Marv nucleocapsid protein gene, partial cds.
- KU139541.1: Tula virus isolate 08_0789_Marv nucleocapsid protein gene, partial cds.
- KU139540.1: Tula virus isolate 11_1433_Marv nucleocapsid protein gene, partial cds.
- KU139539.1: Tula virus isolate 11_1429_Marv nucleocapsid protein gene, partial cds.
- KU139538.1: Tula virus isolate 08_1045_Marv nucleocapsid protein gene, partial cds.
- KU139537.1: Tula virus isolate 08_0639_Marv nucleocapsid protein gene, partial cds.
- KU139536.1: Tula virus isolate 11_1373_Marv nucleocapsid protein gene, partial cds.
- KU139535.1: Tula virus isolate 13_0784_Marv nucleocapsid protein gene, partial cds.
- KU139533.1: Tula virus isolate 07_0862_Marv nucleocapsid protein gene, partial cds.
- KU139531.1: Tula virus isolate 10_0029_Marv nucleocapsid protein gene, partial cds.
- KU139530.1: Tula virus isolate 10_0023_Marv nucleocapsid protein gene, partial cds.
- KU139529.1: Tula virus isolate 09_2375_Marv nucleocapsid protein gene, partial cds.
- KU139528.1: Tula virus isolate 09_2324_Marv nucleocapsid protein gene, partial cds.
- KT885046.1: Sin Nombre virus strain CC107/POR segment S, complete sequence.
- KJ420559.1: Imjin virus segment S nucleoprotein gene, complete cds.
- JQ690282.1: Sin Nombre virus isolate 2 nucleocapsid protein gene, complete cds.
- HQ697347.1: Tula virus isolate GER/09/2155/Arv nucleocapsid protein gene, partial cds.
- HQ697346.1: Tula virus isolate GER/09/815/Arv nucleocapsid protein gene, partial cds.
- HQ697344.1: Tula virus isolate GER/08/712/Arv nucleocapsid protein gene, partial cds.
- GU300137.1: Tula virus strain dpz06-29 nucleocapsid protein gene, partial cds.
- GU300135.1: Tula virus strain dpz06-1 nucleocapsid protein gene, partial cds.
- EU439952.1: Tula virus strain Sennickerode Sen05/222 nucleocapsid protein gene, partial cds.
- EU439951.1: Tula virus strain Sennickerode Sen05/205 nucleocapsid protein gene, complete cds.
- EU439950.1: Tula virus strain Sennickerode Sen05/204 nucleocapsid protein gene, complete cds.
- EU439949.1: Tula virus strain Sennickerode Sen05/175 nucleocapsid protein gene, complete cds.
- EU439948.1: Tula virus strain Sennickerode Sen05/174 nucleocapsid protein gene, partial cds.
- EU439946.1: Tula virus strain Sennickerode Sen05/121 nucleocapsid protein gene, partial cds.
- DQ768143.1: Tula virus strain Brandenburg Mu137/05 nucleocapsid protein (N) gene, partial cds.
- DQ662087.1: Tula virus strain Brandenburg Mu 46/04 nucleocapsid protein (N) gene, partial cds.
- U54575.1: Muleshoe hantavirus nucleocapsid protein (N) and nonstructural protein (NSx) mRNA, complete cds.

- AJ223600.1: Tula virus genomic RNA for nucleocapsid protein, (strain Tula/Koziky/5247Ma/94).
- Y13984.1: Tula virus partial genomic RNA for nucleocapsid protein, strain (Tula/D540/Ma/95).
- U95304.1: Tula virus O20 nucleocapsid protein gene, partial cds.
- U95303.1: Tula virus O52 nucleocapsid protein gene, partial cds.
- U95302.1: Tula virus O24 nucleocapsid protein gene, partial cds.
- Z69991.1: Tula virus genomic RNA for nucleocapsid protein, strain (Tula/Moravia/5302v/95).
- Z49915.1: Tula virus segment S, strain Tula/Moravia/5302Ma/94, genomic RNA.
- Z48741.1: Tula virus segment S, strain Tula/Moravia/5294Ma/94, genomic RNA.
- Z48574.1: Tula virus segment S, strain Tula/Moravia/5293Ma/94, genomic RNA.
- Z48573.1: Tula virus segment S, strain Tula/Moravia/5286Ma/94, genomic RNA.
- L37904.1: Sin Nombre virus (NM R11) RNA S segment encoding nucleocapsid protein (N protein), complete cds.
- L25784.1: Sin Nombre virus S segment nucleocapsid protein gene, complete cds.
- U10890.1: Four Corners hantavirus Sweetwater Canyon 1 nucleocapsid protein gene, partial cds.
- L33683.1: Pulmonary syndrome hantavirus (Convict Creek 107) S RNA segment.

>seq264:2_08-08-2013_(VirIDv3r520827) Start=12 End=412
nnnnnnnnnnnnnnncnnncnnnnnnnntnnnnnnnnnnnnnnnnnnnnnnnnnnnnnnnnnnnncnnncnnnnnnnnnnnnn
nnnnnnnnnngnnnnnnnnnnnnnnnngnnnnnnnnnnnggnnnnnnccnnngnnngnnncnnnnnnnnnnnnnnnn
nnnnnnnncnnnnncnnncnncnnncncnnncnnnnnnnncnnngnanngnnaccaggtaggtttagaacaattgtttgtggnnncnnn
nnnncnnnnantntncacannnncntcnnnagtcctgtcatgnnnnngnnnnnnnntnnnngnnnnnnngnnnnnnnnnnnnnn
nnnnnnnnnnnnnnnnnnnnnnnnnnnnnnnnnnnnnnnnnnnnnnngnnnnncnn
(A) CR (85/401): 21.2%
(B)
Max score: 55.4
Total score: 55.4
Query coverage: 7%
E value: 0.001
Identity: 100%
- AJ223601.1: Tula virus genomic RNA for nucleocapsid protein, (strain Tula/Koziky/5276Ma/94).
- AJ223600.1: Tula virus genomic RNA for nucleocapsid protein, (strain Tula/Koziky/5247Ma/94).
- Y13984.1: Tula virus partial genomic RNA for nucleocapsid protein, strain (Tula/D540/Ma/95).
- Y13981.1: Tula virus partial genomic RNA for nucleocapsid protein, strain (Tula/Kosice676/Ma/95).
- Y13979.1: Tula virus genomic RNA for nucleocapsid protein, strain (Tula/Kosice144/Ma/95).
- Z69991.1: Tula virus genomic RNA for nucleocapsid protein, strain (Tula/Moravia/5302v/95).
- Z49915.1: Tula virus segment S, strain Tula/Moravia/5302Ma/94, genomic RNA.
- Z48741.1: Tula virus segment S, strain Tula/Moravia/5294Ma/94, genomic RNA.
- Z48574.1: Tula virus segment S, strain Tula/Moravia/5293Ma/94, genomic RNA.
- Z48573.1: Tula virus segment S, strain Tula/Moravia/5286Ma/94, genomic RNA.

>seq265:2_08-08-2013_(VirIDv3r520827) Start=12 End=412
tcggccagnncattncaggctttgcacttgcaataatcttgaaggcattgtatatgctgtcaactagaggnaggcaaacaatcaagnaaaataaggg
gacaagaatccggtttaaggatgacagctcatatgaagnantcaatggcattngacgcccgaaacatctgtatgtgtctatgccaacagcccagtcta
ccntgaaagccgatgaattgncaccaggtaggtttagaacaattgtttgtggactctttcctgctcaaattatgcacagaaacatcataagtcctgtcat
gggtgtgattggattttctttttcgttaaagattggcctgaaaagattgaggagttccttattaaaccctgcccattcctgaagaaaagtggtcctagtaa
ggaa
(A) CR (391/401): 97.5%
(B)
Max score: 688
Total score: 688
Query coverage: 100%
E value: 0.0

Identity: 98%

- Z49915.1: Tula virus segment S, strain Tula/Moravia/5302Ma/94, genomic RNA.

>seq266:2_08-08-2013_(VirIDv3r520827) Start=12 End=412
tcggccagnnnannnnnnnncnnnnnnnnnnnnnnnnnnnnncnnnnnnnnnnnnnnnnnngcnnnnnnngnnnnngnnnnntcn
agnanaatnaggngnnnnnnnnnnnnnnnnnnnnnnnnnnngnnnnnnnnnnnnnnnnnnnnnnnnnnnnnnnnnnnnnncnn
ncnnngngncnnnnnnnnnnccnnnnnnnncnnnnngnncnnnnnnnnnnnnngnnnnnnnnnnnnnnnncnnnnntnnnnnntnn
nnnnnnnnnnnnnntnnnnncnncnnnnncatnagtnnnnnnnnnnnnncggcnnnnnnnnnnnnnnnnnnncnnnngnnnnctg
aanagattgaggagttccttattnnnnnnncnnnnnnnnnannnnnnnncnnnnnnnnnnnnncn
(A) CR (86/401): 21.4%
(B)
Max score: 44.6
Total score: 44.6
Query coverage: 6%
E value: 2.7
Identity: 96%

- AJ223601.1: Tula virus genomic RNA for nucleocapsid protein, (strain Tula/Koziky/5276Ma/94).
- AJ223600.1: Tula virus genomic RNA for nucleocapsid protein, (strain Tula/Koziky/5247Ma/94).
- Y13984.1: Tula virus partial genomic RNA for nucleocapsid protein, strain (Tula/D540/Ma/95).
- Z69991.1: Tula virus genomic RNA for nucleocapsid protein, strain (Tula/Moravia/5302v/95).
- Z49915.1: Tula virus segment S, strain Tula/Moravia/5302Ma/94, genomic RNA.
- Z48741.1: Tula virus segment S, strain Tula/Moravia/5294Ma/94, genomic RNA.
- Z48574.1: Tula virus segment S, strain Tula/Moravia/5293Ma/94, genomic RNA.
- Z48573.1: Tula virus segment S, strain Tula/Moravia/5286Ma/94, genomic RNA.

Origin: Slovenia

TULV Slovenia

>seq265:1_08-08-2013_(VirIDv3r520827) Start=12 End=412
nnnnnnnccnnnncnnnnngnnnnnncnnnncnnnnnattnannnnnnnnnnncnnnnnnnnaactagaggnnnnnngnnnnnnnn
nnnnancnngnggacaagaatccggttcaaanntgncagcncannnnnnnnnnnnnnnnnnnnnnnnacgcncannnnnnnnnnnnn
nnngnnnnnnnnnnnnnnnnnnnnnncnngaaagccgatgaattgacaccaggnngannnnnngntnngnngtgngggnnnnnnnct
gctcngntnatgcacagaaacatcataagtcctgtcatgggngnannnnnnnnnntnnnnnnnnncnnnnnnnggnctgannnannnnnn
cnnngnnnnnnnnnnnnnngnnnnnnnnnnnnnnnnnnnnnnnnnnnnnnnnncn
(A) CR (150/401): 37.4%
(B)
Max score: 78.8
Total score: 123
Query coverage: 32%
E value: 1e-10
Identity: 73%

- KF776874.1: Tula virus isolate HtSi_1087_a1999 nucleocapsid protein gene, partial cds.

Species: Seoul orthohantavirus (SEOV)

Origin: France

Seoul Lyon 892 ***(unknown at the moment of the analysis)***

>seq255:3_26-06-2014_(VirIDv3r520827) Start=12 End=412
taattgtcnnnnnnnnnnnnnnnnnnnngnnnnncatcttgaaggcactgtnnatgntaanaacaagaggnaggcagnnttcgnngnncan
caagnnnnnnnnnnnnnngnnnnangatgncagcncnnntgaggnngncancggaancagaaagnncaancnntngnnngtgtcantg
ccaaatgnnnngnnnnnnnnnnnggnngaaganntaacacctggaagannnnnnnnnnnnnnngtgnncnanatcctgcacaganna
aggcaagganctggtaagccctgtcatgagtgtagttgggtttntggcnctggcaaaagactggacatctagaattgaagaanggctnggcnnn
nnnnnnnngntcatggcngngtcnnnnattgncgggngn
(A) CR (257/401): 64.0%
(B)
Max score: 235
Total score: 235
Query coverage: 90%
E value: 8e-58
Identity: 68%
- KF387725.1: Seoul virus isolate LYON/Rn/FRA/2013/LYO852 segment S, complete sequence.

>seq257:3_26-06-2014_(VirIDv3r520827) Start=12 End=412
taattgncnnnnnnnnnnnnnnnnnnnngnnnnnnnnnnngnnggcncngtnnntgnnnanannnnnnggnaggcnnnnnnnnnn
nnnnnnnnngnnnnnnnnnnnnnnngnncaangangacagctnnnnngnggangncnncgnnnnnnnnnngnnnnnnnnngnnng
nnngncannnccnngtnnnnnngncnncangaaggctgaagagannacacctggaannntcngnnnnncngtatgtgngctatatcctnc
acnganaaaggcaaggannnngnnangnnnngncatgagtgtagttgggtnttnggcacnngnnnaanncnggnnntctagnattgaaga
ntggcntnccnnnnnnnnnnnnntcnnnnnnnnnnnnnnnngnnnnnnnnnn
(A) CR (179/401): 44.6%
(B)
Max score: 127
Total score: 127
Query coverage: 39%
E value: 3e-25
Identity: 72%
- KY807170.1: Seoul orthohantavirus isolate XJ5/2011 nucleocapsid protein gene, complete cds.
- KX064273.1: Seoul hantavirus strain MANTENAY-MONTLIN/Rn/FRA/2015/2015.00179 nucleoprotein (N) gene, complete cds.
- KX064272.1: Seoul hantavirus strain MANTENAY-MONTLIN/Rn/FRA/2015/2015.00173 nucleoprotein (N) gene, complete cds.
- KX064269.1: Seoul hantavirus strain DIJON/Hu/FRA/2014/2014-00056 nucleoprotein (N) gene, partial cds.
- KT897726.1: Seoul virus strain Northeast Baltimore segment S nucleoprotein gene, partial cds.
- KP859512.1: Seoul virus isolate JiangxiXinjianRn-09-2011 nucleocapsid protein gene, complete cds.
- KP859511.1: Seoul virus isolate JiangxiXinjianRn-07-2011 nucleocapsid protein gene, complete cds.
- **KF387725.1: Seoul virus isolate LYON/Rn/FRA/2013/LYO852 segment S, complete sequence.**
- JQ665923.1: Seoul virus strain WuhanRn57 nucleocapsid gene, complete cds.
- JQ665922.1: Seoul virus strain WuhanRn53 nucleocapsid gene, complete cds.
- JQ665921.1: Seoul virus strain WuhanRn25 nucleocapsid gene, complete cds.
- JQ665920.1: Seoul virus strain WuhanRn10 nucleocapsid gene, complete cds.
- JQ665919.1: Seoul virus strain WuhanRf49 nucleocapsid gene, complete cds.
- JQ665918.1: Seoul virus strain WuhanRf33 nucleocapsid gene, complete cds.
- JQ665917.1: Seoul virus strain WuhanRf18 nucleocapsid gene, complete cds.

- JQ665915.1: Seoul virus strain WuhanRf11 nucleocapsid gene, complete cds.
- JQ665914.1: Seoul virus strain WuhanRf08 nucleocapsid gene, complete cds.
- JQ665913.1: Seoul virus strain WuhanRf07 nucleocapsid gene, complete cds.
- JQ665911.1: Seoul virus strain WuhanMm24 nucleocapsid gene, complete cds.
- JQ665910.1: Seoul virus strain WuhanMm13 nucleocapsid gene, complete cds.
- JQ898106.1: Seoul virus strain SEO/Belgium/Rn895/2005 nucleocapsid protein gene, complete cds.
- HQ655963.1: Seoul virus strain MA35 nucleocapsid protein gene, partial cds.
- GQ274944.1: Seoul virus isolate Singapore/06(RN41) nucleocapsid protein mRNA, complete cds.
- AJ878417.1: Seoul virus partial N gene for nucleocapsid protein, genomic RNA.
- AJ427501.1: Hantavirus sp., partial gene for nucleoprotein, from rat strains 041 and 049.
- AJ427500.1: Hantavirus sp., partial gene for nucleoprotein, from rat strains 146 and 038.
- AJ427499.1: Hantavirus sp., partial gene for nucleoprotein, from rat strains 141 and 143.
- AJ427498.1: Hantavirus sp., partial gene for nucleoprotein, from rat strain.

>seq258:3_26-06-2014_(VirIDv3r520827) Start=12 End=412
nnnnnnnnnnnnngnnnnnnntcgtggngnngnnnatcttgaagncactgnacatgntaacancaagaggnaggcagnnntcgnnggnc
aacangnnnnnnnnnnnnnngnncaaggatnacagcnnanntgnngnnnnnnncnnannnnnnnnnnnnnnnnnnnnnnntgtgn
cnnngncnngtnnnnnngnnnnnnngnnggctgaagagannanacctggnnnanncngnnnnnnngtatgtgngctntntcctncacag
anaaaggnnnggnncnnggnnagccctgtcatgagtgtagttgngttttnggnnnngncanaagnctgnacatctagaattgaagaatggctn
nncnnaccnngcnanttcanggcnnnngnnnnnnnnnnnnnnnn
(A) CR (215/401): 53.6%
(B)
Max score: 140
Total score: 140
Query coverage: 44%
E value: 5e-29
Identity: 72%
- KX064273.1: Seoul hantavirus strain MANTENAY-MONTLIN/Rn/FRA/2015/2015.00179 nucleoprotein (N) gene, complete cds.
- KX064272.1: Seoul hantavirus strain MANTENAY-MONTLIN/Rn/FRA/2015/2015.00173 nucleoprotein (N) gene, complete cds.
- KX064269.1: Seoul hantavirus strain DIJON/Hu/FRA/2014/2014-00056 nucleoprotein (N) gene, partial cds.
- KT897726.1: Seoul virus strain Northeast Baltimore segment S nucleoprotein gene, partial cds.
- **KF387725.1: Seoul virus isolate LYON/Rn/FRA/2013/LYO852 segment S, complete sequence.**
- GQ274944.1: Seoul virus isolate Singapore/06(RN41) nucleocapsid protein mRNA, complete cds.
- AJ427501.1: Hantavirus sp., partial gene for nucleoprotein, from rat strains 041 and 049.
- AJ427500.1: Hantavirus sp., partial gene for nucleoprotein, from rat strains 146 and 038.
- AJ427499.1: Hantavirus sp., partial gene for nucleoprotein, from rat strains 141 and 143.
- AJ427498.1: Hantavirus sp., partial gene for nucleoprotein, from rat strain 144.

<u>Origin: United Kingdom</u>

<u>Seoul Cherwell UK</u> ***(unknown at the moment of the analysis)***

>seq255:S_UK_03-10-2013_(VirIDv3r520827) Start=12 End=412
nnnnnnnnnnnnnnnnnnnnatttgtggtcccgatcatcttgaaggcactgnncatgntaacancangnggcngncagnnntcanaggacan
caagnncnngnggnncngntnnaaggannncagcnnnnnnnnnnnngnnanngggaancagnaagcccaaacatctgtntgtgncanng
ccanncncnnannccnnnnnnnngnnnnnagagannncacctggnaganncсncannncngnnnntnnncnnnnnncnnnnncnnnn
nnnnnnnnggnncntgnnnagcccngtcatgngtgnngnnnnnnncnngnnacnggnnnaanncnggacatctagaattgaagaatggc
nngnnngnncnnnnnnnnnnnnnnnnnncnngnnnnnnngccgggagt

(A) CR (212/401): 52.9 %
(B)
Max score: 113
Total score: 161
Query coverage: 58%
E value: 6e-21
Identity: 69%
- KC626089.1: Seoul virus nucleocapsid protein gene, complete cds.

(KC626089.1strain="Cherwell")

>seq257:S_UK_03-10-2013_(VirIDv3r520827) Start=12 End=412
nnnnnnnncnnnnnnnnnnnntntgtggtcccgatcatctngnngncacnnnncntgtnancaacnnnnggcaggcagnnnncnnagnnc
annnngnncnngaggnncagnttcaaggatgncagcnnngnnnnagnngnnanngggnancannnnnnnnnnnnnngngnntgtgtca
angccaaacgnnnnnnccagnatganggcngnagagannacacctggaagannccgcacngcngnatgtgngctntntccnncnnnnnn
nnnnnnnnnnnnnnnnnnngnnnngncntgagtgnngnnnnnnncnngnnnnnggnnnnnnncnggnnntctagaattgaagant
ggcnnnccngnnnnnnnnnnnnnnnnggnnnncnnnngnnngnnnnnnnnt
(A) CR (193/401): 48.1%
(B)
Max score: 91.5
Total score: 91.5
Query coverage: 58%
E value: 2e-14
Identity: 61%
- KC626089.1: Seoul virus nucleocapsid protein gene, complete cds.

(KC626089.1strain="Cherwell")

>seq258:S_UK_03-10-2013_(VirIDv3r520827) Start=12 End=412
ngnnnnnncnnnnnnnnnnnnnntntggtcccgatcatcttgaaggcacngnncangtnnacagcnngagncnnncagnnntcanaggac
ancaagnnnntgaggnncagnttcaaggatgncagnnnnnnnnnannnnnnnnngnannnannnnnnnnnnnnnntctgtnngtgncaa
ngccanacgnnnnnncnnnnnngnnggctgnagagannncacctnnnagantccgcanngcngtatgtgngctnnntccnnnnnnnnn
nnnnnnnnnnnncnnngnnngccntgtcatgagtgtngnnnnnnncncnnnnnnggnanaagnctggacatctagaattgaagaatgncn
ngnnngnncnngcaannnnnnnccnnnngnannnngnnncgnnnnt
(A) CR (207/401): 51.6%
(B)
Max score: 86.0
Total score: 136
Query coverage: 33%
E value: 9e-13
Identity: 74%
- MG764079.1: Seoul orthohantavirus strain SEOV/NL/Rn2147/2016 segment S nucleocapsid protein gene, complete cds.
- MG764078.1: Seoul orthohantavirus strain SEOV/NL/Rn2125/2016 segment S nucleocapsid protein gene, complete cds.
- KX064271.1: Seoul hantavirus strain TURCKHEIM/Rn/FRA/2016/2016.00033 nucleoprotein (N) gene, complete cds.
- KX064270.1: Seoul hantavirus strain TURCKHEIM/Hu/FRA/2016/2016.00044 nucleoprotein (N) gene, complete cds.
- KX079476.1: Seoul hantavirus isolate IR162 nucleocapsid protein gene, complete cds.
- KX079473.1: Seoul hantavirus isolate IR33 nucleocapsid protein gene, complete cds.
- KX079470.1: Seoul hantavirus isolate IR473 nucleocapsid protein gene, complete cds.
- KM948598.1: Seoul virus strain Humber segment S, complete sequence.
- **KC626089.1: Seoul virus nucleocapsid protein gene, complete cds.**
- JX879769.1: Seoul virus strain Humber segment S, complete sequence.

<u>Virus (species): Topografov hantavirus</u>

<u>Origin Finland</u>

<u>TOPV laboratory strain</u>

>seq228:4_14-08-2013_(VirIDv3r520827) Start=12 End=412
nnnnnnnngngnnnnnnnncnncnnnnnnnnnnnnnnnnnanncnnnnnnnnnnnnnncnnnnnnnnnnnnncnnnnnnnnnnnn
aagnnnnnnnnnnncnnnnnnnngnnnnnnnnnnnnnnnnngnnnnnncgnnncnnnnnnnnnnnnnnnnnnnnnnngnn
nnnnnnnnnctatgccaacagcacagtctacaannnnnnnnnnnnnnnnannnnnnnnnccncnnnnnnnnnnngnnnnnnnnnnn
nnnnnnnnnnnnngncnnnncnngngnnnnnnnnnnnnnnnnnnnncnnnnnnnnnnnnnannnnnnnnnnnnnnnngnnnn
gnnnnnnnnnnnnnnngnnnngnnnnnnnnnnnnnnnnnnnnnnnnnnnnnnnnnnnnnccnnn
(A) CR (60/401): 15.0%
(B)
Max score: 44.6
Total score: 44.6
Query coverage: 5%
E value: 2.7
Identity: 100%
- AJ011646.1: Topografov hantavirus S segment gene for N protein.

>seq234:4_14-08-2013_(VirIDv3r520827) Start=12 End=238
nnnnnnnnnnnnncnnnngnnnnnnnnnnnnnnnngannngnngtnnnnnnnngnnnnnnncnnnnnngnagnnnnnncnnn
cnnnnnnannnngatgatnnngnnnnnnntanannnnannnnngnnngnagncagtgtcagcacnggaggacaaacttgcagacttnn
annnnnnnngnnnnnnnnngnnnnnnnnnnnnnnnnngnncnnnnggnnnn
(A) CR (70/227): 30.8%
(B)
Max score: 60.8
Total score: 60.8
Query coverage: 16%
E value: 2e-05
Identity: 95%
- KY364995.1: Puumala orthohantavirus isolate Ardennes_2011_87 nucleoprotein gene, complete cds.
- KT247593.1: Puumala virus isolate PUUV/Ardennes/Mg75/2011 segment S nucleoprotein gene, complete cds.
- KT247592.1: Puumala virus isolate PUUV/Ardennes/Mg156/2011 segment S nucleoprotein gene, complete cds.
- AJ277030.1: Puumala virus RNA for nucleocapsid protein (N gene), strain Thuin/33Cg/96.
- AJ277076.1: Puumala virus RNA for nucleocapsid protein (n gene), strain CG14445.
- AJ277075.1: Puumala virus RNA for nucleocapsid protein (n gene), strain CG14444.
- AJ277034.1: Puumala virus RNA for nucleocapsid protein (N gene), strain Couvin/59Cg/97.
- AJ277033.1: Puumala virus RNA for nucleocapsid protein (N gene), strain Momignies/55Cg/96.
- AJ277032.1: Puumala virus RNA for nucleocapsid protein (N gene), strain Momignies/47Cg/96.
- AJ277031.1: Puumala virus RNA for nucleocapsid protein (N gene), strain Montbliart/23Cg/96.
- AJ238779.1: Puumala virus mRNA for nucleocapsid protein.
- **AJ011646.1: Topografov hantavirus S segment gene for N protein.**

>seq235:4_14-08-2013_(VirIDv3r520827) Start=12 End=412
nnnnnnnnnnnnnnnnnnnnnnnnncngnnnnnnnnnnnnncnnnnnnnnnncnnnnnnnnnnnnnnnccnnnnnnngnnnn
nnnnannnnnnnnncnnnnnnnccnnnnnnggctnccnnnnnnnngannnnnnnnnnnnncnnnnncnnnnncnaccaaaacatctct
nngtannnnnnntcaacagcacagtctacaatgnngnnnnngnngnnnnnnnnnnnnnncnnnnnnnnnnnnncnngcngcctatttcct

nnnnncnnnnnnnnnnnnnnnnntcatgagccccgtgntnncnnnnnnnnnnnnnnnnttngnnnnngnnnnnnnnnnnnnnncnnnnng
nnnnnnngnnnnnnnnnngnnnnngnnnctttattaancnngaanngnnnnnnnnnnnn
(A) CR (118/401): 29.4%
(B)
Max score: 50.0
Total score: 50.0
Query coverage: 11%
E value: 0.064
Identity: 80%
AJ011646.1: Topografov hantavirus S segment gene for N protein.
